# Platinum‐Catalysed Hydrofluorination of Alkynes at Room Temperature Promoted by a Fluoride Shuttle

**DOI:** 10.1002/anie.202512181

**Published:** 2025-07-23

**Authors:** Ouchan He, Froze Jameel, Hannah Flammang, Smrithi Suresh Babu, Martin Kaupp, Thomas Braun

**Affiliations:** ^1^ Department of Chemistry Humboldt‐Universität zu Berlin Brook‐Taylor‐Str. 2 12489 Berlin Germany; ^2^ Department of Chemistry, Technische Universität Berlin Theoretische Chemie/Quantenchemie Sekr. C7, Straße des 17. Juni 135 10623 Berlin Germany

**Keywords:** Alkynes, Fluorine chemistry, Fluoroalkenes, Hydrofluorination, Platinum

## Abstract

Hydrofluorination of alkynes provides a synthetic route to access fluoroalkenes, a class of compounds with wide applications in chemical research. Herewith, we describe an exceptional hydrofluorination process of alkynes catalysed by Pt(II) complexes at room temperature. Various Pt(II) dichloride complexes bearing chelating phosphines were synthesised and studied towards their catalytic behaviour. Mechanistic investigations suggest the involvement of a dicationic Pt(II) bis(alkyne) species as well as a cationic *ß*‐fluorovinyl Pt(II) complex in the catalytic cycle. Remarkably, the hydrofluorination is enabled by fluorinated anions. The corresponding acids BF_3_, HF as well as PF_5_ act as fluoride shuttles to allow for an outer‐sphere fluorination of the metal‐bound substrate. Detailed DFT analyses for BF_3_ show that the hydrofluorination is mediated by complexes between the fluorinated anion and HF. This lowers the hydrofluorination barriers sufficiently to outcompete the simultaneously occurring, more exergonic cyclisation of two coordinated alkynes to a structurally characterised cycloallyl complex. The catalytic system can be applied to a wide substrate scope to generate fluoroalkenes bearing bulky alkyl, aryl and electron withdrawing groups, such as ester and carbonyl substituents.

## Graphical abstract

This work presents an air and moisture tolerant platinum‐catalysed hydrofluorination system that gives selectively (*Z*)‐fluoroalkenes. Experimental and computational investigation on the mechanism allowed for the identification of catalytic species. The hydrofluorination is mediated by complexes between the fluorinated anion and HF, resulting into BF_3_, PF_5_ and HF serving as fluoride shuttles.

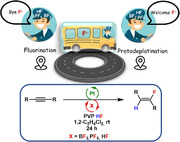

## Introduction

Organofluorine compounds have been found to have a wide range of applications in materials science^[^ ^1^, ^2^, ^3^ ^]^ as well as in agrochemical^[^ ^4^ ^,^ ^5^ ^]^ and pharmaceutical industry.^[^ ^6^, ^7^, ^8^ ^]^ The selective monofluorination of an organic molecule has attracted enormous research interest.^[^ ^9^ ^]^ Monofluorinated fluoroalkenes are attractive bioisosters in medicinal chemistry by replacing amide functional groups.^[^ ^10^ ^]^ This can result in advantageous properties such as an improved lipophilicity, metabolic stability, binding selectivity and physicochemical properties.^[^ ^11^, ^12^ ^]^ Hydrofluorination of alkynes offers a direct and effective pathway to access fluoroalkenes. Several approaches have been described in the literature,^[^ ^13^, ^14^ ^]^ although transition metal‐catalysed systems appear to be the most prevalent.^[^ ^15^, ^16^ ^]^ Recent advances in this field involve gold‐enabled hydrofluorination of unactivated alkynes as the most extensively studied and prominent catalytic system.^[^ ^15^ ^]^

The first examples for catalytic hydrofluorination reactions of alkynes were reported by Sadighi and co‐workers using a Au(I) precatalyst and NEt_3_·HF as the HF source.^[^ ^17^ ^]^ In the following years, the scope of alkyne substrates was extended by the research groups of O'Hagan, Nolan, Toste as well as Paquin including internal aryl,^[^ ^18^ ^]^ electron‐deficient^[^ ^19^ ^]^ and terminal alkynes,^[^ ^20^, ^21^ ^]^ although the Au precatalysts are often air‐sensitive and the conversions required additives, such as additional proton sources.^[^ ^22^ ^]^ While Hammond and co‐workers employed DMPU·HF,^[^ ^23^ ^]^ Paquin used aqueous HF^[^ ^24^, ^25^ ^]^ as HF source. Crimmin and co‐workers developed a gold‐catalysed HF transfer method to access fluoroalkenes at 120 °C within 16 h.^[^ ^26^ ^]^ In addition to the hydrofluorination of unactivated alkynes, He and Zhu described a regioselective *trans*‐hydrofluorination of ynamides catalysed by Cu(I) or Ag(I) complexes.^[^ ^27^ ^]^ Previously, we reported on the first example of platinum‐catalysed hydrofluorination of alkynes at 60 °C within 144 h using a precatalyst bearing indolyl‐substituted phosphine ligands.^[^ ^28^ ^]^ Similar to gold‐based catalytic systems, NEt_3_·HF was employed as the HF source and 2‐chlorobenzoic acid acting as an additive. In 2020, Guo et al. demonstrated a regio‐ and stereoselective stoichiometric hydrofluorination of alkynes to give the corresponding fluoroolefins.^[^ ^29^ ^]^ Pyridinium salts were used as fluorinating reagents and the tetrafluoroborate anions serve as fluorinating species at elevated temperatures (40°C–100 °C).

**Figure 1:**
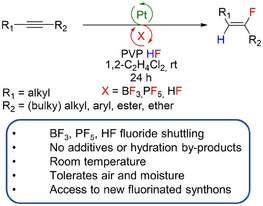
Pt‐catalysed hydrofluorination enabled by fluoride shuttling.

In this study, we report on the development of an exceptional process for the selective *trans*‐hydrofluorination of alkynes to yield fluoroalkenes at room temperature (Figure 1). Mechanistic studies reveal the crucial role of fluorinated anions in the fluorination of an alkyne bound at an electrophilic Pt centre to give intermediate fluorovinyl complexes. Anion regeneration by an HF source ultimately demonstrates that BF_3_, HF or PF_5_ can act as fluoride shuttles to enable access to new fluorinated synthons.

## Results and Discussion

### Development of the Catalytic System and Scope

In order to generate highly electrophilic cationic Pt alkyne complexes^[^ ^30^ ^]^ as intermediate species to achieve hydrofluorination reactions, Pt(II) phosphine complexes were treated with AgBF_4_ and PVP HF (PVP HF = poly[4‐vinylpyridinium poly(hydrogen fluoride)]) in the presence of 3‐hexyne. On using chelating phosphine ligands, the catalytic hydrofluorination of 3‐hexyne was achieved to generate its corresponding monofluoroalkene **2a** at room temperature (Table 1). The results imply a correlation between ligand bite angle and catalytic activity. The reaction of 3‐hexyne and PVP HF in 1,2‐dichloroethane was catalysed by 10 mol% of [PtCl_2_L] (**1**) with 40 mol% of AgBF_4_ and led to the selective formation of (*Z*)‐3‐fluoro‐3‐hexene (**2**a) after 24 h. When using [PtCl_2_(dppm)] (**1‐L1**) (dppm = 1,1‐bis(diphenylphosphino)methane (**L1**)) or [PtCl_2_(dppe)] (**1‐L2**) (dppe = 1,2‐(bis(diphenylphosphino)ethane (**L2**)),^[^ ^31^, ^32^ ^]^ catalytic hydrofluorination of 3‐hexyne to yield **2a** was observed with moderate NMR yields of 68% and 73%, respectively (Table 1, entry 2, 3). The complex [PtCl_2_(PCNP)] (**1‐L3**) (PCNP = 1,2‐bis(diphenylphosphino)‐3‐methyl‐1H‐indole (**L3**)), for which the phosphine exhibits a large bite angle of 88.83(4)° according to X‐ray diffraction analysis (Figure 3a), displays the highest catalytic activity (88%, Table 1, entry 4) within the series. A further increase in bite angle, as found for [PtCl_2_(dppp)] (**1‐L4**) (dppp = 1,3‐bis(diphenylphosphino)propane (**L4**)), [PtCl_2_(dppb)] (**1‐L5**) (dppb = 1,4‐bis(diphenylphosphino)butane (**L5**)), [PtCl_2_(dppf)] (**1‐L6**) (dppf = 1,1′‐bis(diphenylphosphino)ferrocene (**L6**)) and *cis*‐[PtCl_2_(Xantphos)] (**1‐L7**) (Xantphos = 4,5‐bis‐(diphenylphosphino)‐9,9‐dimethylxanthene (**L7**)),^[^ ^33^, ^34^, ^35^, ^36^ ^]^ led to a decrease in yield of the monofluoroalkene **2a** for **1‐L4** (62%, Table 1, entry 5) or to a complete loss of catalytic activity for **1‐L5**, **1‐L6** and **1‐L7** (Table 1, entry 6–8). Small traces of the trans‐isomer (*E*)‐3‐fluoro‐3‐hexene (< 3%) and 3‐chloro‐4‐fluoro‐3‐hexene (< 2%) were always observed, except when using the catalyst **1‐L3**. No catalytic activity was found when employing [PtCl_2_{Ph_2_P(Ind)}_2_] or [PtCl_2_(PPh_3_)_2_] based on non‐chelating phosphine ligands as catalytic precursors (Table 1, entry, 1). Note that the previously reported platinum‐catalysed hydrofluorination of alkynes using [PtMe_2_{Ph_2_P(Ind)}_2_] and 2‐chlorobenzoic acid as additive required rather harsh reaction conditions (60 °C, 144 hours).^[^ ^28^ ^]^ This might be suggestive of a possible in situ formation of the PCNP ligand (**L3**) in this case.^[^ ^37^ ^]^

**Table 1:** Selected examples of Pt(II) dichlorido precatalysts for the hydrofluorination reaction of 3‐hexyne to give (*Z*)‐3‐fluoro‐3‐hexene (**2a**).

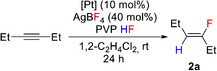				
Entry	[Pt]	P‐Pt‐P bite angle [°]		Yield [%]^a)^
1	[PtCl_2_R_2_] R = Ph_2_P(Ind), Ph_3_P	–		Traces
2	[PtCl_2_(dppm)] (**1‐L1**)	74.27(7)^[^ ^31^ ^]^	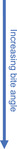	68
3	[PtCl_2_(dppe)] (**1‐L2**)	86.3(2)^[^ ^32^ ^]^		73
4	[PtCl_2_(PCNP)] (**1‐L3**)	88.83(4)		88
5	[PtCl_2_(dppp)] (**1‐L4**)	94.23(9)^[^ ^36^ ^]^		62
6	[PtCl_2_(dppb)] (**1‐L5**)	95.37(5)^[^ ^34^ ^]^		Traces
7	[PtCl_2_(dppf)] (**1‐L6**)	99.3(1)^[^ ^33^ ^]^		Traces
8	[PtCl_2_(Xantphos)] (**1‐L7**)	100.87(8)^[^ ^35^ ^]^		0

^a)^ NMR yields determined by ^19^F NMR spectroscopy using benzotrifluoride as internal standard.

**Figure 2:**
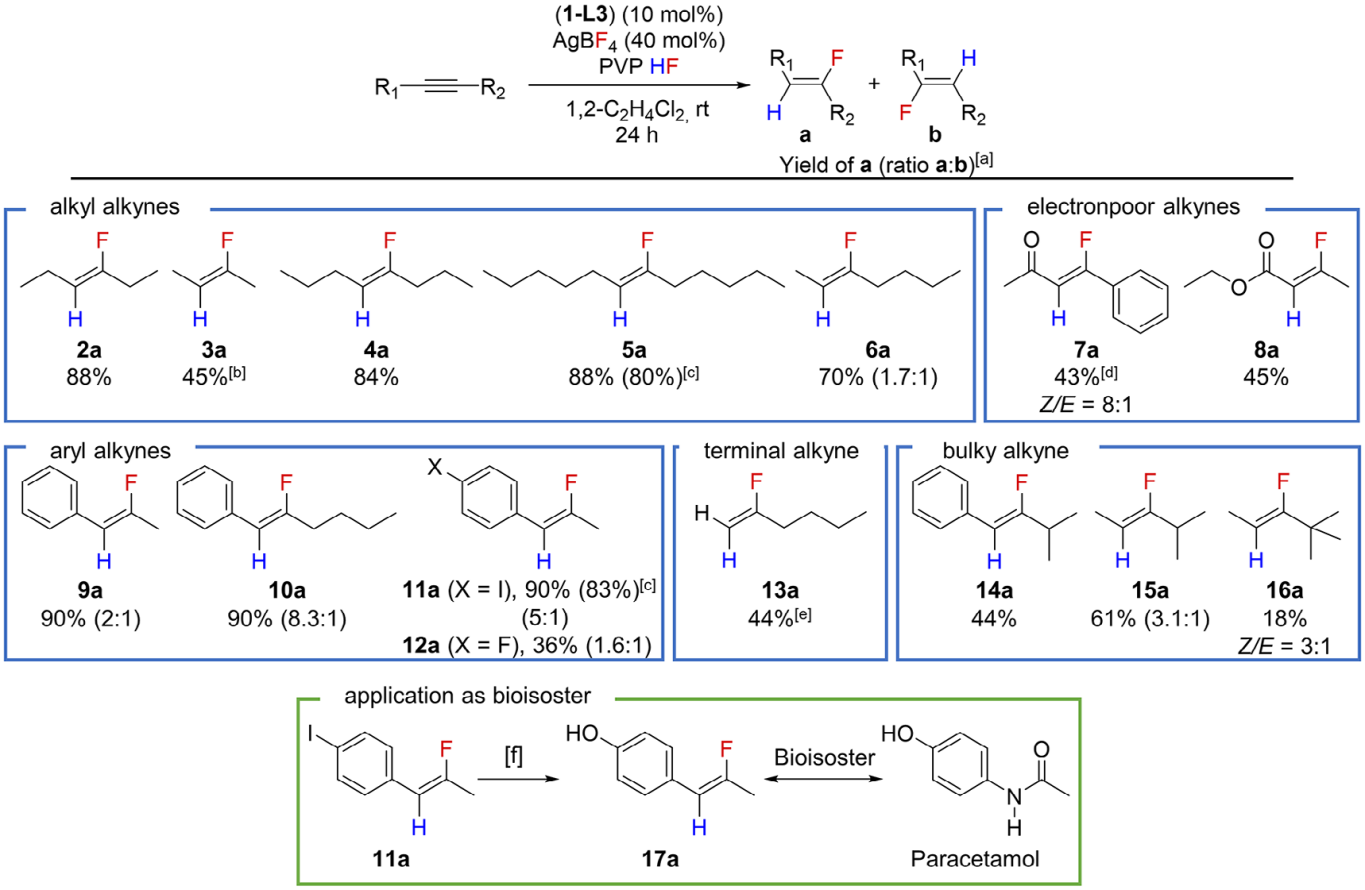
Reaction scope of the Pt‐catalysed hydrofluorination of alkynes under optimised conditions promoted by a fluoride shuttle. NMR yields are calculated by ^19^F NMR spectroscopy of the crude reaction mixture using benzotrifluoride as internal standard. ^a)^ Ratio determined by ^19^F NMR spectroscopy. ^b)^ Reaction was carried out under inert conditions for 72 h. ^c)^ Isolated yield in parentheses. ^d)^ Traces of *gem*‐difluoroalkene were observed. ^e)^ 60 °C for 144 h. ^f)^ 3 eq. CsOH·H_2_O, 0.1 eq. CuI, 0.5 eq. dibenzoylmethane, H_2_O/DMSO (1/1), 24 h, 130 °C.

The scope of the catalytic hydrofluorination was then explored on using [PtCl_2_(PCNP)] (**1‐L3**) as precatalyst at room temperature with reaction times of 24 h (Figure 2). Reactions were carried out under air and reagents were used without further drying or degassing. A range of alkyl alkynes were successfully hydrofluorinated exclusively to their respective *cis*‐fluoroalkenes (**2a**‐**6a**), and with an isolated yield of 80% for (*Z*)‐6‐fluorododec‐6‐ene (**5a**). Hydrofluorination of 2‐heptyne gave (*Z*)‐3‐fluoro‐2‐heptene (**6a**) and (*Z*)‐2‐fluoro‐2‐heptene (**6b**) with a regioselectivity of 1.7:1 in good yield. The catalytic system also exhibits good functional group tolerance, enabling regioselective hydrofluorination of electron‐deficient alkynes bearing carbonyl and ester groups, affording **7a** and **8a** in moderate yields. However, for 4‐phenyl‐3‐butyn‐2‐one a *Z*:*E* isomer mixture of 8:1 as well as formation of the corresponding *gem*‐difluoroalkane was observed. Different aryl alkynes were evaluated showing stereoselective *trans*‐hydrofluorination giving **9a**‐**12a** with yields up to 90%. Electron‐withdrawing groups at the *para* position of aryl alkynes were successfully implemented in the catalysis, whereas no catalytic activity was observed when using electron donating groups such as ‐OMe or ‐OH (see Table S1 in SI). The terminal alkyne 1‐hexyne delivered **13a** in moderate yields after 144 h at 60 °C. Remarkably, substrates bearing bulky substituents, such as *i*Pr or *t*Bu groups proved to be suitable substrates at room temperature to yield **14a** and **15a** and a *Z*:*E* isomer mixture of 3:1 of **16a**, whilst for a previously reported hydrofluorination system,^[^ ^13^ ^]^ the use of 4‐methyl‐2‐pentyne was reported to be unsuccessful. Although the hydrofluorination of 4,4‐dimethyl‐2‐pentyne does not run under catalytic conditions, the generation of the fluoroalkenes **15a**‐**16a** demonstrates the feasibility to access new fluorinated molecules. This is of particular interest considering the role of fluoroalkenes as potential bioisosteric surrogate in drug discovery.

As a proof‐of‐concept, the catalytic system was applied for the synthesis of (*Z*)‐4‐(2‐fluoroprop‐1‐en‐1‐yl)phenol (**17a**) which represents a bioisoster of *para*‐acetylaminophenol (paracetamol). Paracetamol is one of the most commonly used analgesic and antipyrectic drug.^[^ ^38^ ^]^ Since 1977, it is included on the World Health Organisation's list of Essential Medicines^[^ ^39^ ^]^ signifying its importance in global healthcare and widespread usage within the pharmaceutical industry. Catalytic hydrofluorination of 1‐iodo‐4‐(prop‐1‐yn‐1‐yl)benzene gave (*Z*)‐1‐(2‐fluoroprop‐1‐en‐1‐yl)‐4‐iodobenzene (**11a**) which then can undergo hydroxylation to form **17a**. This demonstrates an efficient and simple way for the synthesis of monofluoroalkenes as amide bioisosters.

### Cycloallyl Complex Formation

**Scheme 1:**
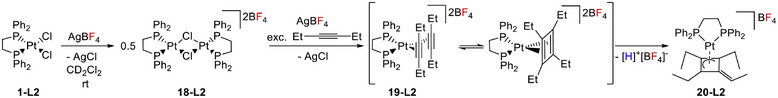
Stoichiometric model reaction showing the formation of the cationic Pt(II) complex **20‐L2** bearing an anionic cycloallyl ligand, derived from the dicationic Pt(II) bis(alkyne) complex **19‐L2**.

**Figure 3:**
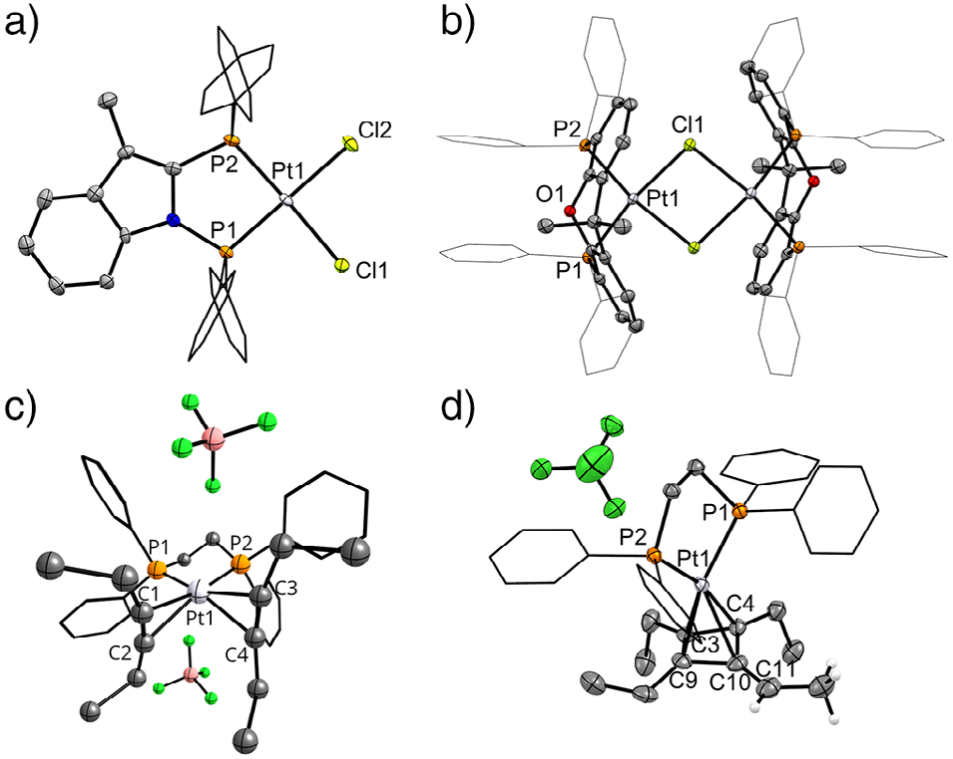
Phenyl substituents are depicted as wireframes. Carbon‐bound hydrogen atoms were omitted for clarity. Thermal ellipsoids of the molecular structures are drawn at 50% probability level. a) Structure of [PtCl_2_(PCNP)] (**1‐L3**). Selected bond angle [°]: P1‐Pt1‐P2 88.83(4). b) Structure of *cis*‐[PtCl(Xantphos)]_2_[BF_4_]_2_·6CH_2_Cl_2_ (**18‐L7**·6CH_2_Cl_2_). CH_2_Cl_2_ and BF_4_^−^ anions are omitted for clarity. Selected bond angle [°]: P1‐Pt1‐P2 99.67(3). c) DFT‐optimised structure of [Pt(*C*,*C*‐η^2^‐C_2_H_5_ *C*≡*C*C_2_H_5_)_2_(dppe)][BF_4_]_2_ (**19‐L2**) at ωB97M‐V/def2‐TZVP//BP86‐D3(BJ)/def2‐TZVP level. d) Structure of [Pt(η^3^‐(C_2_H_5_ *C*)_3_ *C*═C_2_H_4_)(dppe)][BF_4_] (**20‐L2**). Selected bond lengths [Å]: Pt1─–C3 2.240(4), Pt1─–C4 2.169(4), Pt1─–C9 2.206(4), Pt1─–C10 2.393(4), C3─C4 1.438(6), C3─C9 1.451(6), C4─C10 1.515(6), C9─C10 1.502(6), C10─C11 1.346(7).

To gain further insight into the mechanism, stoichiometric model reactions were carried out using [PtCl_2_(dppe)] (**1‐L2**) as a suitable model complex (Scheme 1). It has been reported before that a reaction of **1‐L2** with AgBF_4_ leads to the formation of AgCl precipitate and the binuclear complex [PtCl(dppe)]_2_[BF_4_]_2_ (**18‐L2**)^[^ ^40^, ^41^ ^]^ (Scheme 1). Even for **1‐L7** bearing the bulky Xantphos ligand **L7**, dimerisation was observed resulting in the formation of *cis*‐[PtCl(Xantphos)]_2_[BF_4_]_2_ (**18‐L7**). Suitable single crystals for X‐ray diffraction of **18‐L7** were obtained and the molecular structure is depicted in Figure 3b.

Furthermore, NMR monitoring experiments during the catalytic hydrofluorination revealed signals in the ^31^P{^1^H} NMR spectra for the dimer **18‐L2** and a cationic cycloallyl complex [Pt(η^3^‐(C_2_H_5_ *C*)_3_ *C*═C_2_H_4_)(dppe)][BF_4_] (**20‐L2**) (Scheme 1), whereas signals for the dimer diminishes and the signals for the cycloallyl complex increased over time. To the best of our knowledge, **20‐L2** represents the first isolated and stable complex featuring a cycloallyl ligand. Prior to this work, such complexes were proposed as intermediate in a nickel‐catalysed process at 120 °C by the Inoue group,^[^ ^42^, ^43^ ^]^ and at palladium in the presence of a base.^[^ ^44^ ^]^ The ^31^P{^1^H} NMR spectrum of the Pt cycloallyl complex **20‐L2** displays two doublets with ^195^Pt satellites with a ^2^ *J*_P,P_ coupling constant of 25 Hz, consistent with a *cis* disposition of the phosphorus nuclei.^[^ ^45^, ^46^, ^47^ ^]^ Large ^1^ *J*_P,Pt_ coupling constants of ∼4080 Hz for each phosphorus nuclei suggest the presence of a ligand with a weak *trans* influence in the *trans*‐position. The ^1^H NMR spectrum revealed signals at δ = 5.12 ppm (q+sat.) and δ = 1.52 ppm (d+sat.), which can be assigned to the vinylic proton and the methyl group of the ethylene unit, respectively. Interestingly, both signals exhibit ^195^Pt satellites with *J*_H,Pt_ coupling constants of approximately 10 Hz. This suggests through‐space interactions of the ethylene group with the platinum centre in solution, which was further validated by ^1^H,^195^Pt HMBC NMR experiments (see SI). Slow vapour diffusion of Et_2_O into a saturated solution of [Pt(η^3^‐(C_2_H_5_ *C*)_3_ *C*═C_2_H_4_)(dppe)][BF_4_] (**20‐L2**) in CH_2_Cl_2_ yielded single crystals suitable for X‐ray diffraction. The C3─C4 and C3─C9 bond lengths of 1.438(6) and 1.451(6) Å (Figure 3d) are consistent with a significant degree of π‐electron delocalisation within the ligand, similar to allyl ligands.^[^ ^48^, ^49^ ^]^ The hydrogen atom at the double bond was located in the difference Fourier map.

Mechanistically, the formation of **20‐L2** might proceed via a dicationic Pt(II) bis(alkyne) complex [Pt(*C*,*C*‐η^2^‐C_2_H_5_ *C*≡*C*C_2_H_5_)_2_(dppe)][BF_4_]_2_ (**19‐L2**) followed by a metal‐mediated cycloaddition of the alkyne ligands to form a cyclobutadiene complex, which is then deprotonated. DFT optimisations provide the structure of the dicationic Pt(II) bis(alkyne) complex **19‐L2** (Figure 3c). The results indicate a mono‐dentate fluoride coordination of two tetrafluoroborate anions to the platinum metal centre. Both metal‐bound alkyne ligands are in a perpendicular coordination mode with respect to the plane defined by the platinum and phosphorus atoms. The alkyne ligands exhibit close to linear structures (163.39 °–165.50 °), indicating a weak π‐acceptor interaction with the metal centre.^[^ ^50^ ^]^

To further understand the role of dimer **18‐L2** and cycloallyl complex **20‐L2**, they were isolated and tested as precatalysts. It was found that the dimer **18‐L2** exhibits good catalytic activity, with only 20 mol% of AgBF_4_ (instead of 40 mol%) being sufficient for catalytic formation of **2a** (Table 2, entry 1, 2). This suggests that both chlorido ligands of the Pt(II) dichlorido precatalyst **1‐L2** must be abstracted for the generation of the catalytically active species. On the other hand, the cycloallyl complex **20‐L2** does not exhibit any catalytic activity in for hydrofluorination (Table 2, entry 3, 4). These results suggest that the dimer **18‐L2** serves as a precatalyst and the cycloallyl complex **20‐L2** is an off‐cycle product. Similar observations were made for the corresponding complexes bearing the PCNP ligand **L3**.

**Table 2:** Testing **18‐L2** and **20‐L2** as precatalyst for the hydrofluorination reaction of 3‐hexyne to **2a**.

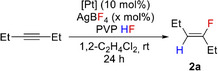			
Entry	[Pt]	AgBF_4_ [mol%]	Yield [%]^a)^
1	[PtCl(dppe)]_2_[BF_4_]_2_ (**18‐L2**)	0	traces
2	[PtCl(dppe)]_2_[BF_4_]_2_ (**18‐L2**)	20	72
3	[Pt(η^3^‐(Et*C*)_3_ *C*═C_2_H_4_)(dppe)][BF_4_] (**20‐L2**)	0	traces
4	[Pt(η^3^‐(Et*C*)_3_ *C*═C_2_H_4_)(dppe)][BF_4_] (**20‐L2**)	20	traces

^a)^ NMR yields determined by ^19^F NMR spectroscopy using benzotrifluoride as internal standard.

### Experimental Studies on the Fluorination Step

**Figure 4:**
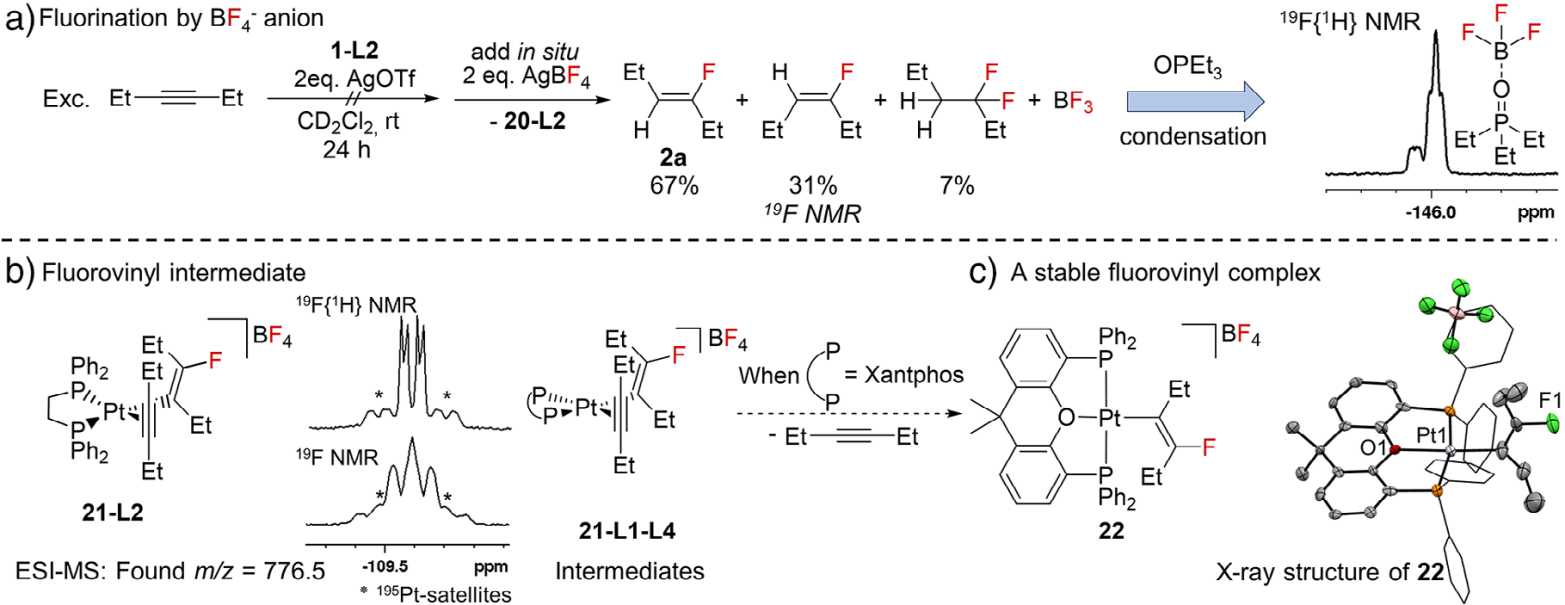
a) Model reactions revealing fluorination by the BF_4_^−^ anion forming BF_3_, which gives in presence of OPEt_3_ BF_3_·OPEt_3_. b) Selected NMR data of the intermediate [Pt(EtC═CFEt)(η^2^‐Et*C*≡*C*Et)(dppe)][BF_4_] (**21‐L2**) during catalysis. ^19^F{^1^H} (top) and ^19^F (bottom) NMR spectrum. c) [Pt(EtC═CFEt)(κ^3^‐Xantphos)][BF_4_] (**22**) and its molecular structure.

Remarkably, the in situ generation of the dicationic Pt bis(alkyne) complex [Pt(*C*,*C*‐η^2^‐C_2_H_5_ *C*≡*C*C_2_H_5_)_2_(dppe)][OTf]_2_ bearing OTf^−^ counter anions did not promote hydrofluorination of 3‐hexyne. However, upon addition of AgBF_4_ in order to exchange the OTf^−^ to BF_4_^−^ anions, formation of fluoroalkenes was observed. Driven by the highly electrophilic nature of the cyclobutadiene complex, it itself acts as a Brønsted acid by formally releasing [H]^+^[BF_4_]^−^, which can then lead to the fluorination of 3‐hexyne producing monofluoroalkenes and traces of *gem*‐difluoroalkane in a quantitative conversion with respect to **1‐L2** (Scheme 1, Figure 4a). In addition, the formation of BF_3_ was detected, which is supported by the formation of BF_3_·OPEt_3_ when condensing the reaction solution into a J. Young NMR tube loaded with OPEt_3_. Distinct coupling patterns of BF_3_·OPEt_3_ were found in the ^19^F{^1^H} NMR spectrum (Figure 4a).^[^ ^51^ ^]^ These observations provide evidence for the involvement of the non‐innocent BF_4_^−^ anion in the fluorination process.^[^ ^13^, ^29^, ^52^, ^53^, ^54^, ^55^ ^]^

This was further tested by using different silver compounds in the catalytic hydrofluorination of 3‐hexyne in order to generate catalytically active Pt species from **1‐L2** bearing different perfluorinated and non‐perfluorinated anions (Table 3). High to moderate yields of the fluoroalkene **2a** were obtained when employing AgF, AgFHF, AgBF_4_ and AgPF_6_ (Table 3, entry 1–4). However, using SbF_6_^−^ as counter anion led to a decrease in yield of **2a** (Table 3, entry 5). This is likely due to the high Lewis acidity of SbF_5_, which makes fluorination by SbF_6_^−^ unfavourable. Low to no conversion was obtained when generating cationic platinum species that have anions which are no fluoride source, such as OTf^−^ or BAr^F^_4_^−^ (Table 3, entry 6, 7). Similar results were obtained when employing **1‐L3** as precatalyst (see SI). Note that a catalytic fluorination reaction involving fluoroborate, leading to a formal C─CF_3_ reductive elimination from a difluoroalkyl Au(III) complex, was reported by Toste and co‐workers in 2017.^[^ ^54^ ^]^

**Table 3:** Selected examples for the influence of different silver compounds on the hydrofluorination reaction of 3‐hexyne to form **2a** using **1‐L2** as a precatalyst.

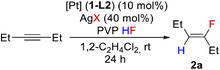							
Entry	1	2	3	4	5	6	7
AgX	AgF	AgFHF	AgBF_4_	AgPF_6_	AgSbF_6_	AgOTf	AgBAr^F^_4_
Yield [%]^a)^	72	73	73	58	25	17	0

^a)^ NMR yields determined by ^19^F NMR spectroscopy using benzotrifluoride as internal standard.

Whilst monitoring the catalytic hydrofluorination with [PtCl_2_(dppe)] (**1‐L2**) as precatalyst, an additional set of signals in the ^31^P{^1^H} NMR spectrum was assigned to the cationic platinum complex [Pt(EtC═CFEt)(η^2^‐Et*C*≡*C*Et)(dppe)][BF_4_] (**21‐L2**) bearing a *β*‐fluorovinyl as well as an alkyne ligand. This complex was identified as a presumable intermediate of the catalysis, which is consistent with prior assumptions regarding the presence of fluorovinyl complexes in gold‐catalysed hydrofluorination systems.^[^ ^15^, ^20^, ^26^ ^]^ **21‐L2** exhibits two doublets of doublets with ^195^Pt satellites in the ^31^P{^1^H} NMR spectrum. The signal at δ = 48.2 ppm can be assigned to the phosphorus nuclei in the *trans* position to the *β*‐fluorovinyl group due to a ^1^ *J*_P,Pt_ coupling constant of 1727 Hz, whilst the signal at δ = 44.7 ppm can be attributed to the phosphorus nuclei in the *trans* position to the alkyne ligand (^1^ *J*_P,Pt_ = 4027 Hz). The multiplicity of a doublet of doublets arises from the coupling to each other (^2^ *J*_P,P_ = 3 Hz) as well as to the fluorine nuclei of the *β*‐fluorovinyl unit with a ^4^ *J*_F,P_ coupling constant of 25 Hz (P *trans* to *β*‐fluorovinyl) and 9 Hz, respectively. The corresponding signal for the *β*‐F atom was observed as doublet of doublets with ^195^Pt satellites in the ^19^F{^1^H} NMR spectrum at δ = −109.58 ppm (^3^ *J*_F,Pt_ = 108 Hz), which is a typical region for *β*‐fluorovinyl species (Figure 4b).^[^ ^45^ ^]^ This finding is further supported by ESI‐MS measurements revealing a peak for [M]^+^ at *m/z* 776.5 that can be assigned to the *β*‐fluorovinyl intermediate **21‐L2**.

Consistent with the attempted catalytic reaction mentioned above, the hydrofluorination product **2a** was not observed when using *cis*‐[PtCl_2_(Xantphos)] (**1‐L7**) in the presence of excess AgBF_4_, 3‐hexyne and PVP HF. Instead, a pincer *β*‐fluorovinyl complex [Pt(EtC═CFEt)(κ^3^‐Xantphos)][BF_4_] (**21**) could be isolated (Figure 4c). Compared to **21‐L2**, the bidentate Xantphos (**L7**) coordinates in a tridentate P,O,P fashion and, therefore, no further alkyne coordination was found. The ^31^P{^1^H} NMR spectrum of **21** displays a doublet (^4^ *J*_P,F_ = 6 Hz) with ^195^Pt satellites with a ^1^ *J*_P,Pt_ coupling constant of 2998 Hz, which is consistent with phosphorus atoms *trans* to each other. The *β*‐fluorovinyl group was identified by a resonance in the ^19^F NMR spectrum as a triplet of triplets with ^195^Pt satellites at δ = −106.15 ppm (^3^ *J*_F,Pt_ = 295 Hz, ^4^ *J*_F,P_ = 6 Hz, ^3^ *J*_F,H_ = 20 Hz). Suitable single crystals for X‐ray diffraction of **22** were obtained and the molecular structure is depicted in Figure 4c.

Monitoring reactions revealed that the bite angle of the phosphine ligands influences both the hydrofluorination catalysis and the cycloallyl complex formation. In both cases, the larger the bite angle of the ligand at the Pt complexes the slower the observed reactivity. When using dppp as ligand the catalytic activity for hydrofluorination is lower, whereas no catalytic activity was observed when using dppb or dppf ligands with larger bite angles (Table 1). On the other hand, the metallacycle formation was observed even for dppb and dppf with the same trend, that is, a slower formation of the complex from dppp to dppf. This suggests that the yields for both pathways do not depend on each other, which implies that the outer‐sphere hydrofluorination pathway is affected in a dissimilar way than the intermolecular cycloaddition pathway by the bite angle of the ligands.

### Mechanism and DFT Calculations

**Figure 5:**
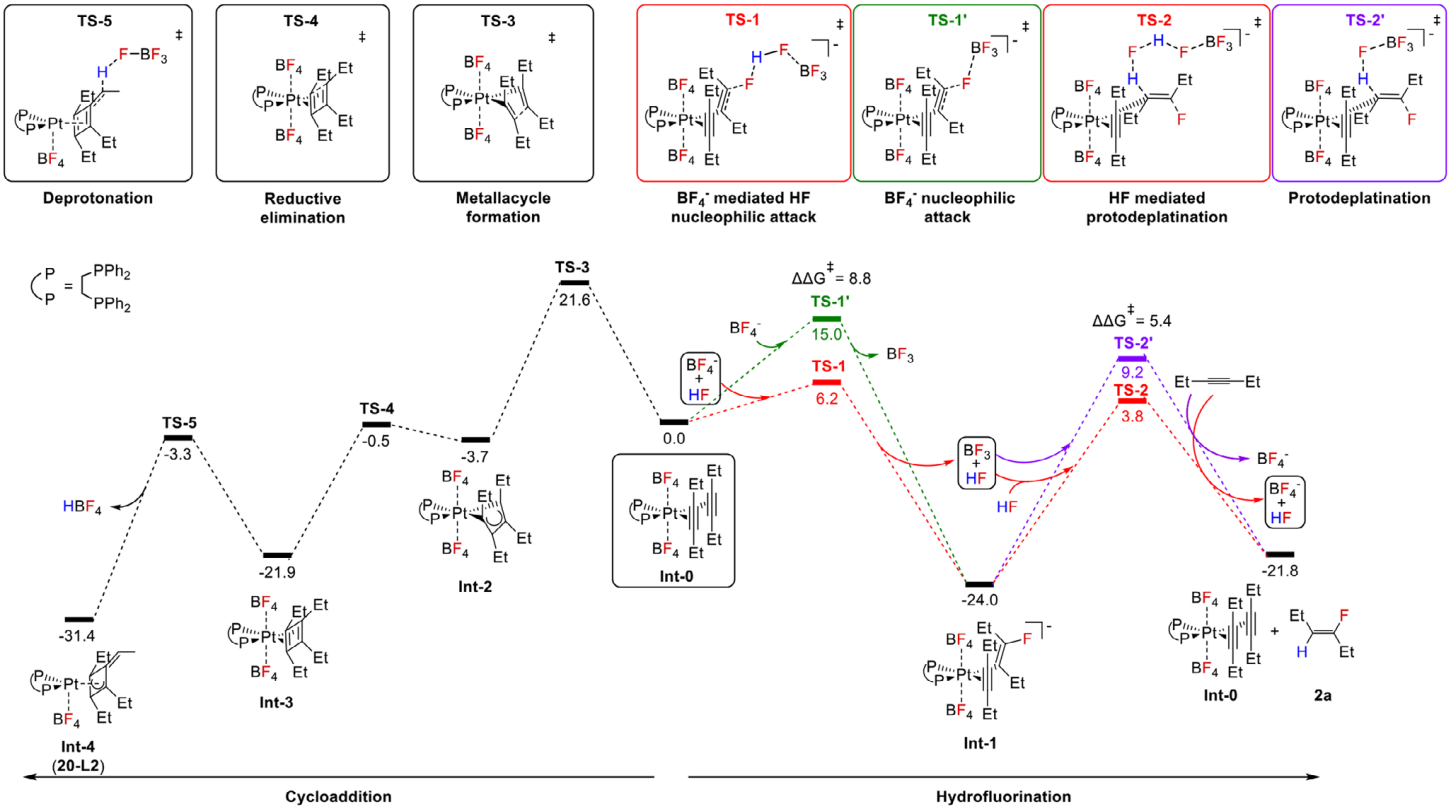
Computed free energy profile for the Pt‐catalysed hydrofluorination of 3‐hexyne and for the formation of an off‐cycle cycloallyl complex. All free energies at 298 K in kcal mol^−1^, obtained at ωB97M‐V/def2‐TZVP//BP86‐D3(BJ)/def2‐TZVP level with internal thermal and entropic contributions at BP86‐D3(BJ)/def2‐TZVP level and solvation contributions at BP86‐D3(BJ)/def2‐TZVP‐COSMO‐RS level (with 1,2‐dichloroethane), relative to [Pt(*C*,*C*‐η^2^‐C_2_H_5_ *C*≡*C*C_2_H_5_)_2_(dppe)][BF_4_]_2_ (**Int‐0**). ΔΔG^‡^ refers to the difference in the activation energy of transition states in the presence of an explicit HF molecule.

**Scheme 2:**
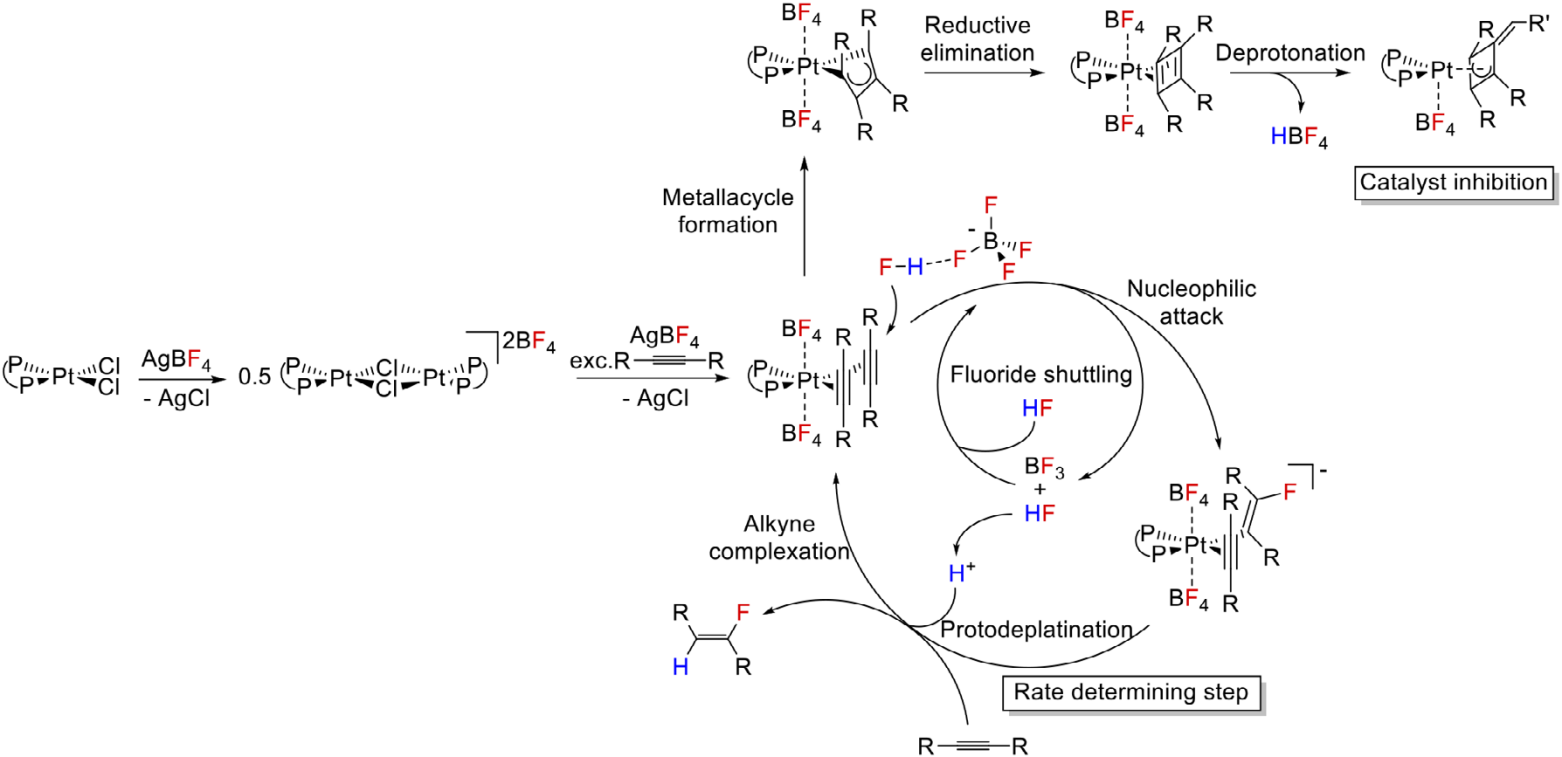
Proposed catalytic cycle based on experimental and computational evaluations for a general chelating precatalyst [PtCl_2_(**L1**‐**L4**)].

DFT calculations were performed to support the experiments and elucidate the reaction mechanism (Figure 5, Scheme 2), at the ωB97M‐V/def2‐TZVP//BP86‐D3(BJ)/def2‐TZVP level for electronic energies, with internal thermal contributions to free energies added at the BP86‐D3(BJ)/def2‐TZVP level and solvation contributions added at the same level using the COSMO‐RS model for 1,2‐dichloroethane. Computational details are provided in the Supporting Information.

An active catalytic species for the hydrofluorination cycle and also the starting point for cycloaddition is [Pt(*C*,*C*‐η^2^‐C_2_H_5_ *C*≡*C*C_2_H_5_)_2_(dppe)][BF_4_]_2_ (**Int‐0**), corresponding to the experimental complex **19‐L2** (cf. Scheme 1). Initially, the binuclear complex [PtCl(dppe)]_2_[BF_4_]_2_ and AgCl are generated (see Figure S81 in SI) from the reaction of [PtCl_2_(dppe)] and AgBF_4_. (see above for the experimental observations). The BF_4_^−^ counter ions in the [PtCl(dppe)]_2_[BF_4_]_2_ dimer coordinate axially to the platinum centre, with Pt–F distances of 3.2  and 3.3 Å. This coordination stabilises the dimer complex significantly (see Table S4 in SI). The computed Gibbs free energy for formation of the dimer [PtCl(dppe)]_2_[BF_4_]_2_ from [PtCl_2_(dppe)] is significantly negative in the gas phase, made somewhat less negative by solvation (see Figure S81 in SI). The bridging chloride ligands in the [PtCl(dppe)]_2_[BF_4_]_2_ dimer are subsequently also abstracted by the silver salt in the presence of the alkyne substrate, forming the Pt bis(alkyne) η^2^‐complex **Int‐0** coordinated by two counter anions (with Pt–F distances of 3.3 Å, cf. Table S4 in SI). By including the two anions, the Pt species becomes overall neutral. This step is somewhat endergonic in the gas phase, but made almost thermoneutral by including solvation contributions. An exploration of alternative conformers for **Int‐0** using tight‐binding methods with subsequent DFT refinement (see Figure S82 in SI) always showed this axial coordination by two BF_4_^−^ anions for any conformer within 6 kcal mol^−1^ of the lowest one. The double BF_4_^−^ coordination stabilises **Int‐0** by −10.5 kcal mol^−1^ in solution. We note in passing that the various roles of counter ions in catalyst stabilisation, hydrogen‐bond anchoring, or proton shuttles have been discussed in detail previously in the context of gold‐promoted hydrofluorination, alkoxylation, or hydration of alkynes.^[^ ^29^, ^56^, ^57^, ^58^, ^59^, ^60^, ^61^, ^62^, ^63^ ^]^

The alkyne ligands in **Int‐0** are susceptible to nucleophilic attack by a fluoride ion, either from a BF_4_^−^ anion or from an HF molecule hydrogen‐bonded to BF_4_^−^. In the computations, this provides the overall system with one negative charge. Given the presence of HF in solution, we have explored both possibilities computationally (Figure 5, TS‐1 and TS‐1′). Both transition states for nucleophilic attack exhibit a slippage of the η^2^‐alkyne coordination toward a vinyl η^1^‐binding mode, with the Pt─C distance to the attacked carbon atom increasing from 2.3  to 2.9 Å and that to the second carbon atom shortening to 2.1 Å for nucleophilic attack by BF_4_^−^ and HF‐BF_4_^−^, respectively. Intrinsic reaction‐coordinate (IRC) analyses confirm the overall breaking of one of the Pt─C bonds, formation of the C─F bond and generation of BF_3_ or HF+BF_3_, for the two cases. The activation free energies for fluoride attack by BF_4_^−^ (TS‐1′) and by HF‐BF_4_^−^ (TS‐1) are 15.0 and 6.2 kcal mol^−1^, respectively (Figure 5). The attack by BF_4_^−^‐bound HF is kinetically more favourable. This can be understood from the larger negative charge on the HF‐based fluoride (see NPA charges in Table S4 in SI), resulting in higher nucleophlicity. The very low barriers for this step seem lower than those computed for a related hydrofluorination in a gold‐catalysed system,^[^ ^20^ ^]^ consistent with the milder reaction conditions in the present case. This may potentially arise from the role of BF_4_^−^ as part of the fluorinating moiety at the cationic platinum centre. Nucleophilic attack leads to facile and exergonic (ΔG = −24 kcal mol^−1^, Figure 5) formation of fluorovinyl complex **Int‐1** (corresponding to **21‐L2** in Figure 4).

A proton source like HF and an alkyne substrate are then required for the protodeplatination step to form **2a** and regenerate **Int‐0**. We have considered the presence of one or two HF molecules in the computations of this step (Figure 5, TS‐2 and TS‐2′), together with BF_3_ as Lewis acid accepting a fluoride. Without the extra HF molecule, a barrier of 33.2 kcal/mol is computed for this step (TS‐2′). The second HF molecule facilitates the protodeplatination (barrier for TS‐2 now 27.5 kcal/mol). NPA charges (see Table S4 in SI) show that this is due to larger charge stabilisation in the transition state, due to an alignment of the H‐F dipoles. It seems possible that the barrier for protodeplatination may be lowered further by involvement of more HF molecules, but such computations are outside the scope of this work. IRC analysis for both transition states reveal the formation of the C─H bond, cleavage of the Pt─C bond, and regeneration of the BF_4_^−^ anion. The protodeplatination is calculated to be slightly endergonic in solution (Figure 5), consistent with the experimental observation of **21‐L2**. Notably, while this step is the rate‐determining one for the overall hydrofluorination, back‐reaction to **Int‐0** is prevented by the notable exergonicity of the fluorination step and the consequently high barrier for the back‐reaction. This is important in the context of the off‐cycle formation of a cycloallyl complex (see below).

We note in passing the recent use of BF_4_^−^ in a metal‐free alkyne hydrofluorination of diphenylacetylenes with 2,6‐dichloropyridinium tetrafluoroborate.^[^ ^29^ ^]^ In that case a reverse mechanism with initial protonation and subsequent nucleophilic attack of BF_4_^−^ on the activated cationic species was proposed. The computed activation barriers seem higher than those found here, consistent with the harsher conditions used.

We have also evaluated computationally the formation of the off‐cycle cycloallyl complex [Pt(η^3^‐(C_2_H_5_ *C*)_3_ *C*═C_2_H_4_)(dppe)][BF_4_] (**Int‐4**, corresponding to **20‐L2** in Scheme 1), see left side of Figure 5. We found that **Int‐4** is formed from **Int‐0** in a three‐step process that starts with an alkyne C‐C coupling to give metallacycle **Int‐2**. The barrier for this initial step is larger than the first barrier of hydrofluorination, in particular when we compare to the HF‐assisted fluorination step (Figure 5, TS‐1/TS‐1′ vs. TS‐3). This makes formation of **Int‐2** kinetically disfavoured compared to formation of fluorovinyl complex **Int‐1**, consistent with the experimental observation that in the presence of BF_4_^−^ and HF hydrofluorination occurs preferentially. Formation of **Int‐2** from **Int‐0** is computed to be slightly exergonic in solution.

The formation of the cyclobutadiene complex **Int‐3** from **Int‐2** occurs via reductive elimination. This step has a very small barrier and is significantly exergonic (Figure 5, TS‐4). The cycloallyl complex **Int‐4** (**20‐L2**) is finally formed from **Int‐3** by an exergonic deprotonation, where we have computationally utilised BF_4_^−^ as a likely base (Figure 5, TS‐5). **Int‐4** is calculated to be 34 kcal/mol lower than **Int‐0** in solution and would therefore constitute an undesirable off‐cycle sink for the catalytic hydrofluorination. It is indeed the dominant observed product under conditions where the hydrofluorination is less favourable (see above). Computed structural and electronic information on the species involved in both the hydrofluorination and the formation of **Int‐4** is provided in Table S4 in Supporting Information.

The cyclisation mechanism from **Int‐0** to **Int‐3** can be related to a recent computational study on cyclisation of cobalt and rhodium bis(alkyne) complexes, which also exhibit d^8^ electronic situations.^[^ ^64^ ^]^ That mechanism also involves metallacycle intermediates.

The suggested overall generic catalytic cycle for hydrofluorination starting from **Int‐0** and the off‐cycle formation of **Int‐4** based on both our experimental and computational investigations is summarised in Scheme 2.

## Conclusion

This work introduces a remarkable method for the selective platinum‐catalysed hydrofluorination of alkynes giving previously undescribed fluorinated olefins. The simple reaction conditions allow access to (*Z*)‐fluoroalkenes that even bear bulky substituents. The transformations are carried out at room temperature and are tolerant to air and moisture, that is, in contrast to some other approaches no formation of hydration products was observed. Experimental and computational investigations reveal that BF_3_, HF or PF_5_ serve as fluoride shuttles enabling fluorination at a metal‐bound alkyne of a dicationic platinum bis(alkyne) complex. As a result, a platinum fluorovinyl intermediate is formed. The computational studies show that this undergoes the protodeplatination as the rate determining step for a HF/BF_3_ mediated hydrofluorination. During the catalysis the BF_4_^−^ counter anions i) stabilise the complexes by coordinating to the Pt centre, ii) act either as direct fluorinating agent or activate bound HF for facilitation of an outer‐sphere fluorination^[^ ^20^, ^65^ ^]^ at an alkyne ligand, and iii) anchor one or more HF molecules during the rate‐determining protodeplatination step. The new methodology opens doors to the synthesis of a wide range of novel fluoroalkenes and offers potential for applications in fields like pharmaceuticals and materials science. Current studies are exploring the utility of Pt catalysts in a broader context of fluorination reactions.

## Supporting Information

The authors have cited additional references within the Supporting Information.^[^ ^17^, ^26^, ^28^, ^29^, ^41^, ^51^, ^66^, ^67^, ^68^, ^69^, ^70^, ^71^, ^72^, ^73^, ^74^, ^75^, ^76^, ^77^, ^78^, ^79^, ^80^, ^81^, ^82^, ^83^, ^84^, ^85^, ^86^, ^87^, ^88^, ^89^, ^90^, ^91^, ^92^, ^93^, ^94^, ^95^, ^96^, ^97^, ^98^, ^99^, ^100^, ^101^, ^102^, ^103^, ^104^, ^105^, ^106^ ^]^

## Author Contributions

O.H. and H.F. carried out the experiments, collected analytic data. F.J. and S.S.B. performed DFT calculations. O.H. and F.J. wrote the original draft. O.H. did the single crystal X‐ray diffraction analyses. T.B. and O.H. conceived the project and T.B. provided guidance throughout the study. M.K. and T.B. supervised the work. All authors contributed to the manuscript and edited it.

## Conflict of Interests

The authors declare no conflict of interest.

## Supporting information



Supporting Information

## Data Availability

The data that support the findings of this study are available in the Supporting Information of this article.
