# Supplementary material for: Platinum‐Catalysed Hydrofluorination of Alkynes at Room Temperature Promoted by a Fluoride Shuttle
Source: Angew Chem Int Ed Engl. 2025 Jul 23;64(37):e202512181. doi: 10.1002/anie.202512181 (PMC12416478; doi:10.1002/anie.202512181)
Supplement: Supplementary file 1 — Supporting Information [file ANIE-64-e202512181-s001.pdf]

Supporting Information

Platinum-Catalysed Hydrofluorination of Alkynes at Room Temperature Promoted by a Fluoride Shuttle

Ouchan He, Froze Jameel, Hannah Flammang, Smrithi Suresh Babu, Martin Kaupp* and Thomas Braun*

Supporting Information

Table of Content

1 Experimental section 2

2 Experimental procedure..... 3

2.1 Synthesis and characterisation of ligands 3

2.2 Synthesis and characterisation of platinum precatalysts 5

2.3 Synthesis and characterisation of [Pt(C_2H_5*C*=CFC_2H_5)(κ^3-Xantphos)][BF_4] (**22**) 9

2.4 Synthesis and characterisation of [Pt(η^3-(C_2H_5*C*)$_3$*C*=C_2H_4)(dppe)][BF_4] (**20-L2**)..... 10

2.5 Synthetic procedures for alkynes 11

2.6 Catalytic hydrofluorination reaction 11

3 Precatalyst screening and hydrofluorination of 3-hexyne 26

3.1 Precatalyst screening..... 26

3.2 Optimisation of reaction conditions 27

3.3 Model reactions..... 29

3.4 Experiments for the Observation of *β*-Fluorovinyl Platinum Complexes (**21**) as Intermediates..... 31

3.5 Monitoring of the catalytic reactions by NMR spectroscopy 34

4 Crystal data and structure refinement..... 36

7 NMR Spectra..... 42

5 Computational Details..... 71

8 Cartesian coordinates..... 76

6 References 95

1 Experimental section

All manipulations, if not otherwise mentioned, were carried out under argon using Schlenk techniques or in an argon-filled glovebox (MBRAUN). Dichloromethane (DCM, CH_2Cl_2) and CD_2Cl_2 were dried over CaH_2 and distilled prior to usage. Tetrahydrofuran and *n*-hexane were dried and purified by distillation from SOLVONA®. Diethylether (Et_2O) and *n*-pentane were dried over alumina from an SPS (solvent purification system). Except for 1,2-dichloroethane and $CDCl_3$, all solvents were degassed and stored under argon over activated molecular sieves.

[$PtCl_2$(COD)],[1] [$PtMe_2$(COD)],[2] [PtMeCl(COD)],[3] and $AgBAr^F_4$[4] were prepared according to the literature.

Chlorobis(diphenyl)phosphine was obtained from abcr GmbH and distilled for purification. $Et_3N\cdot3HF$, poly[4-vinylpyridinium poly(hydrogen fluoride)] (PVPHF containing 38-42w% HF), aqueous HF (48w% in H_2O) and bis(dichlorophosphino)ethane were obtained from Sigma-Aldrich and were used without further purification. **Caution**: Experiments with HF should be handled with care. HF is corrosive and toxic and will damage any equipment composed of borosilicate glass.

Catalytic reactions were carried out in PTFE test tubes with a round bottom and screw cap (8 mL) (SPI Supplies Brand) unless otherwise described.

Reactions were monitored by TLC on pre-coated plates (Roth silica gel 60 Å 5-17 µm) and products were visualised under 254 nm UV light followed by staining with $KMnO_4$ when appropriate. Purifications by flash column chromatography were carried out on silica gel (Roth silica gel 60 Å 0.07-0.2 mm) or by CombiFlash NextGen 300+ from Advion Interchim Scientific chromatography system using RediSep silica gel cartridge.

The NMR spectra were recorded on a Bruker Avance 500, Bruker Avance 300, or a Bruker NEO 300 spectrometer. The ^1H NMR chemical shifts were referenced to residual CD_2Cl_2 at δ = 5.32 ppm or $CDCl_3$ at δ = 7.26 ppm. The $^{13}C\{^1H\}$ NMR spectra were referenced to CD_2Cl_2 at δ = 53.84 ppm or $CDCl_3$ at δ = 77.16 ppm. The ^{19}F NMR chemical shifts were referenced to external $CFCl_3$ at δ = 0.00 ppm. Fluoroalkene yields were determined from ^{19}F NMR data on using benzotrifluoride as internal standard. Data analysis was performed with the software TopSpin 4.0.7. Note, that an appropriate relaxation delay (D1, at least 5 x T1) and acquisition time (aq = 2.0 s) have to be applied for quantification by ^{19}F NMR spectroscopy using an internal standard.[5-7] The $^{31}P\{^1H\}$ NMR chemical shifts were referenced to external 85% H_3PO_4 at δ = 0.00 ppm. The ^{11}B NMR chemical shifts were referenced to external $BF_3\cdot OEt_2$ at δ = 0.00 ppm. The ^{195}Pt NMR chemical shifts were referenced to external Na_2PtCl_4 at δ = 0.00 ppm.

Gas chromatography - mass spectra (GC/MS) were recorded on an Agilent Technologies 6890N instrument with an Agilent 5973 Network mass-selective detector (EI) and a HP5-MS 30 m x 0.25 mm capillary apolar column (stationary phase: 5% diphenyldimethylpolysiloxane film, 0.25 µm). Helium (0.74 bar, 1.2 mL/min, 40 cm/s) was used as the carrier gas. The electron impact ionisation was carried out with an ionisation voltage of 70 eV. GC/MS method: Initial temperature: 50 °C; initial time: 1 min; ramp: 20 °C/min until 150 °C, holding for 1 min, then 20 °C/min; final temperature: 250 °C; final time: 5 min.

Electrospray Ionisation Mass Spectrometry (ESI-MS) spectra were recorded by using an ADVION EXPRESSION CMS spectrometer (in typical ionisation mode); acetonitrile was used as an eluent. Data analysis was carried out with ADVION DATA EXPRESS Version 6.0.11.3.

Structure determination of **L3**, **1-L3**, [$Pt(Me)_2$(PCNP)], **18-L7**·$6CH_2Cl_2$, **20-L2** and **22**: The crystallographic data were collected at a Bruker D8 Venture diffractometer at 100(2) K using Mo-Kα (λ = 0.71073 Å) radiation. Multi-scan absorption corrections implemented in SADABS[8] were applied to the data. The structures were solved by intrinsic phasing method (SHELXT 2014/5)[9] and refined by full-matrix least-squares methods based on F^2 (SHELXL 2016/4 or SHELXL-2018/3)[10] with anisotropic temperature factors for all non-hydrogen atoms. All hydrogen atoms were added geometrically and refined by using a riding model. Deposition numbers CCDC-2429317 (for PCNP (**L3**)), CCDC-2429320 (for [$PtCl_2$(PCNP)] (**1-L3**)), CCDC-2429315 (for *cis*-[PtCl(Xantphos)]$_2$[BF_4]$_2$·$6CH_2Cl_2$ (**18-L7**·$6CH_2Cl_2$), CCDC-2429316 (for [Pt(η^3-(C_2H_5C)$_3C$=C_2H_4)(dppe)][BF_4] (**20-L2**)), CCDC-2429318 (for [Pt(EtC=CFEt)(κ^3-Xantphos)][BF_4] (**22**)) and CCDC-2429319 (for [$Pt(Me)_2$(PCNP)]) contain the supplementary crystallographic data for this paper. These data are provided free of charge by the joint Cambridge Crystallographic Data Centre and Fachinformationszentrum Karlsruhe Access Structures service.

2 Experimental procedure

2.1 Synthesis and characterisation of ligands

The following sections describe ligand syntheses. The ligands were used for the syntheses of precatalyst, which in turn were applied for their screening of the catalytic system (see chapter 2.2 and Table S2).

Diphenyl(3-methyl-2-indolyl)phosphine ($Ph_2P(Ind)$)

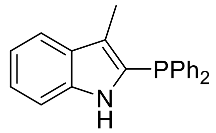

According to a literature procedure,[11] to a solution of 3-methylindole (2.62 g, 20.0 mmol, 1.00 eq.) in THF (60 mL) 2.5 M *n*BuLi in hexane (8.4 mL, 21.0 mmol, 1.05 eq.) was added dropwise over 10 min at -70 °C. The white suspension was stirred for 10 min at -70 °C. While carbon dioxide was bubbled through the mixture for 30 min, the suspension was allowed to warm up to room temperature. The solvent was removed in *vacuo* and the resulting white residue was redissolved in THF (85 mL). To this solution 1.7 M *t*BuLi in pentane (12.4 mL, 21.0 mmol, 1.05 eq.) was added dropwise at -70 °C. After stirring the resulting yellow suspension for 1 h at -70 °C, chlorodiphenylphosphine (3.59 mL, 20.0 mmol, 1.00 eq.) was added dropwise. The reaction mixture was stirred overnight and allowed to warm up to room temperature. The suspension was washed with saturated aq. NH_4Cl (70 mL) until the mixture turned clear. The organic layer was separated and dried over $MgSO_4$, filtered and concentrated in *vacuo*. The beige residue was washed with hexane (2x15 mL) and dried in *vacuo*. The desired product was isolated as a white powder (5.51 g, 87%).

^{1}H NMR (300.1 MHz, CD_2Cl_2): δ [ppm] = 7.59 (dm, $^3J_{H,H}$ = 2 Hz, 2H, CH_{Ar}), 7.40-7.29 (m, 10H, CH_{Ar}), 7.23 (ddd, $^3J_{H,H}$ = 8 Hz, $^4J_{H,H}$ = 1 Hz, $^4J_{H,H}$ = 1 Hz, 1H, CH_{Ar}), 7.19-7.13 (m, 1H, CH_{Ar}), 7.09 (ddd, $^3J_{H,H}$ = 8 Hz, $^3J_{H,H}$ = 7 Hz, $^4J_{H,H}$ = 1 Hz, 1H, CH_{Ar}), 2.46 (s, 3H, CH_3).

^{31}P{^{1}H} NMR (121.5 MHz, CD_2Cl_2): δ [ppm] = -32.9 (s).

1,2-Bis(diphenylphosphino)-3-methyl-1*H*-indole (PCNP, L3)

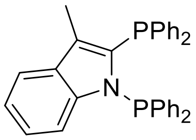

To a solution of 1,2-bis(diphenylphosphino)-3-methyl-1*H*-indole ($Ph_2P(Ind)$) (3.92 g, 12.43 mmol, 1.00 eq.) in THF (40 mL) 2.5 M *n*BuLi in hexane (5.22 mL, 13.05 mmol, 1.1 eq.) was added dropwise at -70 °C. After stirring the resulting yellow-orange solution for 1 h at -70 °C, chlorodiphenylphosphine (2.35 mL, 12.43 mmol, 1.00 eq.) was added dropwise. The reaction mixture was stirred overnight and allowed to warm up to room temperature. Degassed H_2O (40 mL) was added, the organic layer was separated and dried over $MgSO_4$, filtered and concentrated in *vacuo*. The beige residue was washed with hexane (2x10 mL) and dried in *vacuo*. The desired product was isolated as a white powder (4.33 g, 70%). Colourless single crystals of **L3** were obtained by slow evaporation of the solvent from a dichloromethane solution at room temperature.

^{1}H NMR (500.1 MHz, CD_2Cl_2): δ [ppm] = 7.54 (dt, $^3J_{H,H}$ = 8 Hz, $^4J_{H,H}$ = 1 Hz, 1H, CH_{Ar}), 7.47-7.43 (m, 4H, CH_{Ar}), 7.33-7.22 (m, 16H, CH_{Ar}), 7.05 (ddd, $^3J_{H,H}$ = 8 Hz, $^3J_{H,H}$ = 7 Hz, $^4J_{H,H}$ = 1 Hz, 1H, CH_{Ar}), 6.86 (ddd, $^3J_{H,H}$ = 8 Hz, $^3J_{H,H}$ = 7 Hz, $^4J_{H,H}$ = 1 Hz, 1H, CH_{Ar}), 6.80 (dm, $^3J_{H,H}$ = 8 Hz, 1H, CH_{Ar}), 1.88 (s, 3H, CH_3).

^{13}C{^{1}H} NMR (125.8 MHz, CD_2Cl_2): δ [ppm] = 141.4 (d, J = 8 Hz, C_{Ar}), 136.4 (d, J = 6 Hz, C_{Ar}), 136.2 (dd, J = 10 Hz, J = 6 Hz, C_{Ar}), 135.5 (dd, J = 17 Hz, J = 2 Hz, C_{Ar}), 133.7 (d, J = 4 Hz, C_{Ar}), 133.4 (CH$_{Ar}$), 133.2 (CH$_{Ar}$), 131.5 (CH$_{Ar}$), 131.4 (CH$_{Ar}$), 129.3 (CH$_{Ar}$), 128.9 (CH$_{Ar}$), 128.8 (CH$_{Ar}$), 128.8 (CH$_{Ar}$), 128.7 (CH$_{Ar}$), 128.6 (CH$_{Ar}$), 125.4 (dd, J = 5 Hz, J = 4 Hz, C_{Ar}), 123.2 (CH$_{Ar}$), 120.8 (CH$_{Ar}$), 119.6 (CH$_{Ar}$), 115.0 (CH$_{Ar}$), 10.6 (d, $^3J_{C,P}$ = 4 Hz, CH$_3$).

^{31}P{^{1}H} NMR (121.5 MHz, CD_2Cl_2): δ [ppm] = 35.3 (d, $^3J_{P,P}$ = 117 Hz, NP), -26.2 (d, $^3J_{P,P}$ = 117 Hz, CP).

1,2-Bis(diphenyl(3-methyl-2-indolyl)phosphino)ethane (d(Ind)pe)

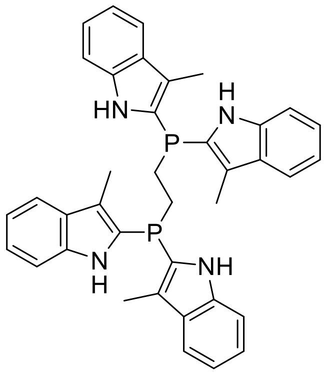

To a solution of 3-methylindole (1.31 g, 10.0 mmol, 4.00 eq.) in THF (30 mL) 2.5 M *n*BuLi in hexane (4.1 mL, 10.25 mmol, 4.1 eq.) was added dropwise at -70 °C. The white suspension was stirred for 10 min at -70 °C. Carbon dioxide was bubbled through the mixture for 30 min and the suspension was allowed to warm up to room temperature. The solvent was removed in *vacuo* and the white residue was redissolved in THF (30 mL). To this solution 1.7 M *t*BuLi in pentane (6.0 mL, 10.25 mmol, 4.1 eq.) was added dropwise at -70 °C. After stirring the yellow solution for 1 h at -70 °C, 1,2-bis(dichlorophosphino)ethane (446 µL, 2.5 mmol, 1.00 eq.) was added dropwise. The reaction mixture was stirred overnight and allowed to warm up to room temperature. The solution was washed with saturated aq. NH_4Cl (40 mL). The organic layer was separated and dried over $MgSO_4$, filtered and concentrated in *vacuo*. The yellow solid was washed with hexane (3x10 mL) and dried in *vacuo*. D(Ind)pe was isolated as an off-white powder (1.48 g, 97%).

^{1}H NMR (500.1 MHz, CD_2Cl_2): δ [ppm] = 7.86 (bs, 4H, N*H*), 7.54 (dm, $^3J_{H,H}$ = 8 Hz, 4H, CH_{Ar}), 7.16 (ddd, $^3J_{H,H}$ = 8 Hz, $^3J_{H,H}$ = 7 Hz, $^4J_{H,H}$ = 1 Hz, 4H, CH_{Ar}), 7.09 (ddd, $^3J_{H,H}$ = 8 Hz, $^3J_{H,H}$ = 8 Hz, $^4J_{H,H}$ = 1 Hz, 8H, CH_{Ar}), 2.40 (s, 12H, CH_3), 2.29 (t, $^2J_{H,P}$ = 5 Hz, 4H, CH_2).

^{13}C{^{1}H} NMR (125.8 MHz, CD_2Cl_2): δ [ppm] = 138.6 (C_{Ar}), 129.6 (t, J = 3 Hz, C_{Ar}), 127.3 (dd, J = 12 Hz, J = 8 Hz, C_{Ar}), 123.5 (dd, J = J = CH_{Ar}), 121.7 (C_{Ar}), 119.9 (CH_{Ar}), 119.3 (CH_{Ar}), 111.5 (CH_{Ar}), 22.6 (m, CH_2), 10.0 (t, $^3J_{C,P}$ = 6 Hz, CH_3).

^{31}P{^{1}H} NMR (202.4 MHz, CD_2Cl_2): δ [ppm] = -68.3 (s).

2.2 Synthesis and characterisation of platinum precatalysts

The following sections describe the synthesis of platinum complexes that were used for precatalyst screening of the catalytic system (see Table S2).

[$PtCl_2L_2$] (1)

General procedure A for the synthesis of platinum dichlorido complexes [$PtCl_2L$] (L = $(PPh_3)_2$, {$Ph_2P(Ind)$}$_2$, dppm, dppe, dcpe, PCNP, d(Ind)pe, dppp, dppb, dppf, Xantphos).

To a solution of [$PtCl_2(COD)$] (0.75 mmol, 1 eq.) in CH_2Cl_2 (10 mL) a solution of the ligand (0.75 mmol, 1 eq.) in CH_2Cl_2 (15 mL) was added. The reaction mixture was stirred at room temperature for 60 min. All volatiles were removed in *vacuo* and the crude product was recrystallised from a CH_2Cl_2/*n*-pentane mixture (1/10) at room temperature. The supernatant was filtered off and the residue was washed with *n*-pentane (3 x 10 mL) and dried in *vacuo*.

[$PtCl_2(Ph_3P)_2$],[12] [$PtCl_2${$Ph_2P(Ind)$}$_2$],[13] [$PtCl_2$(dppm)] (**1-L1**),[12] [$PtCl_2$(dppe)] (**1-L2**),[12] [$PtCl_2$(dcpe)],[14] [$PtCl_2$(dppp)] (**1-L4**),[15] [$PtCl_2$(dppb)] (**1-L5**),[15] [$PtCl_2$(dppf)] (**1-L6**),[16] [$PtCl_2$(Xantphos)] (**1-L7**)[17] were prepared according to the general procedure A. Spectroscopic data are in accordance with the literature.

[$PtCl_2$(PCNP)] (1-L3)

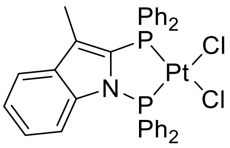

Following the general procedure A [$PtCl_2(COD)$] (0.75 mmol) in 10 mL CH_2Cl_2 reacted with 1,2-bis(diphenylphosphino)-3-methyl-1*H*-indole (PCNP, **L3**) (374 mg, 0.75 mmol) in 15 mL CH_2Cl_2. [$PtCl_2$(PCNP)] (**1-L3**) was isolated as a pale-yellow powder (562 mg, 98%). Colourless single crystals of **1-L3** were obtained by slow evaporation of the solvent from a dichloroethane solution at room temperature.

^{1}H NMR (300.1 MHz, CD_2Cl_2): δ [ppm] = 7.92-7.79 (m, 7H, CH_{Ar}), 7.64-7.45 (m, 14H, CH_{Ar}), 7.25-7.18 (m, 1H, CH_{Ar}), 7.13-7.05 (m, 1H, CH_{Ar}), 6.81 (dm, $^3J_{H,H}$ = 9 Hz, 1H, CH_{Ar}), 1.99 (s, 3H, CH_3).

^{31}P{^{1}H} NMR (121.5 MHz, CD_2Cl_2): δ [ppm] = 62.0 (d + sat., $^1J_{P,Pt}$ = 3970 Hz, $^2J_{P,P}$ = 12 Hz), 7.9 (d + sat., $^1J_{P,Pt}$ = 3427 Hz, $^2J_{P,P}$ = 12 Hz).

[$PtCl_2$(d(Ind)pe)]

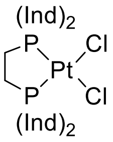

Following the general procedure A [$PtCl_2(COD)$] (0.75 mmol) in 10 mL CH_2Cl_2 reacted with 1,2-bis(diphenyl(3-methyl-2-indolyl)phosphino)ethane (d(Ind)pe) (458 mg, 0.75 mmol) in 15 mL CH_2Cl_2. [$PtCl_2$(d(Ind)pe)] was isolated as a pale-yellow powder (625 mg, 95%).

^{1}H NMR (300.1 MHz, CD_2Cl_2): δ [ppm] = 9.42 (bs, 4H, NH), 7.63 (dm, $^3J_{H,H}$ = 8 Hz, 4H, CH_{Ar}), 7.44 (dt, $^3J_{H,H}$ = 8 Hz, $^4J_{H,H}$ = 1 Hz, 4H, CH_{Ar}), 7.31 (tm, $^3J_{H,H}$ = 8 Hz, 4H, CH_{Ar}), 7.17 (ddd, $^3J_{H,H}$ = 8 Hz, $^3J_{H,H}$ = 7 Hz, $^4J_{H,H}$ = 1 Hz, 4H, CH_{Ar}), 2.84 (m+sat., $^3J_{H,Pt}$ = 41 Hz, 4H, CH_2), 2.46 (s, 6H, CH_3), 2.35 (m, 6H, CH_3).

^{31}P{^{1}H} NMR (121.5 MHz, CD_2Cl_2): δ [ppm] = 13.5 (s + sat., $^1J_{P,Pt}$ = 3566 Hz).

[PtMeCl(L)]

General procedure B for the synthesis of platinum methyl chlorido complexes [PtMeCl(L)] (L = dppe, PCNP).

To a solution of [PtMeCl(COD)] (0.75 mmol, 1 eq.) in CH_2Cl_2 (10 mL) a solution of the ligand (0.75 mmol, 1 eq.) in CH_2Cl_2 (15 mL) was added. The reaction mixture was stirred at room temperature overnight. All volatiles were removed in *vacuo* and the crude product was recrystallised from a CH_2Cl_2/*n*-pentane mixture (1/10) at room temperature. The supernatant was filtered off and the residue was washed with *n*-pentane (3 x 10 mL) and dried in *vacuo*.

[PtMeCl(dppe)] was prepared according to the general procedure B. Spectroscopic data are in accordance with the literature.[18]

[PtMeCl(PCNP)]

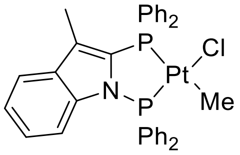

Following the general procedure B [PtMeCl(COD)] (531 mg, 1.5 mmol) in 15 mL CH_2Cl_2 reacted with 1,2-bis(diphenylphosphino)-3-methyl-1*H*-indole (PCNP, **L3**) (749 mg, 1.5 mmol) in 20 mL CH_2Cl_2. [PtMeCl(PCNP)] was isolated as a white powder containing a mixture of the isomers in a ratio of 1:0.6 (1.10 g, 98%).

Major Isomer:

^{1}H NMR (300.1 MHz, CD_2Cl_2): δ [ppm] = 7.81-7.68 (m, CH_{Ar}), 7.58-7.41 (m, CH_{Ar}), 7.17 (tm, $^3J_{H,H}$ = 8 Hz, CH_{Ar}), 6.83 (d, $^3J_{H,H}$ = 8 Hz, 1H, CH_{Ar}), 2.02 (s, 3H, CH_3), 0.46 (dd+sat., $^2J_{H,Pt}$ = 56 Hz, $^3J_{H,P}$ = 4 Hz, $^3J_{H,P}$ = 7 Hz, 3H, PtCH_3).

^{31}P{^{1}H} NMR (121.5 MHz, CD_2Cl_2): δ [ppm] = 67.1 (d + sat., $^1J_{P,Pt}$ = 4512 Hz, $^2J_{P,P}$ = 16 Hz, P *trans* to Cl, N*P*), 16.1 (d + sat., $^1J_{P,Pt}$ = 1643 Hz, $^2J_{P,P}$ = 16 Hz, P *trans* to Me, CP).

Minor isomer:

^{1}H NMR (300.1 MHz, CD_2Cl_2): δ [ppm] = 7.81-7.68 (m, CH_{Ar}), 7.58-7.41 (m, CH_{Ar}), 7.04 (tm, $^3J_{H,H}$ = 8 Hz, CH_{Ar}), 6.92 (d, $^3J_{H,H}$ = 8 Hz, 1H, CH_{Ar}), 1.98 (s, 3H, CH_3), 0.59 (dd+sat., $^2J_{H,Pt}$ = 56 Hz, $^3J_{H,P}$ = 3 Hz, $^3J_{H,P}$ = 8 Hz, 3H, PtCH_3).

^{31}P{^{1}H} NMR (121.5 MHz, CD_2Cl_2): δ [ppm] = 79.6 (d + sat., $^1J_{P,Pt}$ = 1894 Hz, $^2J_{P,P}$ = 16 Hz, P *trans* to Me, N*P*), 11.9 (d + sat., $^1J_{P,Pt}$ = 3999 Hz, $^2J_{P,P}$ = 12 Hz, P *trans* to Cl, CP).

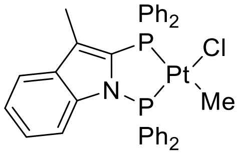

:

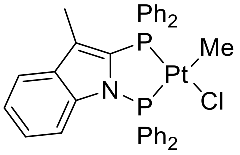

1 : 0.6

[$PtMe_2$(PCNP)]

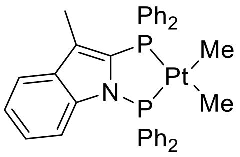

To a solution of [$PtMe_2$(COD)] (500 mg, 1.5 mmol, 1 eq.) in CH_2Cl_2 (15 mL) a solution of the ligand (749 mg, 1.5 mmol, 1 eq.) in CH_2Cl_2 (20 mL) was added. The reaction mixture was stirred at room temperature overnight. All volatiles were removed in *vacuo*. The crude product was washed with *n*-pentane (3 x 10 mL) and dried in *vacuo*. [$PtMe_2$(PCNP)] was obtained as a white powder (1.05 g, 97%). Colourless single crystals of [$PtMe_2$(PCNP)] were obtained by slow evaporation of the solvent from a dichloromethane solution at room temperature.

^{1}H NMR (300.1 MHz, CD_2Cl_2): δ [ppm] = 7.73-7.59 (m, 8H, CH_{Ar}), 7.52 (d, $^{3}J_{H,H}$ = 8 Hz, 1H, CH_{Ar}), 7.49-7.36 (m, 12H, CH_{Ar}), 7.12 (t, $^{3}J_{H,H}$ = 8 Hz, 1H, CH_{Ar}), 7.00 (t, $^{3}J_{H,H}$ = 8 Hz, 1H, CH_{Ar}), 6.91 (d, $^{3}J_{H,H}$ = 8 Hz, 1H, CH_{Ar}), 1.99 (s, 3H, CH_3), 0.57 (t+sat., $^{2}J_{H,Pt}$ = 71 Hz, $^{3}J_{H,P}$ = 8 Hz, 3H, PtCH_3), 0.43 (t+sat., $^{2}J_{H,Pt}$ = 71 Hz, $^{3}J_{H,P}$ = 8 Hz, 3H, PtCH_3).

^{31}P{^{1}H} NMR (121.5 MHz, CD_2Cl_2): δ [ppm] = 82.6 (d + sat., $^{1}J_{P,Pt}$ = 1916 Hz, $^{2}J_{P,P}$ = 15 Hz, N*P*), 17.0 (d + sat., $^{1}J_{P,Pt}$ = 1709 Hz, $^{2}J_{P,P}$ = 15 Hz, C*P*).

[PtCl(L)]$_2$[BF$_4$]$_2$ (18)

General procedure C for the synthesis of [PtCl(L)]$_2$[BF$_4$]$_2$ (L = dppe, PCNP, Xantphos).

To a suspension of [PtCl$_2$(L)] (0.75 mmol, 1 eq.) in CH_2Cl_2 (20 mL) a solution of $AgBF_4$ (0.75 mmol, 1 eq.) in CH_2Cl_2 (10 mL) was added. The reaction mixture was stirred at room temperature for 20 min and filtered. The solvent was removed in *vacuo* and the crude product was recrystallised from a CH_2Cl_2/Et_2O mixture (1/10) at room temperature. The supernatant was filtered off and the residue dried in *vacuo*.

[PtCl(dppe)]$_2$[BF$_4$]$_2$[19] (**18-L2**) was prepared according to the general procedure C and the spectroscopic data are in accordance with the literature.

[PtCl(PCNP)]$_2$[BF$_4$]$_2$ (18-L3)

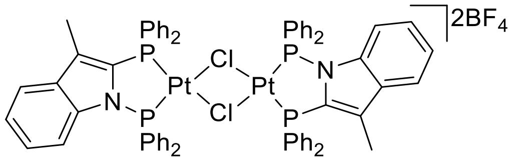

Following the general procedure C [PtCl$_2$(PCNP)] (36 mg, 0.05 mmol) in 1.5 mL CH_2Cl_2 reacted with $AgBF_4$ (10 mg, 0.05 mmol) in 1 mL CH_2Cl_2. [PtCl(PCNP)]$_2$[BF$_4$]$_2$ (**18-L3**) was isolated as an orange powder containing traces of the isomer C_{2v} (78 mg, 96%).

^{1}H NMR (300.1 MHz, CD_2Cl_2): δ [ppm] = 7.82-7.51 (m, 42H, CH_{Ar}), 7.32 (t, $^3J_{H,H}$ = 8 Hz, 2H, CH_{Ar}), 7.21 (t, $^3J_{H,H}$ = 8 Hz, 2H, CH_{Ar}), 6.74 (d, $^3J_{H,H}$ = 8 Hz, 2H, CH_{Ar}), 2.01 (s, 6H, CH_3).

^{31}P{^{1}H} NMR (121.5 MHz, CD_2Cl_2): δ [ppm] = 58.8 (d + sat., $^1J_{P,Pt}$ = 4255 Hz, $^2J_{P,P}$ = 12 Hz, N*P*), 10.2 (d + sat., $^1J_{P,Pt}$ = 3577 Hz, $^2J_{P,P}$ = 12 Hz, C*P*).

^{19}F NMR (282.4 MHz, CD_2Cl_2): δ [ppm] = -153.1 (br s, BF_4).

[PtCl(Xantphos)]$_2$[BF$_4$]$_2$ (18-L7)

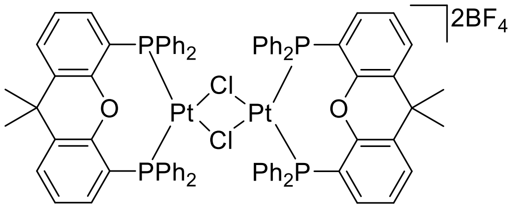

Following the general procedure C [PtCl$_2$(Xantphos)] (36 mg, 0.05 mmol) in 1.5 mL CH_2Cl_2 reacted with $AgBF_4$ (10 mg, 0.05 mmol) in 1 mL CH_2Cl_2. [PtCl(Xantphos)]$_2$[BF$_4$]$_2$ (**18-L7**) was isolated as a yellow powder (87 mg, 97%). Yellow single crystals of **18-L7**·6CH_2Cl_2 were obtained by slow evaporation of the solvent from a dichloromethane solution at room temperature.

^{1}H NMR (500.1 MHz, CD_2Cl_2): δ [ppm] = 7.89 (d, $^3J_{H,H}$ = 8 Hz, 4H, CH_{Ar}), 7.43-7.36 (m, 28H. CH_{Ar}), 7.25-7.19 (m, 20H, CH_{Ar}) 1.91 (s, 12H, CH_3).

^{31}P{^{1}H} NMR (202.5 MHz, CD_2Cl_2): δ [ppm] = -5.6 (s + sat., $^1J_{P,Pt}$ = 4246 Hz).

^{19}F NMR (282.4 MHz, CD_2Cl_2): δ [ppm] = -151.3 (br s, BF_4).

2.3 Synthesis and characterisation of [Pt(C_2H_5*C*=CFC_2H_5)(κ^3-Xantphos)][BF_4] (22)

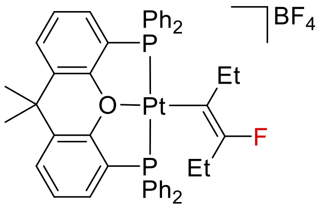

To a PTFE conic tube containing [PtCl_2(Xantphos)] (0.03 mmol, 1 eq.), AgBF_4 (0.09 mmol, 3 eq.), PVPHF (80 mg) and 3-hexyne (0.3 mmol, 10 eq.) 1,2-DCE (0.4 mL) was added. The reaction mixture was stirred at room temperature for 30 min and filtered. All volatiles were removed in *vacuo*. The crude product was washed with pentane and dried in *vacuo*. [Pt(C_2H_5*C*=CFC_2H_5)(κ^3-Xantphos)][BF_4] (**22**) was obtained as a yellow powder (28 mg, 97%). Yellow single crystals of **22** were obtained by gas phase diffusion of Et_2O into a dichloromethane solution.

^{1}H NMR (300.1 MHz, CD_2Cl_2): δ [ppm] = 7.94-7.83 (m, 4H, CH_{Ar}), 7.70-7.44 (m, 22H, CH_{Ar}), 2.07 (q, $^3J_{H,H}$ = 7 Hz, 2H, PtCCH_2), 1.84 (s, 3H, CH_3), 1.75 (s, 3H, CH_3), 1.30 (dq, $^3J_{H,F}$ = 20 Hz, $^3J_{H,H}$ = 7 Hz, 2H, CFCH_2), 0.60 (t, $^3J_{H,H}$ = 7 Hz, 3H, PtC=CH$_2$CH_3), -0.07 (t, $^3J_{H,H}$ = 7 Hz, 3H, CFCH$_2$CH_3).

^{19}F NMR (282.4 MHz, CD_2Cl_2): δ [ppm] = -105.5 (tt + sat., $^3J_{F,Pt}$ = 295 Hz, $^4J_{F,P}$ = 6 Hz, $^3J_{F,H}$ = 20 Hz, 1F, C=C*F*), -152.5 (br s, BF_4).

^{31}P{^{1}H} NMR (121.5 MHz, CD_2Cl_2): δ [ppm] = 24.8 (d + sat., $^1J_{P,Pt}$ = 2998 Hz, $^4J_{P,F}$ = 6 Hz).

MS (ESI-MS, positive mode): for [M]$^+$ Calcd.: *m*/*z* = 874.23. Found: *m*/*z* = 874.1.

2.4 Synthesis and characterisation of [Pt(η^3-(C_2H_5*C*)$_3$*C*=C_2H_4)(dppe)][BF_4] (20-L2)

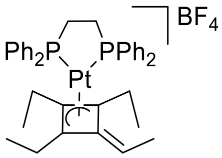

To a Schlenk flask containing [$PtCl_2$(dppe)] (199 mg, 0.3 mmol, 1 eq.), $AgBF_4$ (146 mg, 0.75 mmol, 2.5 eq.), and 3-hexyne (170 µL, 0.3 mmol, 5 eq.) 6 mL DCM were added. The reaction mixture was stirred at room temperature overnight and filtered. All volatiles were removed in *vacuo.* The crude product was washed with pentane and dried in *vacuo*. [Pt(η^3-(C_2H_5*C*)$_3$*C*=C_2H_4)(dppe)][BF_4] (**20-L2**) was obtained as a dark green powder (246 mg, 97%). Single crystals of **20-L2** were obtained by gas phase diffusion of Et_2O into a dichloromethane solution.

^{1}H NMR (300.1 MHz, CD_2Cl_2): δ [ppm] = 7.74–7.36 (m, 20H H_{aryl}), 5.12 (q+sat., $J_{H,Pt}$ = 10 Hz, $^3J_{H,H}$ = 7 Hz, 1H, C*H*-CH_3), 2.74-1.96 (m, 8H, CH_2), 2.27 (q, $^3J_{H,H}$ = 7 Hz, 2H, CH_2), 1.52 (d+sat., $^3J_{H,Pt}$ = 11 Hz, $^3J_{H,H}$ = 7 Hz, 3H, CH-CH_3), 1.10 (t, $^3J_{H,H}$ = 7 Hz, 3H, CH_3), 1.01 (t, $^3J_{H,H}$ = 7 Hz, 3H, CH_3), 0.84 (t, $^3J_{H,H}$ = 7 Hz, 3H, CH_3).

^{13}C{^{1}H} NMR (75.5 MHz, CD_2Cl_2): δ [ppm] = 140.1 (s, C_q), 134.3-129.5 (20C, C_{Ar}), 121.5 (m, C_q=CH), 105.8 (t, $^2J_{C,P}$ = 7 Hz, *C*H-CH_3), 97.7 (d, $^2J_{C,P}$ = 26 Hz, C_q), 96.3 (d, $^2J_{C,P}$ = 26 Hz, C_q), 30.8 (dd, $^1J_{C,P}$ = 27 Hz, $^2J_{C,P}$ = 11 Hz, P*C*H_2), 30.3 (dd, $^1J_{C,P}$ = 26 Hz, $^2J_{C,P}$ = 10 Hz, P*C*H_2), 20.4 (vt, *N* = 30 Hz, *C*H_2), 20.2 (vt, *N* = 34 Hz, *C*H_2), 18.4 (s, *C*H_2), 16.3 (m, *C*H_3), 15.4 (m, *C*H_3), 14.6 (s, CH-*C*H_3), 12.5 (t, *J* = 29 Hz, *C*H_3).

^{19}F NMR (121.5 MHz, CD_2Cl_2): δ [ppm] = -150.7 (br s, BF_4).

^{31}P{^{1}H} NMR (121.5 MHz, CD_2Cl_2): δ [ppm] = 43.5 (d + sat., $^1J_{P,Pt}$ = 4074 Hz, $^2J_{P,P}$ = 25 Hz), 42.4 (d + sat., $^1J_{P,Pt}$ = 4093 Hz, $^2J_{P,P}$ = 25 Hz).

^{11}B{^{1}H} NMR (160.5 MHz, CD_2Cl_2): δ [ppm] = -1.0 (s, *B*F_4).

^{195}Pt{^{1}H} NMR (64.5 MHz, CD_2Cl_2): δ [ppm] = -5575.8 (dd, $^1J_{Pt,P}$ = 4093 Hz, $^1J_{Pt,P}$ = 4074 Hz).

IR (ATR, diamond): $\tilde{\nu}$ = 1435 cm^{-1} (C=C).

MS (ESI-MS, positive mode): for [M]$^+$ Calcd.: *m*/*z* = 756.25. Found: *m*/*z* = 756.3.

2.5 Synthetic procedures for alkynes

(3-Methylbut-1-yn-1-yl)benzene

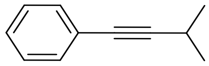

According to a literature procedure,[20] iodobenzene (0.46 mL, 4.2 mmol, 1.0 eq.), $PdCl_2(PPh_3)_2$ (175 mg, 0.25 mmol, 6 mol%) and CuI (24 mg, 0.125 mmol, 3 mol%) were suspended in triethylamine (20 mL). *Iso*-propylacetylene (0.52 mL, 5.0 mmol, 1.2 eq.) was added dropwise and the reaction mixture was stirred for 12 hours at room temperature. After addition of sat. aq. NH_4Cl (25 mL) the aqueous phase was separated and extracted with Et_2O (3 × 20 mL). The combined organic layers were dried over $MgSO_4$, filtered and concentrated *in vacuo*. The crude product was purified by column chromatography (hexane, R_f = 0.55) to give (3-methylbut-1-yn-1-yl)benzene as a yellow oil (422 mg, 71%). The spectroscopic data are in accordance with the literature.[21]

^{1}H NMR (300.1 MHz, $CDCl_3$): δ [ppm] = 7.43-7.35 (m, 2H, CH_{Ar}), 7.32-7.23 (m, 3H, CH_{Ar}), 2.78 (septett, $^3J_{H,H}$ = 7 Hz, 1H, CH), 1.27 (d, $^3J_{H,H}$ = 7 Hz, 6H, CH_3).

^{13}C{^{1}H} NMR (75.5 MHz, $CDCl_3$): δ [ppm] = 131.7 (1C, CH_{Ar}), 128.3 (1C, CH_{Ar}), 127.6 (1C, CH_{Ar}), 124.1 (1C, C_{Arq}), 95.9 (1C, C_q), 79.8 (1C, C_q), 23.2 (1C, CH_3), 21.3 (1C, CH_3).

1-Iodo-4-(prop-1-yn-1-yl)benzene

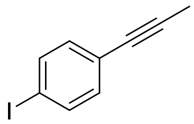

According to a literature procedure,[22] diiodobenzene (1.65 g, 5 mmol, 1.0 eq.), $PdCl_2(PPh_3)_2$ (140 mg, 0.25 mmol, 4 mol%) and CuI (95 mg, 0.5 mmol, 10 mol%) were suspended in triethylamine (6 mL). 1-Propyne (6 mL, 1 M in THF, 6.0 mmol, 1.2 eq.) was added dropwise and the reaction mixture was stirred for 12 hours at room temperature. After addition of sat. aq. NH_4Cl (25 mL) the aqueous phase was separated and extracted with Et_2O (2 × 20 mL). The combined organic layers were washed with water (2 x 10 mL) and brine, dried over $MgSO_4$, filtered and concentrated *in vacuo*. The crude product was purified by column chromatography (hexane, R_f = 0.53) to give 1-iodo-4-(prop-1-yn-1-yl)benzene as a yellow oil (557 mg, 46%). The spectroscopic data are in accordance with the literature.

^{1}H NMR (500.1 MHz, $CDCl_3$): δ [ppm] = 7.63 (d, $^3J_{H,H}$ = 8 Hz, 2H, CH_{Ar}), 7.11 (d, $^3J_{H,H}$ = 8 Hz, 2H, CH_{Ar}), 2.02 (s, 3H, CH_3).

^{13}C{^{1}H} NMR (125.8 MHz, $CDCl_3$): δ [ppm] = 137.8 (2C, CH_{Ar}), 133.4 (2C, CH_{Ar}), 124.0 (1C, C_q), 93.3 (1C, C_q), 88.0 (1C, C_q), 78.9 (1C, C_q), 4.6 (1C, CH_3).

1-Fluoro-4-(prop-1-yn-1-yl)benzene

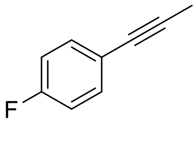

According to a literature procedure,[23] to a solution of 4-fluorophenylacetylene (360.4 mg, 4 mmol, 1.0 eq.) in THF (3 mL) *n*BuLi (2.8 mL, 1.6 M in hexane, 4.5 mmol, 1.5 eq.) was added at -78 °C. After 30 mins MeI (0.28 mL, 4.5 mmol, 1.5 eq.) was added dropwise and the reaction mixture was stirred for 6 hours at room temperature. After addition of sat. aq. NH_4Cl the aqueous phase was separated and extracted with ethyl acetate (2 × 10 mL). The combined organic layers were dried over $MgSO_4$, filtered and concentrated *in vacuo*. 1-Fluoro-4-(prop-1-yn-1-yl)benzene was obtained as a colourless oil (397 mg, 98%) and was used without further purification. The spectroscopic data are in accordance with the literature.

^{1}H NMR (300.1 MHz, CD_2Cl_2): δ [ppm] = 7.40-7.32 (m, 2H, CH_{Ar}), 7.03-6.94 (m, 2H, CH_{Ar}), 2.02 (s, 3H, CH_3).

^{19}F NMR (282.4 MHz, CD_2Cl_2): δ [ppm] = -113.2 (s, 1F).

2.6 Catalytic hydrofluorination reaction

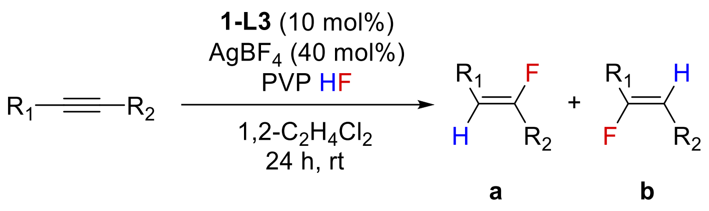

General procedure for the catalytic hydrofluorination of alkynes.

NMR Scale Procedure: The Pt precatalyst (0.03 mmol), $AgBF_4$ (23 mg, 0.12 mmol), PVPHF (100 mg) were added to a 8 mL PTFE tube with screw cap. Then, the alkyne substrate (0.3 mmol), benzotrifluoride (36 µL, 0.3 mmol) and 1,2-$C_2H_4Cl_2$ (1,2-DCE) (0.4 mL) were added under air. The reaction solution was stirred at room temperature for 24 hours and afterwards filtered into an NMR tube equipped with a PFA inliner. Trifluorotoluene (δ = -62.8 ppm) was used as an internal standard for the integration of the ^{19}F NMR spectra.

Preparative Scale: The Pt precatalyst **1-L3** (138 mg, 0.2 mmol), $AgBF_4$ (156 mg, 0.8 mmol), PVPHF (500 mg) were added to a 8 mL PTFE tube with screw cap. Then, the alkyne substrate (2 mmol) and 1,2-$C_2H_4Cl_2$ (1,2-DCE) (2.5 mL) were added under air. The reaction solution was stirred at room temperature for 24 hours and filtered and all volatiles of the filtrate removed into a cooling trap. 2.5 mL DCM was added as well as water and the mixture was stirred and then transferred into a separating funnel. The aqueous phase was separated and extracted with EtOAc (3x). The combined organic layers were washed with brine (1x), dried over $MgSO_4$, filtered and concentrated *in vacuo*. The crude product was purified by column chromatography.

Product characterisation – Fluoroalkenes

(*Z*)-1-fluoro-1-propene (3a)

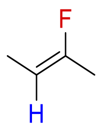

Prepared according to the general procedure using [$PtCl_2$(PCNP)] (**1-L3**) as precatalyst and 2-butyne (24 µL) under inert conditions due to the high flammability of 2-butyne. Integration of the ^{19}F NMR spectrum revealed the formation of (*Z*)-1-fluoro-1-propene (**3a**) with a yield of 45%. Note that the product is highly volatile and requires quick handling. The ^{1}H NMR data are in accordance with the literature.[24]

^{1}H NMR (300.1 MHz, CD_2Cl_2): δ [ppm] = 4.52 (dq, $^{3}J_{H(alkene),F}$ = 38 Hz, $^{3}J_{H(alkene),H}$ = 7 Hz, 1H, H_{alkene}), 1.84 (d, $^{3}J_{H,F}$ = 17 Hz, 3H, CFCH_3), 1.53 (br d, 3H, CH_3).

^{19}F NMR (282.4 MHz, CD_2Cl_2): δ [ppm] = -104.6 (dq, $^{3}J_{F,H(alkene)}$ = 38 Hz, $^{3}J_{F,H}$ = 17 Hz, 1F).

MS (GC-MS, 1,2-$C_6H_4Cl_2$): for [M] Calcd.: *m*/*z* = 74.05. Found: *m*/*z* = 74.10.

(*Z*)-3-fluoro-3-hexene (2a)

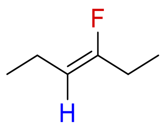

Prepared according to the general procedure using [$PtCl_2$(PCNP)] (**1-L3**) as precatalyst and 3-hexyne (34 µL). Integration of the ^{19}F NMR spectrum revealed the formation of (*Z*)-3-fluoro-3-hexene (**2a**) with a yield of 88%. The spectroscopic data are in accordance with the literature.[24-25]

^{1}H NMR (300.1 MHz, CD_2Cl_2): δ [ppm] = 4.42 (dt, $^{3}J_{H(alkene),F}$ = 38 Hz, $^{3}J_{H(alkene),H}$ = 7 Hz, 1H, H_{alkene}), 2.14-1.92 (m, 4H, CH_2), 0.96 (t, $^{3}J_{H,H}$ = 7 Hz, 3H, CH_3), 0.87 (t, $^{3}J_{H,H}$ = 7 Hz, 3H, CH_3).

^{19}F NMR (282.4 MHz, CD_2Cl_2): δ [ppm] = -110.7 (dt, $^{3}J_{F,H(alkene)}$ = 38 Hz, $^{3}J_{F,H}$ = 15 Hz, 1F).

MS (GC-MS, 1,2-$C_6H_4Cl_2$): for [M] Calcd.: *m*/*z* = 102.08. Found: *m*/*z* = 102.10; for [CH_2CH=CFEt]$^{+}$ Calcd.: *m*/*z* = 87.06. Found: *m*/*z* = 87.08.

(*Z*)-4-fluoro-4-octene (4a)

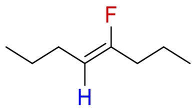

Prepared according to the general procedure using [$PtCl_2$(PCNP)] (**1-L3**) as precatalyst and 4-octyne (44 µL). Integration of the ^{19}F NMR spectrum revealed the formation of (*Z*)-4-fluoro-4-octene (**4a**) with a yield of 84%. The spectroscopic data are in accordance with the literature.[26]

^{1}H NMR (300.1 MHz, CD_2Cl_2): δ [ppm] = 4.50 (dt, $^{3}J_{H(alkene),F}$ = 38 Hz, $^{3}J_{H(alkene),H}$ = 7 Hz, 1H, H_{alkene}), 2.11 (dt, $^{3}J_{H,F}$ = 18 Hz, $^{3}J_{H,H}$ = 7 Hz, 2H, CH_2CF), 2.02 (dt, $^{3}J_{H,H}$ = 7 Hz, $^{3}J_{H,H}$ = 7 Hz, 2H, CH_2CH), 1.49 (q, $^{3}J_{H,H}$ = 7 Hz, 2H, CH_2), 1.36 (q, $^{3}J_{H,H}$ = 7 Hz, 2H, CH_2), 0.92 (t, $^{3}J_{H,H}$ = 7 Hz, 3H, CH_3), 0.90 (t, $^{3}J_{H,H}$ = 7 Hz, 3H, CH_3).

^{19}F NMR (282.4 MHz, CD_2Cl_2): δ [ppm] = -110.5 (dt, $^{3}J_{F,H(alkene)}$ = 38 Hz, $^{3}J_{F,H}$ = 18 Hz, 1F).

MS (GC-MS, 1,2-$C_6H_4Cl_2$): for [M] Calcd.: *m*/*z* = 130.12. Found: *m*/*z* = 130.30.

(*Z*)-6-fluoro-6-dodecene (5a)

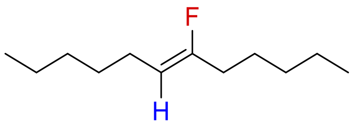

Prepared according to the general procedure using [$PtCl_2$(PCNP)] (**1-L3**) as precatalyst and 6-dodecyne (36 µL for the NMR scale procedure, 240 µL for the preparative procedure). The crude product was purified by column chromatography (hexane, R_f = 0.81) to give (*Z*)-6-fluoro-6-dodecene (**5a**) as a colourless oil (80%; ^{19}F NMR revealed 88%). The spectroscopic data are in accordance with the literature.[27]

^{1}H NMR (300.1 MHz, CD_2Cl_2): δ [ppm] = 4.49 (dt, $^{3}J_{H(alkene),F}$ = 38 Hz, $^{3}J_{H(alkene),H}$ = 7 Hz, 1H, H_{alkene}), 2.12 (dt, $^{3}J_{H,F}$ = 18 Hz, $^{3}J_{H,H}$ = 7 Hz, 2H, CH_2CF), 2.03 (dt, $^{3}J_{H,H}$ = 7 Hz, $^{3}J_{H,H}$ = 7 Hz, 2H, CHCH_2), 1.47 (tt, $^{3}J_{H,H}$ = 7 Hz, $^{3}J_{H,H}$ = 7 Hz, 2H, CH_2), 1.38-1.22 (m, 10H, CH_2), 0.93-0.84 (m, 6H, CH_3).

^{19}F NMR (282.4 MHz, CD_2Cl_2): δ [ppm] = -110.6 (dt, $^{3}J_{F,H(alkene)}$ = 38 Hz, $^{3}J_{F,H}$ = 18 Hz, 1F).

MS (GC-MS, 1,2-$C_6H_4Cl_2$): for [M] Calcd.: *m*/*z* = 186.18. Found: *m*/*z* = 186.20.

(*Z*)-3-fluoro-2-heptene (6a)

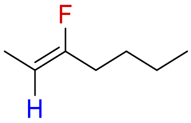

Prepared according to the general procedure using [$PtCl_2$(PCNP)] (**1-L3**) as precatalyst and 2-heptyne (38 μL). Integration of the ^{19}F NMR spectrum revealed the formation of (*Z*)-3-fluoro-2-heptene (**6a**) and (*Z*)-2-fluoro-2-heptene (**6b**) as a mixture of isomers in a ratio of 1.7:1 with a yield of 70%. The spectroscopic data are in accordance with the literature.[26]

Selected analytical data:

(*Z*)-3-fluoro-2-heptene (6a)

^{1}H NMR (300.1 MHz, CD_2Cl_2): δ [ppm] = 4.51 (dq, $^{3}J_{H(alkene),F}$ = 38 Hz, 1H, H_{alkene}), 2.12 (dt, $^{3}J_{H,F}$ = 18 Hz, $^{3}J_{H,F}$ = 7 Hz, 2H, CH_2CF=CH).

^{19}F NMR (282.4 MHz, CD_2Cl_2): δ [ppm] = -108.8 (dt, $^{3}J_{F,H(alkene)}$ = 38 Hz, $^{3}J_{F,H}$ = 18 Hz, 1F).

MS (GC-MS, 1,2-$C_6H_4Cl_2$): for [M] Calcd.: *m*/*z* = 116.10. Found: *m*/*z* = 116.15.

(*Z*)-2-fluoro-2-heptene (6b)

^{1}H NMR (300.1 MHz, CD_2Cl_2): δ [ppm] = 1.84 (d, $^{3}J_{H,F}$ = 18 Hz, 3H, CF=CH_3).

^{19}F NMR (282.4 MHz, CD_2Cl_2): δ [ppm] = -102.1 (dq, $^{3}J_{F,H(alkene)}$ = 38 Hz, $^{3}J_{F,H}$ = 17 Hz, 1F).

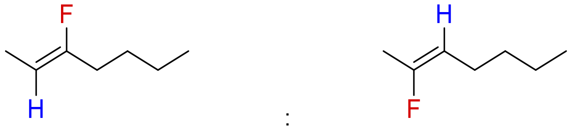

1.7 : 1

(*Z*)-4-fluoro-4-phenylbut-3-en-2-one (7a)

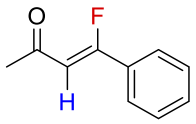

Prepared according to the general procedure using [$PtCl_2$(PCNP)] (**1-L3**) as precatalyst and 4-phenyl-3-butyn-2-one (50 µL). Integration of the ^{19}F NMR spectrum revealed the formation of (*Z*)-4-fluoro-4-phenylbut-3-en-2-one (**7a**), (*E*)-4-fluoro-4-phenylbut-3-en-2-one and 4,4-difluoro-4-phenylbutan-2-one in a ratio of 8:1:1 with a yield of 43%. The spectroscopic data for **7a** are in accordance with the literature.[28]

Selected analytical data:

(*Z*)-4-Fluoro-4-phenylbut-3-en-2-one (7a)

^{1}H NMR (300.1 MHz, CD_2Cl_2): δ [ppm] = 6.15 (d, $^3J_{H(alkene),F}$ = 39 Hz, 1H, H_{alkene}), 2.50 (d, $^5J_{H,F}$ = 4 Hz, 3H, CH_3).

^{19}F NMR (282.4 MHz, CD_2Cl_2): δ [ppm] = -94.6 (dm, $^3J_{F,H(alkene)}$ = 39 Hz, 1F).

MS (GC-MS, 1,2-$C_6H_4Cl_2$): for [M] Calcd.: *m*/*z* = 164.06 Found: *m*/*z* = 164.10.

(*E*)-4-Fluoro-4-phenylbut-3-en-2-one

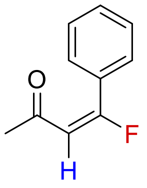

^{1}H NMR (300.1 MHz, CD_2Cl_2): δ [ppm] = 6.21 (d, $^3J_{H(alkene),F}$ = 21 Hz, 1H, H_{alkene}).

^{19}F NMR (282.4 MHz, CD_2Cl_2): δ [ppm] = -76.4 (dm, $^3J_{F,H(alkene)}$ = 21 Hz, 1F).

4,4-Difluoro-4-phenylbutan-2-one

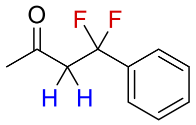

^{1}H NMR (300.1 MHz, CD_2Cl_2): δ [ppm] = 3.33 (t, $^3J_{H(alkene),F}$ = 16 Hz, 2H, CH_2).

^{19}F NMR (282.4 MHz, CD_2Cl_2): δ [ppm] = -91.9 (t, $^3J_{F,H(alkene)}$ = 16 Hz, 2F).

MS (GC-MS, 1,2-$C_6H_4Cl_2$): for [M] Calcd.: *m*/*z* = 184.07 Found: *m*/*z* = 184.20.

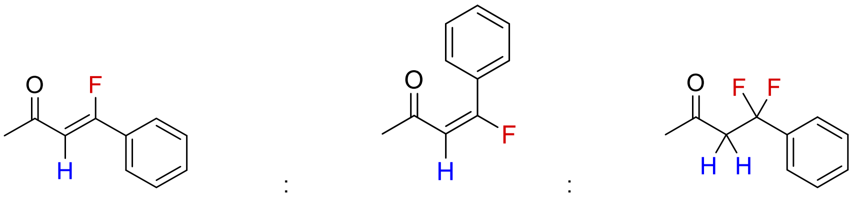

8 : 1 : 1

(*Z*)-Ethyl-3-fluorobut-2-enoate (8a)

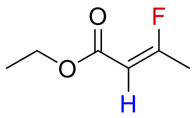

Prepared according to the general procedure using [$PtCl_2$(PCNP)] (**1-L3**) as precatalyst and ethyl 2-butynoate (35 µL). Integration of the ^{19}F NMR spectrum revealed the formation of (*Z*)-ethyl-3-fluorobut-2-enoate (**8a**) with a yield of 45%. The spectroscopic data are in accordance with the literature.[26]

^{1}H NMR (300.1 MHz, CD_2Cl_2): δ [ppm] = 5.19 (dd, $^3J_{H(alkene),F}$ = 33 Hz, $^3J_{H(alkene),H}$ = 1 Hz, 1H, H_{alkene}), 4.13 (q, $^3J_{H,H}$ = 7 Hz, 2H, CH_2), 2.01 (dd, $^3J_{H,F}$ = 17 Hz, $^3J_{H,H}$ = 1 Hz, 3H, CFCH_3), 1.26 (t, $^3J_{H,H}$ = 7 Hz, 3H, CH_2CH_3).

^{19}F NMR (282.4 MHz, CD_2Cl_2): δ [ppm] = -73.8 (dq, $^3J_{F,H(alkene)}$ = 33 Hz, $^3J_{F,H}$ = 17 Hz, 1F).

MS (GC-MS, 1,2-$C_6H_4Cl_2$): for [M] Calcd.: *m*/*z* = 132.06. Found: *m*/*z* = 132.10.

(*Z*)-(2-fluoroprop-1-en-1-yl)benzene (9a)

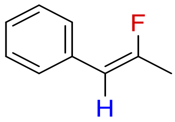

Prepared according to the general procedure using [$PtCl_2$(PCNP)] (**1-L3**) as precatalyst and 1-phenylpropyne (38 µL). Integration of the ^{19}F NMR spectrum revealed the formation of (*Z*)-(2-fluoroprop-1-en-1-yl)benzene (**9a**) and (*Z*)-(1-fluoroprop-1-en-1-yl)benzene (**9b**) as a mixture of isomers in a ratio of 2:1 with a yield of 90%. The spectroscopic data are in accordance with the literature.[29]

Selected analytical data:

(*Z*)-(2-Fluorohex-1-en-1-yl)benzene (10a)

^{1}H NMR (300.1 MHz, CD_2Cl_2): δ [ppm] = 5.51 (d, $^{3}J_{H(alkene),F}$ = 40 Hz, 1H, H_{alkene}), 2.34 (dt, $^{3}J_{H,F}$ = 18 Hz, $^{3}J_{H,H}$ = 8 Hz, 2H, CH_2CF), 0.96 (t, $^{3}J_{H,H}$ = 7 Hz, 3H, CH_3).

^{19}F NMR (282.4 MHz, CD_2Cl_2): δ [ppm] = -101.2 (dt, $^{3}J_{F,H(alkene)}$ = 40 Hz, $^{3}J_{F,H}$ = 18 Hz, 1F).

MS (GC-MS, 1,2-$C_6H_4Cl_2$): for [M-Ph]$^+$ [CF=CH$(CH_2)_4CH_3$]$^+$ Calcd.: *m*/*z* = 115.09 Found: *m*/*z* = 115.10.

(*Z*)-(1-Fluorohex-1-en-1-yl)benzene (10b)

^{1}H NMR (300.1 MHz, CD_2Cl_2): δ [ppm] = 5.45 (dt, $^{3}J_{H(alkene),F}$ = 38 Hz, $^{3}J_{H(alkene),H}$ = 8 Hz, 1H, H_{alkene}).

^{19}F NMR (282.4 MHz, CD_2Cl_2): δ [ppm] = -122.1 (dm, $^{3}J_{F,H(alkene)}$ = 37 Hz, 1F).

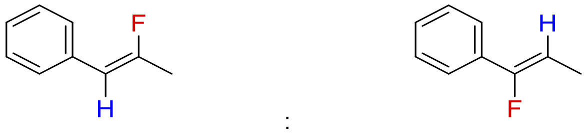

2 : 1

(*Z*)-(2-Fluorohex-1-en-1-yl)benzene (10a)

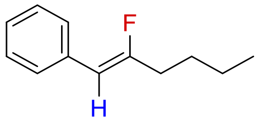

Prepared according to the general procedure using [$PtCl_2$(PCNP)] (**1-L3**) as precatalyst and 1-phenyl-1-hexyne (53 µL). Integration of the ^{19}F NMR spectrum revealed the formation of (*Z*)-(2-fluorohex-1-en-1-yl)benzene (**15a**) and (*Z*)-(1-fluorohex-1-en-1-yl)benzene (**10b**) as a mixture of isomers in a ratio of 8.3:1 with a yield of 90%. The spectroscopic data are in accordance with the literature.[30-31]

Selected analytical data:

(*Z*)-(2-Fluorohex-1-en-1-yl)benzene (10a)

^{1}H NMR (300.1 MHz, $CDCl_3$): δ [ppm] = 7.62 (d, $^3J_{H,H}$ = 8 Hz, 2H, CH_{Ar}), 7.18 (d, $^3J_{H,H}$ = 8 Hz, 2H, CH_{Ar}), 5.44 (d, $^3J_{H(alkene),F}$ = 39 Hz, 1H, H_{alkene}), 2.06 (dd, $^3J_{H,F}$ = 17 Hz, $^4J_{H,H(alkene)}$ = 1 Hz, 3H, CH_3).

^{19}F NMR (282.4 MHz, $CDCl_3$): δ [ppm] = -92.4 (dq, $^3J_{F,H(alkene)}$ = 39 Hz, $^3J_{F,H}$ = 17 Hz, 1F).

MS (GC-MS, 1,2-$C_6H_4Cl_2$): for $[M\text{-}Ph]^+$ $[CF{=}CH(CH_2)_4CH_3]^+$ Calcd.: *m*/*z* = 115.09 Found: *m*/*z* = 115.10.

(*Z*)-(1-Fluorohex-1-en-1-yl)benzene (10b)

^{1}H NMR (300.1 MHz, CD_2Cl_2): δ [ppm] = 5.45 (dt, $^3J_{H(alkene),F}$ = 38 Hz, $^3J_{H(alkene),CH2}$ = 8 Hz, 1H, CH_{alkene}).

^{19}F NMR (282.4 MHz, CD_2Cl_2): δ [ppm] = -122.2 (d, $^3J_{F,H(alkene)}$ = 38 Hz, 1F).

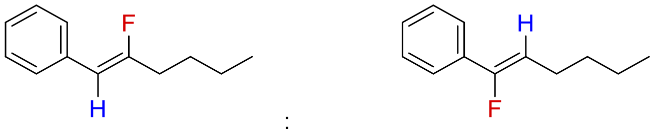

8.3 : 1

(*Z*)-1-(2-Fluoroprop-1-en-1-yl)-4-iodobenzene (11a)

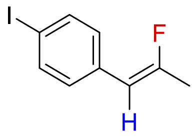

Prepared according to the general procedure using [$PtCl_2$(PCNP)] (**1-L3**) as precatalyst and 1-iodo-4-(prop-1-yn-1-yl)benzene (73 mg for the NMR scale procedure, 218 mg for the preparative procedure on a 0.9 mmol scale). The crude product was purified by column chromatography (hexane, R_f = 0.8) to give (*Z*)-1-(2-fluoroprop-1-en-1-yl)-4-iodobenzene (**11a**) and (*Z*)-1-(1-fluoroprop-1-en-1-yl)-4-iodobenzene (**11b**) as a mixture of isomers in a ratio of 5:1 as a pale-yellow solid (435 mg, 83%; ^{19}F NMR revealed 90%).

(*Z*)-1-(2-Fluoroprop-1-en-1-yl)-4-iodobenzene (11a)

^{1}H NMR (300.1 MHz, $CDCl_3$): δ [ppm] = 7.62 (d, $^3J_{H,H}$ = 8 Hz, 2H, H_{Ar}), 7.18 (d, $^3J_{H,H}$ = 8 Hz, 2H, H_{Ar}), 5.44 (d, $^3J_{H(alkene),F}$ = 39 Hz, 1H, H_{alkene}), 2.06 (dd, $^3J_{H(alkene),F}$ = 17 Hz, $^4J_{H(alkene),H}$ = 1 Hz, 3H, CH_3).

^{19}F NMR (282.4 MHz, $CDCl_3$): δ [ppm] = -92.4 (dq, $^3J_{F,H(alkene)}$ = 39 Hz, $^3J_{F,H(alkene)}$ = 17 Hz, 1F).

^{13}C{^{1}H} NMR (125.8 MHz, $CDCl_3$): δ [ppm] = 159.3 (d, $^1J_{C,F}$ = 266 Hz, C_{vinyl}F), 137.8 (s, CH_{Ar}), 133.4 (s, C_{Ar}), 130.3 (s, CH_{Ar}), 125.8 (s, C_{Ar}), 105.7 (d, $^2J_{C,F}$ = 8 Hz, C_{vinyl}H), 19.2 (d, $^2J_{C,F}$ = 29 Hz, CH_3).

MS (GC-MS, CH_2Cl_2): for [M] Calcd.: *m*/*z* = 261.97 Found: *m*/*z* = 262.15.

Selected NMR data:

(*Z*)-1-(1-Fluoroprop-1-en-1-yl)-4-iodobenzene (11b)

^{1}H NMR (300.1 MHz, CD_2Cl_2): δ [ppm] = 5.45 (dq, $^3J_{H(alkene),F}$ = 37 Hz, $^3J_{H(alkene),H}$ = 7 Hz, 1H, H_{alkene}), 1.79 (dd, $^3J_{H,H(alkene)}$ = 7 Hz, $^3J_{H(alkene),F}$ = 2 Hz, 3H, CH_3).

^{19}F NMR (282.4 MHz, CD_2Cl_2): δ [ppm] = -122.9 (d, $^3J_{F,H(alkene)}$ = 37 Hz, 1F).

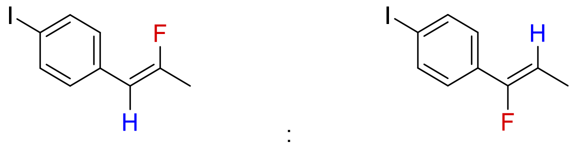

5 : 1

(*Z*)-1-(2-Fluoroprop-1-en-1-yl)-4-fluorobenzene (12a)

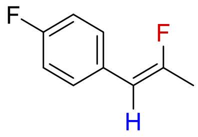

Prepared according to the general procedure using [$PtCl_2$(PCNP)] (**1-L3**) as precatalyst and 1-fluoro-4-(prop-1-yn-1-yl)benzene (40 mg). Integration of the ^{19}F NMR spectrum revealed the formation of (*Z*)-1-(2-fluoroprop-1-en-1-yl)-4-fluorobenzene (**12a**) and (*Z*)-1-(1-fluoroprop-1-en-1-yl)-4-fluorobenzene (**12b**) as a mixture of isomers in a ratio of 1.6:1 with a yield of 36%.

Selected NMR data:

(*Z*)-1-(2-Fluoroprop-1-en-1-yl)-4-fluorobenzene (12a)

^{1}H NMR (300.1 MHz, $CDCl_3$): δ [ppm] = 5.46 (d, $^3J_{\mathrm{H(alkene),F}}$ = 38 Hz, 1H, H_{alkene}), 2.05 (d, $^3J_{\mathrm{H(alkene),F}}$ = 17 Hz, 3H, CH_3).

^{19}F NMR (282.4 MHz, $CDCl_3$): δ [ppm] = -96.0 (dq, $^3J_{\mathrm{F,H(alkene)}}$ = 38 Hz, $^3J_{\mathrm{F,H(alkene)}}$ = 17 Hz, 1F, $F_{\mathrm{(alkene)}}$), -115.7 (s, 1F, F_{Ar}).

MS (GC-MS, 1,2-$C_6H_4Cl_2$): for [M] Calcd.: *m*/*z* = 154.06 Found: *m*/*z* = 154.15.

Selected NMR data:

(*Z*)-1-(1-Fluoroprop-1-en-1-yl)-4-fluorobenzene (12b)

^{1}H NMR (300.1 MHz, $CDCl_3$): δ [ppm] = 5.38 (dq, $^3J_{\mathrm{H(alkene),F}}$ = 37 Hz, $^3J_{\mathrm{H(alkene),H}}$ = 7 Hz, 1H, H_{alkene}), 1.78 (dd, $^3J_{\mathrm{H,H(alkene)}}$ = 7 Hz, $^3J_{\mathrm{H(alkene),F}}$ = 2 Hz, 3H, CH_3).

^{19}F NMR (282.4 MHz, $CDCl_3$): δ [ppm] = -121.1 (d, $^3J_{\mathrm{F,H(alkene)}}$ = 37 Hz, 1F, $F_{\mathrm{(alkene)}}$), -113.6 (s, 1F, F_{Ar}).

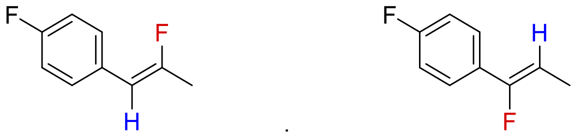

1.6 : 1

(*Z*)-2-Fluoro-1-hexene (13a)

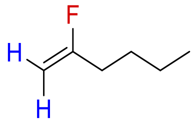

Prepared according to the general procedure using [$PtCl_2$(PCNP)] (**1-L3**) as precatalyst and 1-hexyne (34 µL). The reaction mixture was heated up for 144 hours at 60 °C. Integration of the ^{19}F NMR spectrum revealed the formation of (*Z*)-2-fluoro-1-hexene (**13a**) with a yield of 60%. The spectroscopic data are in accordance with the literature.[32]

Selected NMR data:

^{19}F NMR (282.4 MHz, CD_2Cl_2): δ [ppm] = -90.2 (dtd, $^3J_{\mathrm{F,H(alkene)}}$ = 50 Hz, $^3J_{\mathrm{F,H}}$ = 18 Hz, $^3J_{\mathrm{F,H(alkene)}}$ = 18 Hz, 1F).

(*Z*)-(2-Fluoro-3-methylbut-1-en-1-yl)benzene (14a)

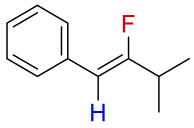

Prepared according to the general procedure using [$PtCl_2$(PCNP)] (**1-L3**) as precatalyst and (3-methyl-1-butyn-1-yl)benzene (43 mg). Integration of the ^{19}F NMR spectrum revealed the formation of (*Z*)-(2-fluoro-3-methylbut-1-en-1-yl)benzene (**14a**) with a yield of 44%.

Selected analytical data:

^{1}H NMR (300.1 MHz, $CDCl_3$): δ [ppm] = 5.49 (d, $^{3}J_{H(alkene),F}$ = 41 Hz, 1H, H_{alkene}), 2.56 (dsept, $^{3}J_{H,F}$ = 16 Hz, $^{3}J_{H,H}$ = 7 Hz, 1H, C*H*), 1.81 (d, $^{3}J_{H,H}$ = 7 Hz, 6H, CH_3).

^{19}F NMR (282.4 MHz, $CDCl_3$): δ [ppm] = -106.9 (dm, $^{3}J_{F,H(alkene)}$ = 41 Hz, 1F).

MS (GC-MS, 1,2-$C_6H_4Cl_2$): for [M] Calcd.: *m*/*z* = 164.10. Found: *m*/*z* = 146.30.

(*Z*)-3-Fluoro-4-methylpent-2-ene (15a)

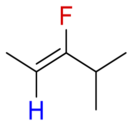

Prepared according to the general procedure using [$PtCl_2$(PCNP)] (**1-L3**) as precatalyst and 4-methylpentyne (30 µL). Integration of the ^{19}F NMR spectrum revealed the formation of (*Z*)-3-fluoro-4-methylpent-2-ene (**15a**) and (*Z*)-2-fluoro-4-methylpent-2-ene (**15b**) as a mixture of isomers in a ratio of 3.1:1 with a yield of 61%.

(*Z*)-3-Fluoro-4-methylpent-2-ene (15a)

^{1}H NMR (300.1 MHz, CD_2Cl_2): δ [ppm] = 4.53 (dq, $^{3}J_{H(alkene),F}$ = 39 Hz, $^{3}J_{H(alkene),H}$ = 7 Hz, 1H, H_{alkene}), 2.37 (dsept, $^{3}J_{H,F}$ = 15 Hz, $^{3}J_{H,H}$ = 7 Hz, 1H, C*H*), 1.55 (dd, $^{3}J_{H,H}$ = 7 Hz, $^{4}J_{H,F}$ = 1 Hz, 3H, CH_3), 1.05 (d, $^{3}J_{H,H}$ = 7 Hz, 6H, CH_3).

^{19}F NMR (282.4 MHz, CD_2Cl_2): δ [ppm] = -116.0 (dd, $^{3}J_{F,H(alkene)}$ = 39 Hz, $^{3}J_{F,H}$ = 15 Hz, 1F).

(*Z*)-2-Fluoro-4-methylpent-2-ene (15b)

^{1}H NMR (300.1 MHz, CD_2Cl_2): δ [ppm] = 4.36 (dd, $^{3}J_{H(alkene),F}$ = 39 Hz, $^{3}J_{H(alkene),H}$ = 9 Hz, 1H, H_{alkene}), 2.70 (dsept, $^{3}J_{H,H}$ = 9 Hz, $^{3}J_{H,H}$ = 7 Hz, 1H, C*H*), 1.83 (d, $^{3}J_{H,F}$ = 17 Hz, 3H, CH_3), 0.95 (d, $^{3}J_{H,H}$ = 7 Hz, 6H, CH_3).

^{19}F NMR (282.4 MHz, CD_2Cl_2): δ [ppm] = -104.4 (dq, $^{3}J_{F,H(alkene)}$ = 39 Hz, $^{3}J_{F,H}$ = 17 Hz, 1F).

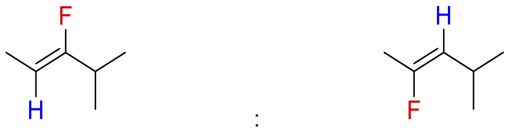

3.1 : 1

(*Z*)-3-Fluoro-4,4-dimethylpent-2-ene (16a)

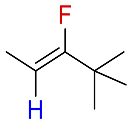

Prepared according to the general procedure using [$PtCl_2$(PCNP)] (**1-L3**) as precatalyst and 2-heptyne (38 µL). Integration of the ^{19}F NMR spectrum revealed the formation of (*Z*)-3-fluoro-4,4-dimethylpent-2-ene (**16a**) and (*E*)-3-fluoro-4,4-dimethylpent-2-ene as a mixture of isomers in a ratio of 3:1 with a yield of 19%.

(*Z*)-3-Fluoro-4,4-dimethylpent-2-ene (16a)

^{1}H NMR (300.1 MHz, CD_2Cl_2): δ [ppm] = 4.57 (dq, $^{3}J_{H(alkene),F}$ = 39 Hz, $^{3}J_{H(alkene),H}$ = 7 Hz, 1H, H_{alkene}), 1.53 (dd, $^{3}J_{H,H(alkene)}$ = 7 Hz, $^{4}J_{H,F}$ = 2 Hz, 3H, CH_3), 1.07 (s, 9H, CH_3).

^{19}F NMR (282.4 MHz, CD_2Cl_2): δ [ppm] = -119.5 (dm, $^{3}J_{F,H(alkene)}$ = 39 Hz, 1F).

MS (GC-MS, 1,2-$C_6H_4Cl_2$): for [M] Calcd.: *m*/*z* = 116.10. Found: *m*/*z* = 116.10.

(*E*)-3-Fluoro-4,4-dimethylpent-2-ene

^{1}H NMR (300.1 MHz, CD_2Cl_2): δ [ppm] = 4.97 (dq, $^{3}J_{H(alkene),F}$ = 29 Hz, $^{3}J_{H(alkene),H}$ = 7 Hz, 1H, H_{alkene}), 1.64 (dd, $^{3}J_{H,H(alkene)}$ = 7 Hz, $^{4}J_{H,F}$ = 2 Hz, 3H, CH_3).

^{19}F NMR (282.4 MHz, CD_2Cl_2): δ [ppm] = -104.5 (dm, $^{3}J_{F,H(alkene)}$ = 29 Hz, 1F).

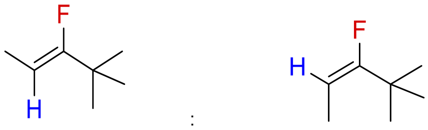

3 : 1

Unsuccessful substrates

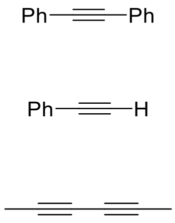	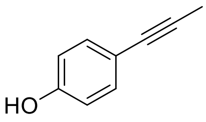	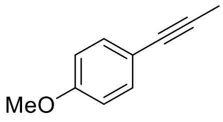	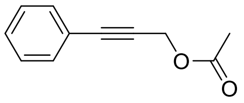

Table S1. Examples of unsuccessful substrates for the Pt-catalysed hydrofluorination reaction under the reported reaction conditions at room temperature.

Formation of (*Z*)-4-(2-fluoroprop-1-en-1-yl)phenol (17a)

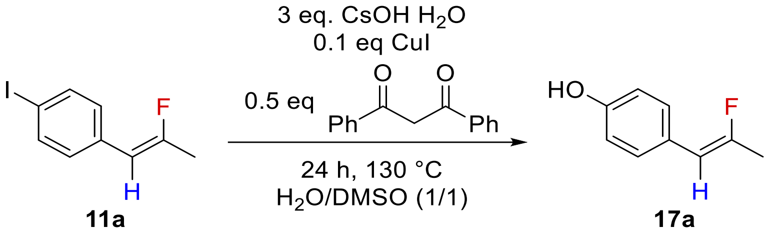

11a **17a**

According to a literature procedure,[33] **11a** (containing approximately 17% **11b**) (97 mg, 0.37 mmol, 1 eq.), CsOH·H_2O (166 mg, 1.11 mmol, 3 eq.), CuI (7 mg, 0.037 mmol, 0.1 eq.) and dibenzoylmethane (42 mg, 0.185 mmol, 0.5 eq.) were added into a Schlenk tube, which was evacuated and back-filled with argon three times. Degassed H_2O (0.37 mL) and DMSO (0.37 mL) were added. The tube was heated 24 hours at 130 °C. The reaction mixture was allowed to cool down to room temperature and DCM (3.7 mL) and HCl (37%, 0.37 mL) were added and the tube stirred for 2 hours. The yellow aqueous phase was separated and extracted three times with DCM. The combined organic layers extracted two times with water, dried over $MgSO_4$, filtered and the filtrate was concentrated *in vacuo*. The crude product was analysed revealing the formation of (*Z*)-4-(2-fluoroprop-1-en-1-yl)phenol (**17a**) and traces of two unidentified fluorinated compounds.

^{1}H NMR (500.1 MHz, $CDCl_3$): δ [ppm] = 7.34 (d, $^3J_{H,H}$ = 9 Hz, 2H, CH_{Ar}), 6.78 (d, $^3J_{H,H}$ = 9 Hz, 2H, CH_{Ar}), 5.39 (d, $^3J_{H(alkene),F}$ = 40 Hz, 1H, H_{alkene}), 5.18 (br s, 1H, O*H*), 2.05 (d, $^3J_{H,F}$ = 17 Hz, 3H, CH_3).

^{19}F NMR (282.4 MHz, $CDCl_3$): δ [ppm] = -98.0 (dq, $^3J_{F,H(alkene)}$ = 40 Hz, $^3J_{F,H(alkene)}$ = 17 Hz, 1F).

3 Precatalyst screening and hydrofluorination of 3-hexyne

For precatalyst screening, the Pt complex (0.03 mmol), $AgBF_4$ (23 mg, 0.12 mmol), PVPHF (100 mg) were added to a 8 mL PTFE tube with screw cap. Then, 3-hexyne (34 µL 0.3 mmol), benzotrifluoride (36 µL, 0.3 mmol) and 1,2-$C_2H_4Cl_2$ (1,2-DCE) (0.4 mL) were added under air. The reaction solution was stirred at room temperature for 24 hours and afterwards filtered into an NMR tube equipped with a PFA inliner.

3.1 Precatalyst screening

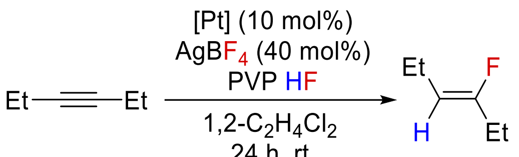

2a

Table S2. Precatalyst screening for the hydrofluorination of 3-hexyne.

Entry	[Pt]	[Pt] load [mol%]	$AgBF_4$ load [mol%]	Temperature [°C]	Time [h]	Yield [%][a]
1	[$PtCl_2$(d(Ind)pe)]	10	40	60	24	
2	[$PtCl_2$(d(Ind)pe)]	10	40	rt	24	0
3	[PtI_2(dppe)]	10	40	rt	24	0
4	[PtMeCl(dppe)]	10	40	rt	24	73
5	[PtMeCl(PCNP)]	10	40	rt	24	78
6	[$PtMe_2$(dppe)]	10	40	rt	24	58
7	[$PtCl_2(PPh_3)_2$]	10	40	rt	24	traces
8	[$PtCl_2\{Ph_2P(Ind)\}_2$]	10	40	rt	24	traces
9	[$PtCl_2$(dppm)] (**1-L1**)	10	40	rt	24	68
10	[$PtCl_2$(dppe)] (**1-L2**)	10	40	rt	24	73
11	[$PtCl_2$(dcpe)] (**1-L2**)	10	40	rt	24	traces
12	[$PtCl_2$(PCNP)] (**1-L3**)	10	40	rt	24	88
13	[$PtCl_2$(dppp)] (**1-L4**)	10	40	rt	24	62
14	[$PtCl_2$(dppb)] (**1-L5**)	10	40	rt	24	traces
15	[$PtCl_2$(dppf)] (**1-L6**)	10	40	rt	24	traces
16	[$PtCl_2$(Xantphos)] (**1-L7**)	10	40	rt	24	0
17	-	0	40	rt	24	0
18	**1-L3**	5	20	rt	24	29
19	**1-L3**	5	20	rt	72	60
20	**1-L3**	5	20	rt	144	60
21	**1-L3**	7.5	30	rt	24	6
22	**1-L3**	7.5	30	rt	48	90
23	**18-L2**	10	0	rt	24	traces
24	**18-L2**	10	20	rt	24	72
25	**18-L3**	10	0	rt	24	traces
26	**18-L3**	10	20	rt	24	87

27	**20-L3**	10	0	rt	24	0
28	**20-L3**	10	20	rt	24	traces

[a] NMR yield.

3.2 Optimisation of the reaction conditions

Precatalysts [$PtCl_2$(dppe)] (**1-L2**) and [$PtCl_2$(PCNP)] (**1-L3**) proved to exhibit the highest catalytic reactivity. Both precatalysts were chosen for further optimisation of the reaction conditions. Based on these results precatalyst **1-L3** demonstrates the highest catalytic activity and was therefore chosen for scope extension.

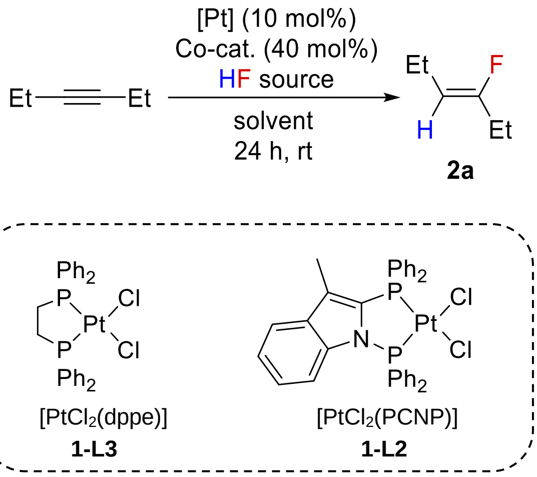

Table S3. Complete optimisation results for the hydrofluorination of 3-hexyne.

Entry	[Pt]	Anion source (load) [mol%]	Solvent	Reaction container	HF source	Yield [%][a]
1	**1-L2**	$AgBF_4$ (40)	1,2-DCE	PE vial	NEt_3·HF	48
2	**1-L2**	$AgBF_4$ (40)	1,2-DCE	PE vial	PVPHF	63
3	**1-L2**	$AgBF_4$ (40)	1,2-DCE	PTFE conic tube	PVPHF	73
4	**1-L3**	$AgBF_4$ (40)	1,2-DCE	PE vial	NEt_3·HF	50
5	**1-L3**	$AgBF_4$ (40)	1,2-DCE	PE vial	PVPHF	73
6	**1-L3**	$AgBF_4$ (40)	1,2-DCE	PTFE conic tube	PVPHF	88
7	**1-L3**	$AgBF_4$ (40)	1,2-DCE	PTFE conic tube	Aq. HF (48)	9
8	**1-L3**	$AgBF_4$ (40)	1,2-DCE	PTFE conic tube	PVPHF	88
9	**1-L3**	$AgBF_4$ (40)	CD_2Cl_2	PTFE conic tube	PVPHF	75
10	**1-L3**	$AgBF_4$ (40)	Tol-d_8	PTFE conic tube	PVPHF	55
11	**1-L3**	$AgBF_4$ (40)	NO_2Me	PTFE conic tube	PVPHF	traces
12	**1-L3**	$AgBF_4$ (40)	HFIP	PTFE conic tube	PVPHF	0
13	**1-L3**	$AgBF_4$ (40)	MeCN	PTFE conic tube	PVPHF	traces
14	**1-L3**	0	1,2-DCE	PTFE conic tube	PVPHF	0
15	**1-L3**	$AgBF_4$ (10)	1,2-DCE	PTFE conic tube	PVPHF	traces
16	**1-L2**	$AgBF_4$ (10)	1,2-DCE	PTFE conic tube	PVPHF	traces
17	**1-L2**	$AgBF_4$ (20)	1,2-DCE	PTFE conic tube	PVPHF	12
18	**1-L3**	$AgBF_4$ (30)	1,2-DCE	PTFE conic tube	PVPHF	56

19	**1-L2**	$AgBF_4$ (30)	1,2-DCE	PTFE conic tube	PVPHF	46
20	**1-L3**	$AgBF_4$ (35)	1,2-DCE	PTFE conic tube	PVPHF	63
21	**1-L3**	$AgBF_4$ (40)	1,2-DCE	PTFE conic tube	PVPHF	88
22	**1-L2**	$AgBF_4$ (40)	1,2-DCE	PTFE conic tube	PVPHF	74
23	**1-L3**	$AgBF_4$ (45)	1,2-DCE	PTFE conic tube	PVPHF	85
24	**1-L3**	AgFHF (40)	1,2-DCE	PTFE conic tube	PVPHF	86
25	**1-L2**	AgFHF (40)	1,2-DCE	PTFE conic tube	PVPHF	74
26	**1-L3**	$AgPF_6$ (10)	1,2-DCE	PTFE conic tube	PVPHF	69
27	**1-L2**	$AgPF_6$ (10)	1,2-DCE	PTFE conic tube	PVPHF	58
28	**1-L3**	$AgSbF_6$ (10)	1,2-DCE	PTFE conic tube	PVPHF	35
29	**1-L2**	$AgSbF_6$ (10)	1,2-DCE	PTFE conic tube	PVPHF	25
30	**1-L3**	AgOTf (10)	1,2-DCE	PTFE conic tube	PVPHF	26
31	**1-L2**	AgOTf (10)	1,2-DCE	PTFE conic tube	PVPHF	17
32	**1-L3**	$AgBAr^F{}_4$ (10)	1,2-DCE	PTFE conic tube	PVPHF	0
33	**1-L2**	$AgBAr^F{}_4$ (10)	1,2-DCE	PTFE conic tube	PVPHF	0
34	**1-L3**	$NaBF_4$ (10)	1,2-DCE	PTFE conic tube	PVPHF	0
35	**1-L2**	NaBF4 (10)	1,2-DCE	PTFE conic tube	PVPHF	0
36	**1-L3**	CsF (10)	1,2-DCE	PTFE conic tube	PVPHF	0
37	**1-L2**	CsF (10)	1,2-DCE	PTFE conic tube	PVPHF	0

[a] NMR yield.

3.3 Model reactions

[$PtCl_2$(dppe)] (**1-L2**) was chosen to be a suitable precatalyst for the following model reactions.

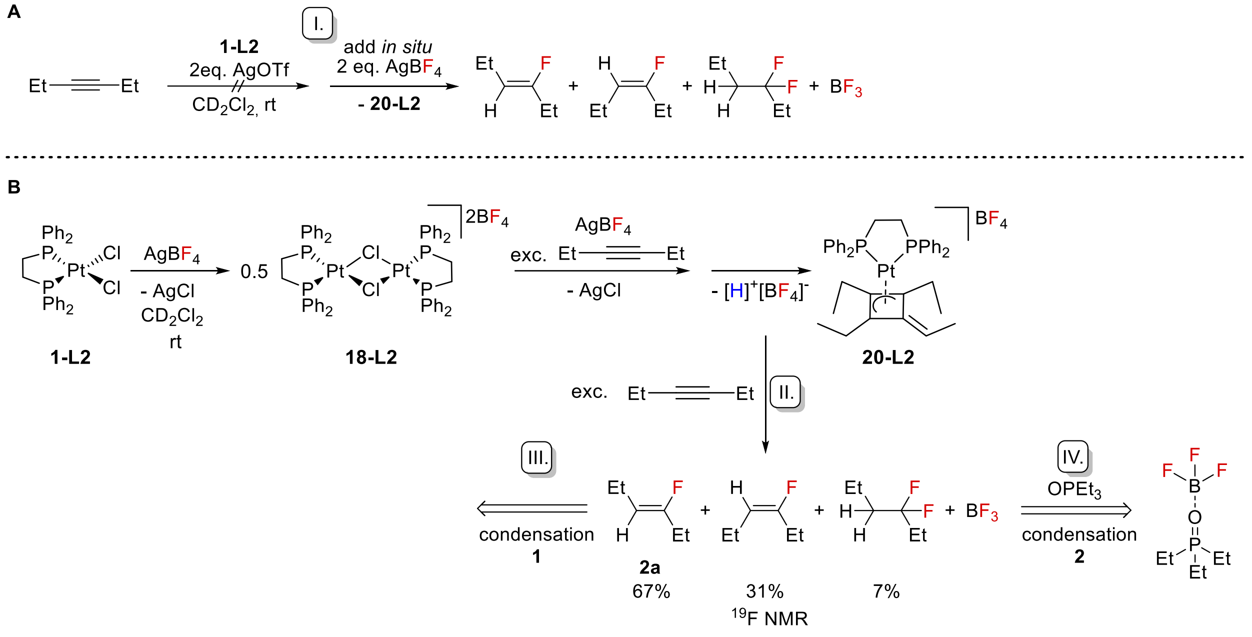

Figure S1. **A** Model reaction I. for the observation of fluorination of the alkyne by BF_4^- anions forming (*Z*)-, (*E*)-3-fluoro-3-hexene and 3,3-difluorohexane. **B** Model reactions II.-IV. for the observation of the formation of **20-L2**, fluoroalkenes, 3,3-difluorohexane and BF_3. The latter was characterised by condensation of the reaction mixture (condensation **1**), which forms the adduct BF_3·$OPEt_3$ in the presence of $OPEt_3$ (condensation **2**).

I. **1-L2** (0.03 mmol, 1 eq.), AgOTf (23 mg, 0.09 mmol, 3 eq.) and 3-hexyne (34 µL 0.3 mmol, 10 eq.) and CD_2Cl_2 (0.5 mL) were added to a J.Young NMR tube. The reaction solution was monitored at room temperature. $^{31}P\{^1H\}$ NMR data revealed the formation of [PtCl(dppe)]$_2$[OTf]$_2$ and [Pt(η^3-(C_2H_5*C*)$_3$*C*=C_2H_4)(dppe)][OTf]. After 24 hours no formation of fluoroalkenes was observed. The reaction solution was filtered into second J.Young NMR tube containing $AgBF_4$ (18 mg, 0.09 mmol, 3eq.). After 2 hours the formation of (*Z*)-, (*E*)-3-fluoro-3-hexene and 3,3-difluoroalkane was observed (Figure S1A, Figure S73).

II. **1-L2** (0.034 mmol, 1 eq.), $AgBF_4$ (27 mg, 0.136 mmol, 4 eq.), 3-hexyne (34 µL 0.3 mmol, 10 eq.), benzotrifluoride (36 µL, 0.3 mmol, 10 eq.) and CD_2Cl_2 (0.5 mL) were added to a J.Young NMR tube. The reaction solution was monitored at room temperature at different time points. $^{31}P\{^1H\}$ NMR data revealed the formation of [PtCl(dppe)]$_2$[BF_4]$_2$ (**18-L2**) and [Pt(η^3-(C_2H_5*C*)$_3$*C*=C_2H_4)(dppe)][BF_4] (**20-L2**) (Figure S78). Over time the signal for **18-L2** diminished, whilst the signals for **20-L2** increased. Once only the signals of **20-L2** were observed in the $^{31}P\{^1H\}$ NMR spectrum, integration of the ^{19}F NMR spectrum was performed using the trifluorotoluene singlet resonance as an internal standard. The formation of (*Z*)-3-fluoro-3-hexene (**2a**) (67%), (*E*)-3-fluoro-3-hexene (31%) and traces of 3,3-difluorohexane (7%) was observed (Figure S1B, Figure S74-S75).

III. Model reaction II. was repeated without the addition of benzotrifluoride. Once only the signals of **20-L2** were observed all volatiles of the J.Young NMR tube were condensed *via* a condensing bridge into a second J.Young NMR tube (Figure S1B, condensation **1**). ^{19}F NMR and ^{11}B NMR spectroscopic data revealed the formation of **2a**, its *trans* isomer, traces of 3,3-difluorohexane as well as BF_3 (Figure S76-S77). The spectroscopic data are in accordance with the literature.[34]

NMR data of BF_3:

^{19}F NMR (282.4 MHz, CD_2Cl_2): δ [ppm] = -126.5 (br s, 3F).

^{11}B NMR (96.3 MHz, CD_2Cl_2): δ [ppm] = 9.3 (br s, 1B).

IV. Model reaction II. was repeated without the addition of benzotrifluoride. Once only the signals of **20-L2** were observed all volatiles of the J.Young NMR tube were condensed *via* a condensing bridge into a second J.Young NMR tube containing $OPEt_3$ (1.1 eq.) (Figure S1B, condensation **2**). ^{19}F, $^{31}P\{^1H\}$ and ^{11}B NMR data revealed the formation of **2a**, its *trans* isomer, traces of 3,3-difluorohexane as well as $BF_3{\cdot}OPEt_3$ (Figure S78-S80). The spectroscopic data are in accordance with the literature.[35]

Selected NMR data of $BF_3{\cdot}OPEt_3$:

^{19}F NMR (282.4 MHz, CD_2Cl_2): δ [ppm] = -146.1 (m, 3F).

^{31}P{^{1}H} NMR (121.5 MHz, CD_2Cl_2): δ [ppm] = 78.6 (q, $^3J_{P,F}$ = 6 Hz, 1P).

^{11}B NMR (96.3 MHz, CD_2Cl_2): δ [ppm] = -1.0 (m, 1B).

3.4 Experiments for the Observation of *β*-Fluorovinyl Platinum Complexes as Intermediates

General procedure for the observation of *β*-fluorovinyl Pt complexes as intermediates of the catalytic reactions: Following the general procedure for the catalytic hydrofluorination of alkynes, the Pt precatalyst (**1-L1-L4**) (0.03 mmol), $AgBF_4$ (23 mg, 0.12 mmol), PVPHF (80 mg) were added to a 8 mL PTFE tube with screw cap. Then, 3-hexyne (34 µL 0.3 mmol) and 1,2-$C_2H_4Cl_2$ (1,2-DCE) (0.4 mL) were added under air. The reaction solution was stirred at room temperature for 2-6 hours and filtered into an NMR tube equipped with a PFA inliner. Subsequent NMR spectroscopic and ESI-MS measurements revealed the formation of the *β*-fluorovinyl Pt intermediates (**21-L1-L4**) formed during the catalysis.

[Pt(EtC=CFEt)(η^2-Et*C*≡*C*Et)(dppm)][BF_4] (**21-L1**)

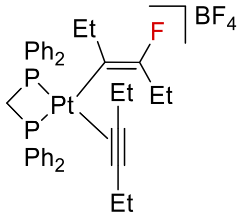

Prepared according to the general procedure using [$PtCl_2$(dppm)] (**1-L1**) as precatalyst. NMR spectroscopy (Figure S67) revealed the formation of [Pt(EtC=CFEt)(η^2-Et*C*≡*C*Et)(dppm)][BF_4] (**21-L1**) besides the formation of [Pt(η^3-(C_2H_5*C*)$_3$*C*=C_2H_4)(dppm)][BF_4].

Selected analytical data:

^{19}F NMR (470.6 MHz, $CDCl_3$): δ [ppm] = -108.8 (m + sat., 1F, C*F*).

^{19}F{^{1}H} NMR (470.6 MHz, $CDCl_3$): δ [ppm] = -108.8 (dd + sat., $^1J_{F,Pt}$ = 146 Hz, $^4J_{F,P}$ = 25 Hz, $^4J_{F,P}$ = 21 Hz, 1F, C*F*).

MS (ESI-MS, positive mode): for [M]$^+$ Calcd.: *m*/*z* = 762.24. Found: *m*/*z* = 762.1.

[Pt(EtC=CFEt)(η^2-Et*C*≡*C*Et)(dppe)][BF_4] (**21-L2**)

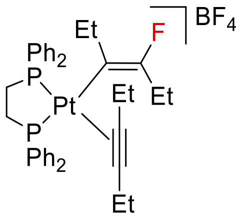

Prepared according to the general procedure using [$PtCl_2$(dppe)] (**1-L2**) as precatalyst. NMR spectroscopy (Figure S68-S69) revealed the formation of [Pt(EtC=CFEt)(η^2-Et*C*≡*C*Et)(dppe)][BF_4] (**21-L2**) besides the formation of [Pt(η^3-(C_2H_5*C*)$_3$*C*=C_2H_4)(dppe)][BF_4] (**20-L2**).

Selected analytical data:

^{31}P{^{1}H} NMR (121.5 MHz, CD_2Cl_2): δ [ppm] = 46.9 (dd + sat., $^1J_{P,Pt}$ = 1727 Hz, $^4J_{P,F}$ = 25 Hz, $^2J_{P,P}$ = 3 Hz, 1P, P *trans* to fluorovinyl ligand), 43.4 (dd + sat., $^1J_{P,Pt}$ = 4027 Hz, $^4J_{P,F}$ = 9 Hz, $^2J_{P,P}$ = 3 Hz, 1P, P *trans* to alkyne ligand).

^{19}F NMR (470.6 MHz, CD_2Cl_2): δ [ppm] = -109.6 (m + sat., 1F, C*F*).

^{19}F{^{1}H} NMR (470.6 MHz, CD_2Cl_2): δ [ppm] = -109.6 (dd + sat., $^3J_{F,Pt}$ = 108 Hz, $^4J_{F,P}$ = 25 Hz, $^4J_{F,P}$ = 9 Hz, 1F, C*F*).

MS (ESI-MS, positive mode): for [M] Calcd.: *m*/*z* = 776.26. Found: *m*/*z* = 776.5.

[Pt(EtC=CFEt)(η^2-Et*C*≡*C*Et)(PCNP)][BF_4] (**21-L3**)

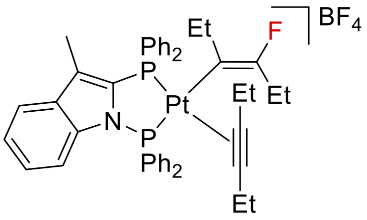

Prepared according to the general procedure using [$PtCl_2$(PCNP)] (**1-L3**) as precatalyst. NMR spectroscopy (Figure S70-S71) revealed the formation of both isomers of [Pt(EtC=CFEt)(η^2-Et*C*≡*C*Et)(PCNP)][BF_4] (**21-L3**) with the vinyl ligand in the *trans*-position to the different phosphorus atoms in a ratio of 1:1 besides the formation of both isomers of [Pt(η^3-(C_2H_5*C*)$_3$*C*=C_2H_4)(PCNP)][BF_4].

Selected analytical data:

^{31}P{^{1}H} NMR (202.5 MHz, CD_2Cl_2): δ [ppm] = 78.1 (dd + sat., $^1J_{P,Pt}$ = 1886 Hz, $^4J_{P,F}$ = 28 Hz, $^2J_{P,P}$ = 18 Hz, 1P, N*P*), 12.5 (dd + sat., $^1J_{P,Pt}$ = 3890 Hz, $^4J_{P,P}$ = 18 Hz, $^2J_{P,F}$ = 10 Hz, 1P, C*P*).

^{19}F NMR (470.6 MHz, CD_2Cl_2): δ [ppm] = -108.2 (m + sat., 1F, C*F*).

^{19}F{^{1}H} NMR (470.6 MHz, CD_2Cl_2): δ [ppm] = -108.2 (dd + sat., $^1J_{F,Pt}$ = 103 Hz, $^4J_{F,P}$ = 28 Hz, $^4J_{F,P}$ = 10 Hz, 1F, C*F*).

MS (ESI-MS, positive mode): for [M] Calcd.: *m*/*z* = 877.28. Found: *m*/*z* = 877.1.

Isomer:

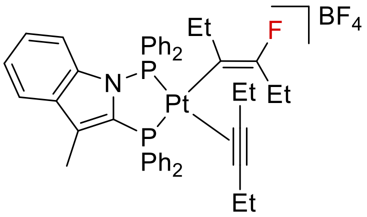

^{31}P{^{1}H} NMR (202.5 MHz, CD_2Cl_2): δ [ppm] = 68.8 (dd + sat., $^1J_{P,Pt}$ = 4386 Hz, $^2J_{P,P}$ = 18 Hz, $^4J_{P,F}$ = 10 Hz, 1P, N*P*), 14.1 (dd + sat., $^1J_{P,Pt}$ = 1686 Hz, $^4J_{P,F}$ = 29 Hz, $^2J_{P,P}$ = 18 Hz, 1P, C*P*).

^{19}F NMR (470.6 MHz, CD_2Cl_2): δ [ppm] = -108.5 (m + sat., 1F, C*F*).

^{19}F{^{1}H} NMR (470.6 MHz, CD_2Cl_2): δ [ppm] = -108.5 (dd + sat., $^1J_{F,Pt}$ = 95 Hz, $^4J_{F,P}$ = 29 Hz, $^4J_{F,P}$ = 10 Hz, 1F, C*F*).

[Pt(EtC=CFEt)(η^2-Et*C*≡*C*Et)(dppp)][BF_4] (**21-L4**)

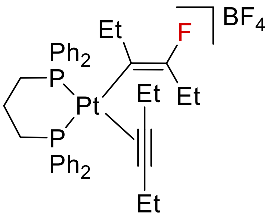

Prepared according to the general procedure using [$PtCl_2$(dppp)] (**1-L4**) as precatalyst. NMR spectroscopy (Figure S72) revealed the formation of [Pt(EtC=CFEt)(η^2-Et*C*≡*C*Et)(dppp)][BF_4] (**21-L4**) besides the formation of [Pt(η^3-(C_2H_5*C*)$_3$*C*=C_2H_4)(dppp)][BF_4].

Selected analytical data:

^{19}F NMR (282.4 MHz, CD_2Cl_2): δ [ppm] = -106.8 (m + sat., 1F, C*F*).

^{19}F{^{1}H} NMR (282.4 MHz, $CDCl_3$): δ [ppm] = -106.8 (dd + sat., $^1J_{F,Pt}$ = 119 Hz, $^4J_{F,P}$ = 23 Hz, $^4J_{F,P}$ = 9 Hz, 1F, C*F*).

MS (ESI-MS, positive mode): for [M]$^+$ Calcd.: *m*/*z* = 790.27. Found: *m*/*z* = 790.1.

3.5 Monitoring of the catalytic reactions by NMR spectroscopy

For monitoring experiments, the Pt complex (0.03 mmol), $AgBF_4$ (23 mg, 0.12 mmol), PVPHF (100 mg) were added to a 8 mL PTFE tube with screw cap. Then, 3-hexyne (34 µL 0.3 mmol), benzotrifluoride (36 µL, 0.3 mmol) as internal standard and 1,2-$C_2H_4Cl_2$ (1,2-DCE) (0.4 mL) were added under air. The reaction solution was stirred at room temperature and filtered into an NMR tube equipped with a PFA inliner at different time points.

For the monitoring of the Pt catalyst $^{31}P\{^1H\}$ NMR spectroscopy was performed using an acquisition time of aq = 1 s. The formation of the intermediate [Pt(EtC=CFEt)(η^2-Et*C*≡*C*Et)(dppe)][BF_4] (**21-L2**) and of the off-cycle complex [Pt(η^3-(C_2H_5*C*)$_3$*C*=C_2H_4)(dppe)][BF_4] (**20-L2**) was monitored and their sum of integrals in the $^{31}P\{^1H\}$ NMR spectrum was hypothesised to be constant *in situ*. Integration of both complexes towards each other resulted in the calculated amount of each complex.

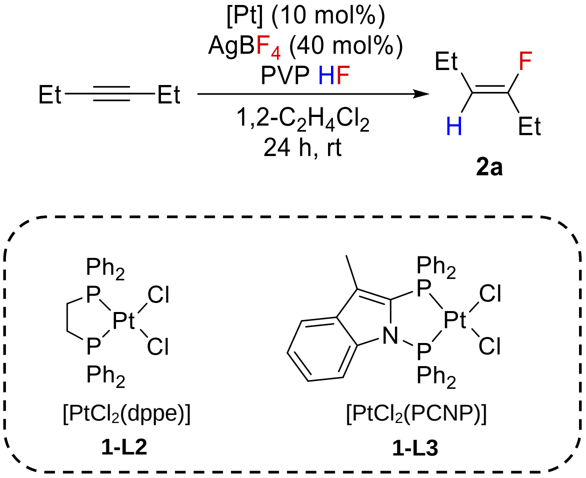

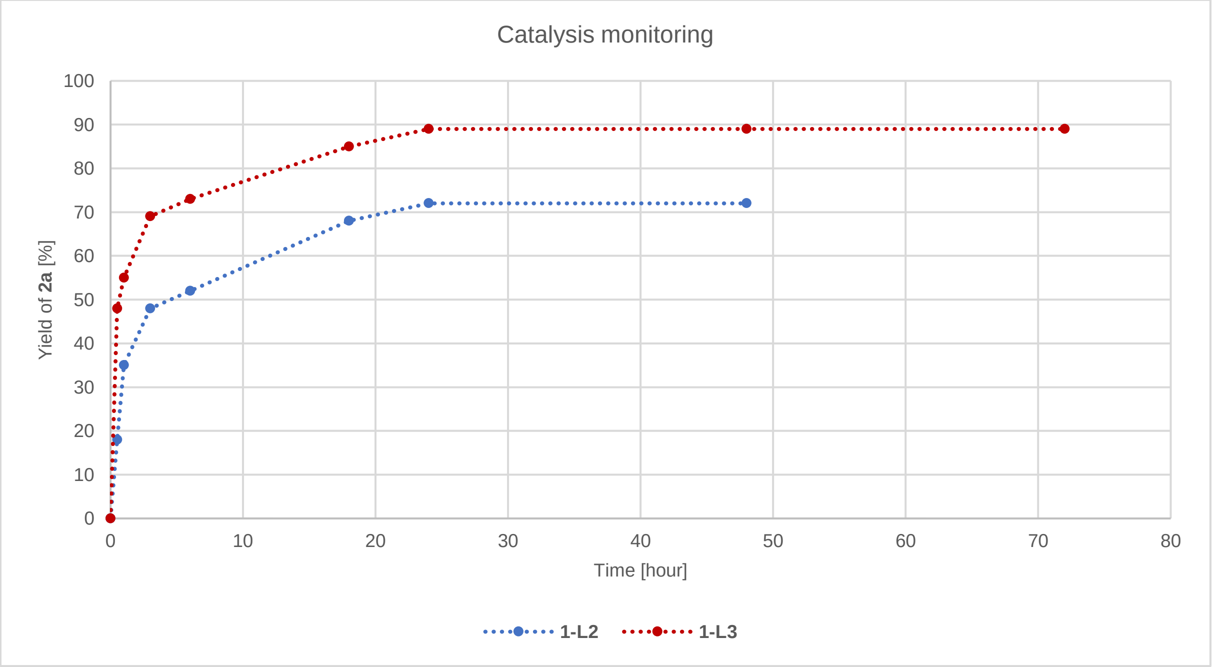

Figure S2. Reaction monitoring of catalytic hydrofluorination of 3-hexyne giving (*Z*)-3-fluoro-3-hexene (**2a**) over time using the precatalysts [$PtCl_2$(dppe)] (**1-L2**) (blue) and [$PtCl_2$(PCNP)] (**1-L3**) (red).

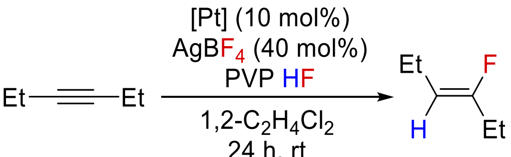

2a

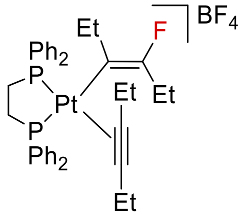

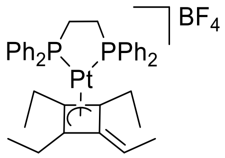

[Pt(EtC=CFEt)(η^2-Et*C*≡*C*Et)(dppe)][BF_4]
21-L2

[Pt(η^3-$(C_2H_5C)_3C=C_2H_4$)(dppe)][BF_4]
20-L2

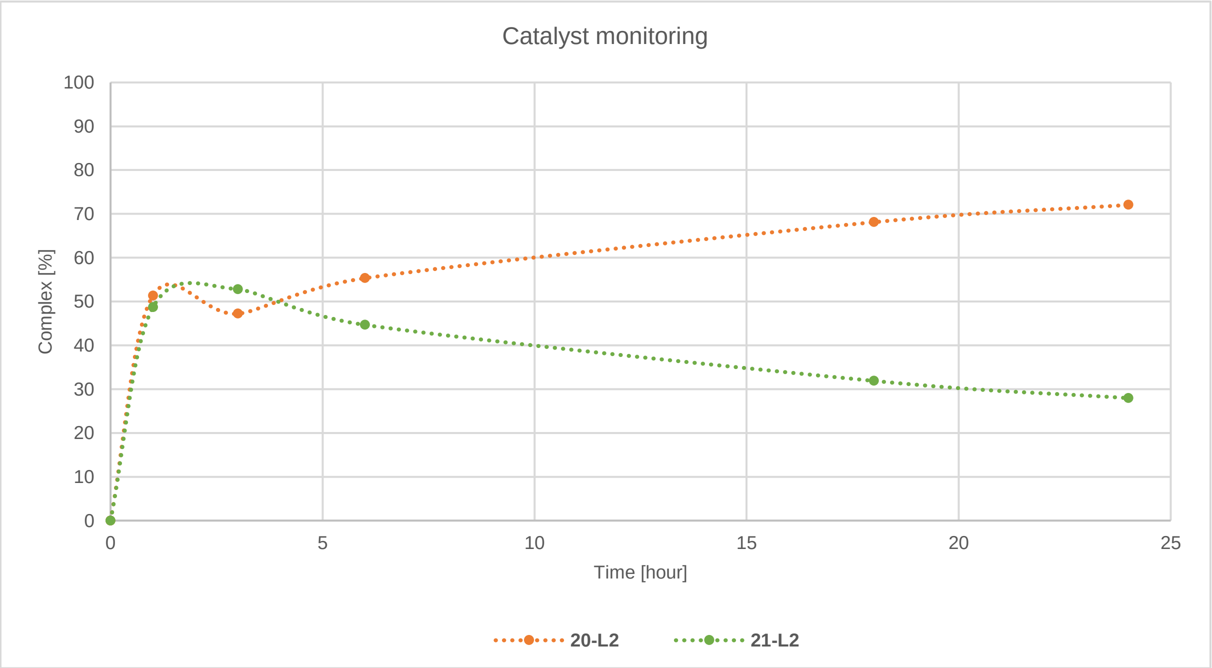

Figure S3. Catalyst monitoring of the catalytic hydrofluorination of 3-hexyne using the precatalyst [$PtCl_2$(dppe)] (**1-L2**) showing the amount of the intermediate [Pt(EtC=CFEt)(η^2-Et*C*≡*C*Et)(dppe)][BF_4] (**21-L2**) (green dashed line) and off-cycle complex [Pt(η^3-$(C_2H_5C)_3C=C_2H_4$)(dppe)][BF_4] (**20-L2**) (orange dashed line).

4 Crystal data and structure refinement

Crystal data and structure refinement for PCNP (**L3**)

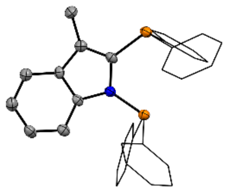

Empirical formula	$C_{33} H_{27} N P_2$
Formula weight	499.49
Temperature	100(2) K
Wavelength	0.71073 Å
Crystal system	Monoclinic
Space group	$P2_1/c$
Unit cell dimensions	a = 9.9700(10) Å<br>b = 18.0907(19) Å β = 107.676(4) °<br>c = 15.0229(17) Å
Volume	2581.7(5) $Å^3$
Z	4
Density (calculated)	1.285 Mg/m^3
Absorption coefficient	0.191 mm^{-1}
F(000)	1048
Crystal size	0.512 x 0.056 x 0.015 mm^3
Theta range for data collection	1.814 ° to 25.023 °
Index ranges	-11<=h<=11, -21<=k<=21, -17<=l<=17
Reflections collected	114381
Independent reflections	4542 [R(int) = 0.1290]
Completeness to theta = 25.023 °	99.9%
Absorption correction	Multi-scan
Max. and min. transmission	0.7452 and 0.6201
Refinement method	Full-matrix least-squares on F^2
Data / restraints / parameters	4542 / 0 / 326
Goodness-of-fit on F2	1.158
Final R indices [I>2sigma(I)]	R1 = 0.0778, wR2 = 0.2211
R indices (all data)	R1 = 0.0900, wR2 = 0.2297
Largest diff. peak and hole	1.163 and -0.390 e.$Å^{-3}$
CCDC	2429317

Crystal data and structure refinement for [$PtCl_2$(PCNP)] (**1-L3**).

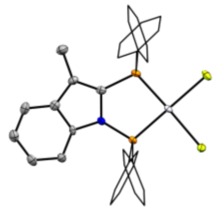

Empirical formula	$C_{33} H_{27} Cl_2 N P_2 Pt$
Formula weight	765.48
Temperature	100(2) K
Wavelength	0.71073 Å
Crystal system	Monoclinic
Space group	$P2_1/n$
Unit cell dimensions	a = 8.8561(4) Å<br>b = 20.2802(9) Å β = 96.186(2) °<br>c = 16.3668(8) Å
Volume	2922.4(2) $Å^3$
Z	4
Density (calculated)	1.740 Mg/m^3
Absorption coefficient	5.118 mm^{-1}
F(000)	1496
Crystal size	0.117 x 0.044 x 0.016 mm^3
Theta range for data collection	2.367 ° to 28.327 °
Index ranges	-11<=h<=11, -27<=k<=27, -21<=l<=21
Reflections collected	71350
Independent reflections	7273 [R(int) = 0.0869]
Completeness to theta = 25.242 °	100%
Absorption correction	Multi-scan
Max. and min. transmission	0.5633 and 0.4622
Refinement method	Full-matrix least-squares on F^2
Data / restraints / parameters	7273 / 0 / 356
Goodness-of-fit on F2	1.043
Final R indices [I>2sigma(I)]	R1 = 0.0313, wR2 = 0.0551
R indices (all data)	R1 = 0.0479, wR2 = 0.0620
Largest diff. peak and hole	1.002 and -1.259 e.$Å^{-3}$
CCDC	2429320

Crystal data and structure refinement for [Pt(Me)$_2$(PCNP)].

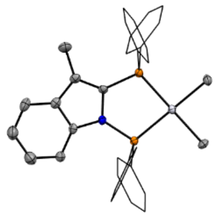

Empirical formula	$C_{35} H_{33} N P_2 Pt$	
Formula weight	724.65	
Temperature	100(2) K	
Wavelength	0.71073 Å	
Crystal system	Monoclinic	
Space group	*P*2$_1$/*n*	
Unit cell dimensions	a = 8.8825(6) Å	
	b = 20.4195(14) Å	β = 95.857(2) °
	c = 16.4957(10) Å	
Volume	2976.3(3) Å^3	
Z	4	
Density (calculated)	1.617 Mg/m^3	
Absorption coefficient	4.847 mm^{-1}	
F(000)	1432	
Crystal size	0.119 x 0.013 x 0.002 mm^3	
Theta range for data collection	1.995 ° to 25.113 °	
Index ranges	-10<=h<=10, -24<=k<=24, -19<=l<=19	
Reflections collected	116975	
Independent reflections	5303 [R(int) = 0.0800]	
Completeness to theta = 25.113 °	99.6%	
Absorption correction	Multi-scan	
Max. and min. transmission	0.4899 and 0.3933	
Refinement method	Full-matrix least-squares on F^2	
Data / restraints / parameters	5303 / 0 / 355	
Goodness-of-fit on F2	1.132	
Final R indices [I>2sigma(I)]	R1 = 0.0244, wR2 = 0.0420	
R indices (all data)	R1 = 0.0288, wR2 = 0.0431	
Largest diff. peak and hole	0.626 and -1.596 e.Å^{-3}	
CCDC	2429319	

Crystal data and structure refinement for *cis*-$[PtCl(Xantphos)]_2[BF_4]_2 \cdot 6CH_2Cl_2$ (**18-L7**$\cdot 6CH_2Cl_2$).

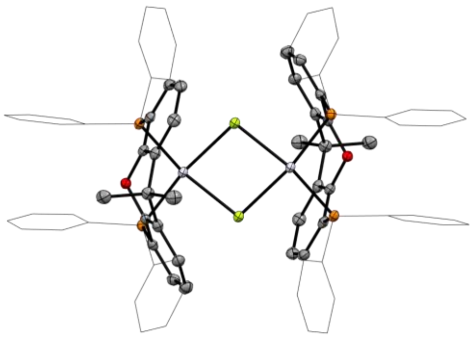

Empirical formula	$C_{84} H_{76} B_2 F_8 Cl_{14} O_2 P_4 Pt_2$	
Formula weight	2301.42	
Temperature	100(2) K	
Wavelength	0.71073 Å	
Crystal system	Triclinic	
Space group	$P\bar{1}$	
Unit cell dimensions	a = 12.8252(12) Å	α =107.760(3) °
	b = 14.5593(12) Å	β = 113.962(3) °
	c = 14.9278(12) Å	γ = 101.538(3) °
Volume	2251.5(3) $Å^3$	
Z	1	
Density (calculated)	1.697 Mg/m^3	
Absorption coefficient	3.625 mm^{-1}	
F(000)	1132	
Crystal size	0.094 x 0.084 x 0.034 mm^3	
Theta range for data collection	2.365 ° to 26.453 °	
Index ranges	-16<=h<=16, -18<=k<=18, -18<=l<=18	
Reflections collected	112606	
Independent reflections	9253 [R(int) = 0.0701]	
Completeness to theta = 25.242 °	99.9%	
Absorption correction	Multi-scan	
Max. and min. transmission	0.4908 and 0.4401	
Refinement method	Full-matrix least-squares on F^2	
Data / restraints / parameters	9253 / 15 / 535	
Goodness-of-fit on F2	1.043	
Final R indices [I>2sigma(I)]	R1 = 0.0247, wR2 = 0.0564	
R indices (all data)	R1 = 0.0279, wR2 = 0.0583	
Largest diff. peak and hole	1.548 and -1.430 e.$Å^{-3}$	
CCDC	2429315	

Crystal data and structure refinement for [Pt(η^3-(C_2H_5*C*)$_3$*C*=C_2H_4)(dppe)][BF_4] (**20-L2**).

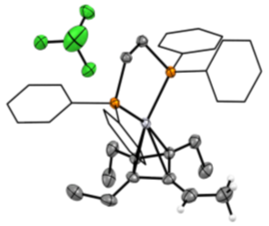

Empirical formula	C_{38} H_{43} B F_4 P_2 Pt
Formula weight	843.56
Temperature	100(2) K
Wavelength	0.71073 Å
Crystal system	Orthorombic
Space group	*Pbca*
Unit cell dimensions	a = 16.9603(19) Å<br>b = 19.318(2) Å<br>c = 21.230(2) Å
Volume	6955.8(14) Å^3
Z	8
Density (calculated)	1.611 Mg/m^3
Absorption coefficient	4.175 mm^{-1}
F(000)	3360
Crystal size	0.101 x 0.043 x 0.017 mm^3
Theta range for data collection	1.864 ° to 25.053 °
Index ranges	-20<=h<=20, -22<=k<=23, -25<=l<=25
Reflections collected	187599
Independent reflections	6159 [R(int) = 0.0828]
Completeness to theta = 25.053 °	100%
Absorption correction	Multi-scan
Max. and min. transmission	0.4899 and 0.4169
Refinement method	Full-matrix least-squares on F^2
Data / restraints / parameters	6159 / 0 / 419
Goodness-of-fit on F2	1.055
Final R indices [I>2sigma(I)]	R1 = 0.0280, wR2 = 0.0524
R indices (all data)	R1 = 0.0386, wR2 = 0.0566
Largest diff. peak and hole	1.189 and -1.090 e.Å^{-3}
CCDC	2429316

Crystal data and structure refinement for [Pt(EtC=CFEt)(κ^3-Xantphos)][BF_4]·CH_2Cl_2 (**22**·CH_2Cl_2).

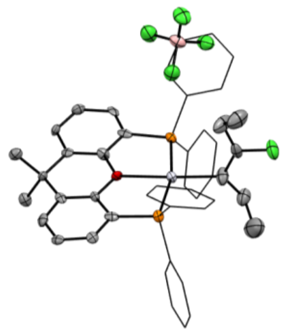

Empirical formula	C_{46} H_{44} B Cl_2 F_5 O P_2 Pt
Formula weight	1046.55
Temperature	100(2) K
Wavelength	0.71073 Å
Crystal system	Monoclinic
Space group	$P2_1$
Unit cell dimensions	a = 11.1046(6) Å<br>b = 15.2205(8) Å β = 107.555(2) °<br>c = 13.4293(7) Å
Volume	2164.1(2) Å^3
Z	2
Density (calculated)	1.606 Mg/m^3
Absorption coefficient	3.496 mm^{-1}
F(000)	1040
Crystal size	0.104 x 0.068 x 0.005 mm^3
Theta range for data collection	2.079 ° to 25.342 °
Index ranges	-13<=h<=13, -18<=k<=18, -16<=l<=16
Reflections collected	68795
Independent reflections	7927 [R(int) = 0.0893]
Completeness to theta = 25.242 °	99.9%
Absorption correction	Multi-scan
Max. and min. transmission	0.4920 and 0.3835
Refinement method	Full-matrix least-squares on F^2
Data / restraints / parameters	7927 / 19 / 529
Goodness-of-fit on F2	1.062
Final R indices [I>2sigma(I)]	R1 = 0.0346, wR2 = 0.0800
R indices (all data)	R1 = 0.0403, wR2 = 0.0827
Largest diff. peak and hole	1.253 and -1.097 e.Å^{-3}
CCDC	2429318

7 NMR Spectra

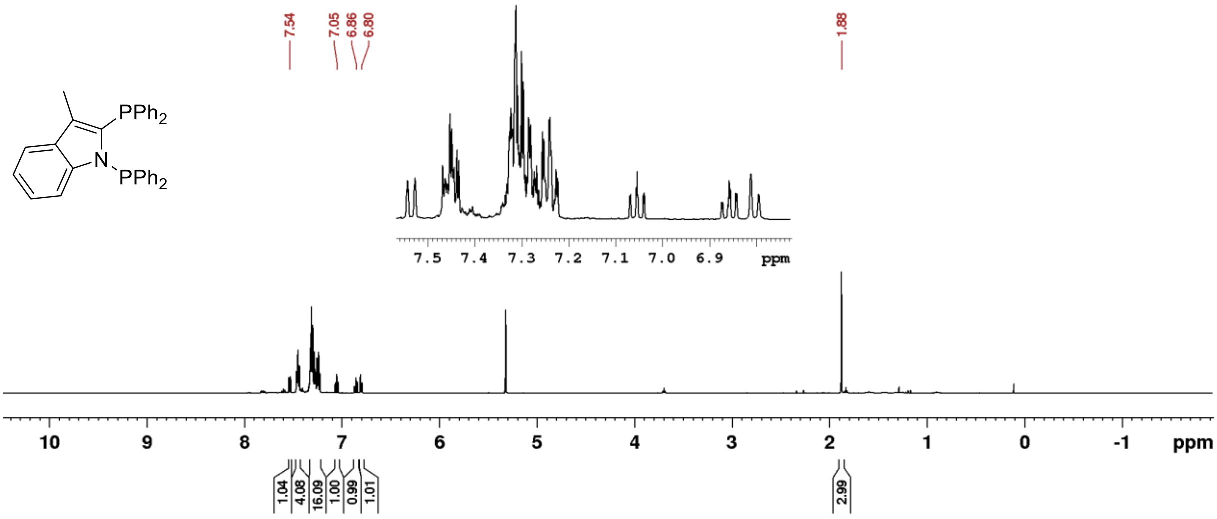

Figure S4. ^{1}H NMR spectrum of PCNP (**L3**).

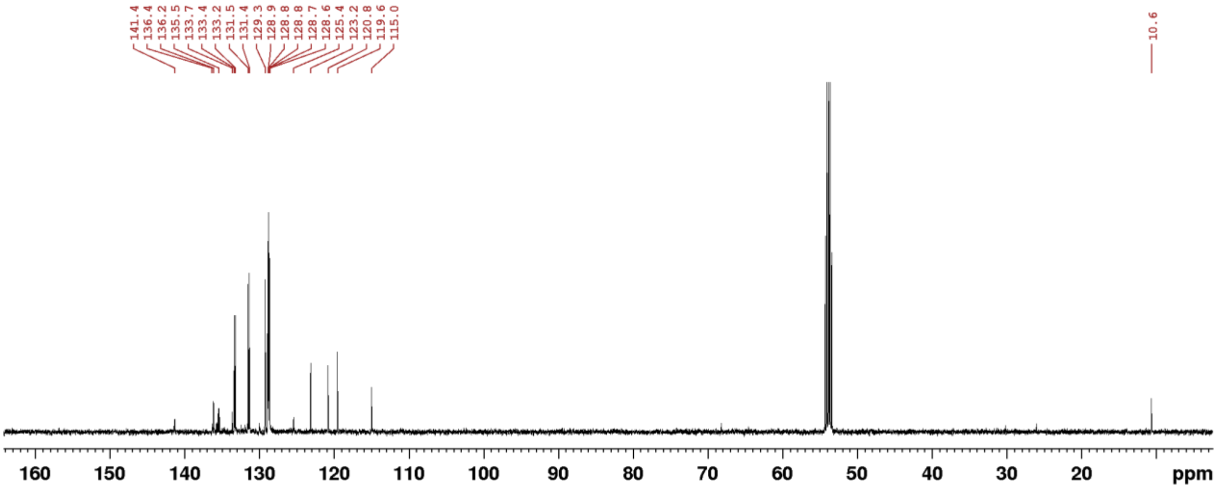

Figure S5. $^{13}C\{^{1}H\}$ NMR spectrum of PCNP (**L3**).

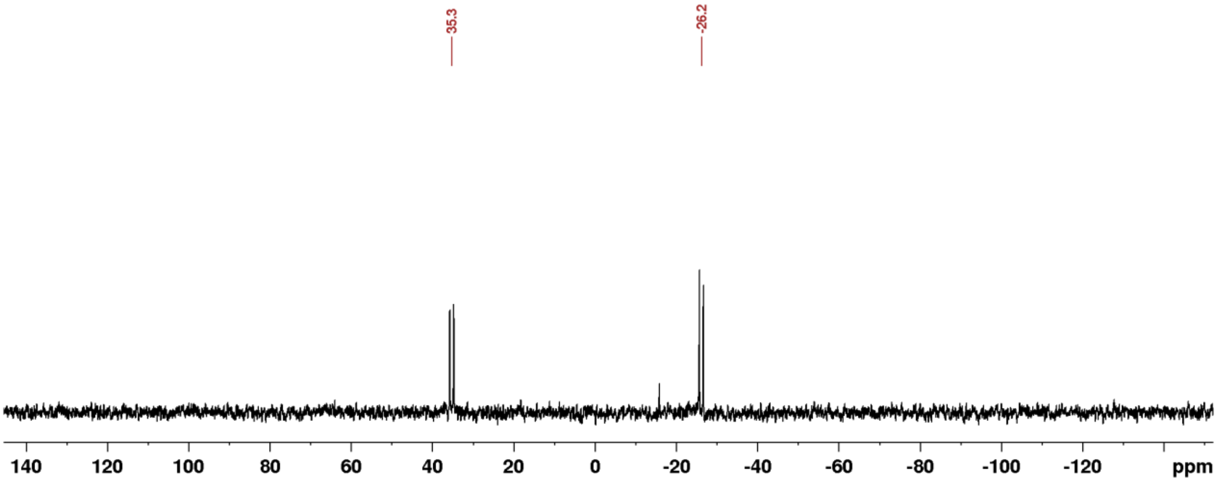

Figure S6. $^{31}P\{^{1}H\}$ NMR spectrum of PCNP (**L3**).

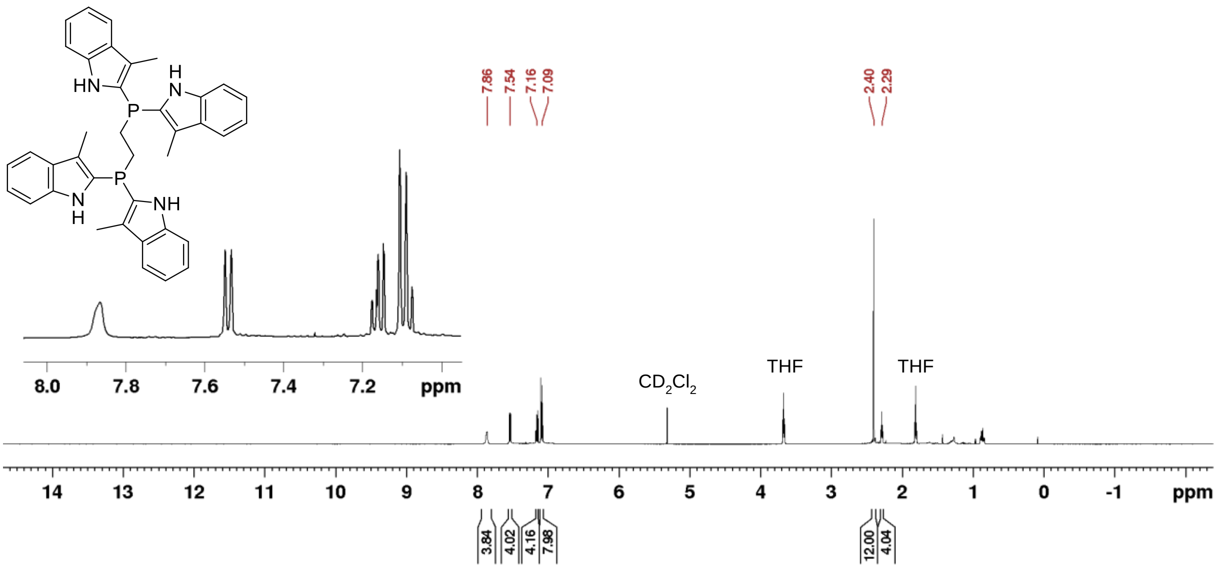

Figure S7. ^{1}H NMR spectrum of d(Ind)pe.

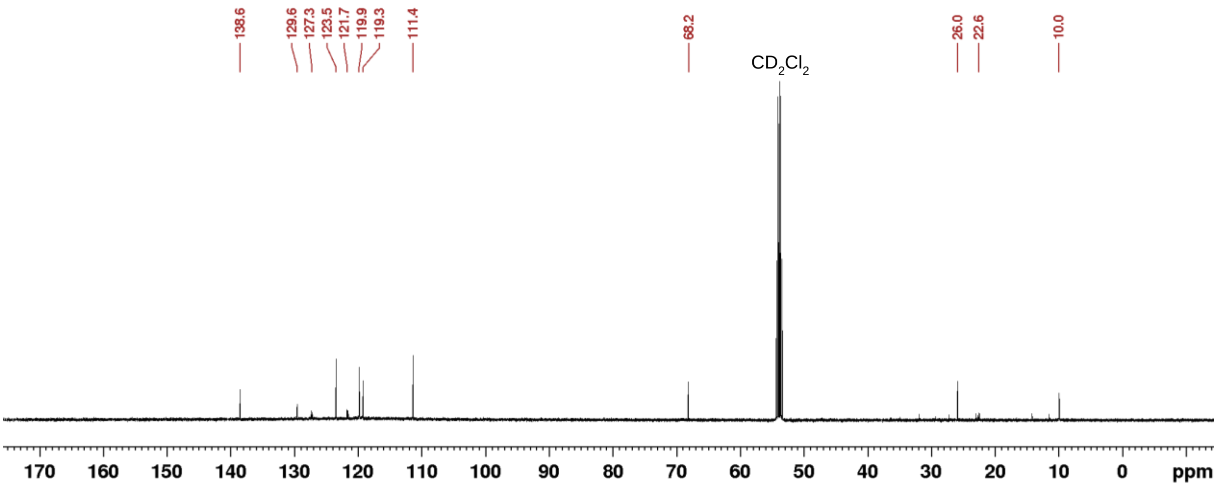

Figure S8. $^{13}C\{^{1}H\}$ NMR spectrum of d(Ind)pe.

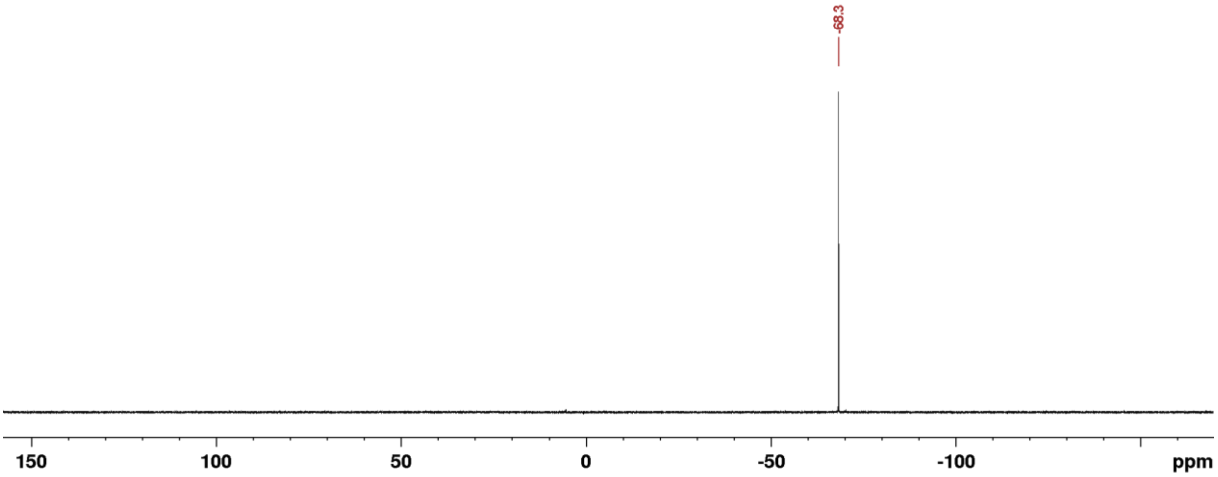

Figure S9. $^{31}P\{^{1}H\}$ NMR spectrum of d(Ind)pe.

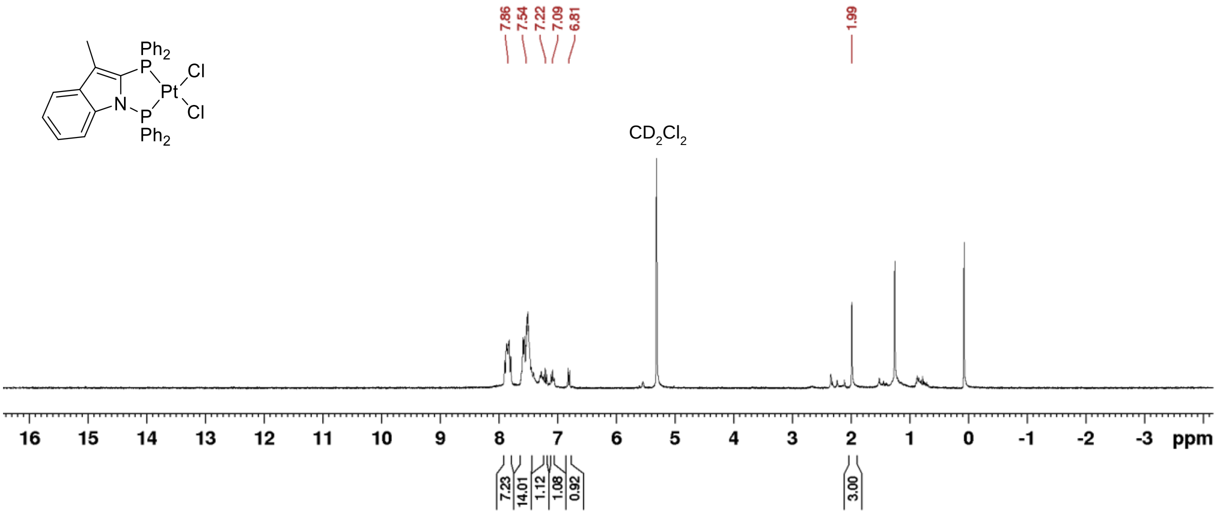

Figure S10. ^{1}H NMR spectrum of **1-L3**.

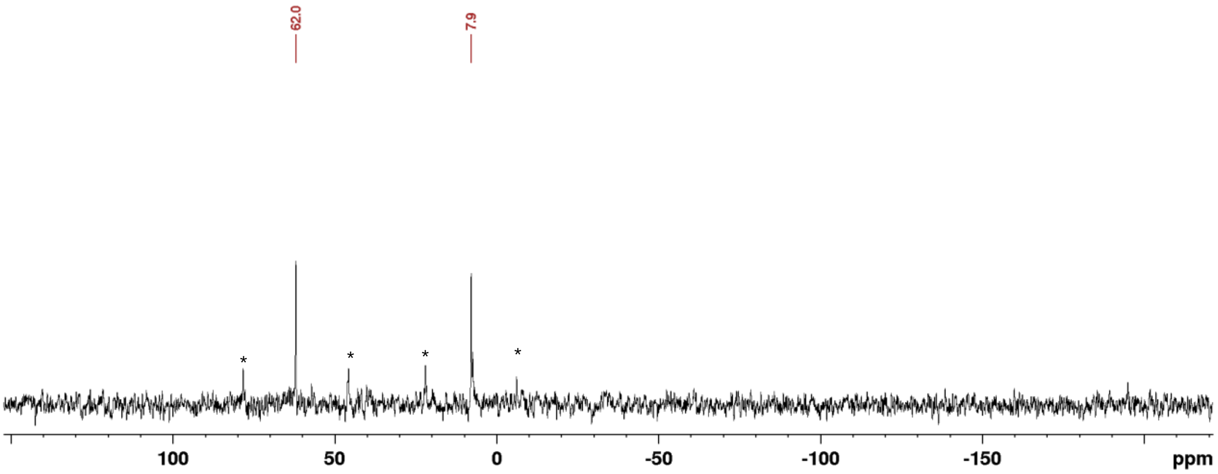

Figure S11. $^{31}P\{^{1}H\}$ NMR spectrum of **1-L3**. * depict ^{195}Pt satellites.

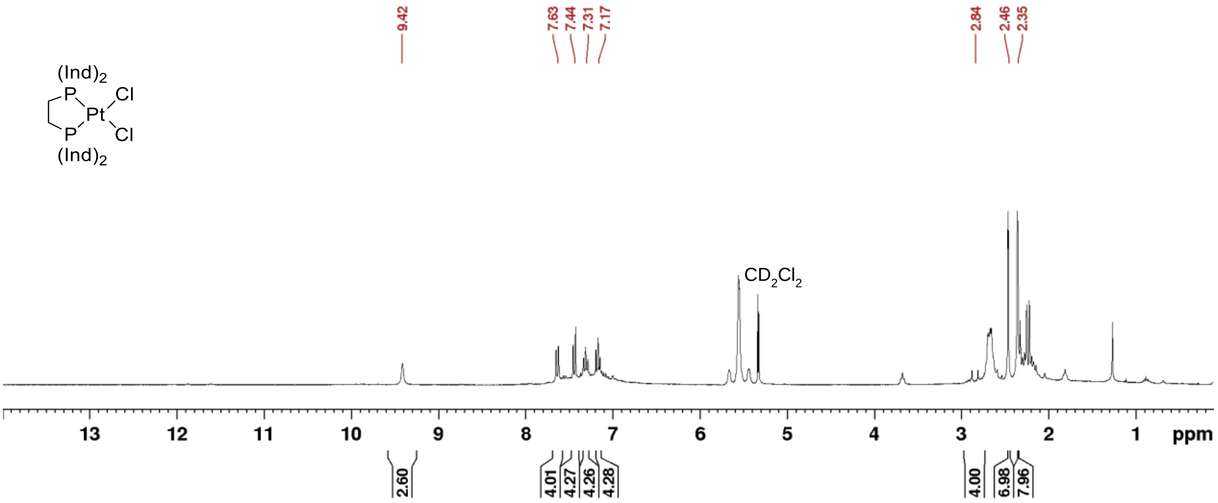

Figure S12. ^{1}H NMR spectrum of $[PtCl_2(d(Ind)pe)]$.

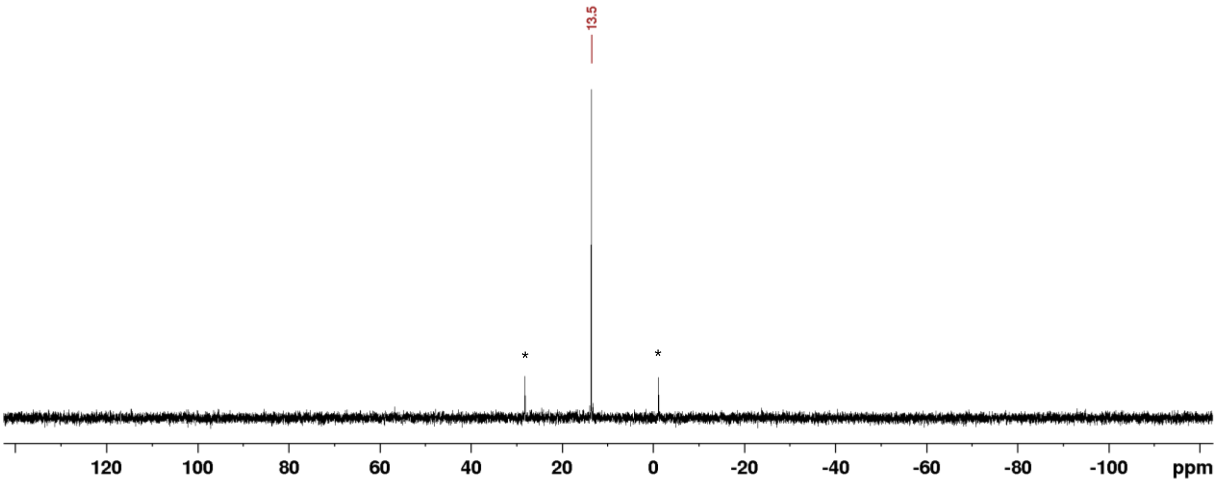

Figure S13. $^{31}P\{^{1}H\}$ NMR spectrum of $[PtCl_2(d(Ind)pe)]$. * depict ^{195}Pt satellites.

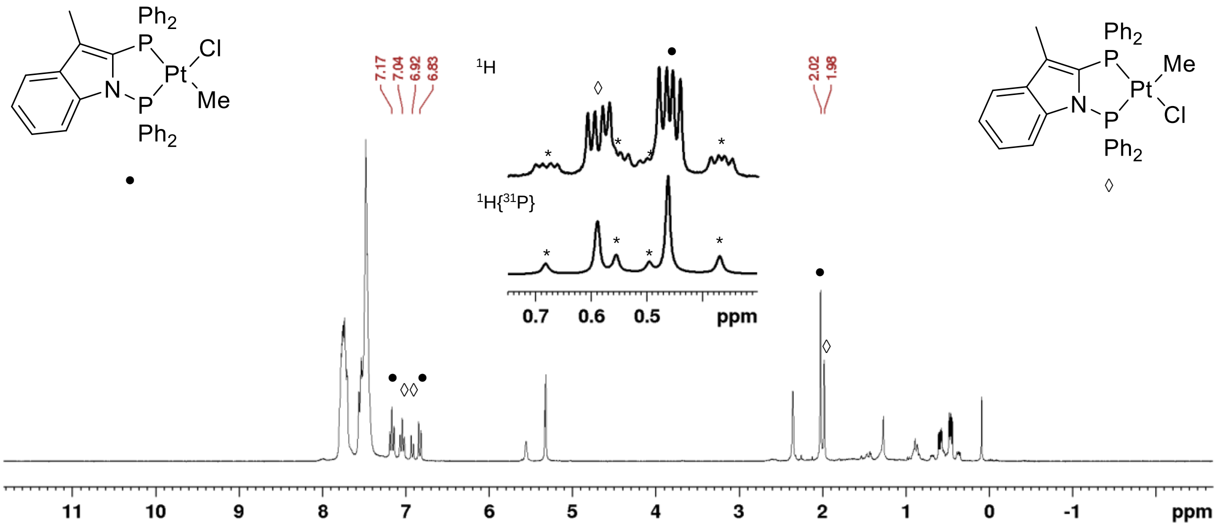

Figure S14. ^{1}H NMR spectrum of a mixture of isomers of [PtMeCl(PCNP)]. * depict ^{195}Pt satellites.

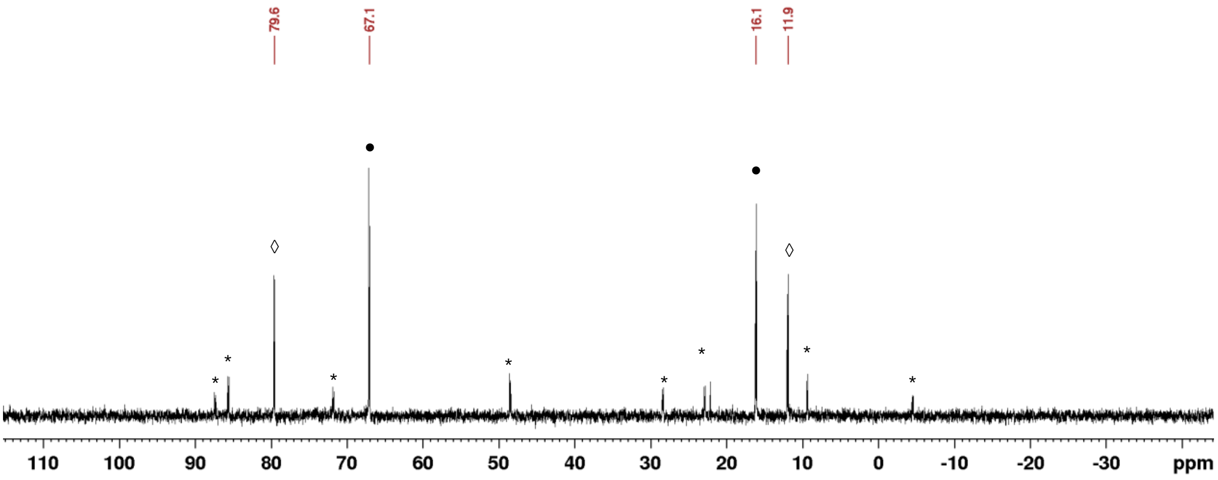

Figure S15. $^{31}P\{^{1}H\}$ NMR spectrum of a mixture of isomers of [PtMeCl(PCNP)]. * depict ^{195}Pt satellites.

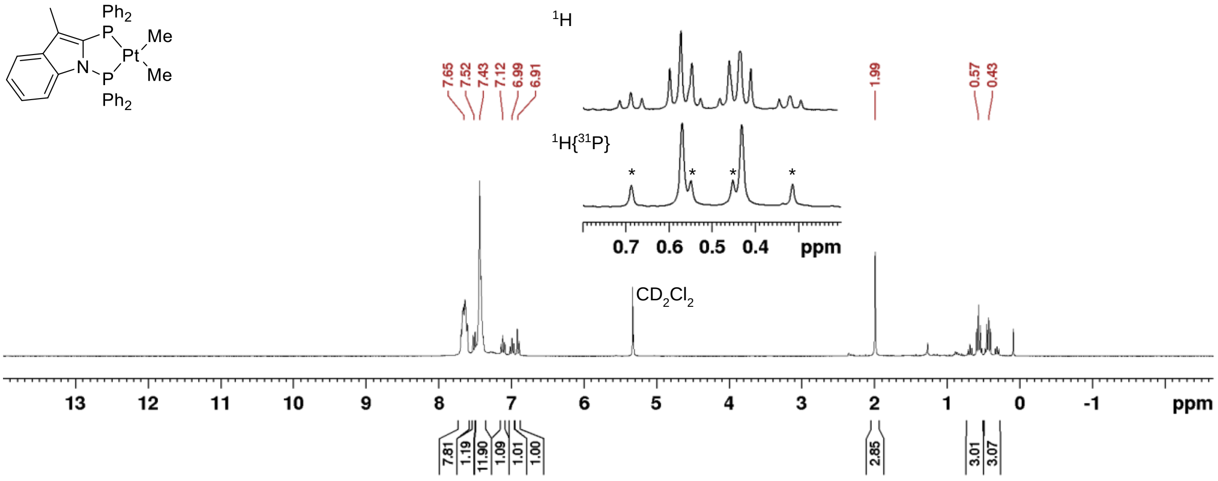

Figure S16. ^{1}H NMR spectrum of a mixture of isomers of [$PtMe_2$(PCNP)]. * depict ^{195}Pt satellites.

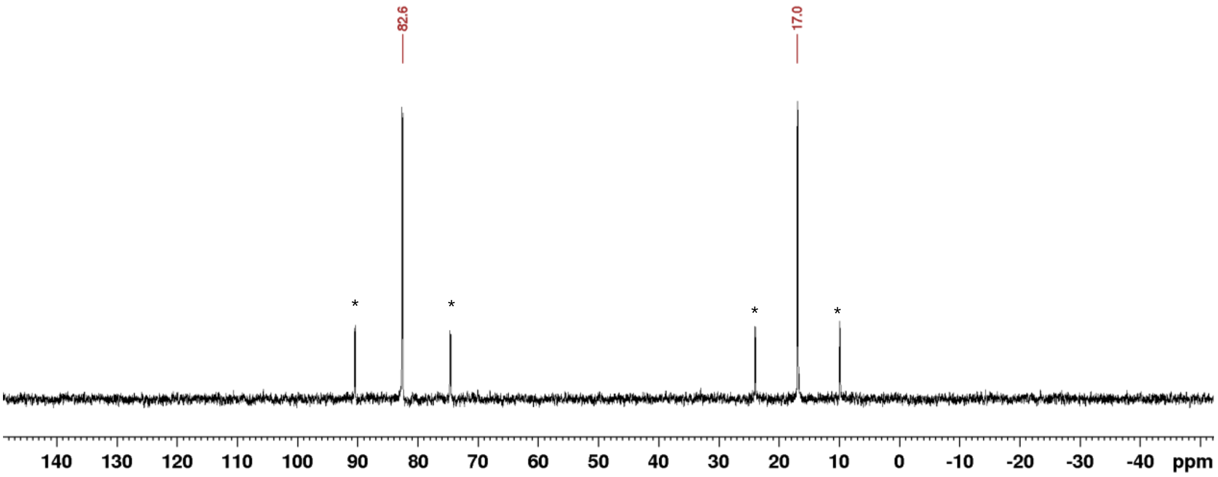

Figure S17. $^{31}P\{^{1}H\}$ NMR spectrum of [$PtMe_2$(PCNP)]. * depict ^{195}Pt satellites.

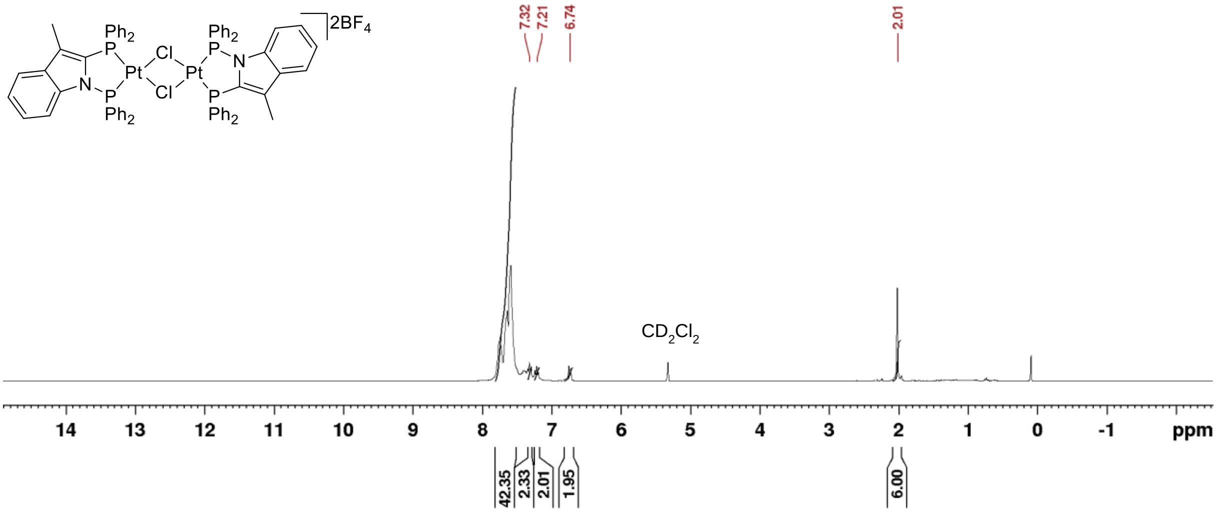

Figure S18. ^{1}H NMR spectrum of $[PtCl(PCNP)]_2[BF_4]_2$ (**18-L3**).

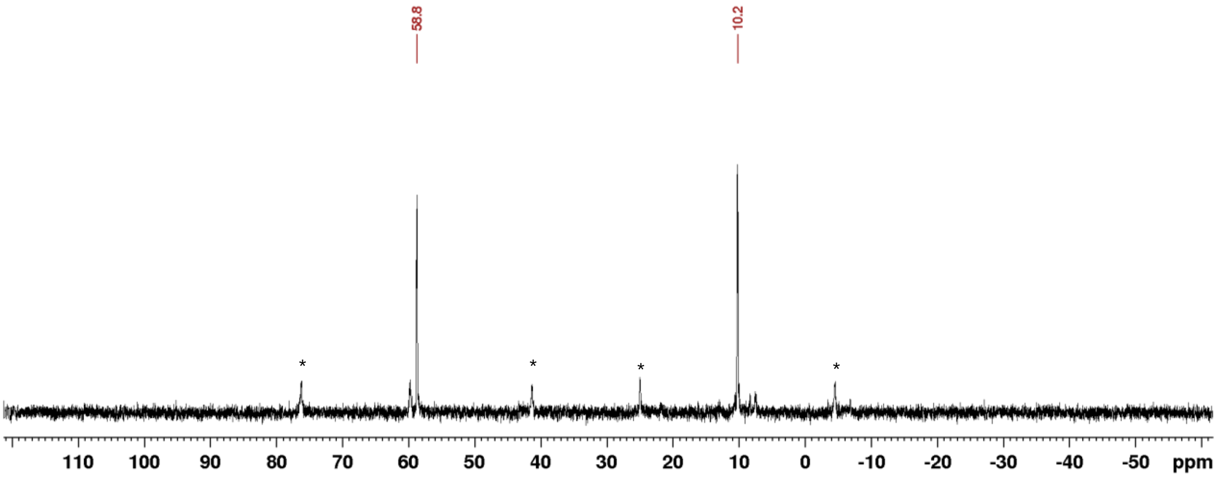

Figure S19. $^{31}P\{^{1}H\}$ NMR spectrum of $[PtCl(PCNP)]_2[BF_4]_2$ (**18-L3**). * depict ^{195}Pt satellites.

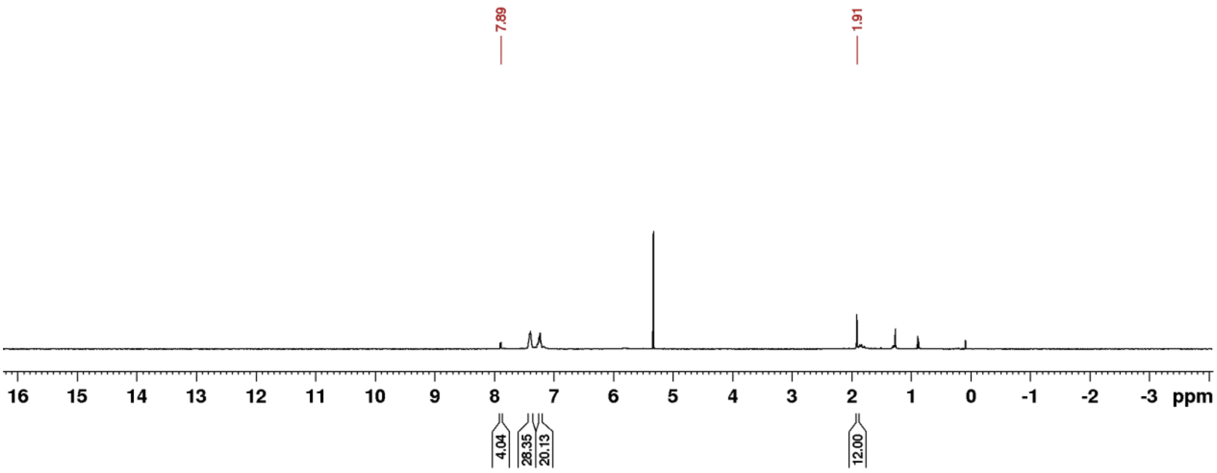

Figure S20. ^{1}H NMR spectrum of $[PtCl(Xantphos)]_2[BF_4]_2$ (**18-L7**).

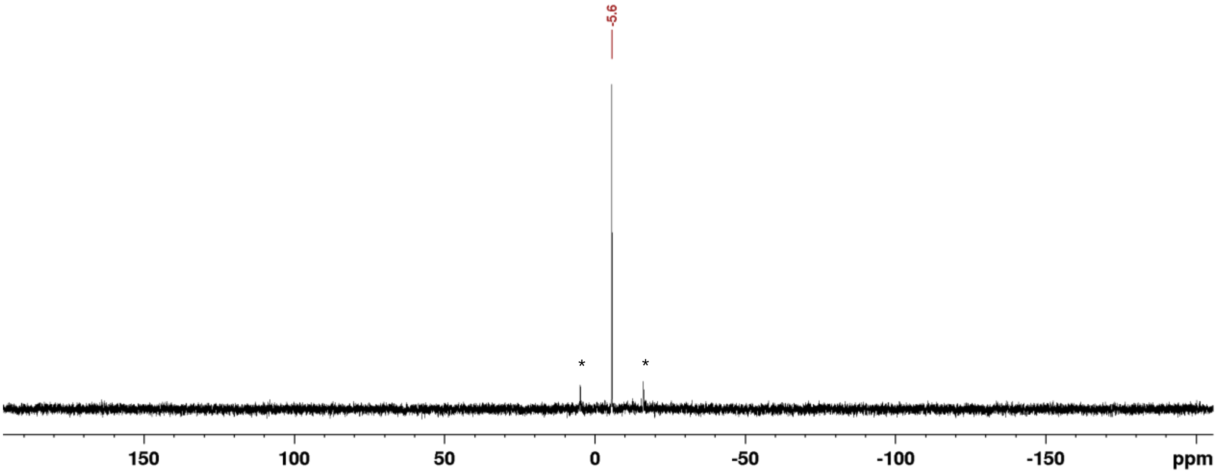

Figure S21. $^{31}P\{^{1}H\}$ NMR spectrum of $[PtCl(Xantphos)]_2[BF_4]_2$ (**18-L7**). * depict ^{195}Pt satellites.

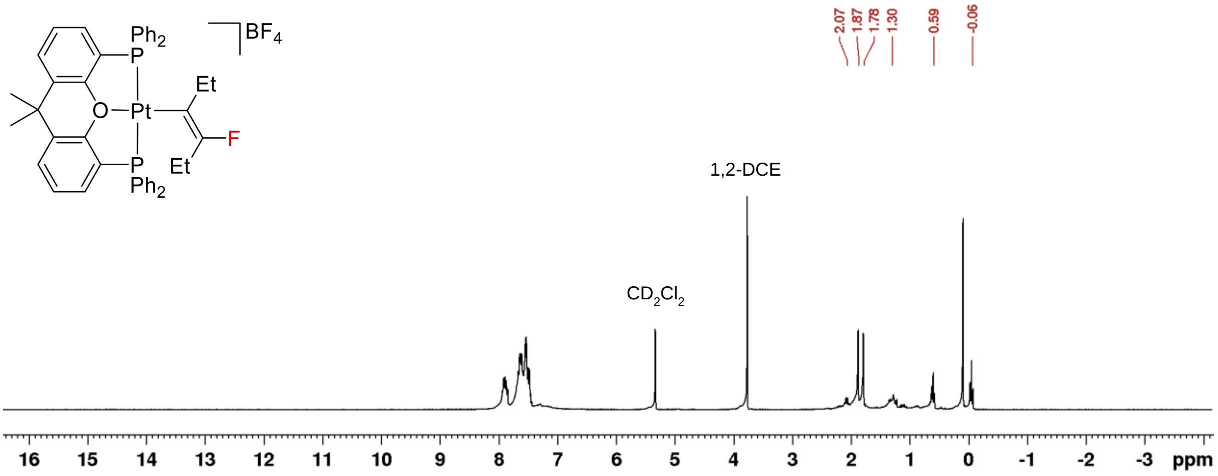

Figure S22. ^{1}H NMR spectrum of $[Pt(C_2H_5\mathit{C}{=}CFC_2H_5)(\kappa^3\text{-Xantphos})][BF_4]$ (**22**)

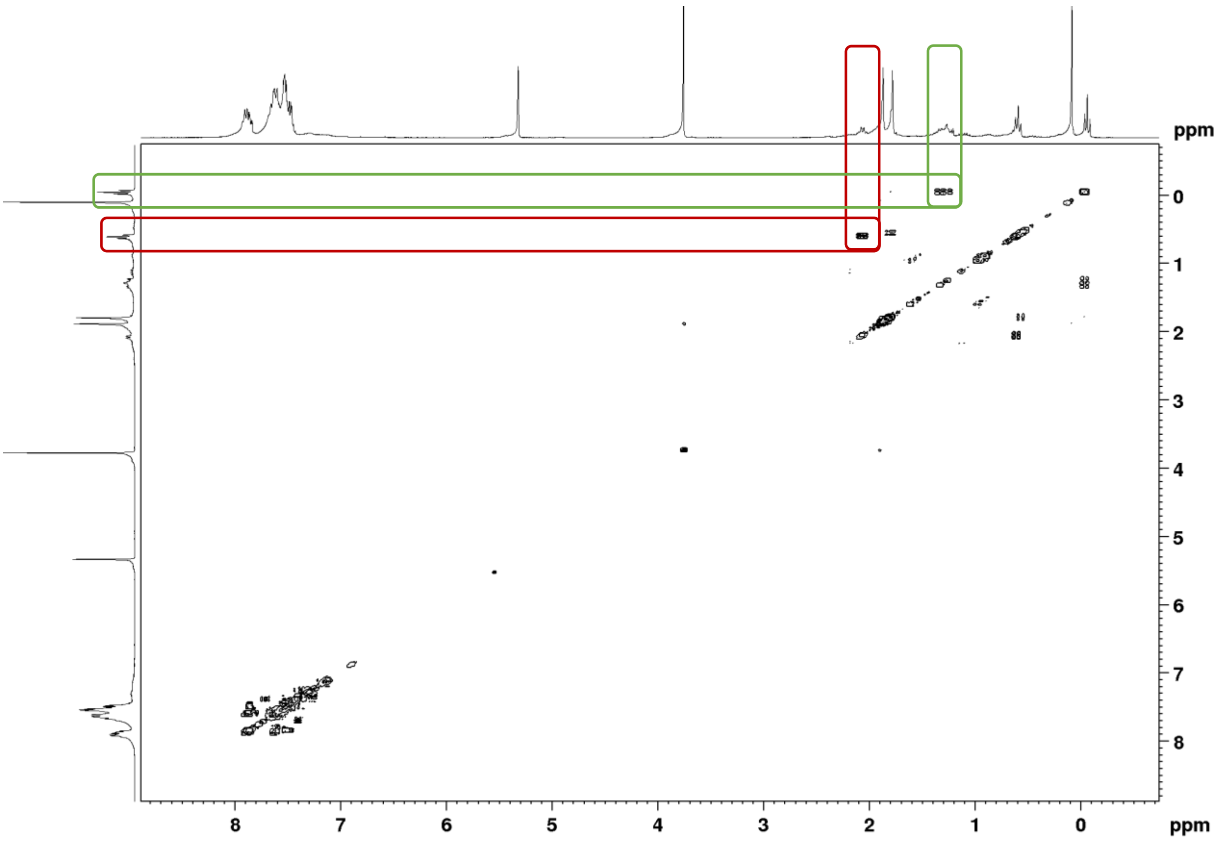

Figure S23. ^{1}H-^{1}H Cosy NMR spectrum of $[Pt(C_2H_5\mathit{C}{=}CFC_2H_5)(\kappa^3\text{-Xantphos})][BF_4]$ (**22**)

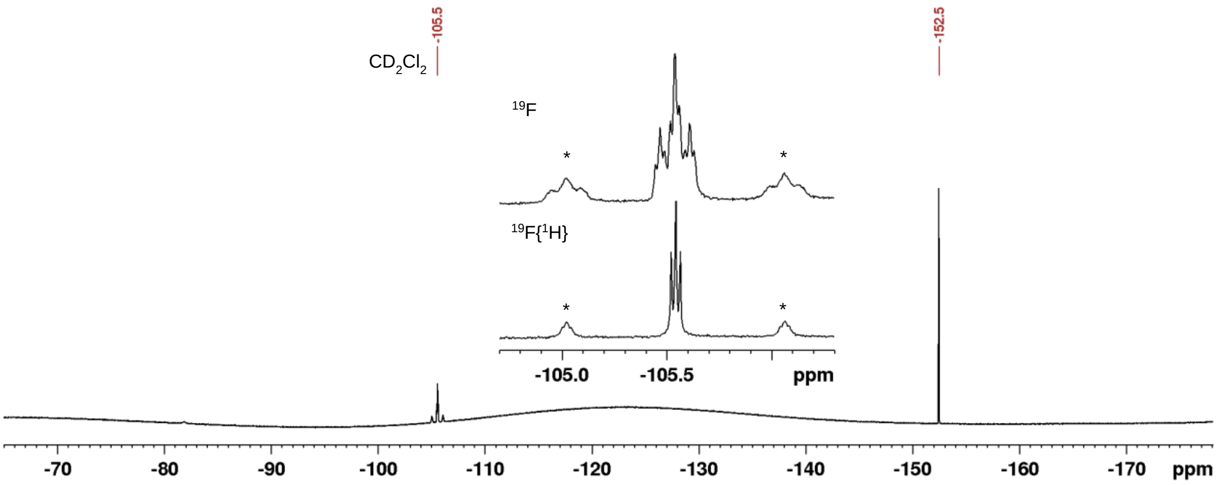

Figure S24. ^{19}F NMR spectrum of [Pt(C_2H_5*C*=CFC_2H_5)(κ^3-Xantphos)][BF_4] (**22**). * depict ^{195}Pt satellites.

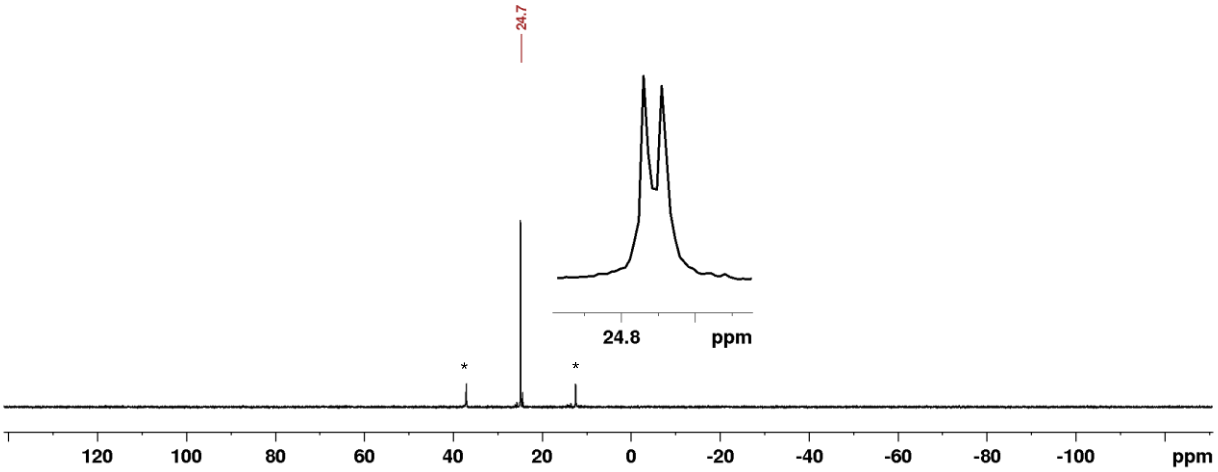

Figure S25. $^{31}P\{^1H\}$ NMR spectrum of [Pt(C_2H_5*C*=CFC_2H_5)(κ^3-Xantphos)][BF_4] (**22**). * depict ^{195}Pt satellites.

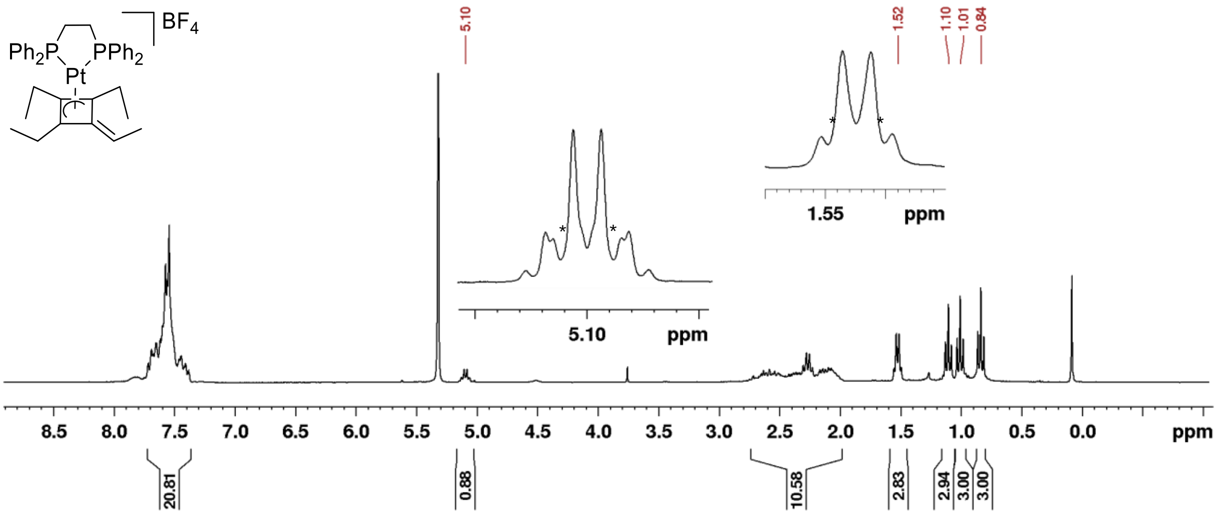

Figure S26. ^1H NMR spectrum of [Pt(η^3-(C_2H_5*C*)$_3$*C*=C_2H_4)(dppe)][BF_4] (**20-L2**). * depict ^{195}Pt satellites.

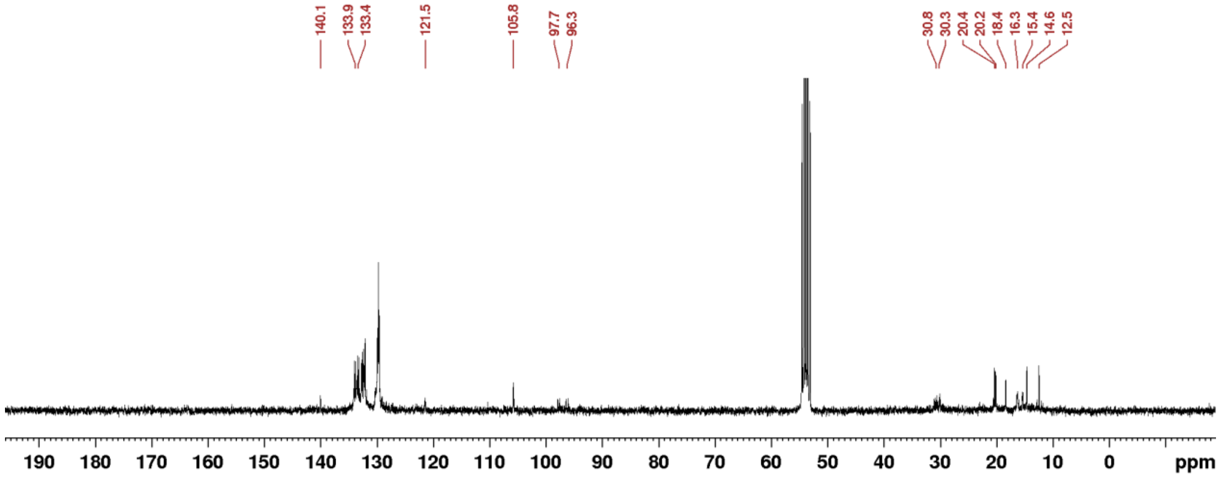

Figure S27. $^{13}C\{^{1}H\}$ NMR spectrum of $[Pt(\eta^3\text{-}(C_2H_5\mathit{C})_3\mathit{C}{=}C_2H_4)(dppe)][BF_4]$ (**20-L2**).

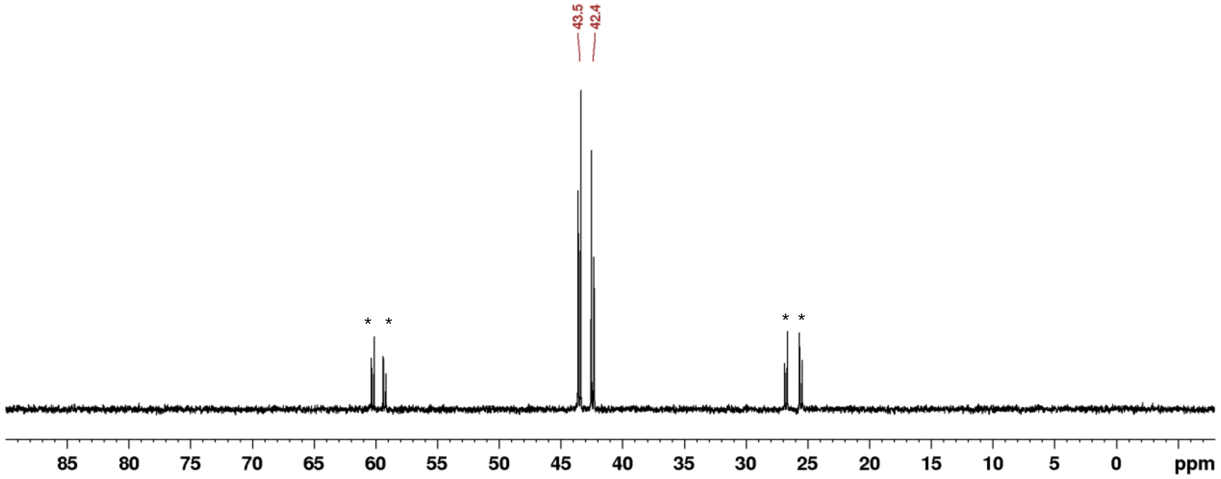

Figure S28. $^{31}P\{^{1}H\}$ NMR spectrum of $[Pt(\eta^3\text{-}(C_2H_5\mathit{C})_3\mathit{C}{=}C_2H_4)(dppe)][BF_4]$ (**20-L2**). * depict ^{195}Pt satellites.

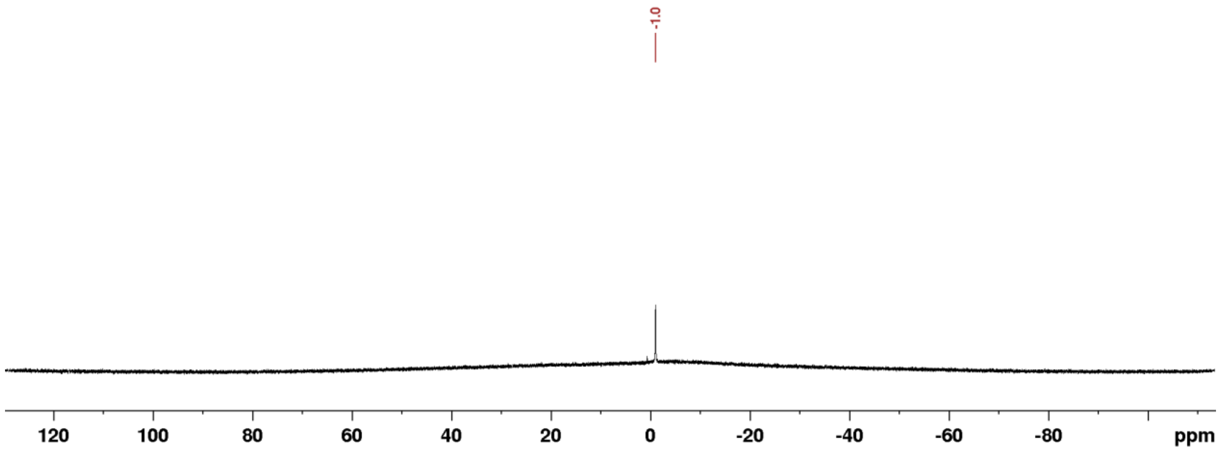

Figure S29. $^{11}B\{^{1}H\}$ NMR spectrum of $[Pt(\eta^3\text{-}(C_2H_5\mathit{C})_3\mathit{C}{=}C_2H_4)(dppe)][BF_4]$ (**20-L2**).

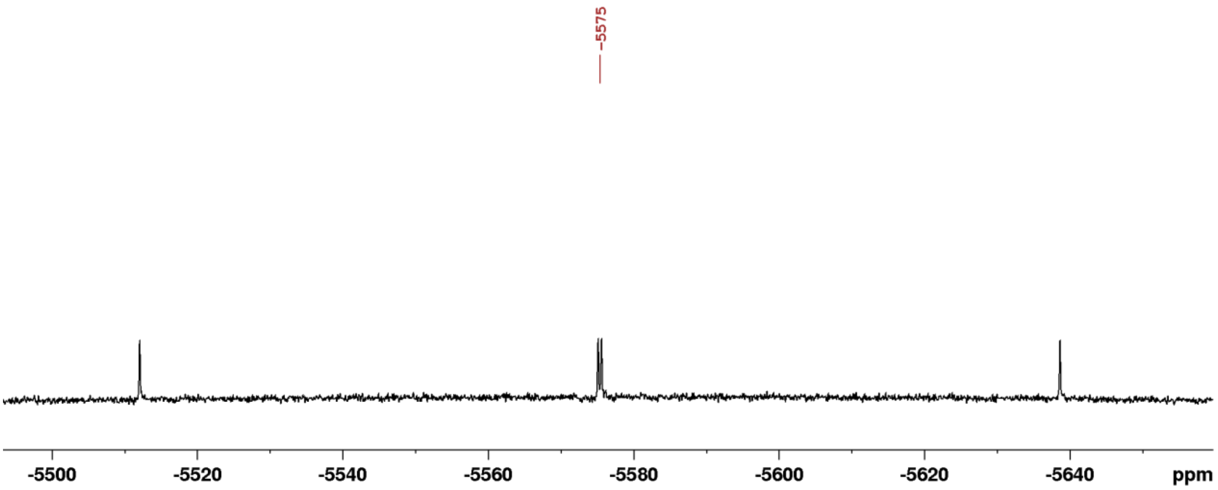

Figure S30. $^{195}Pt\{^1H\}$ NMR spectrum of $[Pt(\eta^3\text{-}(C_2H_5C)_3C{=}C_2H_4)(dppe)][BF_4]$ (**20-L2**).

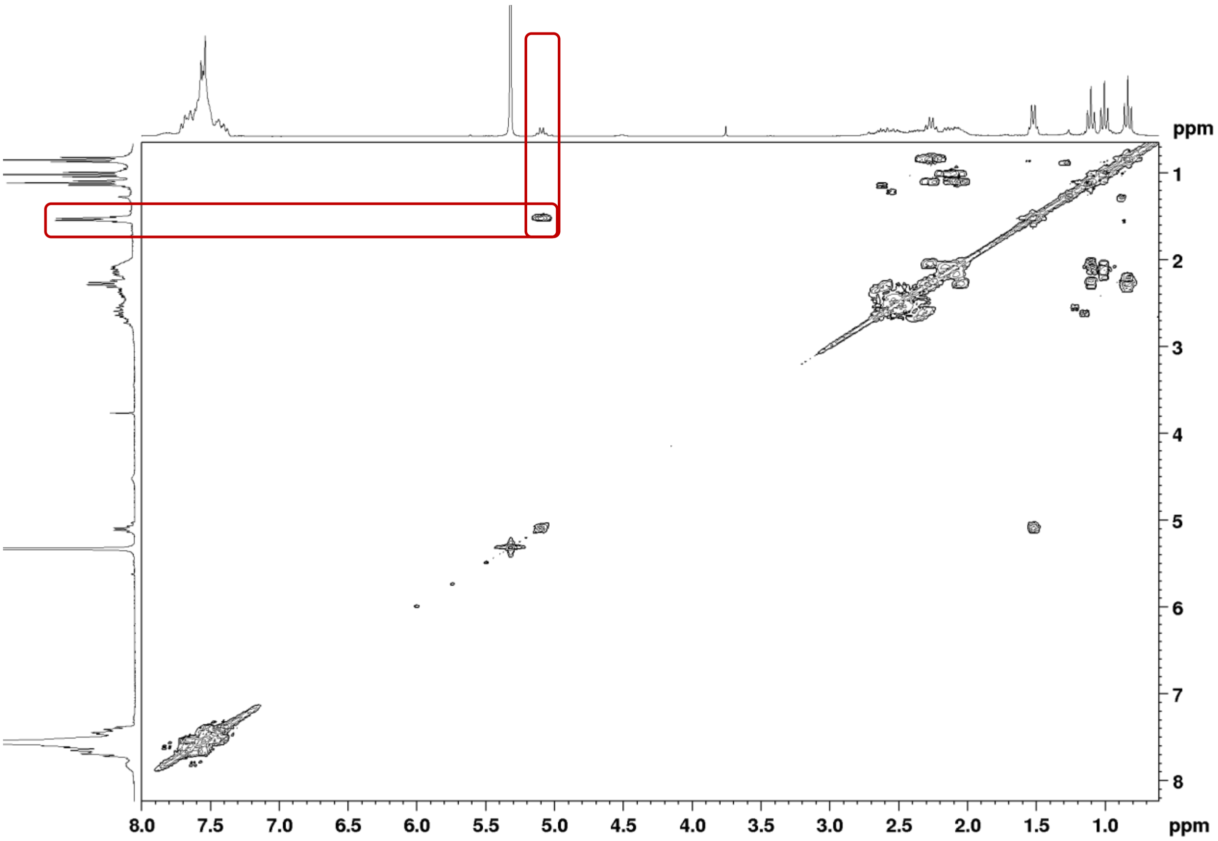

Figure S31. ^1H-^1H Cosy NMR spectrum of $[Pt(\eta^3\text{-}(C_2H_5C)_3C{=}C_2H_4)(dppe)][BF_4]$ (**20-L2**).

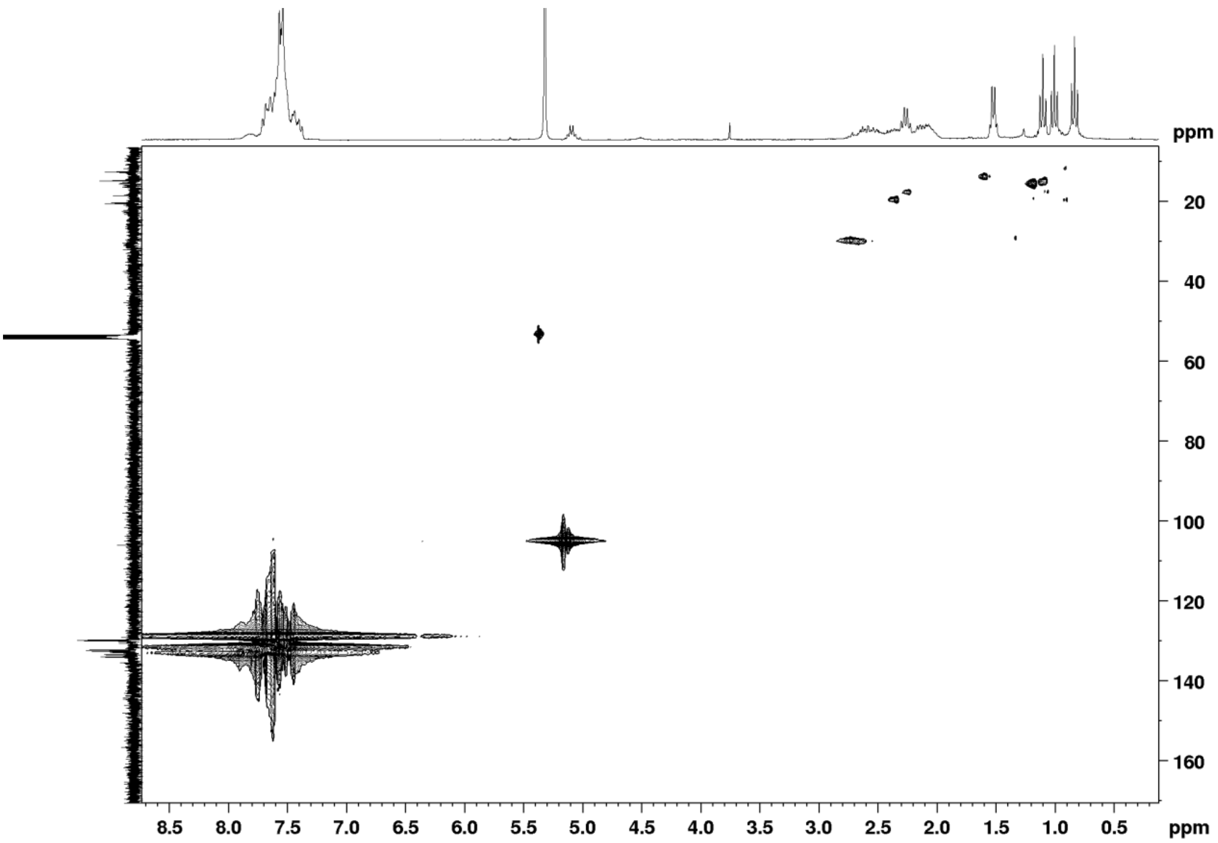

Figure S32. ^{1}H-^{13}C HSQC NMR spectrum of [Pt(η^{3}-(C_2H_5*C*)$_3$*C*=C_2H_4)(dppe)][BF_4] (**20-L2**).

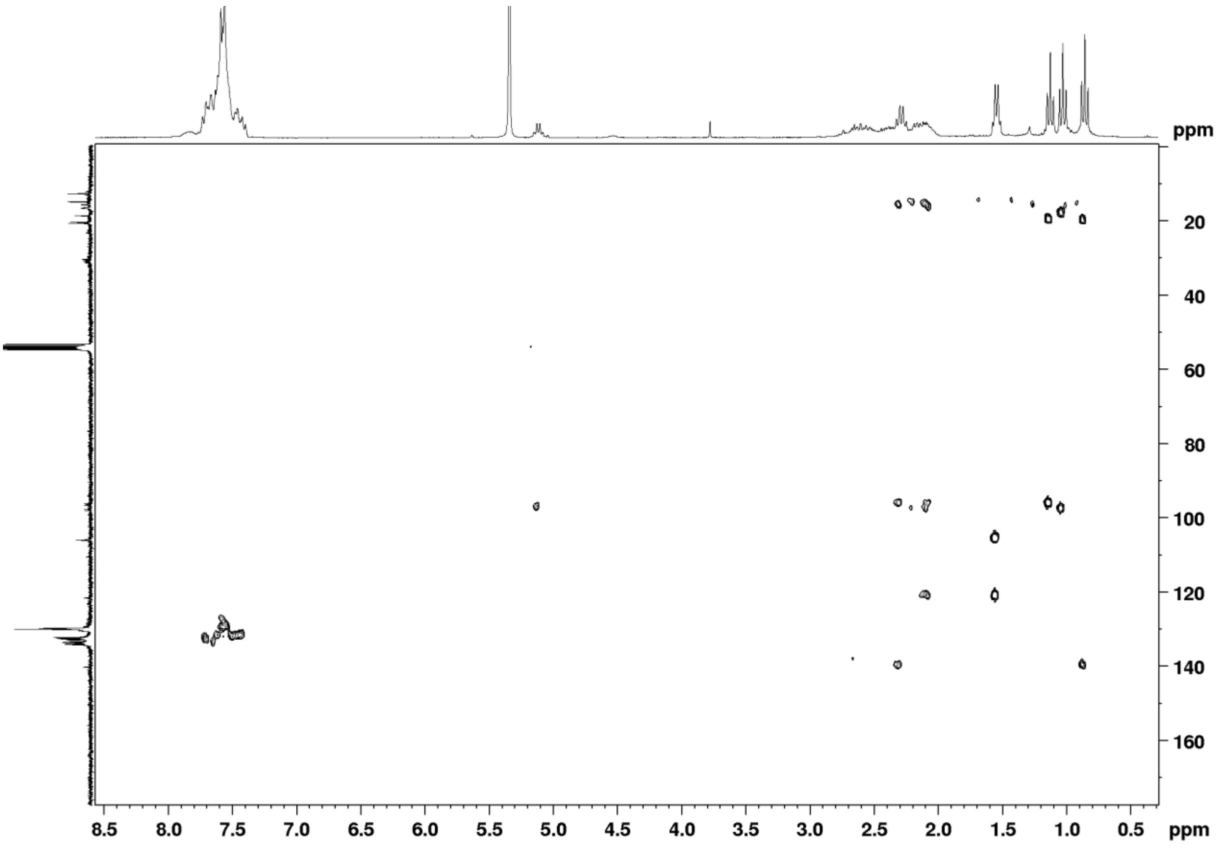

Figure S33. ^{1}H-^{13}C HMBC NMR spectrum of [Pt(η^{3}-(C_2H_5*C*)$_3$*C*=C_2H_4)(dppe)][BF_4] (**20-L2**).

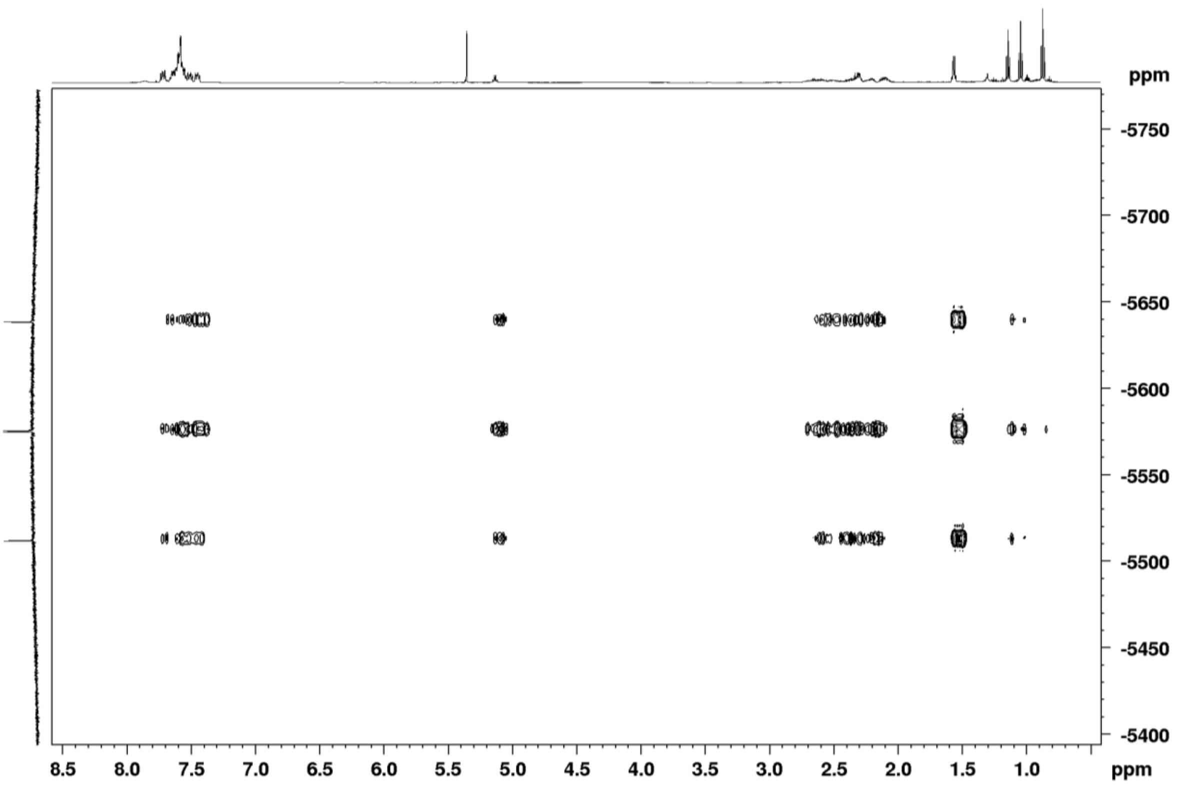

Figure S34. ^{195}Pt-^{1}H HMBC NMR spectrum of [Pt(η^3-($C_2H_5C)_3C$=C_2H_4)(dppe)][BF_4] (**20-L2**).

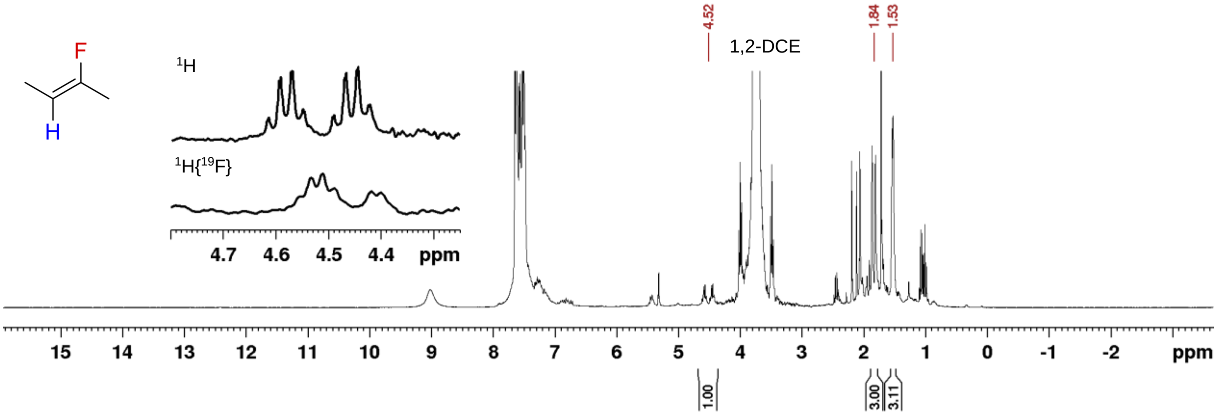

Figure S35. ^{1}H NMR spectrum of (*Z*)-3-fluoro-4,4-dimethylpent-2-ene (**3a**) from the catalytic hydrofluorination reaction of 2-butyne using **1-L3** as precatalyst and benzotrifluoride as internal standard.

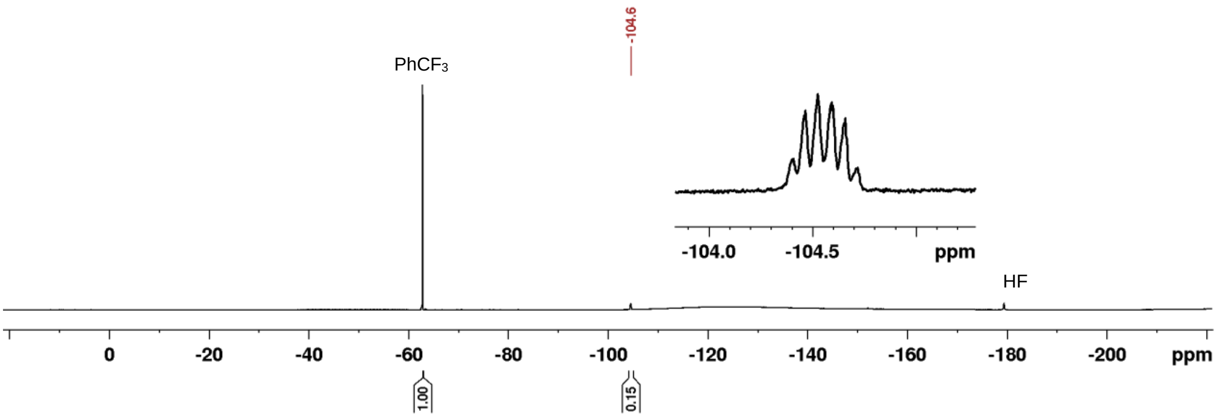

Figure S36. ^{19}F NMR spectrum (aq = 2 s, D1 = 30 s) of (*Z*)-3-fluoro-4,4-dimethylpent-2-ene (**3a**) from the catalytic hydrofluorination reaction of 2-butyne using **1-L3** as precatalyst and benzotrifluoride as internal standard.

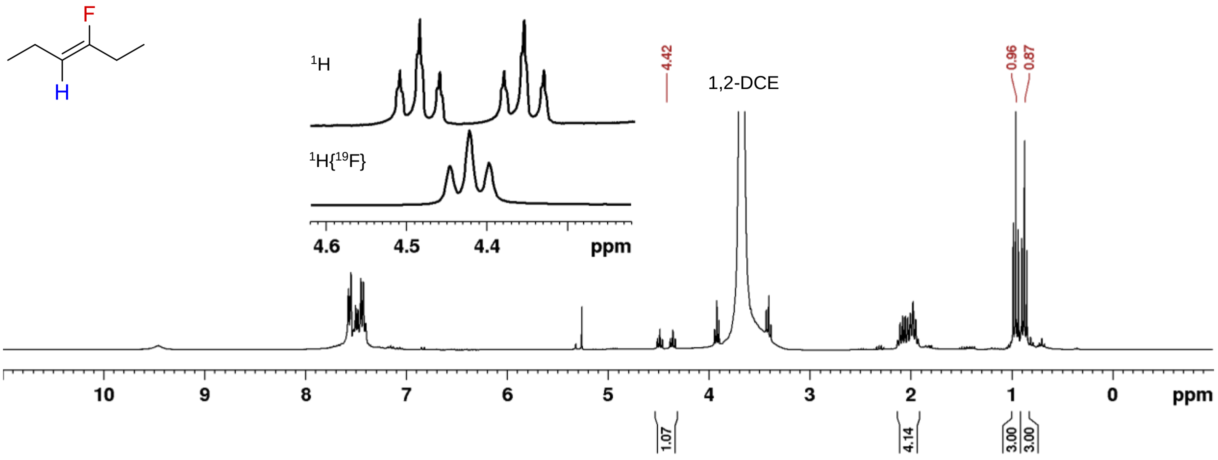

Figure S37. ^{1}H NMR spectrum of (*Z*)-3-fluoro-3-hexene (**2a**) from the catalytic hydrofluorination reaction of 3-hexyne using **1-L3** as precatalyst and benzotrifluoride as internal standard.

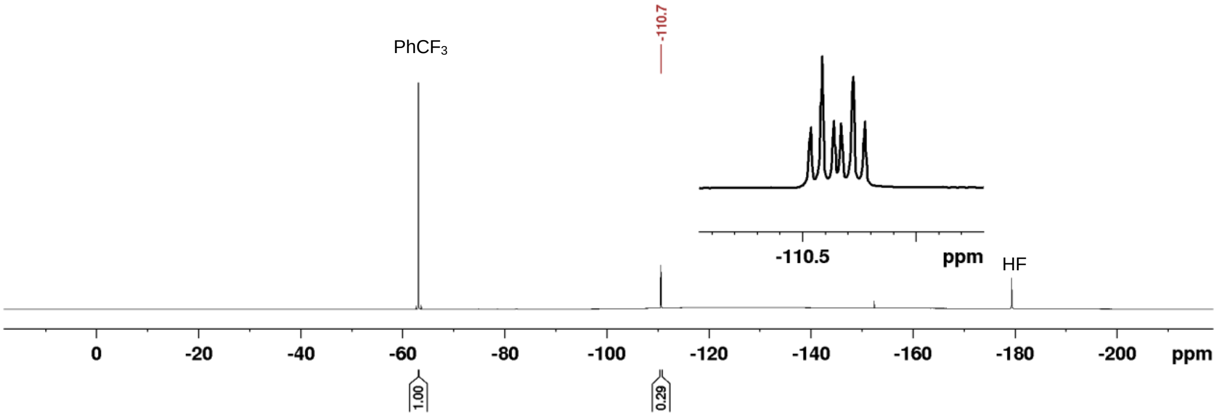

Figure S38. ^{19}F NMR spectrum (aq = 2 s, D1 = 30 s) of (*Z*)-3-fluoro-3-hexene (**2a**) from the catalytic hydrofluorination reaction of 3-hexyne using **1-L3** as precatalyst and benzotrifluoride as internal standard.

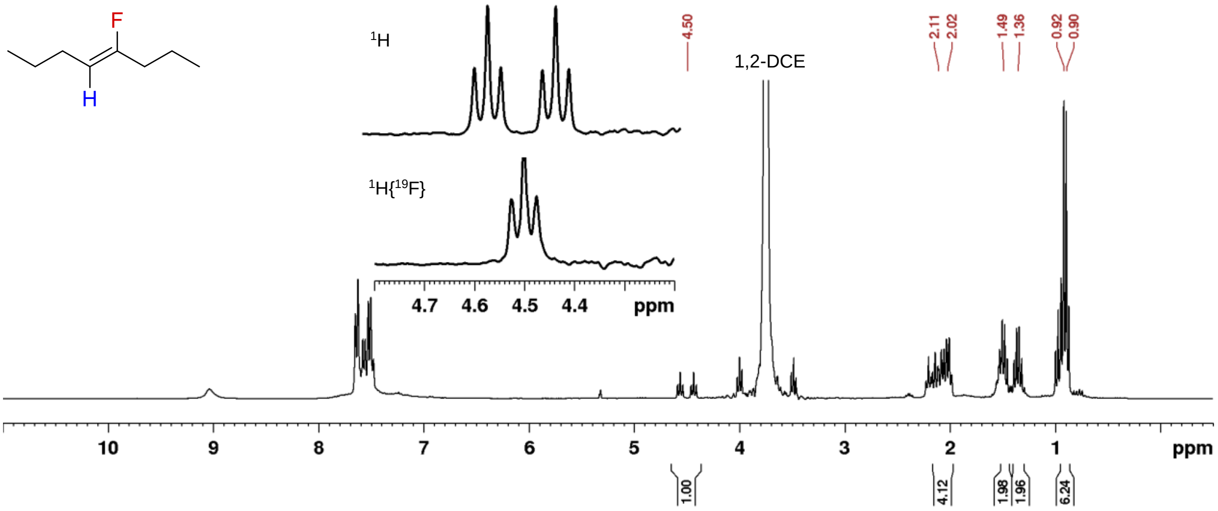

Figure S39. ^{1}H NMR spectrum of (*Z*)-4-fluoro-4-octene (**4a**) from the catalytic hydrofluorination reaction of 4-octyne using **1-L3** as precatalyst and benzotrifluoride as internal standard.

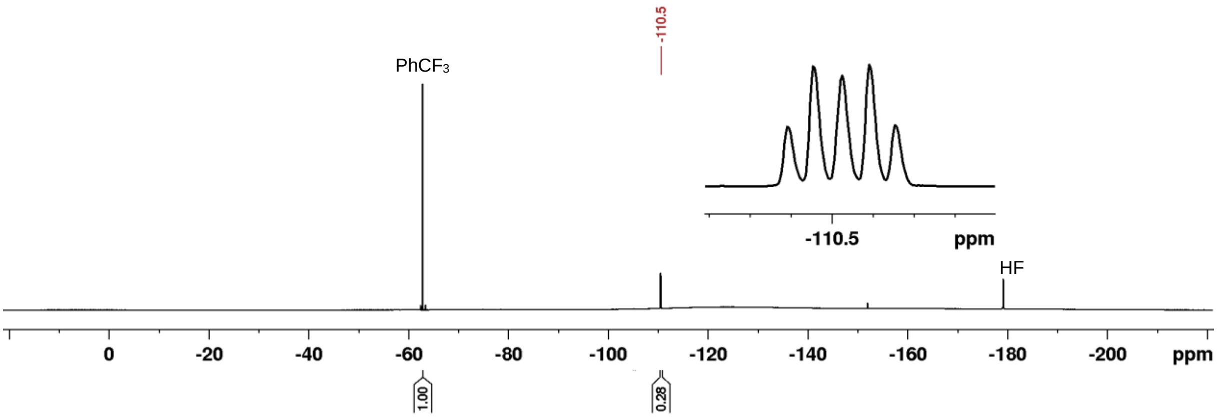

Figure S40. ^{19}F NMR spectrum (aq = 2 s, D1 = 30 s) of (*Z*)-4-fluoro-4-octene (**4a**) from the catalytic hydrofluorination reaction of 4-octyne using **1-L3** as precatalyst and benzotrifluoride as internal standard.

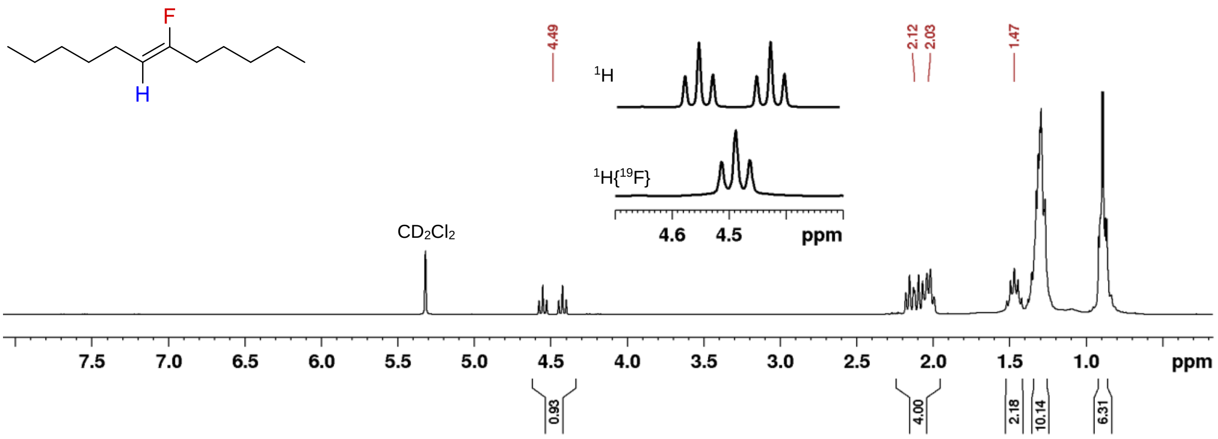

Figure S41. ^{1}H NMR spectrum of isolated (*Z*)-6-fluoro-6-dodecene (**5a**).

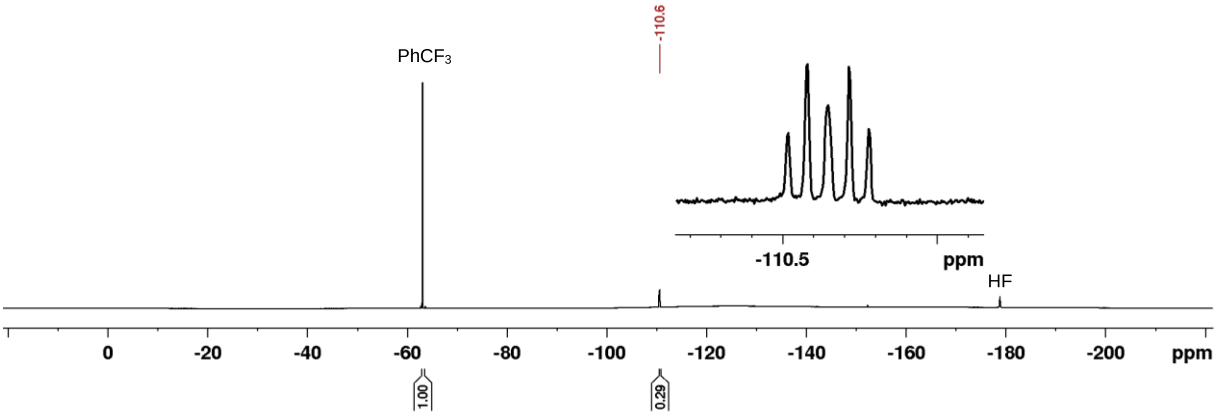

Figure S42. ^{19}F NMR spectrum (aq = 2 s, D1 = 30 s) of (*Z*)-6-fluoro-6-dodecene (**5a**) from the catalytic hydrofluorination reaction of 6-dodecyne using **1-L3** as precatalyst and benzotrifluoride as internal standard.

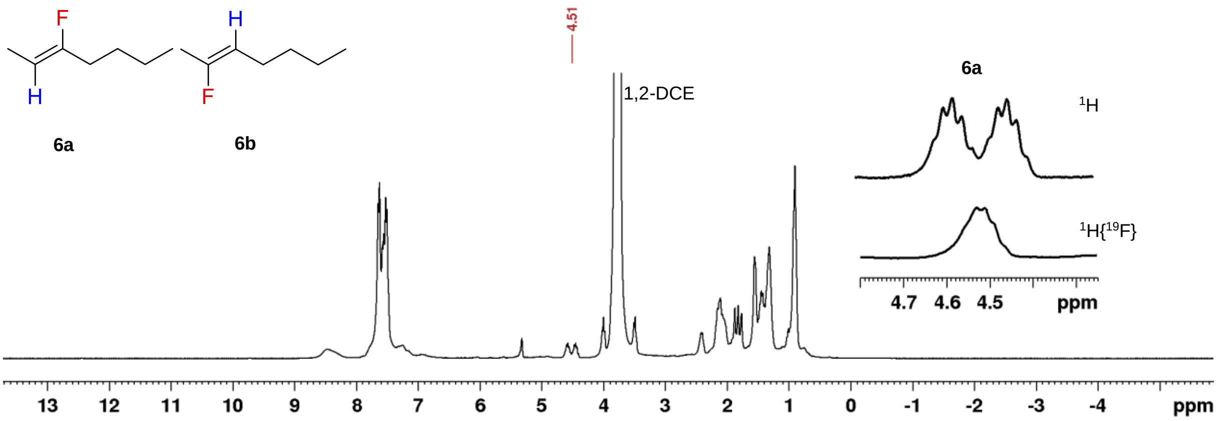

Figure S43. ^{1}H NMR spectrum of (*Z*)-3-fluoro-2-heptene (**6a**) and (*Z*)-2-fluoro-2-heptene (**6b**) (1.7:1) from the catalytic hydrofluorination reaction of 2-heptyne using **1-L3** as precatalyst and benzotrifluoride as internal standard.

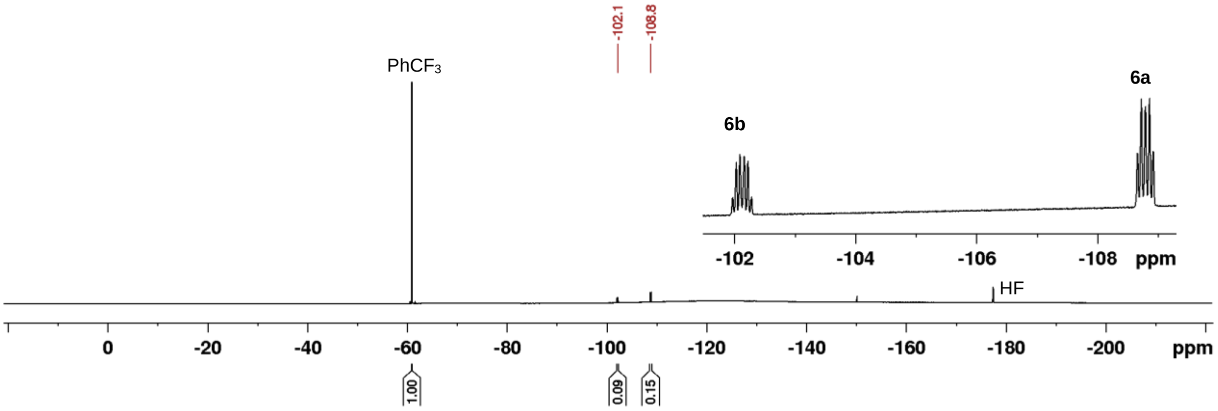

Figure S44. ^{19}F NMR spectrum (aq = 2 s, D1 = 30 s) of (*Z*)-3-fluoro-2-heptene (**6a**) and (*Z*)-2-fluoro-2-heptene (**6b**) (1.7:1) from the catalytic hydrofluorination reaction of 2-heptyne using **1-L3** as precatalyst and benzotrifluoride as internal standard.

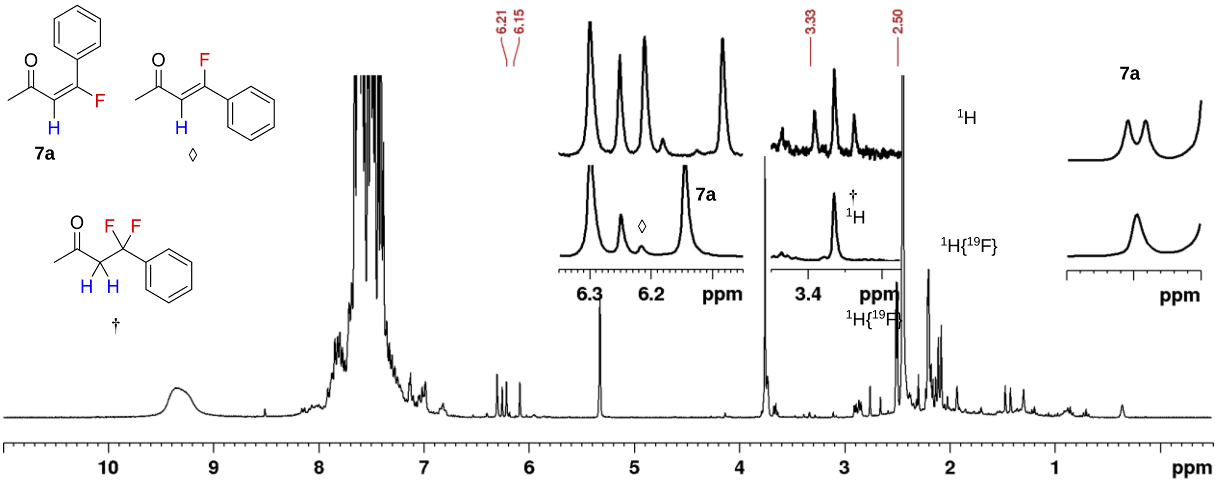

Figure S45. ^{1}H NMR spectrum of (*Z*)-4-fluoro-4-phenylbut-3-en-2-one (**7a**), (*E*)-4-fluoro-4-phenylbut-3-en-2-one (◊) and 4,4-difluoro-4-phenylbutan-2-one (†) (8:1:1) from the catalytic hydrofluorination reaction of 4-phenyl-3-butyn-2-one using **1-L3** as precatalyst and benzotrifluoride as internal standard.

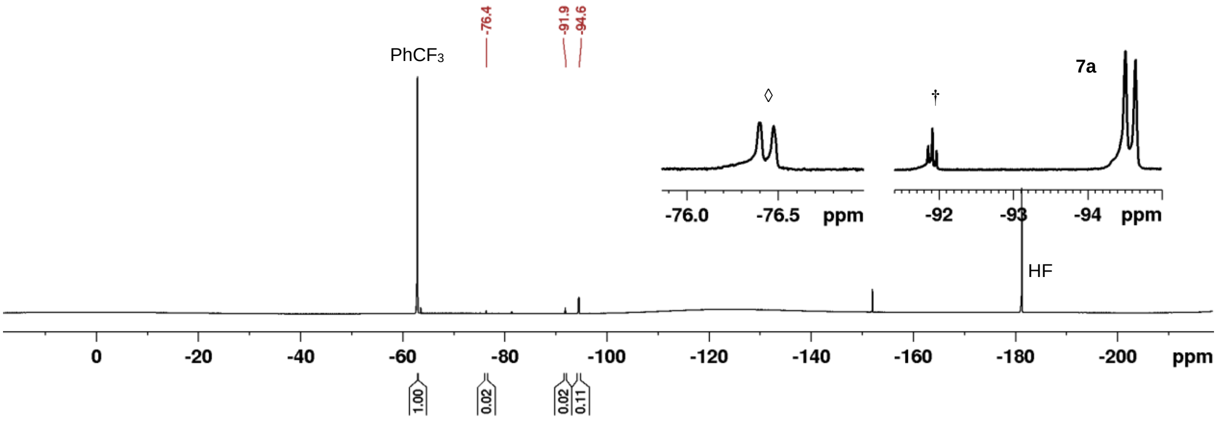

Figure S46. ^{19}F NMR spectrum (aq = 2 s, D1 = 30 s) of (*Z*)-4-fluoro-4-phenylbut-3-en-2-one (**7a**), (*E*)-4-fluoro-4-phenylbut-3-en-2-one (◊) and 4,4-difluoro-4-phenylbutan-2-one (†) (8:1:1) from the catalytic hydrofluorination reaction of 4-phenyl-3-butyn-2-one using **1-L3** as precatalyst and benzotrifluoride as internal standard.

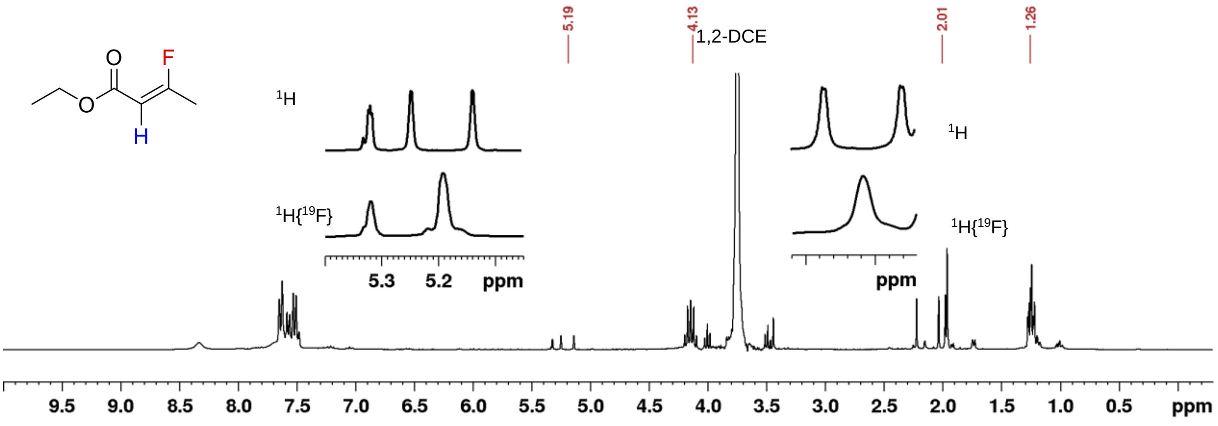

Figure S47. ^{1}H NMR spectrum of (*Z*)-ethyl-3-fluorobut-2-enoate (**8a**) from the catalytic hydrofluorination reaction of ethyl 2-butynoate using **1-L3** as precatalyst and benzotrifluoride as internal standard.

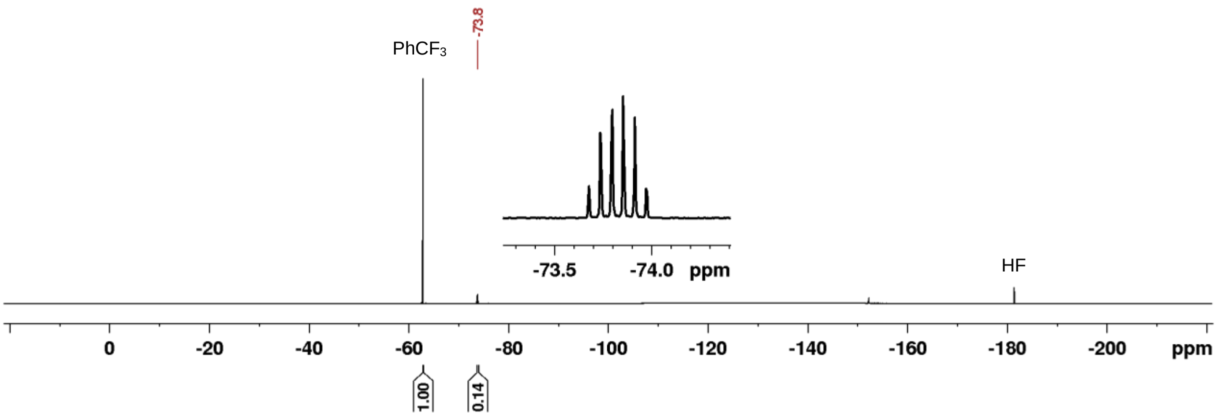

Figure S48. ^{19}F NMR spectrum (aq = 2 s, D1 = 30 s) of (*Z*)-ethyl-3-fluorobut-2-enoate (**8a**) from the catalytic hydrofluorination reaction of ethyl 2-butynoate using **1-L3** as precatalyst and benzotrifluoride as internal standard.

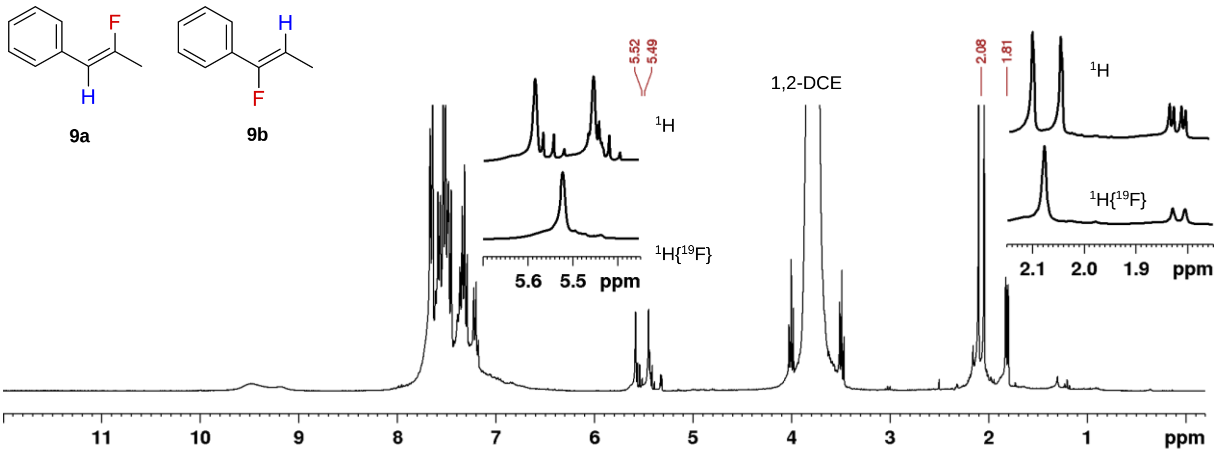

Figure S49. ^{1}H NMR spectrum of (*Z*)-(2-fluoroprop-1-en-1-yl)benzene (**9a**) and (*Z*)-(1-fluoroprop-1-en-1-yl)benzene (**9b**) (2:1) from the catalytic hydrofluorination reaction of 1-phenylpropyne using **1-L3** as precatalyst and benzotrifluoride as internal standard.

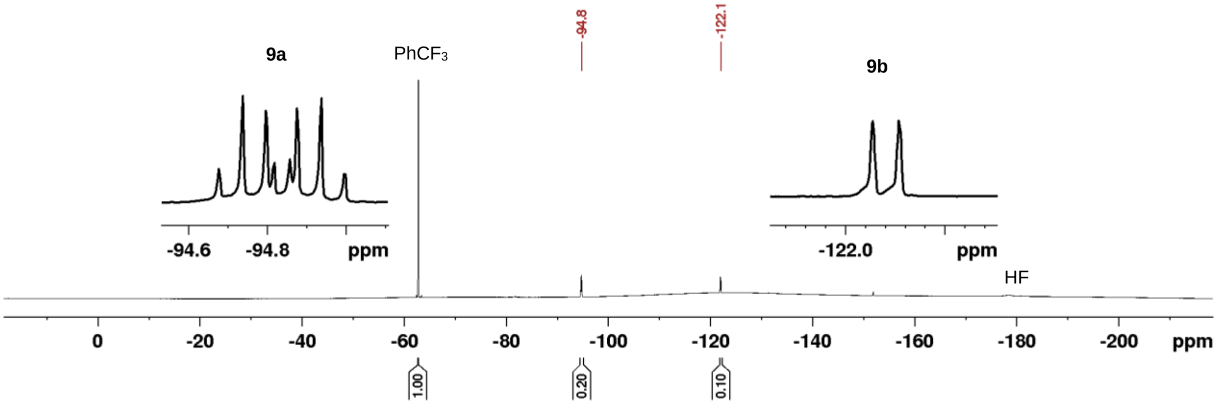

Figure S50. ^{19}F NMR spectrum (aq = 2 s, D1 = 30 s) of (*Z*)-(2-fluoroprop-1-en-1-yl)benzene (**9a**) and (*Z*)-(1-fluoroprop-1-en-1-yl)benzene (**9b**) (2:1) from the catalytic hydrofluorination reaction of 1-phenylpropyne using **1-L3** as precatalyst and benzotrifluoride as internal standard.

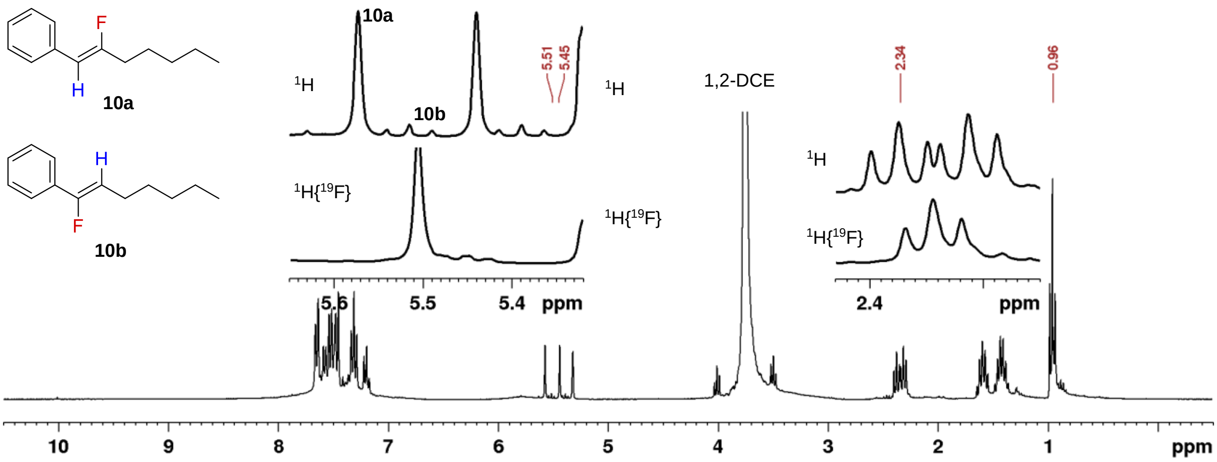

Figure S51. ^{1}H NMR spectrum of (*Z*)-(2-fluorohept-1-en-1-yl)benzene (**10a**) and (*Z*)-(1-fluorohept-1-en-1-yl)benzene (**10b**) (8.3:1) from the catalytic hydrofluorination reaction of 1-phenyl-1-hexyne using **1-L3** as precatalyst and benzotrifluoride as internal standard.

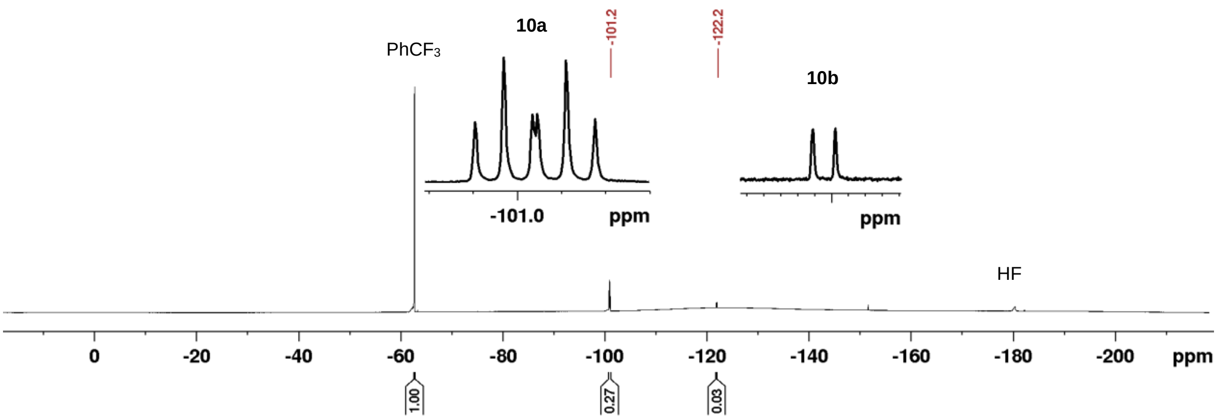

Figure S52. ^{19}F NMR spectrum (aq = 2 s, D1 = 30 s) of (*Z*)-(2-fluorohept-1-en-1-yl)benzene (**10a**) and (*Z*)-(1-fluorohept-1-en-1-yl)benzene (**10b**) (8.3:1) from the catalytic hydrofluorination reaction of 1-phenyl-1-hexyne using **1-L3** as precatalyst and benzotrifluoride as internal standard.

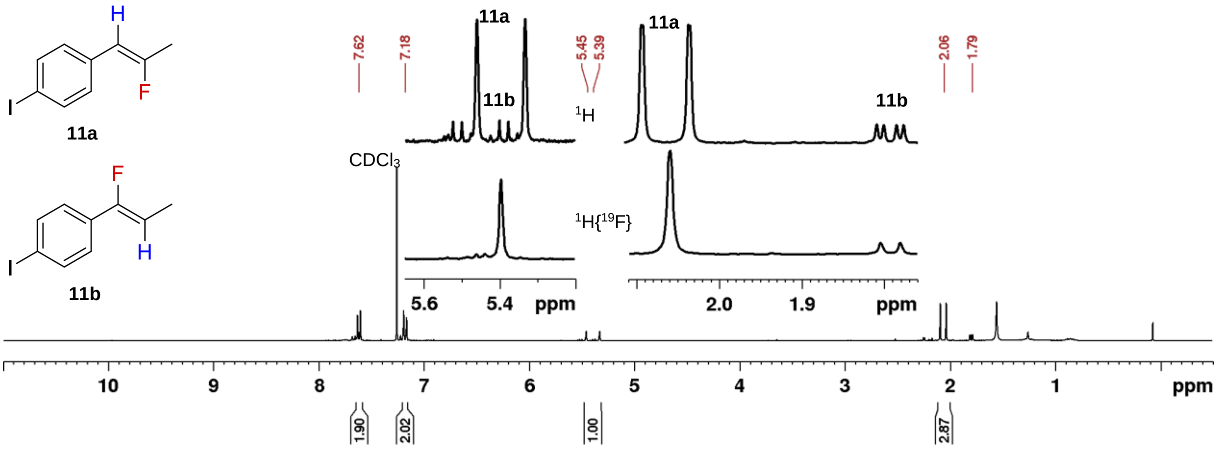

Figure S53. ^{1}H NMR spectrum of isolated (*Z*)-1-(2-fluoroprop-1-en-1-yl)-4-iodobenzene (**11a**) and (*Z*)-1-(2-fluoroprop-1-en-1-yl)-4-iodobenzene (**11b**) (5:1).

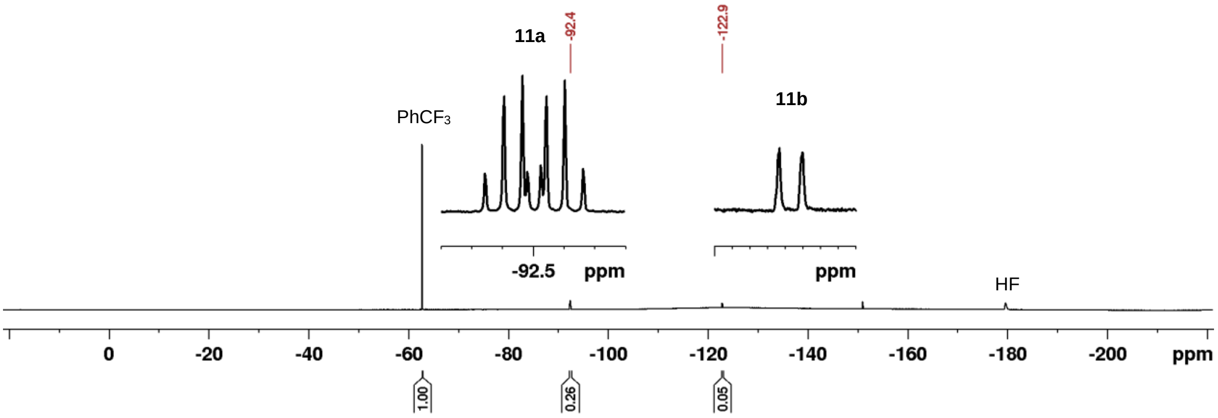

Figure S54. ^{19}F NMR spectrum (aq = 2 s, D1 = 30 s) of (*Z*)-1-(2-fluoroprop-1-en-1-yl)-4-iodobenzene (**11a**) and (*Z*)-1-(2-fluoroprop-1-en-1-yl)-4-iodobenzene (**11b**) (5:1) from the catalytic hydrofluorination reaction of 1-iodo-4-(prop-1-yn-1-yl)benzene using **1-L3** as precatalyst and benzotrifluoride as internal standard.

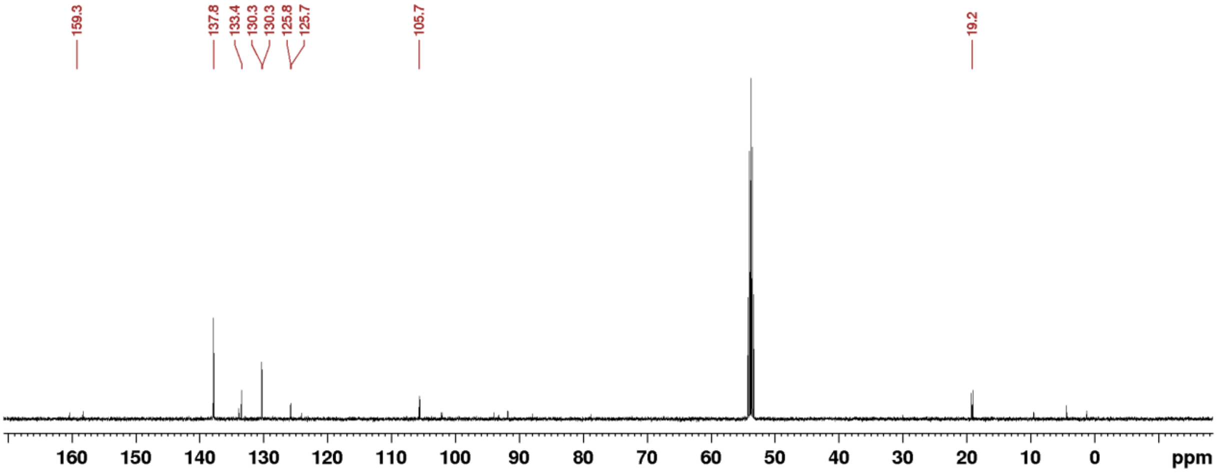

Figure S55. $^{13}C\{^{1}H\}$ NMR spectrum of isolated (*Z*)-1-(2-fluoroprop-1-en-1-yl)-4-iodobenzene (**11a**) and (*Z*)-1-(2-fluoroprop-1-en-1-yl)-4-iodobenzene (**11b**) (5:1).

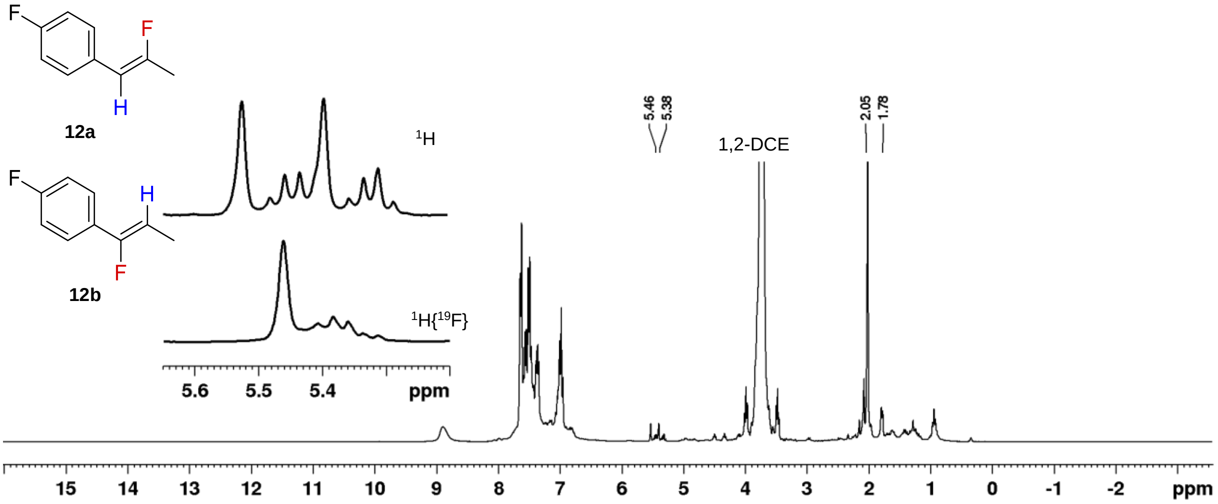

Figure S56. ^{1}H NMR spectrum of (*Z*)-1-(2-fluoroprop-1-en-1-yl)-4-fluorobenzene (**12a**) and (*Z*)-1-(1-fluoroprop-1-en-1-yl)-4-fluorobenzene (**12b**) (1.6:1) from the catalytic hydrofluorination reaction of 1-fluoro-4-(prop-1-yn-1-yl)benzene using **1-L3** as precatalyst and benzotrifluoride as internal standard.

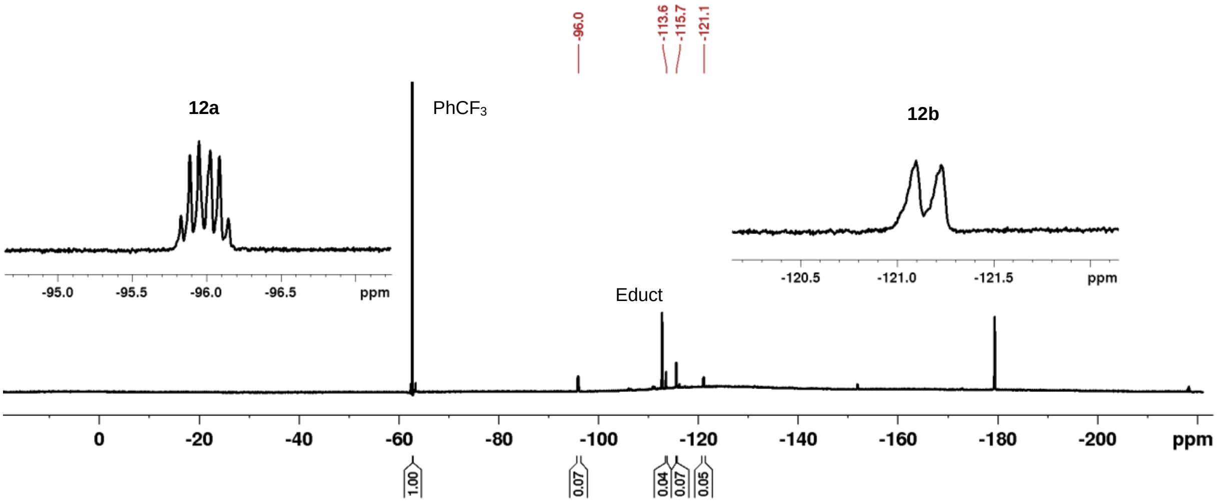

Figure S57. ^{19}F NMR spectrum (aq = 2 s, D1 = 30 s) of (*Z*)-1-(2-fluoroprop-1-en-1-yl)-4-fluorobenzene (**12a**) and (*Z*)-1-(1-fluoroprop-1-en-1-yl)-4-fluorobenzene (**12b**) (1.6:1) from the catalytic hydrofluorination reaction of 1-fluoro-4-(prop-1-yn-1-yl)benzene using **1-L3** as precatalyst and benzotrifluoride as internal standard.

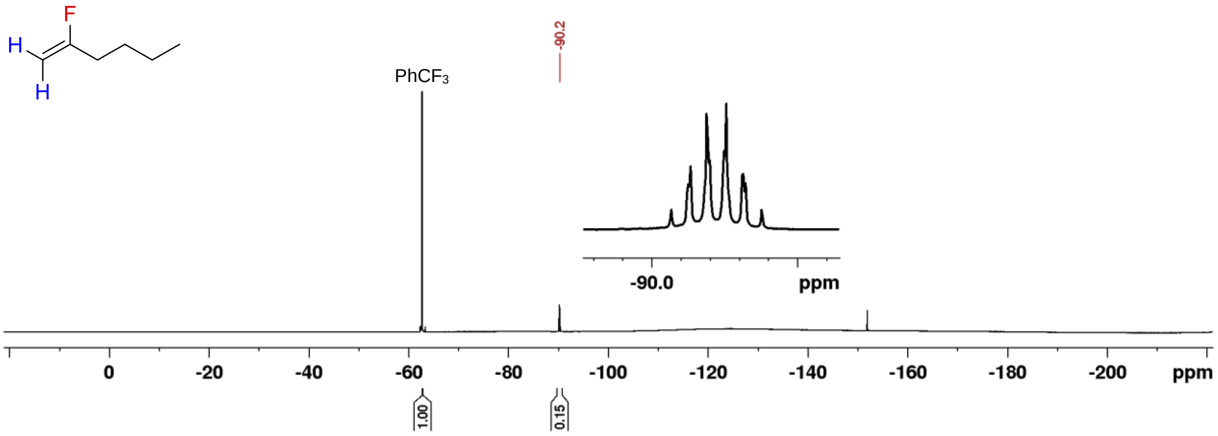

Figure S58. ^{19}F NMR spectrum (aq = 2 s, D1 = 30 s) of (*Z*)-2-fluoro-1-hexene (**13a**) from the catalytic hydrofluorination reaction of 1-hexyne using **1-L3** as precatalyst and benzotrifluoride as internal standard.

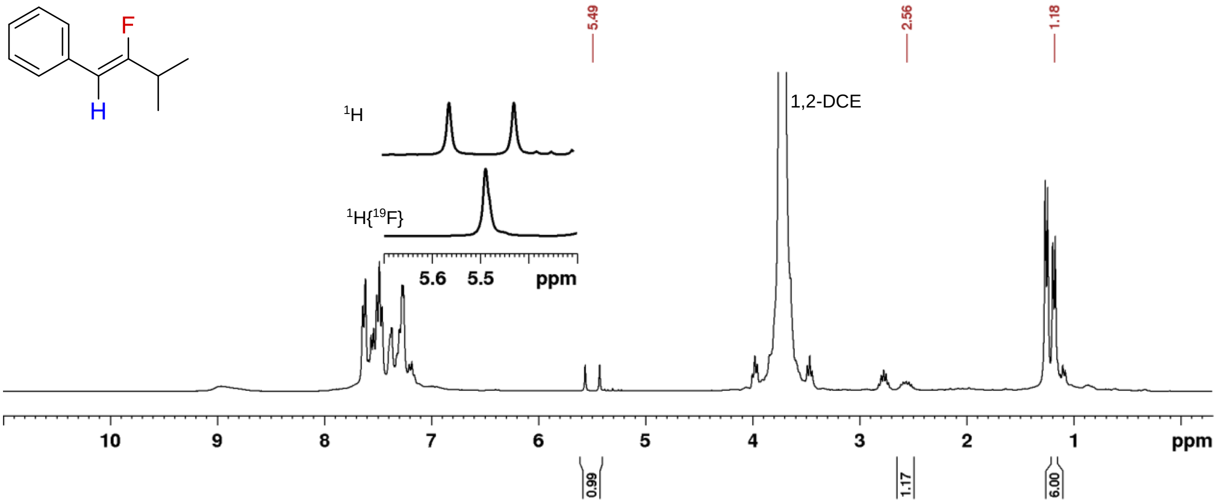

Figure S59. ^{1}H NMR spectrum of (*Z*)-(2-fluoro-3-methylbut-1-en-1-yl)benzene (**14a**) from the catalytic hydrofluorination reaction of (3-methyl-1-butyn-1-yl)benzene using **1-L3** as precatalyst and benzotrifluoride as internal standard.

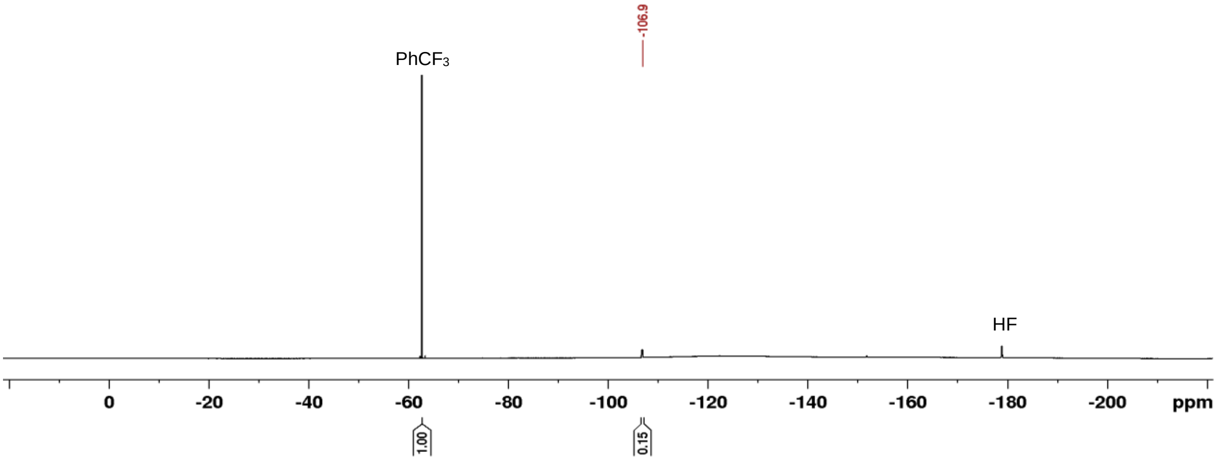

Figure S60. ^{19}F NMR spectrum (aq = 2 s, D1 = 30 s) of (*Z*)-(2-fluoro-3-methylbut-1-en-1-yl)benzene (**14a**) from the catalytic hydrofluorination reaction of (3-methyl-1-butyn-1-yl)benzene using **1-L3** as precatalyst and benzotrifluoride as internal standard.

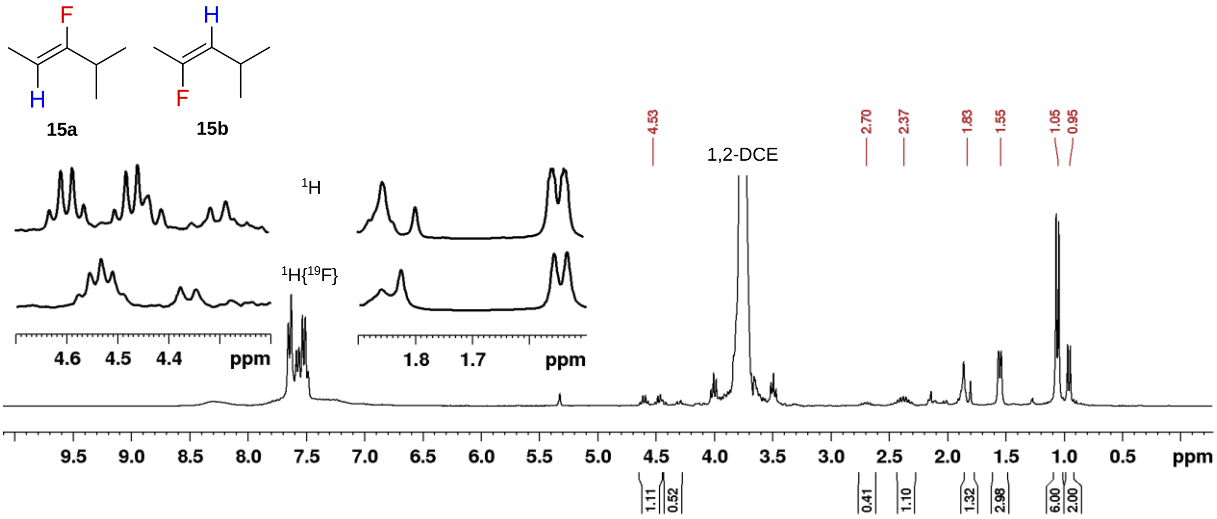

Figure S61. ^{1}H NMR spectrum of (*Z*)-3-fluoro-4-methylpent-2-ene (**15a**) and (*Z*)-2-fluoro-4-methylpent-2-ene (**15b**) as a mixture of isomers in a ratio of 3.1:1 from the catalytic hydrofluorination reaction of 4-methylpentyne using **1-L3** as precatalyst and benzotrifluoride as internal standard.

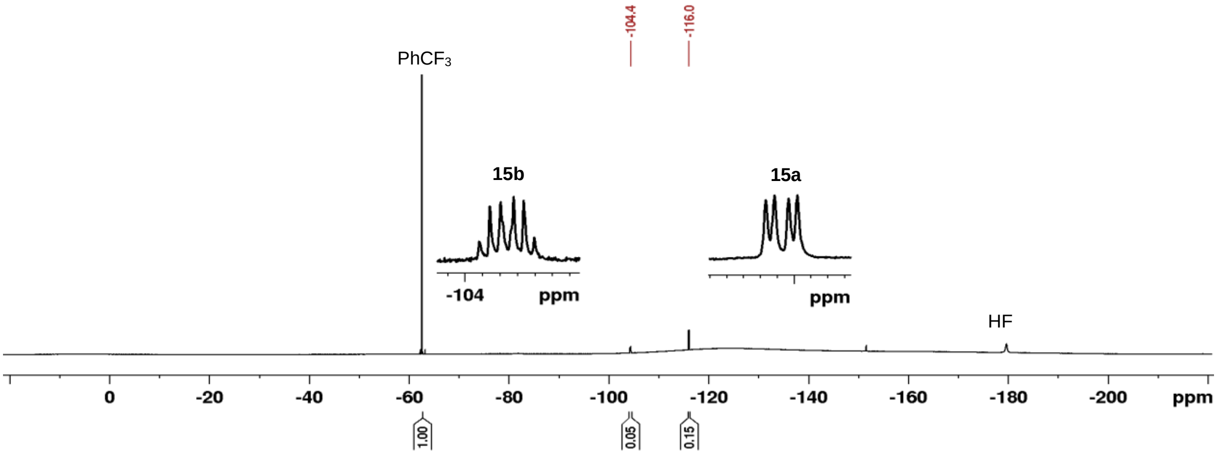

Figure S62. ^{19}F NMR spectrum (aq = 2 s, D1 = 30 s) of (*Z*)-3-fluoro-4-methylpent-2-ene (**15a**) and (*Z*)-2-fluoro-4-methylpent-2-ene (**15b**) as a mixture of isomers in a ratio of 3.1:1 from the catalytic hydrofluorination reaction of 4-methylpentyne using **1-L3** as precatalyst and benzotrifluoride as internal standard.

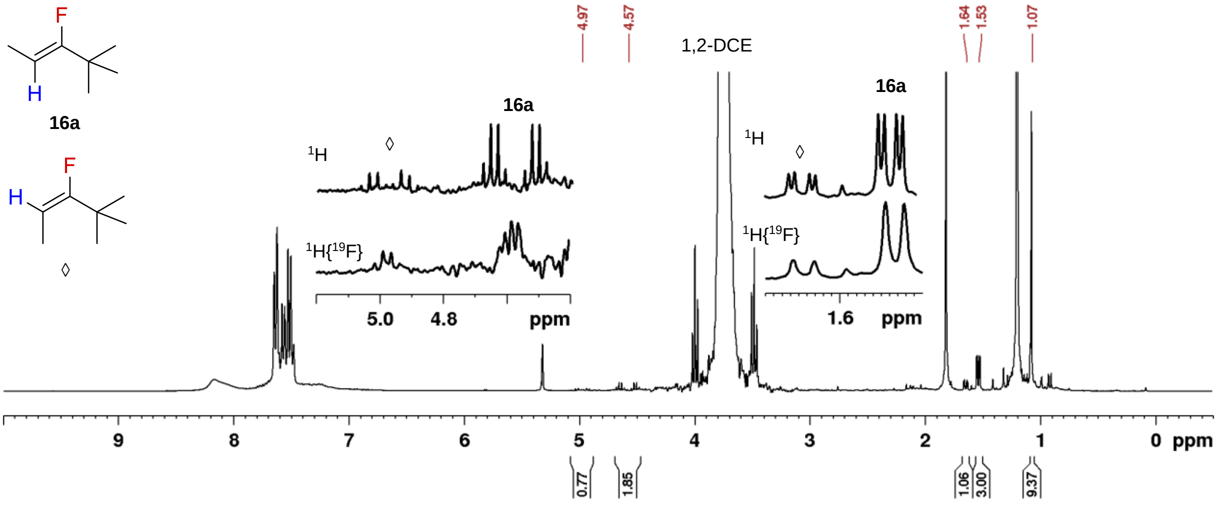

Figure S63. ^{1}H NMR spectrum of (*Z*)-3-fluoro-4,4-dimethylpent-2-ene (**16a**) and (*E*)-3-fluoro-4,4-dimethylpent-2-ene (◊) as a mixture of isomers in a ratio of 3:1 from the catalytic hydrofluorination reaction of 4,4-dimethyl-2-pentyne using **1-L3** as precatalyst and benzotrifluoride as internal standard.

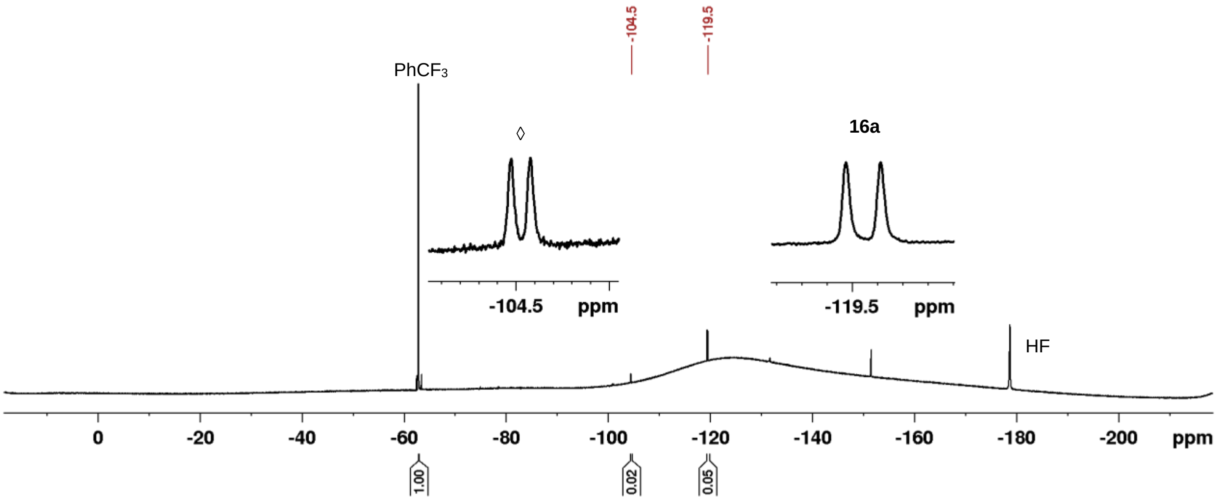

Figure S64. ^{19}F NMR spectrum (aq = 2 s, D1 = 30 s) of (*Z*)-3-fluoro-4,4-dimethylpent-2-ene (**16a**) and (*E*)-3-fluoro-4,4-dimethylpent-2-ene (◊) as a mixture of isomers in a ratio of 3:1 from the catalytic hydrofluorination reaction of 4,4-dimethyl-2-pentyne using **1-L3** as precatalyst and benzotrifluoride as internal standard.

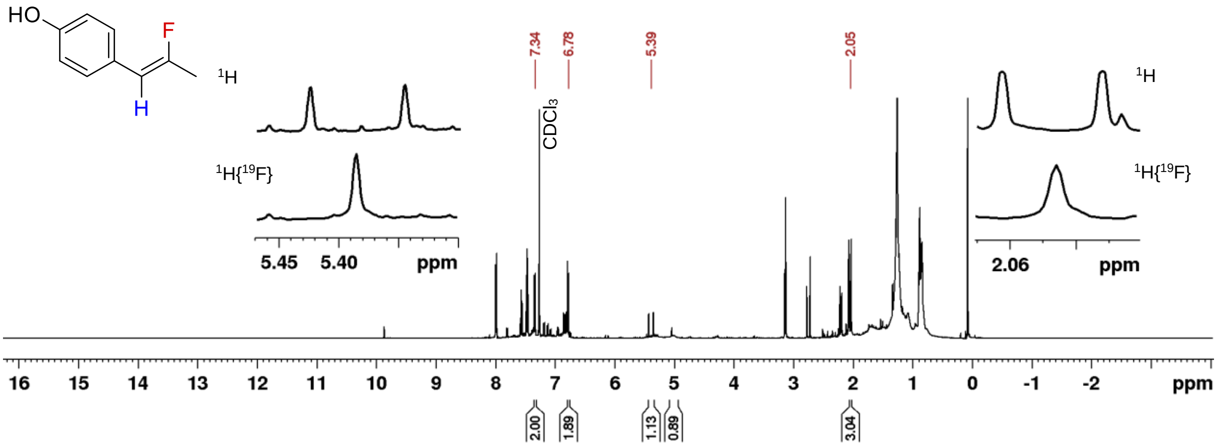

Figure S65. Crude ^{1}H NMR spectrum of (*Z*)-4-(2-fluoroprop-1-en-1-yl)phenol (**17a**) from the hydroxylation reaction of (*Z*)-3-fluoro-4,4-dimethylpent-2-ene (**11a**).

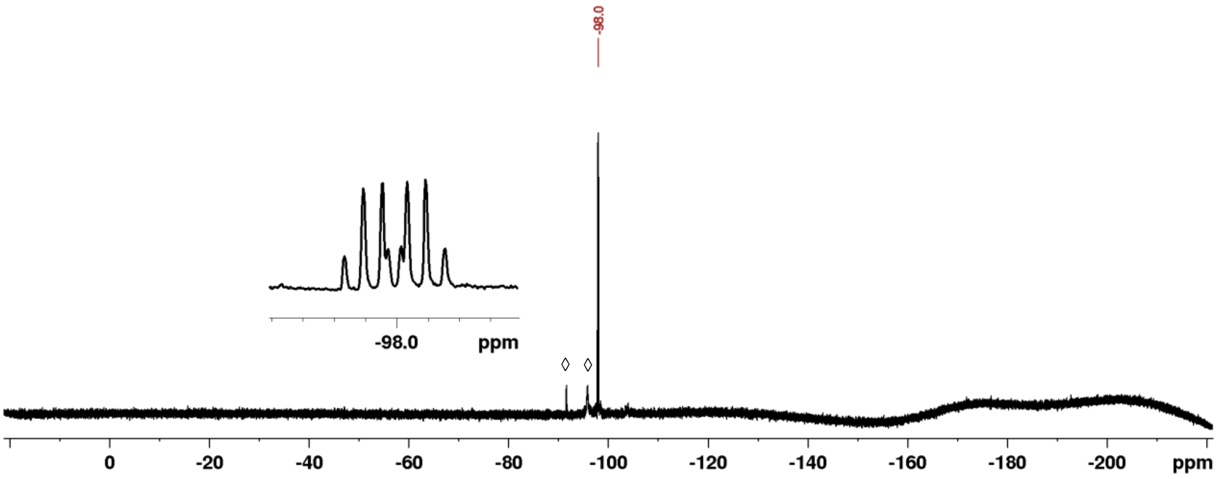

Figure S66. Crude ^{19}F NMR spectrum of (*Z*)-4-(2-fluoroprop-1-en-1-yl)phenol (**17a**) from the hydroxylation reaction of (*Z*)-3-fluoro-4,4-dimethylpent-2-ene (**11a**). ◊ unidentified compounds.

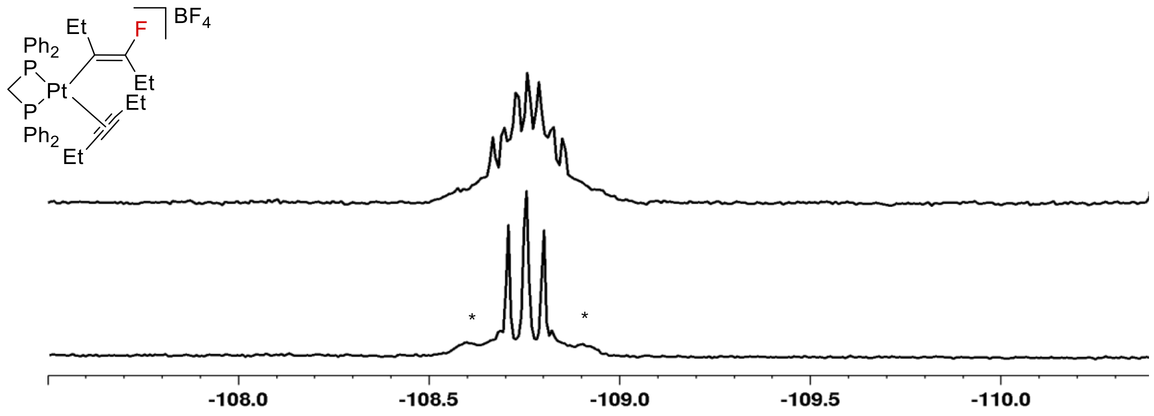

Figure S67. Excerpt of ^{19}F NMR (top) and $^{19}F\{^{1}H\}$ NMR (bottom) spectrum of [Pt(EtC=CFEt)(η^{2}-Et*C*≡*C*Et)(dppm)][BF_4] (**21-L1**). * depict ^{195}Pt satellites.

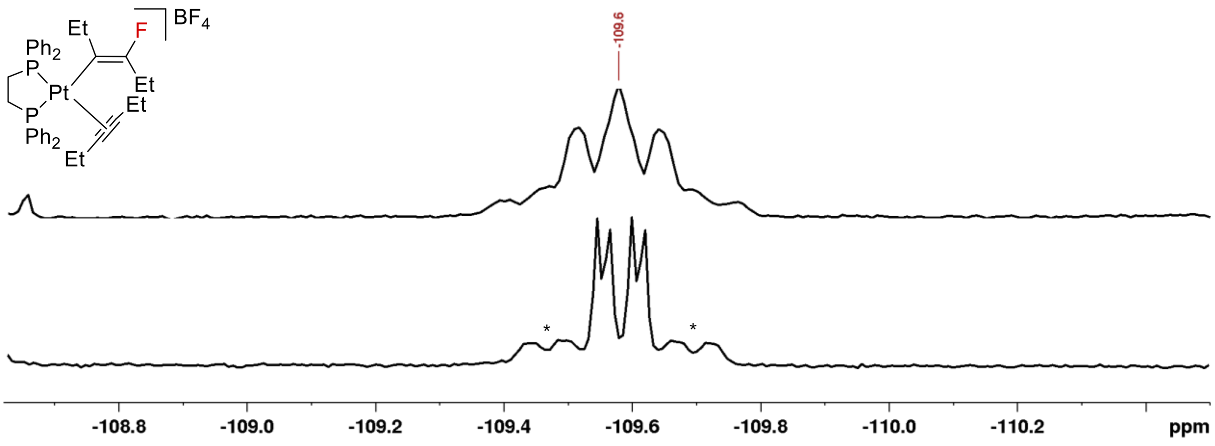

Figure S68. Excerpt of ^{19}F NMR (top) and $^{19}F\{^1H\}$ NMR (bottom) spectrum of [Pt(EtC=CFEt)(η^2-Et*C*≡*C*Et)(dppe)][BF_4] (**21-L2**). * depict ^{195}Pt satellites.

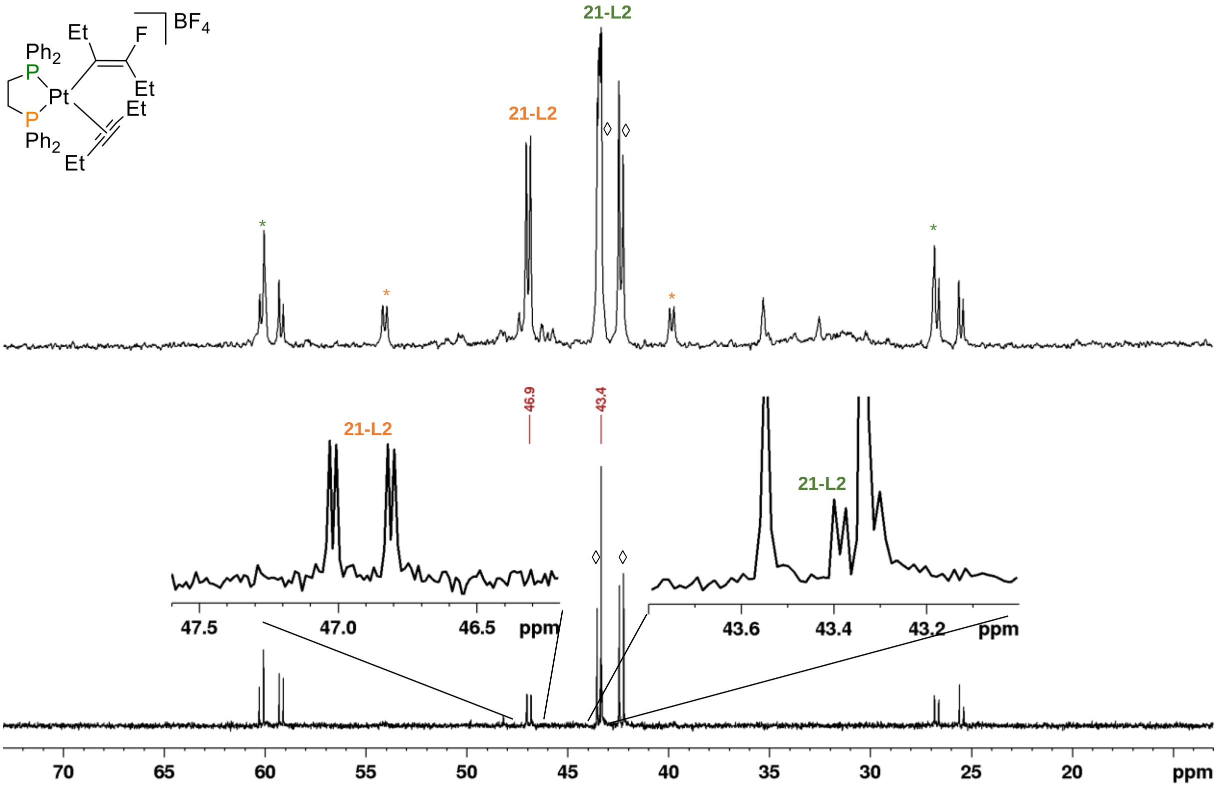

Figure S69. $^{31}P\{^1H\}$ NMR spectrum showing the formation of [Pt(EtC=CFEt)(η^2-Et*C*≡*C*Et)(dppe)][BF_4] (**21-L2**) besides [Pt(η^3-(C_2H_5*C*)$_3$*C*=C_2H_4)(dppe)][BF_4] (**20-L2**) ◊. Top spectrum: Higher concentration of **21-L2** allowing visible ^{195}Pt satellites (*). Bottom spectrum: higher resoluted signal of **21-L2**. * depict ^{195}Pt satellites.

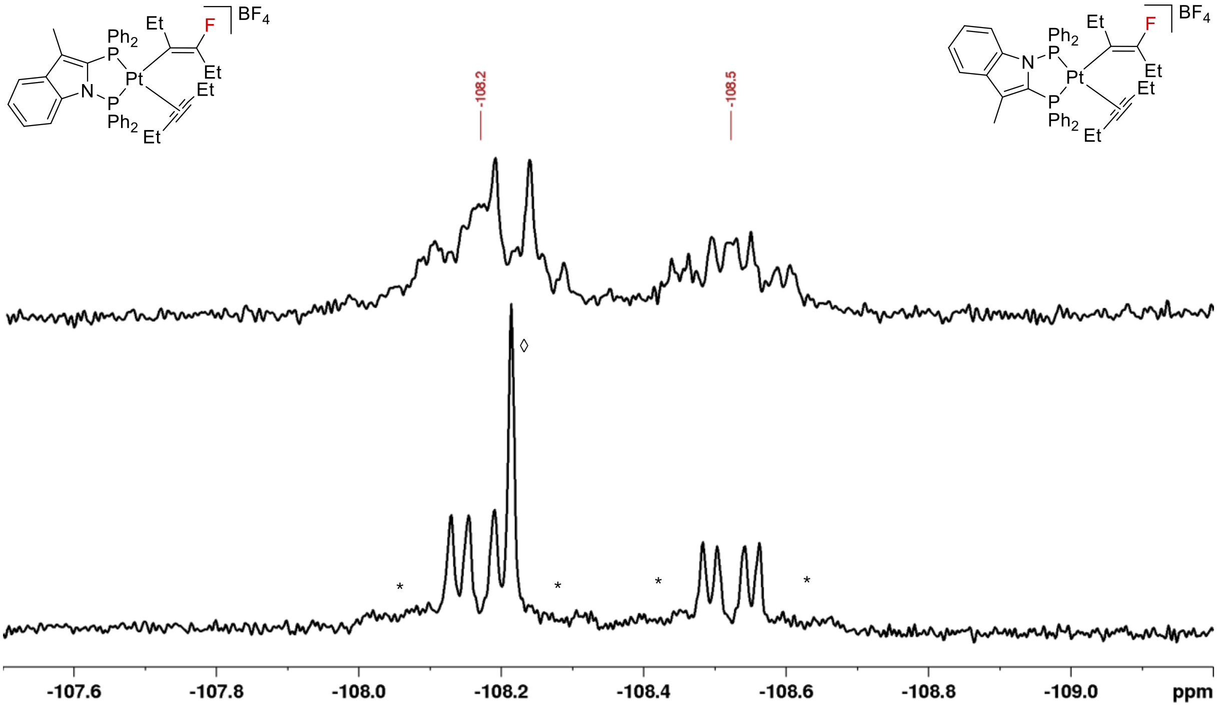

Figure S70. Excerpt of ^{19}F NMR (top) and ^{19}F{^{1}H} NMR (bottom) spectrum of both isomers of [Pt(EtC=CFEt)(η^2-Et*C*≡*C*Et)(PCNP)][BF$_4$] (**21-L3**) and (*E*)-3-fluoro-3-hexene ◊. * depict ^{195}Pt satellites.

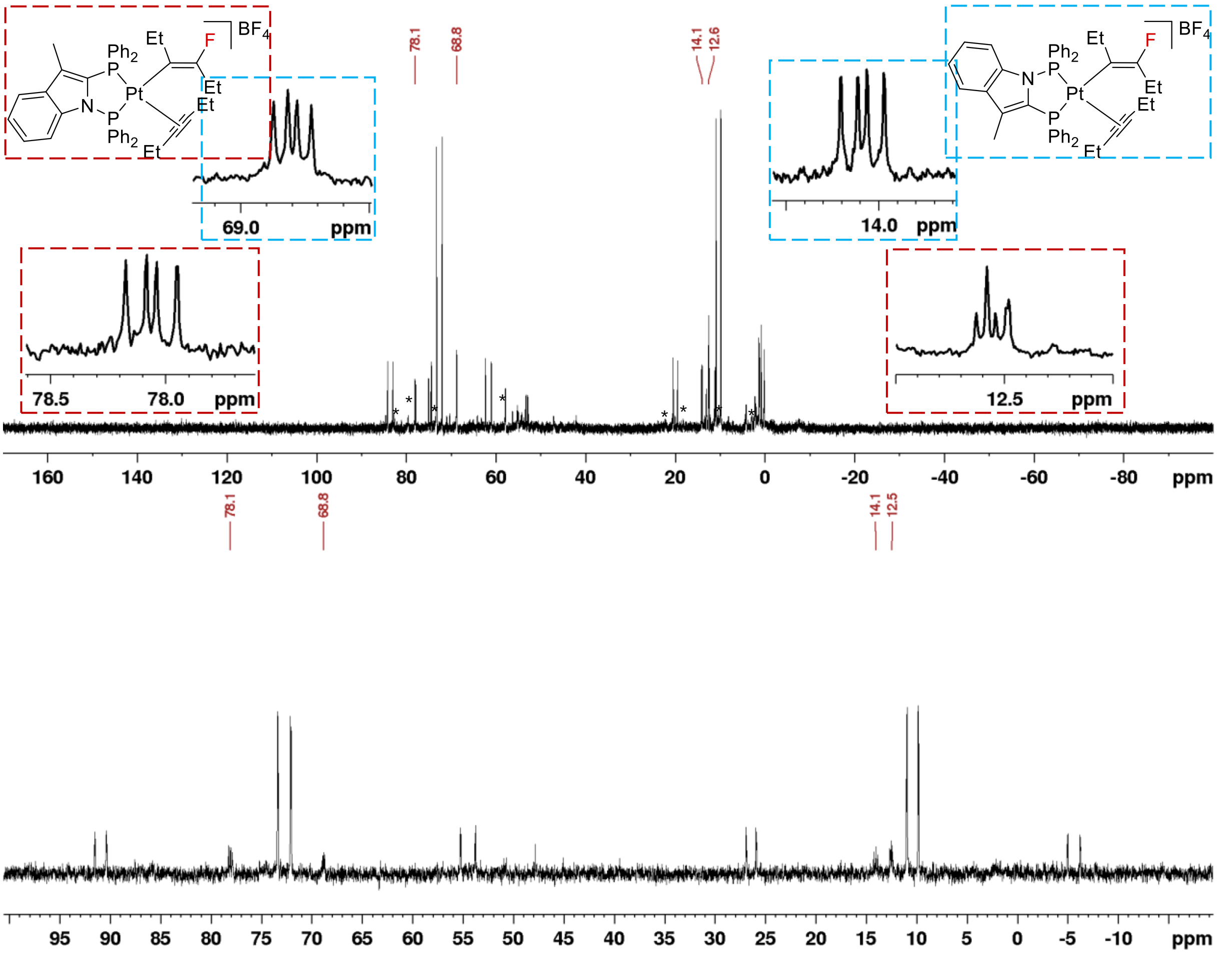

Figure S71. ^{31}P{^{1}H} NMR spectrum showing the formation of [Pt(EtC=CFEt)(η^2-Et*C*≡*C*Et)(PCNP)][BF$_4$] (**21-L3**) besides [Pt(η^3-(C$_2$H$_5$*C*)$_3$*C*=C$_2$H$_4$)(PCNP)][BF$_4$] (**20-L3**). Top spectrum: Higher concentration of **21-L3** allowing visible ^{195}Pt satellites (*) including zoom in of signals that belong each isomer. Bottom spectrum: Excerpt of ^{31}P{^{1}H} NMR spectrum signal of **21-L3**.

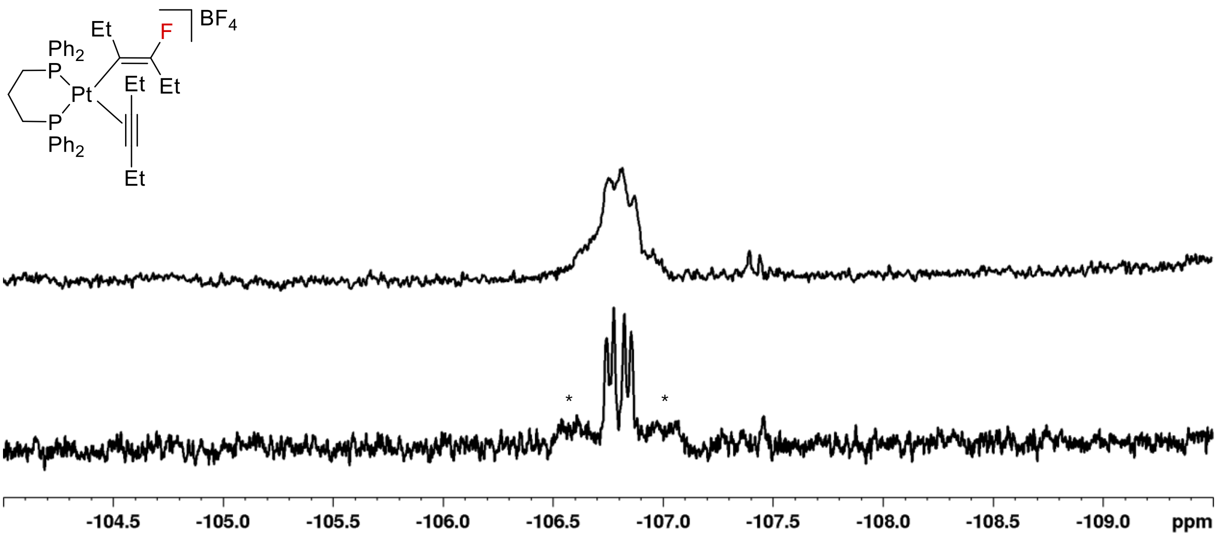

Figure S72. Excerpt of ^{19}F NMR (top) and $^{19}F\{^1H\}$ NMR (bottom) spectrum of $[Pt(EtC{=}CFEt)(\eta^2\text{-}Et\mathit{C}{\equiv}\mathit{C}Et)(dppp)][BF_4]$ (**21-L4**). * depict ^{195}Pt satellites.

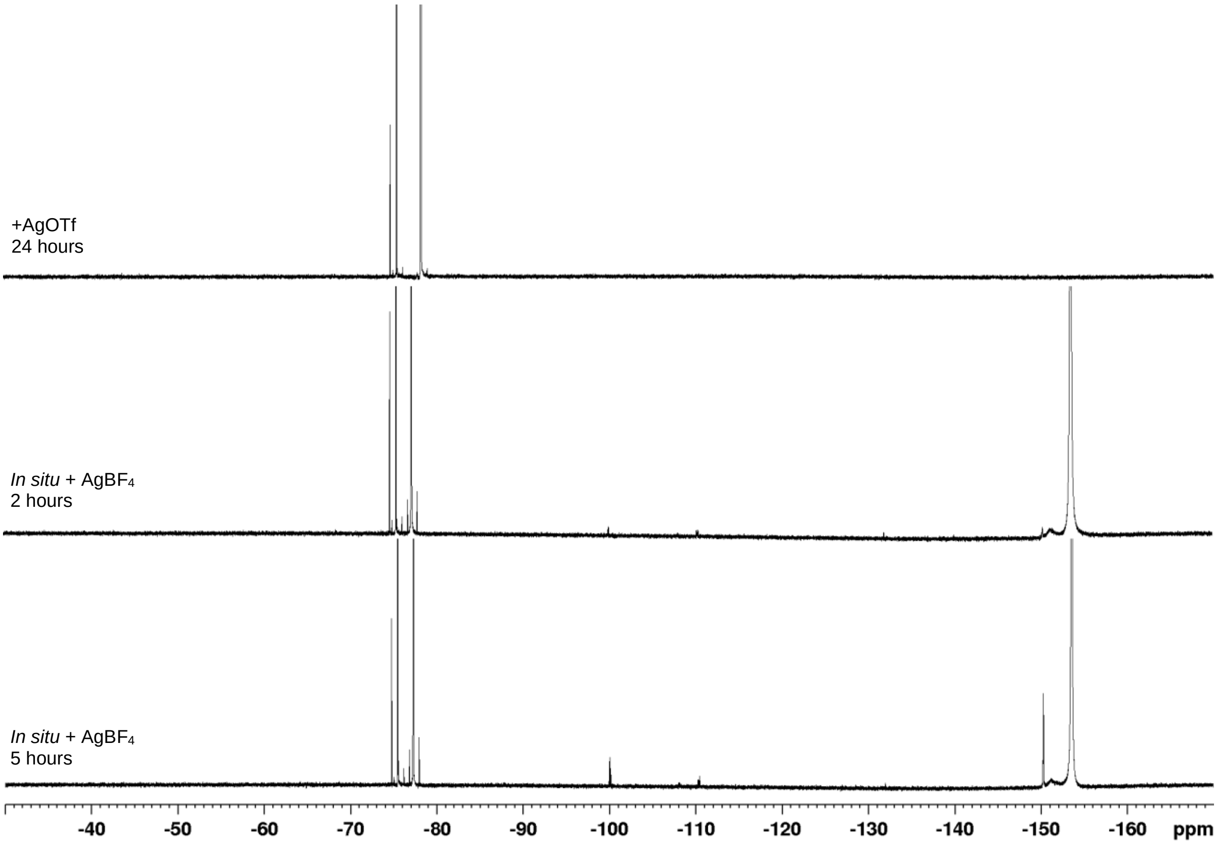

Figure S73. ^{19}F NMR spectrum of the model reaction I. (Figure S1A) showing the formation of fluoroalkenes and fluoroalkane upon *in situ* addition of AgBF4.

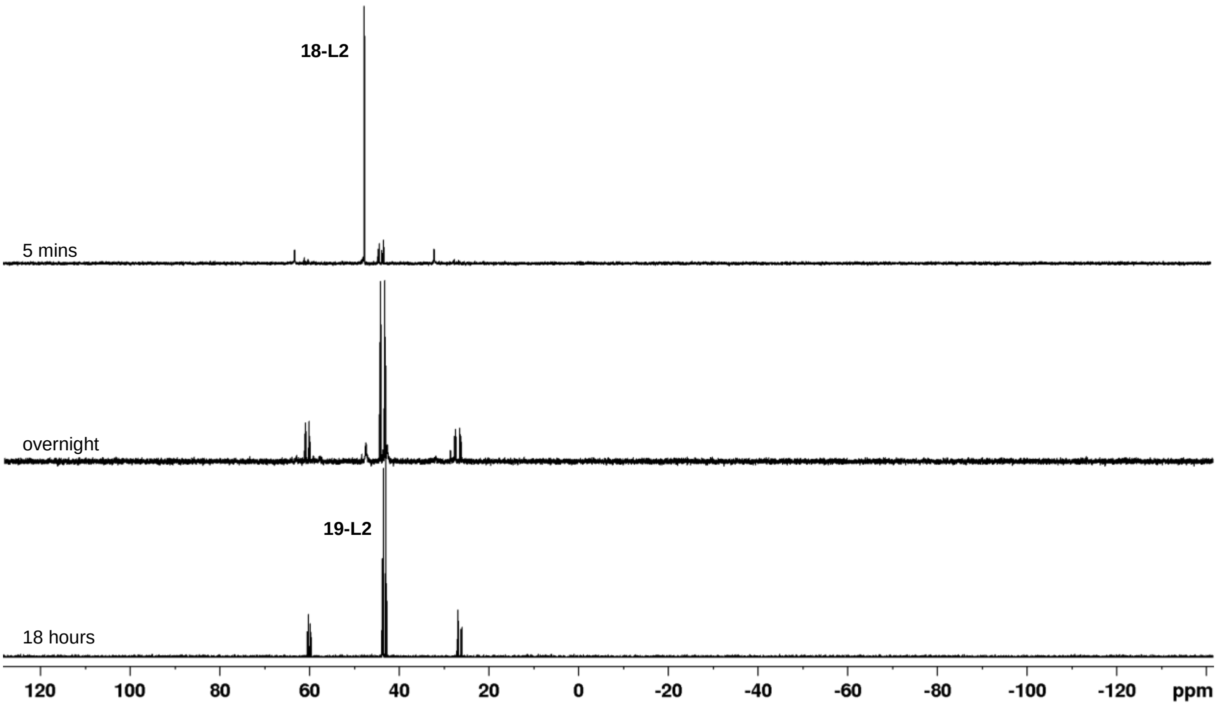

Figure S74. $^{31}P\{^{1}H\}$ NMR monitoring spectrum of the model reaction II (Scheme 2) at different time points showing the decrease of signal intensity of **18-L2** and increasing signal intensity for **20-L2** over time.

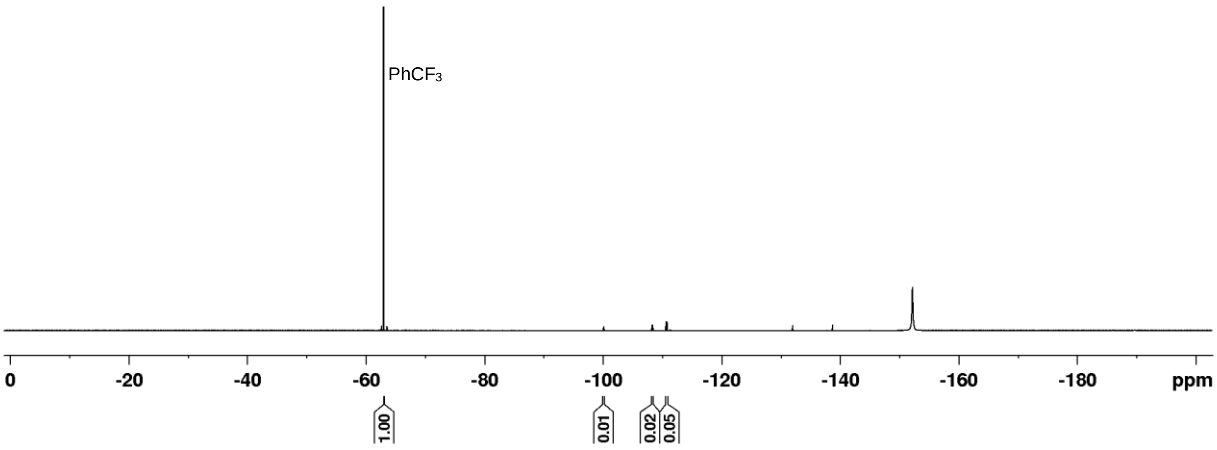

Figure S75. ^{19}F NMR spectrum (aq = 2 s, D1 = 30 s) of the model reaction II (Figure S1B) after reaction completion showing the formation of (*Z*)-, (*E*)-3-fluoro-3-hexene and 3,3-difluoroalkane.

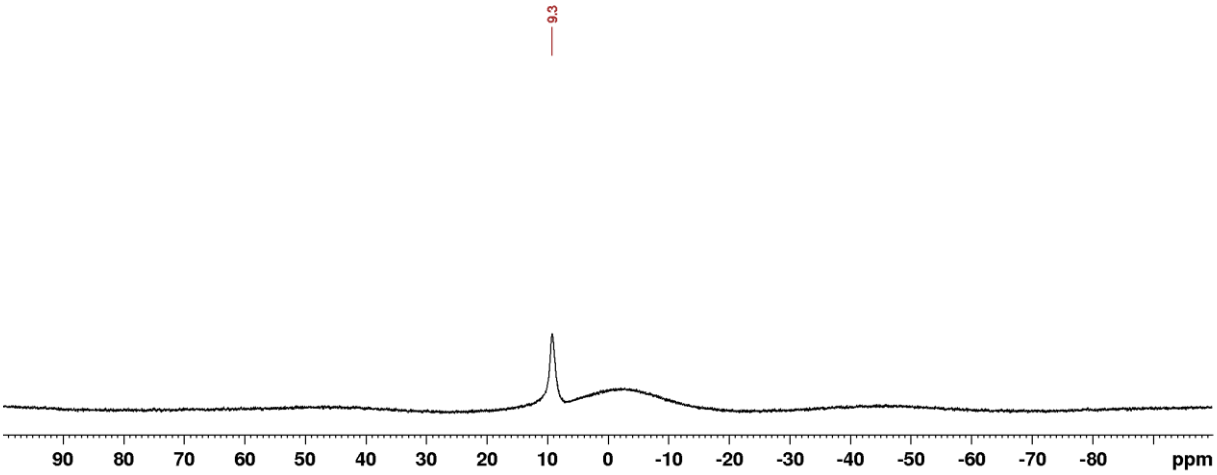

Figure S76. ^{11}B NMR spectrum after condensation of the model reaction III. (Figure S1B, condensation **1**) showing the formation of BF_3.

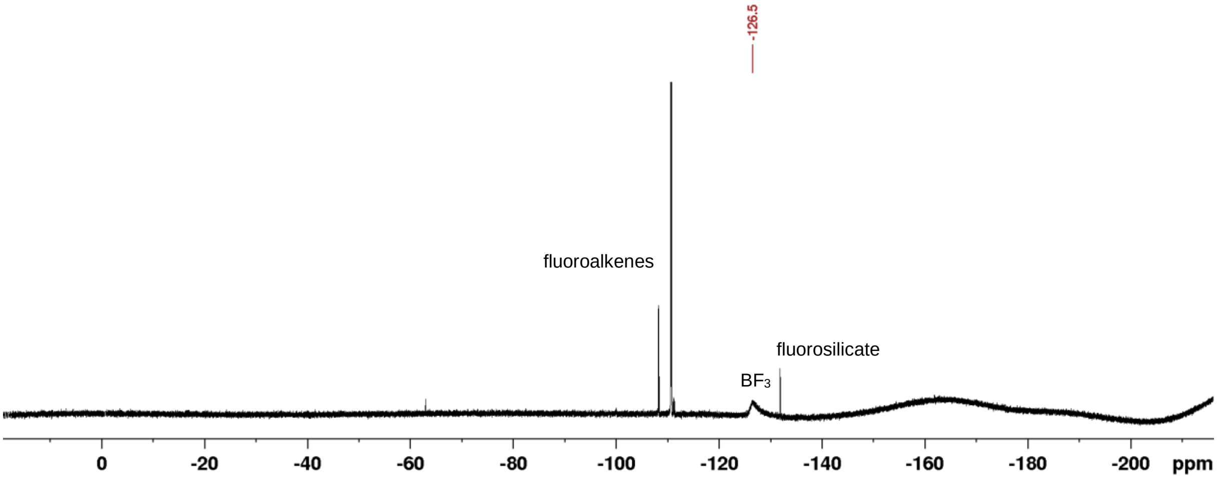

Figure S77. $^{19}F\{^1H\}$ NMR spectrum after condensation of the model reaction III. (Figure S1B, condensation **1**) showing the formation of BF_3.

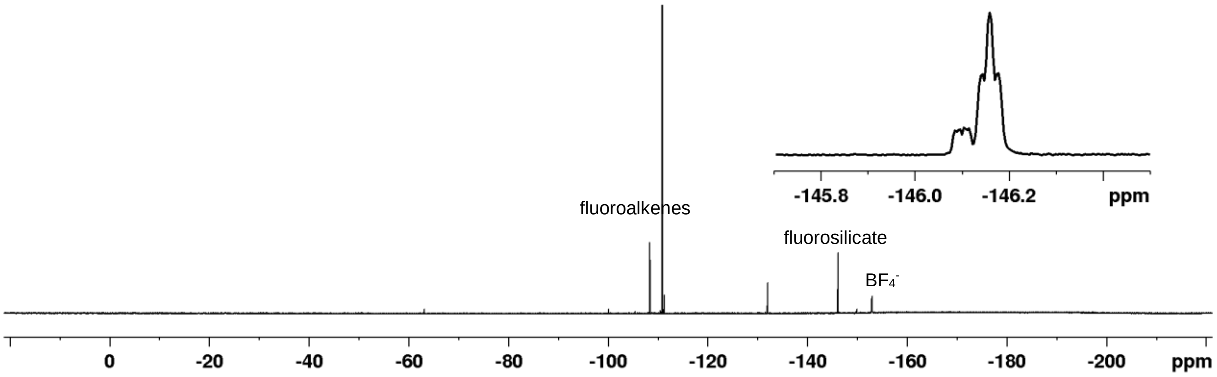

Figure S78. $^{19}F\{^1H\}$ NMR spectrum after condensation of the model reaction IV over $OPEt_3$ (Figure S1B, condensation **1**) showing the formation of $BF_3{\cdot}OPEt_3$.

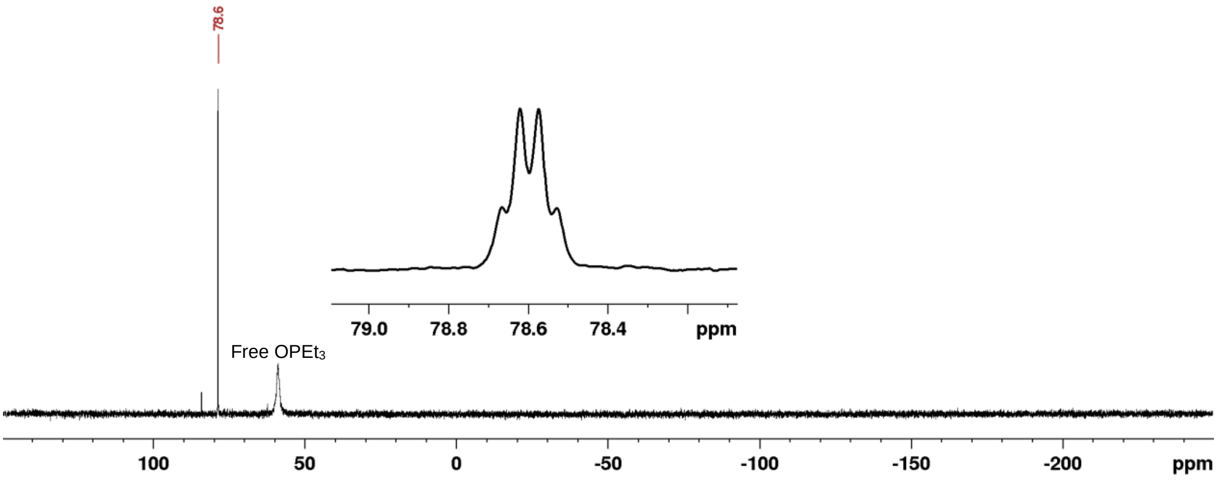

Figure S79. $^{31}P\{^{1}H\}$ NMR spectrum of the model reaction IV over $OPEt_3$ (Figure S1B, condensation **1**) showing the formation of $BF_3{\cdot}OPEt_3$.

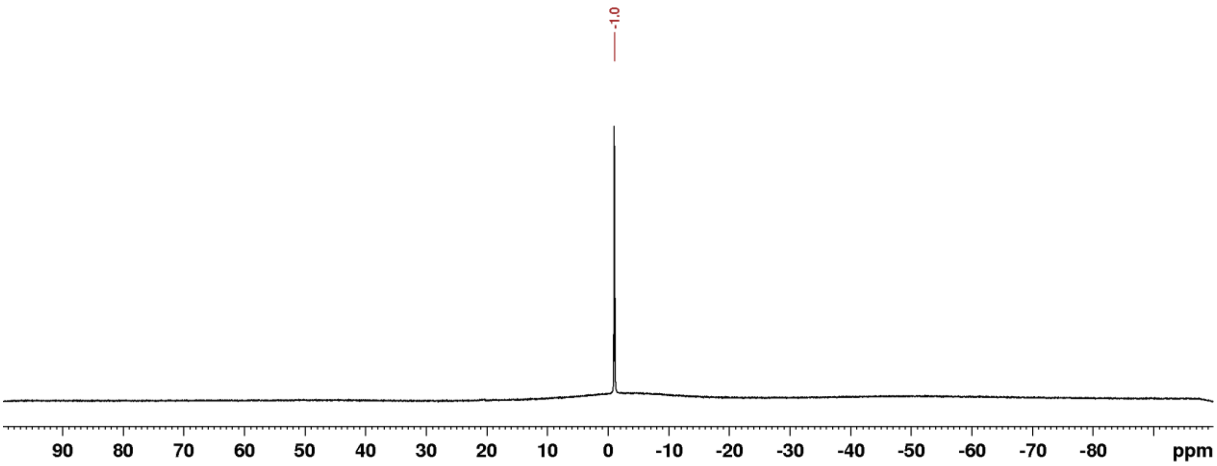

Figure S80. ^{11}B NMR spectrum of the model reaction IV over $OPEt_3$ (Figure S1B, condensation **B**) showing the formation of $BF_3{\cdot}OPEt_3$.

5 Computational Details

All DFT calculations were carried out using the TURBOMOLE program suite, version 7.7.1.[36-37] Molecular structures were optimised with the BP86[38-39] GGA (generalised gradient approximation) functional, including DFT-D3 dispersion corrections[40] with Becke-Johnson (BJ) damping[41] and using Ahlrichs' triple-ζ valence polarisation basis set (def2-TZVP)[42] for all atoms. The calculated electronic energies were refined at the ωB97M-V/def2-TZVP level.[43] Internal contributions to Gibbs free energies were calculated using the harmonic-oscillator/rigid rotor approximations at 298 K and 1 bar at the BP86-D3(BJ)/def2-TZVP level. This level was used also to identify minima and transition states. Transition-state searches were preceded by relaxed energy scans along pre-conceived reaction coordinate guesses. The nature of the transition states was further established by connecting them to minima using intrinsic reaction coordinate (IRC) scans. Initial conformational searches were performed at the tight-binding GFN2-xTB level with mquick settings to further reduce the computational costs using CREST version 2.12.[44] Non-covalent interactions were also considered during the CREST runs by using the nci settings.

Solvent effects were calculated using the implicit solvation model COSMO-RS (conductor like solvation model for real solvents)[45-46] v 2024.1. BP_TZVPD_FINE_24 parameters were used to generate the σ-profiles of the 1,2-dichloroethane solvent. The input files for COSMO-RS were generated *via* single point calculations at BP86-D3(BJ)/def2-TZVPD level of theory, setting an infinite permittivity and using the refined COSMO cavity construction algorithm. Gibbs free energies of solvation ΔG_{solu} were calculated in a standard way for infinite dilution and a reference state of 1 mol/L.

Natural atomic charges were calculated using the NBO[47] module implemented in TURBOMOLE.

Formation of [Pt(*C*,*C*-η^2-C_2H_5*C*≡*C*C_2H_5)$_2$(dppe)][BF_4]$_2$ (Int-0, 19-L2) from [$PtCl_2$(dppe)] in the presence of $AgBF_4$ and the alkyne substrate 3-hexyne

The complex for the hydrofluorination cycle and starting point for cycloaddition is the complex [Pt(*C*,*C*-η^2-C_2H_5*C*≡*C*C_2H_5)$_2$(dppe)][BF_4]$_2$ (**Int-0, 19-L2**). It is generated from the reaction of [$PtCl_2$(dppe)] and $AgBF_4$, resulting first in the binuclear complex [PtCl(dppe)]$_2$[BF_4]$_2$ and AgCl. The BF_4^- counter ions in the [PtCl(dppe)]$_2$[BF_4]$_2$ dimer coordinate axially to the platinum centre, with coordinating Pt–F distances of 3.2 Å and 3.3 Å. This coordination stabilises the dimer complex significantly (see Figure S81). The computed Gibbs free energy for formation of [PtCl(dppe)]$_2$[BF_4]$_2$ from [$PtCl_2$(dppe)] is significantly negative in the gas phase, made somewhat less negative by solvation. In the second step, the bridging chlorido ligands in the [PtCl(dppe)]$_2$[BF_4]$_2$ dimer are also abstracted by the silver salt in the presence of the alkyne substrate, forming the Pt bis-alkyne η^2-complex **Int-0** coordinated by two counter anions. By including the two anions, the Pt species becomes overall neutral. This step is somewhat endergonic in the gas phase, but made almost thermoneutral by including solvation contributions.

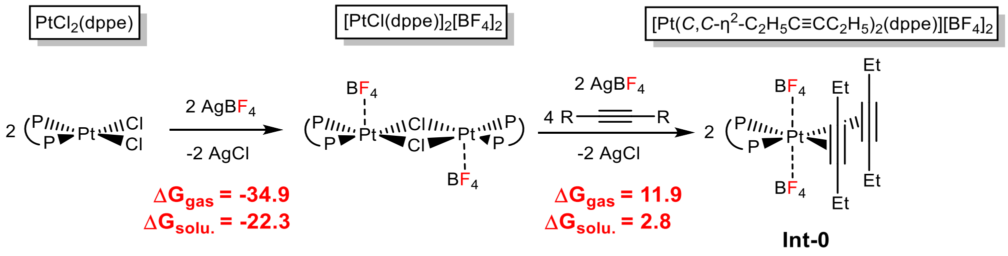

Figure S81. Formation of the hydrofluorination catalyst [Pt(*C*,*C*-η^2-C_2H_5*C*≡*C*C_2H_5)$_2$(dppe)][BF_4]$_2$ (**Int-0**, **19-L2**) from the [$PtCl_2$(dppe)] (**1-L2**) precursor in the presence of $AgBF_4$ and alkyne substrate (3-hexyne). All free energies (in kcal/mol) have been obtained computationally, with solvent effects for 1,2 dichloroethane included using COSMO-RS.

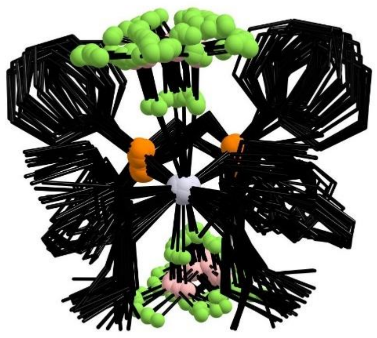

Figure S82. Conformer-rotamer ensemble of [Pt(C,C-η^2-$C_2H_5C{\equiv}CC_2H_5$)$_2$(dppe)][BF_4]$_2$ (**Int-0**) generated at GFN2-xTB level using CREST, showing the lowest-lying conformers within a 6 kcal/mol energy window. Hydrogen atoms of the dppe ligand (**L2**) and 3-hexyne substrates are omitted for clarity. The carbon atoms are shown as black sticks.

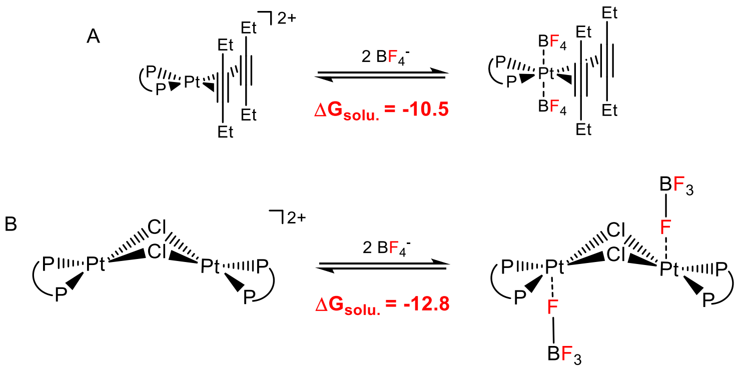

Figure S83. Gibbs free energies in kcal/mol for coordination of explicit BF_4^- anions to [PtCl(dppe)]$_2$[BF_4]$_2$ (A) and [Pt(C,C-η^2-$C_2H_5C{\equiv}CC_2H_5$)$_2$(dppe)][BF_4]$_2$ (B) in solution. Solvent effects were included at COSMO-RS level.

Table S4. Structural details and NPA atomic charges of intermediates and transition states along the hydrofluorination reaction cycle. Bond lengths (black) in Å, and NPA charges (blue). (P P = dppe.

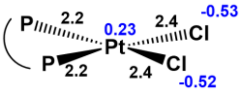

1-L2

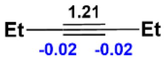

3-Hexyne

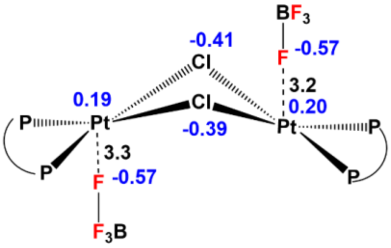

18-L2

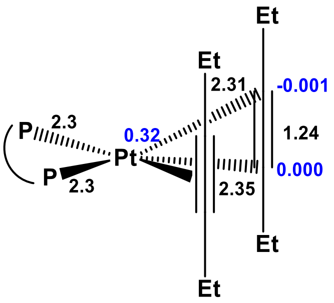

Int-0 without BF_4^-

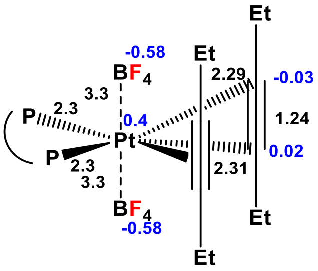

Int-0

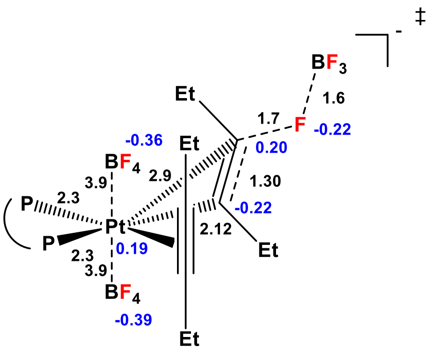

TS-1'

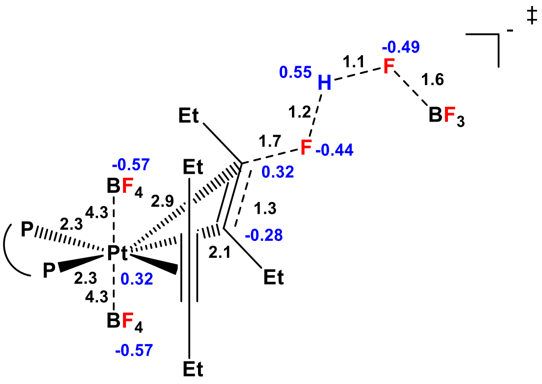

TS-1

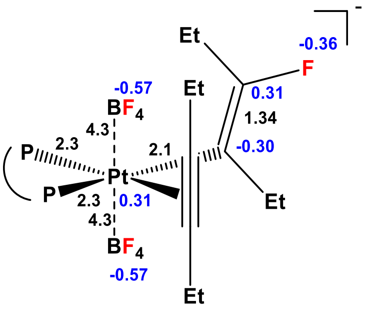

Int-1

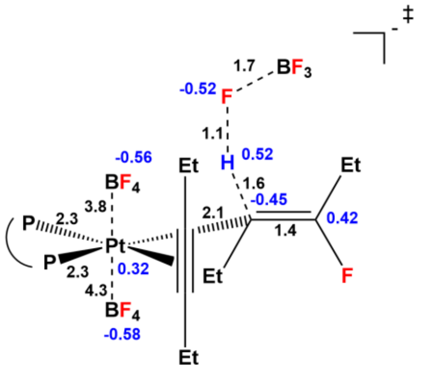

TS-2’

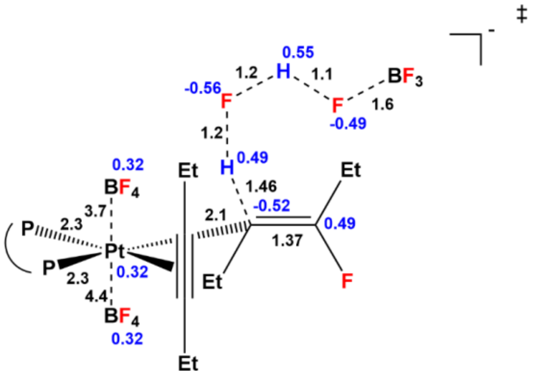

TS-2

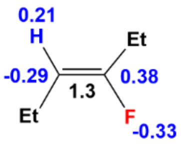

(*Z*)-3-fluoro-3-hexene (2a)

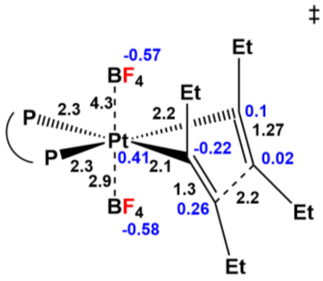

TS-3

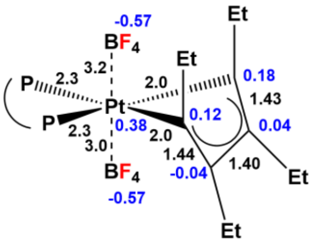

Int-2

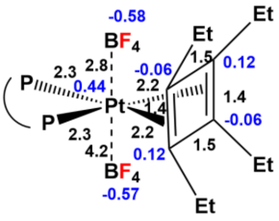

TS-4

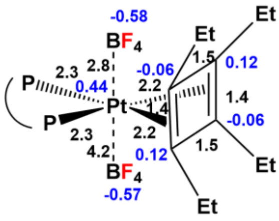

Int-3

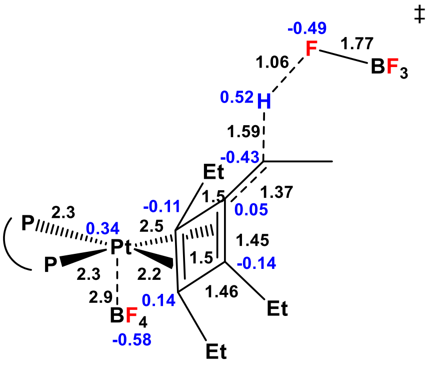

TS-5

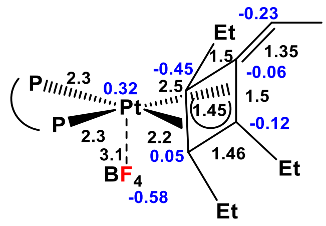 Et<br>-0.23<br>1.35<br>-0.45<br>1.5<br>-0.06<br>P<br>2.3<br>0.32<br>2.5<br>Pt<br>1.5<br>1.45<br>P<br>-0.12<br>2.3<br>2.2<br>3.1<br>0.05<br>1.46<br>BF4<br>-0.58<br>Et<br>Et<br>**Int-4 (20-L2)**	

8 Cartesian coordinates

3-Hexyne

Thermal correction = 279.93 kJ/mol

Solvation energy = -1.62 kJ/mol

C	-1.3410386	-0.0113143	2.8349766
C	-0.1592449	0.2566515	2.7559262
C	-2.7639844	-0.3152628	2.9490221
H	-3.2922239	0.0857496	2.0675845
H	-2.9072504	-1.4084313	2.9152527
C	1.2631348	0.5758892	2.6847586
H	1.3961797	1.5236246	2.1363301
H	1.7787391	-0.1940367	2.0863306
C	-3.4007726	0.2486117	4.2316727
H	-4.4699981	-0.0019643	4.2733449
H	-2.9120937	-0.1665046	5.1230707
H	-3.2986873	1.3413038	4.2680778
C	1.9281905	0.6838237	4.0684650
H	1.8411299	-0.2636785	4.6165350
H	2.9945827	0.9287305	3.9653134
H	1.4488491	1.4675947	4.6698841

HF

Thermal correction = -19.49 kJ/mol

Solvation energy = 9.17 kJ/mol

F	0.0137826	0.0002712	-0.0000105
H	0.9460314	0.0186158	-0.0007195

BF_4^-

Thermal correction= -37.07 kJ/mol

Solvation energy = -211.48 kJ/mol

B	0.0029192	-0.0039778	0.0012557
F	-1.4192938	0.0012589	0.0038528
F	0.4818334	1.3350202	-0.0249245
F	0.4770742	-0.6541771	1.1740375
F	0.4721120	-0.6982003	-1.1480126

BF_3

Thermal correction = -37.34 kJ/mol

Solvation energy = -215.42 kJ/mol

B	-0.0142122	-0.0498326	-0.0006180
F	-1.4299525	-0.0180756	0.0031801
F	0.4728984	-0.6693326	1.1759646
F	0.4652542	-0.7213702	-1.1515448

(*Z*)-3-fluoro-3-hexene (2a)

Thermal correction = 313.4 kJ/mol

Solvation energy = -1.11 kJ/mol

C	0.5877614	-1.5981992	1.7998541
C	0.3217012	-1.3221704	3.0782240
C	1.9451992	-1.8650255	1.2201995
H	2.1488494	-1.1289652	0.4234545
H	2.7132525	-1.7140126	1.9908357
C	-0.9889373	-1.0638111	3.7470162
H	-0.9338105	-0.0725321	4.2293549
H	-1.0945490	-1.7867985	4.5742485
C	2.0616619	-3.2802147	0.6326172
H	3.0524342	-3.4413152	0.1843631
H	1.9103563	-4.0398397	1.4122910
H	1.3065442	-3.4490388	-0.1493504
C	-2.2056396	-1.1352653	2.8261320
H	-2.3020766	-2.1291839	2.3673572
H	-3.1246192	-0.9358133	3.3927746
H	-2.1404233	-0.3927610	2.0186636
F	1.3568368	-1.2437808	3.9806939
H	-0.2695245	-1.6547558	1.1262503

$PtCl_2$(dppe)

Thermal correction = 918.65 kJ/mol

Solvation energy = -118.33 kJ/mol

C	-0.4234659	1.8429149	-2.4340824
C	-0.8817377	0.4699244	-2.9447029

H	-0.7313438	2.6583485	-3.1031431
H	0.6708689	1.8846628	-2.3441006
H	-1.8436678	0.5577740	-3.4626995
H	-0.1637509	0.0466167	-3.6578259
Pt	-0.5102679	0.2409835	0.3827274
P	-1.0453132	2.0969772	-0.7134686
P	-1.0538214	-0.7538968	-1.5389400
C	-0.4219069	3.7189272	-0.2075359
C	0.8939972	3.8014517	0.2730617
C	-1.2071671	4.8746198	-0.3078352
C	1.4255579	5.0411743	0.6231538
H	1.4771779	2.8903111	0.4067839
C	-0.6699323	6.1120509	0.0514225
H	-2.2398210	4.8057628	-0.6506571
C	0.6460153	6.1963594	0.5115153
H	2.4446455	5.1024567	1.0056952
H	-1.2843840	7.0100660	-0.0210229
H	1.0614119	7.1631940	0.7981893
C	-2.8533210	2.2225235	-0.9018066
C	-3.6565849	1.6552883	0.1003149
C	-3.4556152	2.7668738	-2.0453932
C	-5.0420714	1.6282943	-0.0464659
H	-3.1781486	1.2123586	0.9753142
C	-4.8437123	2.7355260	-2.1896380
H	-2.8444175	3.2099936	-2.8329513
C	-5.6364668	2.1621458	-1.1925870
H	-5.6571463	1.1711761	0.7284865
H	-5.3055391	3.1535866	-3.0848998
H	-6.7197290	2.1252610	-1.3129217
C	-0.1403651	-2.2190722	-2.0987191
C	-0.7400248	-3.1500355	-2.9579912
C	1.1957948	-2.3802654	-1.7075742
C	0.0018188	-4.2304525	-3.4373612
H	-1.7885848	-3.0378603	-3.2374903
C	1.9317978	-3.4612678	-2.1923605
H	1.6350413	-1.6785594	-0.9986544
C	1.3378511	-4.3841647	-3.0577003
H	-0.4669905	-4.9578674	-4.1011111
H	2.9674121	-3.5934657	-1.8777581
H	1.9139354	-5.2334846	-3.4271254
C	-2.8065539	-1.2657769	-1.4829438
C	-3.7707126	-0.9025267	-2.4303650
C	-3.1920104	-2.0147350	-0.3563982
C	-5.1060524	-1.2713149	-2.2528730
H	-3.5031765	-0.3157391	-3.3078512
C	-4.5239595	-2.3865524	-0.1893851
H	-2.4419092	-2.2786415	0.3933399
C	-5.4854602	-2.0117159	-1.1331958
H	-5.8500337	-0.9697378	-2.9907593
H	-4.8138260	-2.9617959	0.6906000
H	-6.5298134	-2.2932820	-0.9927885
Cl	-0.0479729	-1.8365580	1.4163769
Cl	-0.1484723	1.3964790	2.4033559

$[PtCl(dppe)]_2[BF_4]_2$

Thermal correction = 1959.54 kJ/mol

Solvation energy = -210.72 kJ/mol

C	-1.4654390	3.7184959	3.1981831
C	-2.4915917	2.6136901	3.4591856
H	-1.0981620	4.1620444	4.1336186
H	-1.9093535	4.5047622	2.5706358
H	-2.1175140	1.8683762	4.1763078
H	-3.4303343	3.0296344	3.8467782
Pt	-0.9440324	1.6525750	0.7008704
Cl	-1.7876679	0.1629493	-1.0602278
P	-0.0726281	2.9434186	2.2997398
P	-2.8405834	1.7062272	1.8837063
C	2.5538118	-3.1444961	-0.2442360
C	1.2477646	-3.8623333	-0.6399279
H	3.4420875	-3.6568155	-0.6352948
H	2.6316453	-3.1189202	0.8519054
H	1.4247412	-4.7717930	-1.2287777
H	0.7012998	-4.1630495	0.2664323
Pt	0.5018564	-0.6554662	-0.9996043
Cl	1.0941110	1.6656778	-0.6210021
P	2.5923076	-1.3936591	-0.8168157
P	0.0866450	-2.7729498	-1.5513448
C	3.4424906	-1.3709804	-2.4241796
C	2.9097198	-0.6077176	-3.4726518

C	4.6112849	-2.1184070	-2.6319768
C	3.5307161	-0.6058365	-4.7207390
H	1.9947047	-0.0383807	-3.3044480
C	5.2271677	-2.1164764	-3.8831878
H	5.0476296	-2.6986618	-1.8175700
C	4.6847287	-1.3640747	-4.9291122
H	3.1039563	-0.0193201	-5.5341022
H	6.1329839	-2.7024033	-4.0418005
H	5.1644330	-1.3696685	-5.9084203
C	3.6153344	-0.4328011	0.3203060
C	4.5526040	0.4834263	-0.1808920
C	3.3718601	-0.5144775	1.7007306
C	5.2620312	1.2988705	0.6997118
H	4.7226604	0.5597338	-1.2545069
C	4.0857487	0.3084366	2.5708804
H	2.6300165	-1.2085388	2.0976689
C	5.0303308	1.2109917	2.0747745
H	5.9936646	2.0064208	0.3087208
H	3.8866123	0.2523572	3.6404346
H	5.5786040	1.8557020	2.7625512
C	0.4525193	-2.9104235	-3.3274196
C	-0.2664414	-2.0821804	-4.2070568
C	1.4935987	-3.7044054	-3.8228831
C	0.0486155	-2.0632817	-5.5632022
H	-1.0613088	-1.4424300	-3.8200426
C	1.8136406	-3.6736347	-5.1816183
H	2.0752035	-4.3360289	-3.1528540
C	1.0924023	-2.8562153	-6.0519785
H	-0.5139351	-1.4190340	-6.2393844
H	2.6353439	-4.2842346	-5.5557036
H	1.3468973	-2.8296149	-7.1118875
C	-1.5894907	-3.3870221	-1.3089012
C	-2.2446856	-4.1129979	-2.3152237
C	-2.2269741	-3.1276447	-0.0847049
C	-3.5401813	-4.5806912	-2.0956089
H	-1.7463318	-4.3071369	-3.2647468
C	-3.5192000	-3.6030422	0.1219240
H	-1.7115218	-2.5654756	0.6942775
C	-4.1765276	-4.3232394	-0.8791692
H	-4.0516076	-5.1440074	-2.8766900
H	-4.0135979	-3.3981031	1.0693584
H	-5.1918461	-4.6844796	-0.7105244
C	-4.1917994	2.5640838	1.0525680
C	-4.0905338	2.9020369	-0.3016033
C	-5.3403801	2.9155251	1.7808098
C	-5.1333762	3.5861994	-0.9235497
H	-3.1788070	2.6783282	-0.8510704
C	-6.3797166	3.5933931	1.1507853
H	-5.4227697	2.6697416	2.8404396
C	-6.2750049	3.9306352	-0.2013254
H	-5.0311003	3.8868296	-1.9651102
H	-7.2628176	3.8799791	1.7222444
H	-7.0768941	4.4885660	-0.6853369
C	-3.4776752	0.0805566	2.3928962
C	-2.6247089	-0.7961704	3.0823493
C	-4.8059762	-0.2962494	2.1443008
C	-3.1034467	-2.0157806	3.5571695
H	-1.5784238	-0.5420042	3.2407643
C	-5.2857526	-1.5119104	2.6345314
H	-5.4633604	0.3637566	1.5802930
C	-4.4414512	-2.3650405	3.3499178
H	-2.4153781	-2.6870768	4.0697561
H	-6.3235960	-1.7927291	2.4507939
H	-4.8201649	-3.3141992	3.7319627
C	1.1485949	4.1617223	1.7781173
C	2.4771465	3.7242381	1.6258625
C	0.7968210	5.4831130	1.4724612
C	3.4492832	4.6130905	1.1780452
H	2.7455804	2.6931513	1.8538447
C	1.7846201	6.3663616	1.0320506
H	-0.2319718	5.8337725	1.5388218
C	3.1046231	5.9367622	0.8829416
H	4.4768744	4.2691304	1.0568027
H	1.5036479	7.3918792	0.7926688
H	3.8679685	6.6320791	0.5306501
C	0.7801446	1.9569858	3.5681713
C	0.7545114	0.5587394	3.5470172
C	1.4065668	2.6294022	4.6321088
C	1.3110592	-0.1690411	4.6031026
H	0.3172495	0.0331767	2.6968241

C	1.9770178	1.9012884	5.6738566
H	1.4536541	3.7196656	4.6382020
C	1.9167641	0.5023534	5.6657797
H	1.2609428	-1.2570634	4.5856290
H	2.4623317	2.4256639	6.4979607
H	2.3501839	-0.0656895	6.4900474
B	0.4682243	-3.1954491	2.7500914
F	-0.0371622	-4.3758341	2.1695164
F	1.8850206	-3.2649681	2.8531121
F	0.1537825	-2.0888267	1.8861886
F	-0.0976497	-2.9773014	4.0163579
B	-2.7212865	5.9295775	0.5543005
F	-3.5658368	6.2739139	-0.5008043
F	-3.4756116	5.5421654	1.7043570
F	-1.8959035	4.8092917	0.1816431
F	-1.8720967	7.0057042	0.9111962

Int-0 (19-L2)

Thermal correction = 1641.69 kJ/mol

Solvation energy = -133.06 kJ/mol

C	-0.5659985	0.0158409	-2.6885048
C	0.9550872	-0.1708065	-2.6082286
H	-1.0874531	-0.9522616	-2.6634462
H	-0.8613934	0.5604441	-3.5945829
H	1.3396071	-0.7385758	-3.4651258
H	1.4755297	0.7966402	-2.5541752
Pt	0.0463579	-0.0041994	0.5755083
P	-1.0665845	0.9691334	-1.1991666
P	1.2940329	-1.0765546	-1.0466324
C	-0.5688944	2.6788228	-1.5513614
C	-1.0894538	3.2905938	-2.7068061
C	0.2588953	3.4029953	-0.6937306
C	-0.7608179	4.6116752	-2.9966294
H	-1.7670437	2.7441971	-3.3650708
C	0.5867705	4.7280365	-0.9876846
H	0.6933235	2.9243748	0.1759097
C	0.0788790	5.3309882	-2.1368952
H	-1.1611115	5.0831898	-3.8947558
H	1.2696548	5.2611527	-0.3283613
H	0.3428519	6.3621994	-2.3737334
C	-2.8692322	1.1231864	-1.1485718
C	-3.3999203	2.2158089	-0.4387519
C	-3.7280687	0.2082552	-1.7702736
C	-4.7791796	2.3748863	-0.3402774
H	-2.7307058	2.9373817	0.0294557
C	-5.1088531	0.3811186	-1.6732688
H	-3.3310640	-0.6611350	-2.2866288
C	-5.6351252	1.4553107	-0.9550533
H	-5.1875408	3.2196582	0.2152004
H	-5.7711503	-0.3479625	-2.1381408
H	-6.7154674	1.5762627	-0.8704066
C	3.0790000	-1.2771305	-0.8202094
C	3.5043435	-2.3407354	-0.0034806
C	4.0223840	-0.4312126	-1.4167481
C	4.8629381	-2.5374839	0.2269306
H	2.7704426	-3.0125667	0.4415206
C	5.3820607	-0.6423298	-1.1868599
H	3.7044272	0.4161326	-2.0173940
C	5.8031995	-1.6858226	-0.3620198
H	5.1894279	-3.3595429	0.8645874
H	6.1090946	0.0341249	-1.6343326
H	6.8667784	-1.8363409	-0.1745510
C	0.7793975	-2.7864270	-1.3724791
C	-0.0478779	-3.4899562	-0.4961114
C	1.3050871	-3.4285291	-2.5083641
C	-0.3722491	-4.8225838	-0.7562132
H	-0.4826349	-2.9912272	0.3622384
C	0.9788350	-4.7572898	-2.7646447
H	1.9843944	-2.9006557	-3.1795754
C	0.1375999	-5.4544212	-1.8890576
H	-1.0559984	-5.3406316	-0.0860109
H	1.3821549	-5.2514010	-3.6491843
H	-0.1265847	-6.4910474	-2.1004319
C	-1.6936999	0.6119215	1.9698757
C	-0.7718564	1.4172457	2.1824301
C	-2.9508206	-0.1312372	1.9737751
H	-3.3322301	-0.2294485	0.9499448
H	-2.7746104	-1.1661443	2.2991401
C	0.0541007	2.4937438	2.7415132

H	0.9764920	2.6296496	2.1586202
H	0.3713736	2.1852201	3.7512308
C	-3.9931607	0.5530071	2.8706552
H	-4.9214719	-0.0307865	2.8388268
H	-3.6507098	0.6131455	3.9126007
H	-4.2080075	1.5686520	2.5146070
C	-0.7194720	3.8209281	2.7987989
H	-1.6374461	3.7220267	3.3928395
H	-0.0871072	4.5958364	3.2507774
H	-0.9919533	4.1500227	1.7877115
C	0.7452497	-1.3210966	2.3337576
C	1.6691136	-0.5131427	2.1406827
C	-0.1566440	-2.3126844	2.9300356
H	-1.0218931	-2.5031728	2.2780226
H	0.3894653	-3.2693194	2.9827588
C	2.9227620	0.2350243	2.1798500
H	2.7231282	1.2888038	2.4194914
H	3.3715530	0.2636523	1.1787271
C	-0.6345172	-1.8875034	4.3275068
H	-1.3147032	-2.6478424	4.7327198
H	0.2115429	-1.7666400	5.0165374
H	-1.1717082	-0.9318933	4.2767334
C	3.9054246	-0.3796117	3.1878156
H	4.1460547	-1.4153742	2.9163340
H	4.8325410	0.2068860	3.1769902
H	3.4948102	-0.3710132	4.2064331
F	2.5744089	1.8261135	-0.5821781
F	-2.3617100	-1.9203184	-0.5773157
B	-3.2770730	-2.8752356	0.0120930
B	3.4494005	2.8111171	0.0223971
F	-2.6939109	-3.3066543	1.2475677
F	-3.4329505	-3.9554841	-0.8547760
F	-4.4983085	-2.2287542	0.2661595
F	2.7732427	3.3167414	1.1802660
F	4.6397802	2.1744065	0.4086981
F	3.6805797	3.8376091	-0.8912842

TS-1

Thermal correction = 1677.47 kJ/mol

Solvation energy = -252.87 kJ/mol

C	0.0316414	3.0954040	-0.9608525
C	1.0417689	3.0135655	0.1921611
H	0.5100368	2.8890067	-1.9312881
H	-0.4477931	4.0824319	-1.0092670
H	1.8757747	3.7153327	0.0581256
H	0.5571413	3.2275758	1.1560782
Pt	-0.0042076	-0.1122521	-0.1666699
P	-1.2318694	1.7892889	-0.6322426
P	1.7195044	1.2880215	0.2417728
C	-2.2246911	2.4980398	0.7152600
C	-3.1293061	3.5400119	0.4425169
C	-2.0630631	2.0413783	2.0282074
C	-3.8667630	4.1055657	1.4798495
H	-3.2664621	3.8892923	-0.5819720
C	-2.8078151	2.6080615	3.0671930
H	-1.3712244	1.2310460	2.2587944
C	-3.7083618	3.6368000	2.7900883
H	-4.5720175	4.9102332	1.2654800
H	-2.6748048	2.2334630	4.0816909
H	-4.2939661	4.0761461	3.5990794
C	-2.3770162	1.6798350	-2.0278127
C	-3.6800794	1.2059248	-1.7909156
C	-1.9562793	1.9394231	-3.3380431
C	-4.5546145	1.0146681	-2.8576547
H	-4.0062971	0.9912748	-0.7744996
C	-2.8363742	1.7353300	-4.4038274
H	-0.9413402	2.2742751	-3.5516291
C	-4.1324347	1.2766146	-4.1657440
H	-5.5643346	0.6479860	-2.6672117
H	-2.4709269	1.9101040	-5.4147344
H	-4.8144456	1.1109442	-5.0011483
C	2.5369055	1.0489494	1.8389181
C	3.7718840	0.3842625	1.9187707
C	1.8863571	1.4505089	3.0155530
C	4.3750908	0.1871322	3.1598337
H	4.2576008	0.0212270	1.0145804

C	2.4873807	1.2340807	4.2559343
H	0.8948144	1.9025763	2.9970438
C	3.7388557	0.6201256	4.3267753
H	5.3357058	-0.3268476	3.2142011
H	1.9424325	1.5190161	5.1540239
H	4.2036291	0.4468144	5.2981443
C	3.0057588	1.3405364	-1.0315599
C	2.7267819	0.8551230	-2.3140778
C	4.2373005	1.9692626	-0.7710421
C	3.6823650	0.9648147	-3.3264280
H	1.7574168	0.4128213	-2.5454109
C	5.1912865	2.0648198	-1.7810980
H	4.4508377	2.3703520	0.2201783
C	4.9154656	1.5567873	-3.0560272
H	3.4302582	0.6176990	-4.3279318
H	6.1498496	2.5432835	-1.5743343
H	5.6617114	1.6403655	-3.8474932
C	-1.5921869	-1.4494950	-1.1229719
C	-1.9263240	-1.3779732	0.0689986
C	-1.4399618	-1.7324458	-2.5495244
H	-1.1423770	-0.8210949	-3.0898248
H	-0.6129923	-2.4476089	-2.6690716
C	-2.5708330	-1.5140989	1.3794875
H	-1.9694256	-1.0308961	2.1620011
H	-2.5782556	-2.5897744	1.6172290
C	-2.7311851	-2.3187181	-3.1415112
H	-2.5833764	-2.5099201	-4.2124161
H	-2.9894348	-3.2632648	-2.6462246
H	-3.5630745	-1.6124853	-3.0282522
C	-3.9942804	-0.9378376	1.4066008
H	-4.6212623	-1.3879796	0.6252875
H	-4.4499775	-1.1363722	2.3854922
H	-3.9765806	0.1501034	1.2634585
C	1.2049155	-1.8254950	0.0307526
C	1.1695245	-2.3901340	1.2068430
C	1.9087804	-2.3499098	-1.1988885
H	1.5740714	-3.3807376	-1.3917443
H	1.6059839	-1.7641083	-2.0766603
C	0.6509829	-2.2359665	2.5692910
H	-0.1679110	-2.9549147	2.7315670
H	0.1972969	-1.2344306	2.6104651
C	3.4380672	-2.3095474	-1.0742144
H	3.9049615	-2.6790513	-1.9982454
H	3.7895351	-1.2820668	-0.9145754
H	3.7781148	-2.9276264	-0.2341268
C	1.6860221	-2.3457968	3.6943083
H	2.5529466	-1.7082051	3.4871705
H	1.2294973	-2.0065688	4.6326737
H	2.0366581	-3.3789749	3.8128105
F	-0.9367119	-0.0203676	3.9761181
F	0.4868822	0.1333949	-4.4074842
B	0.7981901	1.2478094	-5.2515836
B	-0.8767448	0.6084855	5.2723240
F	1.9900505	0.9873695	-5.9549170
F	0.9733451	2.3963823	-4.4105216
F	-0.2650402	1.4792575	-6.1450225
F	-2.1730461	0.6431048	5.8173479
F	0.0122539	-0.1104687	6.0891166
F	-0.4017962	1.9407827	5.0896206
F	-0.7989073	-4.6318265	-1.2716426
B	-0.8123856	-5.3318370	-0.0887041
F	-1.2898074	-6.6080933	-0.1800966
F	-1.2755983	-4.6118777	0.9916636
F	0.7680246	-5.5554724	0.2481729
F	1.9661344	-3.8545730	1.2350465
H	1.3042577	-4.7045201	0.7274622

TS-1’

Thermal correction = 1657.78 kJ/mol

Solvation energy = -258.67 kJ/mol

C	0.6935941	-0.0485765	-3.0000967
C	2.0893758	0.2314659	-2.4262842
H	0.5803027	-1.1187551	-3.2220626
H	0.5068848	0.5198448	-3.9202218
H	2.8800786	-0.1344943	-3.0954750
H	2.2523095	1.3051892	-2.2448423
Pt	0.2805368	0.0625996	0.3117155
P	-0.5837036	0.4433749	-1.7433502
P	2.1505310	-0.6659738	-0.8169677

C	-0.9155382	2.1635653	-2.1910412
C	-1.9572388	2.5073455	-3.0665389
C	-0.0357591	3.1455324	-1.7180387
C	-2.1235564	3.8397361	-3.4427472
H	-2.6420871	1.7421905	-3.4320525
C	-0.1957496	4.4732371	-2.1146706
H	0.7670928	2.8900166	-1.0265377
C	-1.2446985	4.8197935	-2.9685791
H	-2.9448431	4.1132701	-4.1067504
H	0.5084870	5.2165679	-1.7405163
H	-1.3824440	5.8605984	-3.2657150
C	-2.0886815	-0.5188573	-1.9939774
C	-3.2915579	-0.0195741	-1.4630109
C	-2.0467089	-1.8140536	-2.5286772
C	-4.4422190	-0.8024782	-1.5054066
H	-3.3245988	0.9590050	-0.9852648
C	-3.1972607	-2.6007604	-2.5435860
H	-1.1108882	-2.2386712	-2.8894831
C	-4.3951022	-2.0935123	-2.0399492
H	-5.3651353	-0.4106513	-1.0782233
H	-3.1389438	-3.6282942	-2.8995319
H	-5.2875631	-2.7197190	-2.0299980
C	3.7185786	-0.3387344	0.0210722
C	4.2584726	-1.3170982	0.8738171
C	4.3149934	0.9265004	-0.0572207
C	5.3950071	-1.0305519	1.6273002
H	3.7938427	-2.3011350	0.9396615
C	5.4478137	1.2098920	0.7080333
H	3.9001709	1.7030461	-0.6981528
C	5.9890301	0.2341614	1.5474988
H	5.8114087	-1.7933453	2.2867284
H	5.8760271	2.2099270	0.6476960
H	6.8708711	0.4593521	2.1493200
C	2.2318709	-2.4039671	-1.3401152
C	1.0964489	-3.2166783	-1.2426781
C	3.4069201	-2.9070395	-1.9266224
C	1.1273520	-4.5283482	-1.7265730
H	0.1830153	-2.8507772	-0.7716617
C	3.4350742	-4.2147652	-2.4062402
H	4.2968462	-2.2787600	-1.9934326

C	2.2954300	-5.0232701	-2.3075572
H	0.2332213	-5.1448181	-1.6232754
H	4.3492360	-4.6064900	-2.8553747
H	2.3246290	-6.0492182	-2.6780974
C	-2.1448890	0.2367491	1.8840915
C	-1.3036307	1.0559552	1.3223550
C	-2.4318668	-1.1805939	2.1208464
H	-1.8169125	-1.7171357	1.3813342
H	-2.0455271	-1.4549787	3.1178329
C	-1.2120035	2.5574937	1.4598768
H	-0.2286571	2.8922753	1.1023259
H	-1.2610421	2.8060833	2.5321336
C	-3.8727454	-1.6747774	1.9667182
H	-3.8645804	-2.7717127	1.9804782
H	-4.5154904	-1.2982592	2.7696746
H	-4.2906957	-1.3509034	1.0086204
C	-2.3119663	3.3372665	0.7260382
H	-3.2900735	3.1767422	1.1868223
H	-2.0826038	4.4115556	0.7610522
H	-2.3730225	3.0492609	-0.3291807
C	1.0232166	-1.1155319	2.1442987
C	1.2987999	0.0757635	2.3562791
C	0.8678789	-2.5716657	2.1711851
H	-0.0376538	-2.8777822	1.6296428
H	1.7016044	-3.0286434	1.6134035
C	1.7961849	1.3538284	2.8679580
H	0.9611123	1.8940363	3.3411780
H	2.1298649	1.9828968	2.0277748
C	0.8216350	-3.1170503	3.6054267
H	0.6817355	-4.2044855	3.5662962
H	1.7471022	-2.8887379	4.1519535
H	-0.0215031	-2.6808386	4.1569428
C	2.9493096	1.1439033	3.8616992
H	3.7875835	0.6331485	3.3715752
H	3.3035125	2.1179687	4.2242548
H	2.6290393	0.5423516	4.7235284
F	2.2931102	3.4157404	0.4227397
F	-1.6499091	-3.3716191	0.1246243
B	-1.8180437	-4.7918004	0.3289536
B	3.1294135	4.1343073	-0.4995590

F	-0.7036676	-5.2555166	1.0704426
F	-1.8449527	-5.4172511	-0.9451042
F	-3.0131352	-5.0196152	1.0208197
F	2.5901657	5.4095665	-0.7266249
F	4.4339587	4.2124317	0.0177178
F	3.1459859	3.3895552	-1.7269950
F	-3.2016531	1.0697847	2.9603901
B	-4.7169482	1.5285038	2.7002702
F	-5.4621615	0.7465721	3.5512206
F	-4.9476805	1.2916189	1.3588049
F	-4.7063393	2.8632017	3.0402658

Int-1

Thermal correction = 1645.77 kJ/mol

Solvation energy = -274.58 kJ/mol

C	-0.3167182	0.0311599	-2.9732592
C	1.1691661	-0.3362719	-2.8711708
H	-0.9413564	-0.8725552	-3.0256286
H	-0.5296368	0.6515409	-3.8543818
H	1.5011278	-0.9350198	-3.7298122
H	1.7962881	0.5652791	-2.8076571
Pt	0.2291197	-0.0384238	0.2872624
P	-0.7752282	0.9812119	-1.4453877
P	1.3540932	-1.3062266	-1.3042451
C	-0.1658871	2.6378879	-1.8565655
C	-0.8728554	3.4502416	-2.7606396
C	1.0740519	3.0592558	-1.3594571
C	-0.3507928	4.6878730	-3.1305918
H	-1.8322407	3.1195654	-3.1603575
C	1.5977166	4.2972878	-1.7396223
H	1.6408216	2.4347695	-0.6679947
C	0.8805602	5.1116116	-2.6169087
H	-0.9063341	5.3229304	-3.8226533
H	2.5654863	4.6074176	-1.3442085
H	1.2853028	6.0823401	-2.9075891
C	-2.5814944	1.0389406	-1.3641659
C	-3.2542933	2.2132317	-0.9916959
C	-3.3055577	-0.1451510	-1.5686488
C	-4.6444002	2.2097518	-0.8841992
H	-2.6929189	3.1211744	-0.7780218
C	-4.6948778	-0.1458650	-1.4437953
H	-2.8121648	-1.0917505	-1.7878194
C	-5.3652127	1.0346777	-1.1177599
H	-5.1625597	3.1247063	-0.5936718
H	-5.2283167	-1.0875278	-1.5650586
H	-6.4509343	1.0312958	-1.0117068
C	3.1101783	-1.4783107	-0.8988728
C	3.5871181	-2.6712137	-0.3294844
C	3.9468101	-0.3539976	-0.9572167
C	4.8970232	-2.7420054	0.1434826
H	2.9339668	-3.5412024	-0.2591166
C	5.2552329	-0.4277967	-0.4764976
H	3.5983154	0.6027681	-1.3468193
C	5.7316636	-1.6222837	0.0692040
H	5.2615832	-3.6719524	0.5829820
H	5.8734297	0.4697772	-0.5043632
H	6.7514193	-1.6757699	0.4535929
C	0.7974835	-2.9590619	-1.8214723
C	-0.4716750	-3.4129546	-1.4436405
C	1.6012500	-3.7511185	-2.6619765
C	-0.9344338	-4.6576011	-1.8837226
H	-1.1141832	-2.8115077	-0.7995159
C	1.1388825	-4.9908091	-3.0974811
H	2.5910351	-3.4008132	-2.9593594
C	-0.1270471	-5.4445249	-2.7051073
H	-1.9256456	-4.9901030	-1.5749843
H	1.7670534	-5.6058301	-3.7442981
H	-0.4848378	-6.4176088	-3.0456613
C	-1.6850447	0.9090666	2.3379439
C	-0.6116353	1.3061288	1.6485467
C	-2.5002435	-0.3319310	2.2175863
H	-2.1735369	-0.8653754	1.3140853
H	-2.2804090	-0.9998740	3.0705014
C	0.0666251	2.6270503	1.9442500
H	1.0609323	2.6651883	1.4787997
H	0.2352987	2.7054826	3.0326514
C	-4.0114410	-0.0919145	2.1389381
H	-4.5258633	-1.0336060	1.9088067
H	-4.4004160	0.3125502	3.0834685

H	-4.2456252	0.6244698	1.3416613
C	-0.7465395	3.8489465	1.4910750
H	-1.7330743	3.8566594	1.9723880
H	-0.2215718	4.7819940	1.7446595
H	-0.8920192	3.8404279	0.4031304
C	0.8218688	-1.5984162	1.8557394
C	1.5703567	-0.6298207	2.0542623
C	0.1338604	-2.8921991	1.8999375
H	-0.9205294	-2.7766308	1.6101006
H	0.5778309	-3.5562739	1.1406417
C	2.5925902	0.3157814	2.5013353
H	2.1294301	1.0311290	3.1989897
H	2.9548660	0.9147205	1.6519898
C	0.2332358	-3.5289858	3.2943203
H	-0.2814624	-4.4990422	3.2950550
H	1.2809805	-3.6843697	3.5859436
H	-0.2408255	-2.8846387	4.0463568
C	3.7734739	-0.4124947	3.1622949
H	4.2370803	-1.1093989	2.4528490
H	4.5307324	0.3221191	3.4660886
H	3.4505931	-0.9771802	4.0481908
F	3.5779675	2.6008385	0.5073352
F	-2.9223963	-2.8782618	0.3961961
B	-3.9498682	-3.5383104	-0.3610248
B	4.6267521	3.1261439	-0.3221623
F	-3.8049992	-4.9336872	-0.2096171
F	-3.7771164	-3.1923771	-1.7350098
F	-5.2096342	-3.1038021	0.0855605
F	4.5542703	4.5339022	-0.3203800
F	5.8724045	2.6844638	0.1672706
F	4.4263979	2.6377370	-1.6461394
F	-2.1520629	1.7290041	3.3734896

TS-2

Thermal correction = 1696.42 kJ/mol

Solvation energy = -255.67 kJ/mol

C	0.4764468	0.2633254	-2.9351611
C	1.8603297	0.8219877	-2.5978452
H	0.5327930	-0.8028193	-3.1974017
H	0.0092865	0.7993556	-3.7718261
H	2.5777345	0.6589272	-3.4134952
H	1.8157708	1.8936488	-2.3563332
Pt	0.6368472	0.1568291	0.4147005
P	-0.6464974	0.4207186	-1.4614705
P	2.3864378	-0.0689302	-1.0770922
C	-1.3618338	2.0682680	-1.6641098
C	-2.1580898	2.3591829	-2.7847997
C	-1.0495475	3.0754857	-0.7464219
C	-2.6523792	3.6493317	-2.9632355
H	-2.4041178	1.5785277	-3.5062039
C	-1.5271249	4.3706081	-0.9426783
H	-0.4015638	2.8660042	0.1011733
C	-2.3348021	4.6550741	-2.0437506
H	-3.2862042	3.8705104	-3.8228305
H	-1.2374303	5.1510470	-0.2405223
H	-2.7140983	5.6667007	-2.1945086
C	-1.9185234	-0.8458891	-1.7180703
C	-3.2875434	-0.5484473	-1.7740528
C	-1.4929206	-2.1854954	-1.7714056
C	-4.2149521	-1.5859901	-1.8858869
H	-3.6399355	0.4768692	-1.6935613
C	-2.4224691	-3.2180146	-1.8670994
H	-0.4351020	-2.4355698	-1.6946018
C	-3.7858747	-2.9141844	-1.9255319
H	-5.2772105	-1.3444547	-1.8923059
H	-2.0728436	-4.2501236	-1.8439277
H	-4.5175366	-3.7215370	-1.9713286
C	4.0565750	0.4714977	-0.6133658
C	4.9289371	-0.4635254	-0.0301802
C	4.4638186	1.8048847	-0.7635320
C	6.2037650	-0.0708004	0.3744247
H	4.6115191	-1.4991008	0.0935920
C	5.7395712	2.1909434	-0.3488715
H	3.7729958	2.5523382	-1.1506542
C	6.6121765	1.2568324	0.2125777
H	6.8764705	-0.8024301	0.8239954
H	6.0322651	3.2359998	-0.4473409
H	7.6066974	1.5646886	0.5387437
C	2.6602311	-1.7678933	-1.6711465

C	1.9402581	-2.8391726	-1.1330871
C	3.5786732	-1.9991870	-2.7106870
C	2.1106667	-4.1324930	-1.6371526
H	1.2295081	-2.6812738	-0.3230745
C	3.7540806	-3.2880963	-3.2083170
H	4.1632017	-1.1728603	-3.1192453
C	3.0152480	-4.3526661	-2.6759071
H	1.5055232	-4.9413482	-1.2265781
H	4.4661903	-3.4637711	-4.0163114
H	3.1461043	-5.3591235	-3.0761836
C	-1.5373580	-0.6117341	2.4062505
C	-0.8812062	0.4708209	1.8916702
C	-1.8568660	-1.9179952	1.7832224
H	-1.4011300	-1.9511471	0.7878708
H	-1.3536840	-2.7059741	2.3662823
C	-0.6584028	1.7092256	2.7630026
H	0.0382985	2.3823076	2.2470947
H	-0.1596870	1.3917346	3.6935686
C	-3.3564049	-2.2383951	1.7051613
H	-3.4662430	-3.2509671	1.3009992
H	-3.8270699	-2.2000410	2.6956064
H	-3.8813901	-1.5388313	1.0441937
C	-1.9149419	2.5233753	3.1072201
H	-2.6716172	1.9173012	3.6215874
H	-1.6410923	3.3613263	3.7631494
H	-2.3692006	2.9444088	2.2015453
C	1.8582167	-0.7417799	2.1401052
C	2.1849166	0.4553486	2.1001283
C	1.6871750	-2.1481332	2.5193432
H	0.9281786	-2.6383588	1.8951297
H	2.6283086	-2.6775473	2.2956266
C	2.8069884	1.7653260	2.2836224
H	2.0943436	2.4460124	2.7689355
H	3.0062706	2.2254963	1.3073269
C	1.3201218	-2.3060950	4.0028803
H	1.1917253	-3.3709687	4.2369877
H	2.1016086	-1.8898742	4.6530442
H	0.3792251	-1.7879218	4.2281208
C	4.1093229	1.6659419	3.0901463
H	4.8307582	1.0122407	2.5846000
H	4.5501340	2.6671434	3.1778671
H	3.9248570	1.2704467	4.0986331
F	1.1282506	4.2724328	1.2270640
F	0.0044017	-4.1765267	0.8002156
B	-0.7054806	-5.4100364	0.5511437
B	2.0194539	4.5055366	0.1427154
F	-0.1635079	-6.4169029	1.3597901
F	-0.5345053	-5.7329837	-0.8271967
F	-2.0717036	-5.2073641	0.8275926
F	1.7022073	5.7017939	-0.5042703
F	3.3539094	4.5135552	0.6151719
F	1.8847013	3.4057647	-0.7863078
F	-2.0434642	-0.5187843	3.6773423
B	-6.3836827	0.7814050	0.4162977
F	-6.5846361	-0.5733555	0.3372622
F	-5.8557860	1.3411765	-0.7275316
F	-7.4005024	1.4884693	0.9926668
H	-2.1188201	0.7076867	1.1630431
F	-3.0596840	1.1431220	0.5973589
H	-4.1555381	1.0225893	1.0724457
F	-5.1659773	0.9327904	1.5086658

TS-2’

Thermal correction = 1685.35 kJ/mol

Solvation energy = -258.11 kJ/mol

C	0.1610077	0.4538587	-2.9923831
C	0.5452752	1.8787609	-2.5916045
H	1.0530645	-0.1481288	-3.2178929
H	-0.4968523	0.4364623	-3.8714572
H	1.1581683	2.3686600	-3.3604016
H	-0.3418183	2.4945855	-2.3846546
Pt	0.1331620	0.4184601	0.3623880
P	-0.7347901	-0.3820640	-1.5927050
P	1.4819436	1.7078052	-1.0170445
C	-2.4635590	0.0526073	-1.8933282
C	-3.1003961	-0.3427970	-3.0822256
C	-3.1375423	0.8643115	-0.9765131
C	-4.4136163	0.0530326	-3.3261421
H	-2.5770272	-0.9652011	-3.8096550

C	-4.4441650	1.2752654	-1.2355876
H	-2.6344265	1.2143787	-0.0784498
C	-5.0852829	0.8615633	-2.4024994
H	-4.9129548	-0.2666962	-4.2418173
H	-4.9381860	1.9388870	-0.5276415
H	-6.1098517	1.1778537	-2.6026857
C	-0.4695268	-2.1512521	-1.9028244
C	-1.5145335	-3.0505484	-2.1505291
C	0.8528732	-2.6239683	-1.8308452
C	-1.2304174	-4.4044866	-2.3366119
H	-2.5468660	-2.7110516	-2.1575266
C	1.1306260	-3.9779001	-2.0008025
H	1.6730633	-1.9445849	-1.6013185
C	0.0841334	-4.8680108	-2.2595413
H	-2.0536662	-5.0995412	-2.5020689
H	2.1569426	-4.3264977	-1.8832473
H	0.2953918	-5.9322887	-2.3702984
C	2.0333858	3.3565503	-0.4913404
C	3.2989446	3.4997320	0.1024654
C	1.1910700	4.4711636	-0.6145765
C	3.7260131	4.7526792	0.5407853
H	3.9512223	2.6327200	0.2078583
C	1.6234578	5.7198650	-0.1650350
H	0.1834785	4.3638468	-1.0158994
C	2.8904108	5.8655598	0.4036967
H	4.7113120	4.8574881	0.9971737
H	0.9464420	6.5701204	-0.2441653
H	3.2229455	6.8433142	0.7555869
C	3.0291480	0.9059601	-1.5456709
C	3.4262469	-0.3130709	-0.9872698
C	3.8134895	1.4974403	-2.5518001
C	4.5821352	-0.9575342	-1.4389886
H	2.8347218	-0.7850199	-0.2042275
C	4.9692917	0.8602617	-2.9967834
H	3.5244939	2.4605605	-2.9763854
C	5.3491371	-0.3701568	-2.4451055
H	4.8431385	-1.9286012	-1.0179574
H	5.5740527	1.3211044	-3.7795936
H	6.2473565	-0.8737753	-2.8055423
C	-0.7743083	-1.8334314	2.2027788
C	-1.1309509	-0.6127802	1.7164695
C	0.1575728	-2.8499301	1.6472866
H	0.6120473	-2.4452380	0.7361737
H	0.9854867	-2.9718742	2.3639642
C	-2.0493896	0.3018310	2.5260500
H	-2.0676779	1.2918874	2.0529703
H	-1.5950444	0.4372365	3.5223649
C	-0.4683222	-4.2227581	1.3723732
H	0.3273445	-4.9019416	1.0447061
H	-0.9414807	-4.6323569	2.2735748
H	-1.2291968	-4.1598053	0.5855980
C	-3.5048007	-0.1582466	2.6872238
H	-3.5768599	-1.1463990	3.1560353
H	-4.0475053	0.5658405	3.3109199
H	-4.0156962	-0.2064532	1.7177475
C	1.4651392	0.7747923	2.1971687
C	0.7341176	1.7759762	2.1297118
C	2.4236905	-0.2476517	2.6304365
H	2.3998052	-1.1216252	1.9657615
H	3.4384369	0.1699936	2.5218832
C	0.0733650	3.0681566	2.3115876
H	-0.8947178	2.9119060	2.8078024
H	-0.1831223	3.5047033	1.3376532
C	2.1750753	-0.6909934	4.0803953
H	2.9031856	-1.4646685	4.3573709
H	2.2705172	0.1534194	4.7765936
H	1.1668059	-1.1101096	4.1901794
C	0.9487114	4.0444579	3.1092078
H	1.9027005	4.2185788	2.5968753
H	0.4233085	5.0038664	3.1990412
H	1.1582229	3.6594860	4.1169446
F	-2.8310544	3.2846381	0.9976920
F	3.1503449	-2.7426170	0.8174063
B	3.7552735	-4.0329122	0.5875014
B	-2.4894634	4.1931565	-0.0421226
F	4.8607146	-4.1747043	1.4369195
F	4.1727993	-4.0751146	-0.7765752
F	2.7930056	-5.0333355	0.8229826
F	-3.6411293	4.7062008	-0.6427840
F	-1.6786729	5.2298654	0.4849380

F	-1.7098490	3.4767817	-1.0243567
F	-1.3200192	-2.2433253	3.3951130
H	-2.0794023	-1.5875455	0.9372587
F	-2.8446122	-2.0231644	0.2461173
B	-4.1031701	-3.0745877	0.6611536
F	-3.9814242	-4.0335738	-0.3079339
F	-3.7825078	-3.4730845	1.9268268
F	-5.2016369	-2.2743652	0.5481104

TS-3

Thermal correction = 1638.79 kJ/mol

Solvation energy = -152.62 kJ/mol

P	-1.7686182	1.1310713	1.0724756
C	1.9170158	0.7004363	-3.7377148
H	2.5240093	1.5289900	-3.3353221
C	0.1590410	1.8104013	-1.6218578
Pt	-0.2533878	0.0655534	-0.3203792
C	1.0717569	2.8928382	-1.2519699
C	-1.7944439	0.3177436	2.7413953
H	-1.2103361	0.9387315	3.4328089
H	-2.8340700	0.2981641	3.0922675
C	-1.2143645	-1.0967944	2.6373305
H	-1.9149037	-1.7700843	2.1173558
H	-0.9966400	-1.5243892	3.6255603
C	1.1813063	0.0838931	-2.6495596
P	0.3074657	-1.0418548	1.6129527
C	1.6042405	-2.1854890	-1.6394305
H	2.3424697	-2.1646896	-0.8241242
C	1.0301411	-0.8005469	-1.7064103
C	-0.7135339	1.1454161	-2.2611027
C	-1.8431653	0.8087434	-3.1398329
H	-2.7552573	1.0905157	-2.5867703
H	0.8307481	3.2108012	-0.2274665
H	0.7859875	-2.8470080	-1.3167309
H	2.6851455	-0.0692239	-3.9529605
H	-1.7889649	1.4638470	-4.0248762
H	2.0959287	2.4839826	-1.2097492
C	1.1391637	1.1002824	-4.9897814
H	1.8314119	1.4702646	-5.7565800
H	0.5847017	0.2505303	-5.4077608
H	0.4240063	1.9048439	-4.7704684
C	2.2636392	-2.6929650	-2.9189349
H	3.1671707	-2.1138297	-3.1486246
H	2.5630816	-3.7411413	-2.7855987
H	1.5712647	-2.6497029	-3.7727920
C	-1.9338831	-0.6649944	-3.5525921
H	-1.9786292	-1.3243359	-2.6774694
H	-2.8400737	-0.8190740	-4.1509525
H	-1.0633603	-0.9570689	-4.1516775
C	0.9789352	4.0743143	-2.2276436
H	1.2623512	3.7731949	-3.2444163
H	-0.0374125	4.4880455	-2.2565928
H	1.6669319	4.8655318	-1.9039608
C	-1.3139987	2.8701884	1.3047554
C	-1.8084807	3.8411812	0.4170030
C	-0.2937229	3.2162465	2.2071101
C	-1.2980709	5.1394692	0.4429644
C	0.2142314	4.5142183	2.2243232
C	-0.2838364	5.4777683	1.3419925
H	-2.5900903	3.5770756	-0.2953060
H	0.1363789	2.4689955	2.8738687
H	-1.6900123	5.8876415	-0.2466443
H	1.0128844	4.7667745	2.9217726
H	0.1202680	6.4902494	1.3541634
C	-3.4803966	1.0996821	0.4843895
C	-4.4450263	1.9998416	0.9692551
C	-3.8552853	0.0770829	-0.3963213
C	-5.7691700	1.8819695	0.5504403
C	-5.1859166	-0.0466715	-0.7977196
C	-6.1394125	0.8601332	-0.3324060
H	-4.1609605	2.7892454	1.6665673
H	-3.1179730	-0.6461650	-0.7409140
H	-6.5175312	2.5835391	0.9207935
H	-5.4604565	-0.8720897	-1.4533636
H	-7.1797681	0.7665624	-0.6461911
C	1.4451031	0.0791654	2.4840812
C	1.4288969	0.1784887	3.8844802
C	2.3188115	0.8834935	1.7392805
C	2.2594438	1.0979698	4.5259841

C	3.1657165	1.7829066	2.3866164
C	3.1263166	1.9022230	3.7771281
H	0.7700863	-0.4567773	4.4778268
H	2.3666797	0.7931431	0.6560740
H	2.2372050	1.1794622	5.6132385
H	3.8577264	2.3759562	1.7892262
H	3.7809326	2.6120933	4.2843379
C	1.0530041	-2.6793338	1.6153061
C	0.2473699	-3.8173753	1.4508972
C	2.4512489	-2.7969645	1.6822609
C	0.8474336	-5.0724490	1.3629905
C	3.0372440	-4.0584284	1.5918066
C	2.2380977	-5.1941129	1.4322058
H	-0.8340520	-3.7360922	1.3600284
H	3.0790872	-1.9102661	1.7645257
H	0.2187373	-5.9512024	1.2210096
H	4.1229027	-4.1477917	1.6303104
H	2.7022425	-6.1779536	1.3523464
F	-3.7673767	-2.9286859	-1.3331306
F	5.8666737	0.5548803	-0.7474690
B	-2.7809769	-3.2031541	-0.3726410
F	-3.2109333	-2.7612225	0.9148156
F	-1.5981232	-2.4279863	-0.7087878
F	-2.4385868	-4.5529967	-0.3321497
B	4.5610144	0.2645815	-1.1220109
F	3.9784393	-0.6851436	-0.2326966
F	4.4946257	-0.2333070	-2.4463202
F	3.7627584	1.4719103	-1.0564011

Int-2

Thermal correction = 1654.96 kJ/mol

Solvation energy = -146.19 kJ/mol

C	0.5997235	-3.4159697	-0.3108404
C	1.3595930	-3.1652191	0.9928453
H	-0.4753067	-3.5663082	-0.1264298
H	0.9858704	-4.2962542	-0.8437505
H	1.0438016	-3.8633779	1.7789556
H	2.4422734	-3.2780207	0.8453638
Pt	0.0332426	-0.1667579	-0.0379867
P	0.7412230	-1.9229739	-1.3777452
P	1.1157009	-1.4192584	1.6008048
C	-0.5981182	1.4138548	0.9982628
C	-2.0019496	1.5721615	0.7163887
C	0.1415986	2.3178476	1.8904036
H	1.2059425	2.0659031	1.9236179
C	-2.9884463	1.8893243	1.7887016
H	-2.6720422	2.8017948	2.3184062
C	-1.1010026	0.9009616	-1.3475010
C	-2.2549112	1.4026131	-0.6535686
C	-0.9563278	1.0259562	-2.8072652
H	-1.6415908	1.7899379	-3.2083064
C	-3.5498647	1.7206885	-1.3350565
H	-4.2205120	2.2387858	-0.6366520
H	-0.2845525	2.0959085	2.8956964
H	-3.9732491	2.0840592	1.3454451
H	-3.3546245	2.4238443	-2.1614053
H	-1.3230604	0.0500967	-3.1989375
C	0.1024863	-1.5119790	3.1012567
C	0.3920123	-0.6878928	4.2010960
C	-1.0448501	-2.3188304	3.1079456
C	-0.4574693	-0.6904268	5.3084898
C	-1.8892998	-2.3116897	4.2175899
C	-1.5962003	-1.5020085	5.3180897
H	1.2834778	-0.0599451	4.1931482
H	-1.3109515	-2.9283199	2.2434398
H	-0.2272943	-0.0569930	6.1659945
H	-2.7911267	-2.9224173	4.1970234
H	-2.2611057	-1.4957467	6.1824869
C	2.7499181	-0.8467092	2.1149802
C	3.2835755	0.3133904	1.5437519
C	3.4925915	-1.5754055	3.0614629
C	4.5583637	0.7506351	1.9096099
C	4.7654595	-1.1377481	3.4193711
C	5.2974497	0.0233190	2.8426544
H	2.7106093	0.8771701	0.8087741
H	3.0734568	-2.4721645	3.5211553
H	4.9465324	1.6593332	1.4519125
H	5.3448432	-1.7007375	4.1521109
H	6.2944200	0.3606316	3.1291792

C	-0.2332180	-2.2626278	-2.8623185
C	0.3200080	-2.1755858	-4.1480332
C	-1.6045096	-2.5292738	-2.6997477
C	-0.4955428	-2.3640162	-5.2654053
C	-2.4081636	-2.7244355	-3.8213828
C	-1.8561662	-2.6394139	-5.1039160
H	1.3803772	-1.9550675	-4.2719163
H	-2.0530950	-2.5667071	-1.7074017
H	-0.0652342	-2.2934612	-6.2648327
H	-3.4680085	-2.9363552	-3.6804501
H	-2.4888167	-2.7851645	-5.9802354
C	2.4871916	-1.7974614	-1.8359119
C	3.1495587	-0.5705518	-1.6829832
C	3.1931939	-2.9244472	-2.2917216
C	4.5100947	-0.4698289	-1.9773488
C	4.5512927	-2.8173517	-2.5857021
C	5.2092194	-1.5917157	-2.4251709
H	2.6121530	0.3117390	-1.3316340
H	2.6841526	-3.8813114	-2.4194820
H	4.9991355	0.4942259	-1.8406109
H	5.0992178	-3.6921067	-2.9377711
H	6.2737370	-1.5159938	-2.6506070
C	-0.0344614	3.8145188	1.5684046
H	-1.0875381	4.1248790	1.5858654
H	0.5250482	4.4082381	2.2997518
H	0.3886626	4.0171655	0.5786062
C	-3.0903391	0.7211952	2.7971294
H	-3.8235202	0.9726994	3.5738518
H	-3.3970815	-0.2028556	2.2948951
H	-2.1250084	0.5356661	3.2855517
C	-4.2448004	0.4579448	-1.8829783
H	-5.2026550	0.7330666	-2.3434988
H	-3.6291913	-0.0355605	-2.6460331
H	-4.4227456	-0.2750781	-1.0884357
C	0.4681269	1.2470589	-3.3249920
H	0.4510589	1.3364811	-4.4184887
H	0.9082627	2.1488434	-2.8850668
H	1.1197738	0.4069384	-3.0626626
F	-2.4351997	-3.6924314	0.5281434
B	-3.4101303	-2.6432405	0.5068070
F	-4.2593611	-2.8104510	-0.5976495
F	-2.6978188	-1.4014815	0.3505702
F	-4.1137476	-2.6162411	1.7165926
F	3.0391971	3.4510500	0.9452099
B	2.9767839	3.2122068	-0.4515487
F	4.1344762	2.5058818	-0.8611523
F	2.8144025	4.3982603	-1.1631917
F	1.8297776	2.3577622	-0.6956550

TS-4

Thermal correction = 1644.65 kJ/mol

Solvation energy = -135.38 kJ/mol

C	2.7105557	-1.1243959	-0.7711113
C	3.0685935	-0.3015472	0.4688781
H	2.3422910	-2.1220017	-0.4894949
H	3.5744288	-1.2501771	-1.4381099
H	3.6256553	-0.8981940	1.2026364
H	3.6748367	0.5739017	0.1983024
Pt	-0.2750534	0.0482805	-0.0790369
P	1.3361197	-0.2689427	-1.6531396
P	1.5262444	0.3680955	1.2673278
C	-1.8993380	0.1177113	1.2531189
C	-2.2292332	-1.2569263	1.2434003
C	-2.1543018	1.1386463	2.2828475
H	-1.8591662	2.1327959	1.9249488
C	-2.3411119	-2.1958080	2.3862055
H	-1.6460251	-3.0276876	2.1649794
C	-2.2673093	-0.2198206	-0.7345149
C	-2.5115649	-1.4889072	-0.1524449
C	-3.0186101	0.5096906	-1.7695187
H	-3.3878665	1.4211334	-1.2539825
C	-3.0684390	-2.7068938	-0.7963795
H	-2.7741716	-3.5781824	-0.1951513
H	-1.4462941	0.8775870	3.0997572
H	-1.9736373	-1.6974113	3.2939106
H	-4.1719550	-2.6394837	-0.7442210
H	-3.9018654	-0.0747529	-2.0756838
C	1.2729398	-0.5439508	2.8148485
C	0.9035151	0.1305128	3.9894735

C	1.3056845	-1.9469062	2.7886071
C	0.5682806	-0.6016453	5.1307454
C	0.9709206	-2.6687270	3.9331954
C	0.5985284	-1.9985786	5.1026975
H	0.8874491	1.2205032	4.0107227
H	1.5641183	-2.4895221	1.8787001
H	0.2845087	-0.0765312	6.0435166
H	0.9818666	-3.7574086	3.8905329
H	0.3309672	-2.5664326	5.9946093
C	1.8772290	2.0767928	1.7368056
C	0.9479288	3.0762773	1.4269851
C	3.0551537	2.3977522	2.4332790
C	1.1884167	4.3952843	1.8161453
C	3.2955934	3.7184099	2.8072579
C	2.3614808	4.7159316	2.5009183
H	0.0396072	2.8401947	0.8726543
H	3.7753593	1.6193118	2.6913137
H	0.4419579	5.1536209	1.5813357
H	4.2106080	3.9707202	3.3443193
H	2.5525063	5.7465821	2.8026063
C	0.9917736	-1.2690056	-3.1234277
C	0.6931148	-0.6387818	-4.3417547
C	0.9738804	-2.6700575	-3.0364578
C	0.3766417	-1.4059077	-5.4621018
C	0.6609737	-3.4296091	-4.1639634
C	0.3587664	-2.8013198	-5.3744076
H	0.7116652	0.4490454	-4.4110292
H	1.1736125	-3.1747211	-2.0934982
H	0.1449909	-0.9118707	-6.4062355
H	0.6426730	-4.5162826	-4.0816615
H	0.1097180	-3.3987684	-6.2521678
C	2.0628345	1.2835344	-2.2519011
C	1.4342963	2.4993060	-1.9533679
C	3.2663697	1.2751352	-2.9771177
C	2.0114029	3.7015633	-2.3677549
C	3.8391616	2.4782425	-3.3844804
C	3.2133607	3.6923634	-3.0765890
H	0.4926544	2.5106475	-1.4028606
H	3.7499786	0.3301887	-3.2306558
H	1.5119909	4.6409939	-2.1310840
H	4.7748868	2.4709752	-3.9445628
H	3.6660238	4.6317588	-3.3962556
C	-3.5935538	1.1760357	2.8144926
H	-3.9009239	0.2092866	3.2357794
H	-3.6755254	1.9388308	3.5988115
H	-4.2767967	1.4564256	2.0039586
C	-3.7558011	-2.7589271	2.6180840
H	-3.7392649	-3.4400743	3.4777895
H	-4.4782787	-1.9584953	2.8257327
H	-4.1124271	-3.3265363	1.7499538
C	-2.6149965	-2.8897651	-2.2498807
H	-3.0323341	-3.8192997	-2.6557991
H	-2.9386629	-2.0607196	-2.8913531
H	-1.5228832	-2.9601397	-2.2882135
C	-2.1892651	0.9511956	-2.9827722
H	-2.8235842	1.5221991	-3.6723913
H	-1.3686734	1.5997826	-2.6564392
H	-1.7770330	0.0853038	-3.5161236
F	1.7619856	-4.1342700	0.4121585
B	0.3540273	-4.2601693	0.2566046
F	0.0438549	-5.1531346	-0.7723315
F	-0.1449990	-2.9531009	-0.0952734
F	-0.2454314	-4.6414692	1.4765272
F	-2.0482037	4.2015266	1.2121894
B	-2.4962839	3.8336223	-0.0826860
F	-2.8011340	4.9457208	-0.8541406
F	-3.6245956	2.9676538	0.0435268
F	-1.4413099	3.0739809	-0.7129991

Int-3

Thermal correction = 1659.18 kJ/mol

Solvation energy = -135.16 kJ/mol

C	0.4263229	-3.3649973	-0.1622623
C	1.2396719	-2.9861959	1.0788267
H	-0.6147952	-3.6040959	0.0919194
H	0.8712158	-4.2293953	-0.6750694
H	1.1059612	-3.7209017	1.8838794
H	2.3116040	-2.9361188	0.8409473
Pt	-0.2109850	-0.1147571	-0.0020797

P	0.4276889	-1.9268022	-1.3062313
P	0.8179950	-1.2934710	1.7266559
C	-0.3796815	2.1077766	0.0792949
C	-1.4833811	1.5145157	0.7675537
C	0.6765691	3.0560214	0.4566478
H	1.6412498	2.7315063	0.0343407
C	-2.0356130	1.6493755	2.1426732
H	-1.1956694	1.6295976	2.8539861
C	-0.9011050	1.5737818	-1.2493005
C	-2.0466054	1.0602746	-0.5636889
C	-0.5879957	1.8330242	-2.6813911
H	-1.0187088	2.8208595	-2.9292907
C	-3.3475367	0.4798727	-0.9445316
H	-3.5243818	-0.3930229	-0.2966160
H	0.7736415	3.0835659	1.5510910
H	-2.4758252	2.6596954	2.2279937
H	-4.1150828	1.2200572	-0.6463032
H	-1.1416746	1.1015161	-3.2868020
C	-0.2516707	-1.5180375	3.1695558
C	-0.1984718	-0.5647235	4.2008530
C	-1.1723429	-2.5724811	3.2349743
C	-1.0616846	-0.6733189	5.2892318
C	-2.0360483	-2.6703519	4.3257418
C	-1.9833052	-1.7236018	5.3504210
H	0.5326559	0.2435415	4.1583470
H	-1.2519900	-3.2997847	2.4290544
H	-1.0131905	0.0628191	6.0923059
H	-2.7681273	-3.4760355	4.3507036
H	-2.6646370	-1.8008123	6.1982566
C	2.3697212	-0.6480913	2.3925576
C	2.9707336	0.5042686	1.8753543
C	2.9923775	-1.3612747	3.4341548
C	4.1924622	0.9477566	2.3881196
C	4.2117345	-0.9180328	3.9390933
C	4.8105664	0.2340904	3.4150542
H	2.5060332	1.0597647	1.0629501
H	2.5172996	-2.2490044	3.8545839
H	4.6448547	1.8423884	1.9630870
H	4.6954556	-1.4713375	4.7449196
H	5.7670506	0.5764595	3.8115877
C	-0.6001508	-2.3155055	-2.7358052
C	-0.3297543	-1.6699591	-3.9556083
C	-1.7162228	-3.1566525	-2.6151474
C	-1.1753547	-1.8670975	-5.0452362
C	-2.5552556	-3.3472591	-3.7140461
C	-2.2902378	-2.7039522	-4.9245225
H	0.5439337	-1.0241056	-4.0490911
H	-1.9547650	-3.6450908	-1.6719224
H	-0.9628684	-1.3680078	-5.9911563
H	-3.4285198	-3.9900736	-3.6075802
H	-2.9535893	-2.8516269	-5.7773354
C	2.1412387	-1.8122339	-1.8779457
C	2.9462613	-0.7446586	-1.4640695
C	2.6871942	-2.8383211	-2.6693676
C	4.2947226	-0.6935676	-1.8212202
C	4.0326808	-2.7846152	-3.0273948
C	4.8351632	-1.7181587	-2.5997806
H	2.5250747	0.0740689	-0.8838026
H	2.0609155	-3.6641608	-3.0115284
H	4.8941371	0.1575826	-1.4936766
H	4.4581908	-3.5758806	-3.6457345
H	5.8867924	-1.6839814	-2.8868720
C	0.3859207	4.4679131	-0.0974604
H	-0.5501245	4.8801632	0.3037542
H	1.2181434	5.1253903	0.1798153
H	0.3224376	4.4536001	-1.1933623
C	-3.0754034	0.5899923	2.5147194
H	-3.3490177	0.6861175	3.5721580
H	-3.9912160	0.7011842	1.9202348
H	-2.6965791	-0.4241543	2.3484882
C	-3.5044737	0.0898583	-2.4120969
H	-4.4682181	-0.4125012	-2.5547869
H	-3.4658777	0.9612063	-3.0793295
H	-2.7233232	-0.6185453	-2.7118522
C	0.8956960	1.8135628	-3.0433154
H	1.0251909	2.0513838	-4.1071148
H	1.4898640	2.5246135	-2.4590295
H	1.3294987	0.8225000	-2.8604807
F	-2.6025888	-4.2724910	0.4820460
B	-3.3153123	-3.0440027	0.5639101

F	-4.1541557	-2.8804087	-0.5465846
F	-2.3097302	-1.9860908	0.5218745
F	-4.0125840	-2.9485801	1.7662989
F	3.8842918	3.6006472	0.6726851
B	4.2964708	3.0377006	-0.5661287
F	5.4205695	2.2070679	-0.3714025
F	4.5351551	4.0272152	-1.5139550
F	3.2052052	2.1933594	-1.0275219

TS-5

Thermal correction = 1643.01 kJ/mol

Solvation energy = -120.88 kJ/mol

C	-1.1263271	-2.9714485	-0.3474624
C	-0.8031229	-3.0128835	1.1503855
H	-2.1281184	-2.5480833	-0.5045436
H	-1.1043606	-3.9781932	-0.7869079
H	-1.6725446	-3.3599338	1.7228871
H	0.0358334	-3.6936014	1.3504606
Pt	0.1175526	0.0489531	0.0140047
P	0.0746465	-1.8794015	-1.2147562
P	-0.2803681	-1.3421207	1.7918131
C	1.4738062	2.0497949	0.0653094
C	0.1306262	2.1127586	0.7105469
C	2.7162035	2.3778474	0.5436140
H	2.7702706	2.4875257	1.6337895
C	-0.2932626	2.6489053	2.0372644
H	0.2275552	2.0856287	2.8284298
C	0.7856039	1.7384395	-1.2436603
C	-0.4681943	2.1327664	-0.6268447
C	1.2339700	1.6381769	-2.6649412
H	1.5009774	2.6506057	-3.0104002
C	-1.8130540	2.5046365	-1.1158872
H	-2.5502074	2.0032218	-0.4710652
H	2.3306897	3.9065740	0.3382277
H	0.0633300	3.6917765	2.1207000
H	-1.9167910	3.5878937	-0.9247789
H	0.3722814	1.3162134	-3.2647079
C	-1.4738180	-0.8553907	3.0640776
C	-1.0307814	-0.1958783	4.2221443
C	-2.8472338	-1.0229127	2.8314880
C	-1.9605208	0.2933167	5.1393608
C	-3.7683823	-0.5235086	3.7514181
C	-3.3288968	0.1356371	4.9024151
H	0.0365057	-0.0726557	4.4082653
H	-3.2088281	-1.5107981	1.9265887
H	-1.6141137	0.8024748	6.0395590
H	-4.8324062	-0.6368167	3.5461979
H	-4.0536644	0.5300937	5.6154738
C	1.2601844	-1.6521260	2.7194081
C	2.3550976	-0.7933098	2.5616500
C	1.3594135	-2.7406549	3.5991775
C	3.5397766	-1.0241129	3.2626824
C	2.5462785	-2.9751063	4.2928430
C	3.6390204	-2.1188612	4.1236638
H	2.2730989	0.0530816	1.8776616
H	0.5052668	-3.4024991	3.7500459
H	4.3866599	-0.3494181	3.1320534
H	2.6182652	-3.8261881	4.9707320
H	4.5657969	-2.3049247	4.6674039
C	-0.4643040	-1.8012074	-2.9415164
C	0.4328226	-2.0010223	-4.0019833
C	-1.7969145	-1.4437883	-3.2087407
C	-0.0013210	-1.8476126	-5.3189119
C	-2.2228212	-1.3022164	-4.5291415
C	-1.3285109	-1.4993868	-5.5845533
H	1.4673873	-2.2775537	-3.7994956
H	-2.5069965	-1.2580319	-2.4022469
H	0.7000679	-2.0031628	-6.1394706
H	-3.2602654	-1.0285613	-4.7211614
H	-1.6649211	-1.3815259	-6.6152580
C	1.6303566	-2.8259742	-1.1785379
C	2.7545164	-2.2670884	-0.5568048
C	1.7010558	-4.1324055	-1.6886043
C	3.9325008	-3.0042983	-0.4327129
C	2.8814203	-4.8653116	-1.5713235
C	3.9963461	-4.3037366	-0.9393209
H	2.6869415	-1.2518139	-0.1638186
H	0.8341160	-4.5738524	-2.1828877
H	4.7976404	-2.5641557	0.0637207

H	2.9319699	-5.8790723	-1.9699303
H	4.9154176	-4.8828185	-0.8431751
C	4.0281259	2.2382454	-0.1883144
H	3.9622440	2.6146146	-1.2160721
H	4.8131254	2.8121749	0.3207603
H	4.3605814	1.1880940	-0.2295092
C	-1.8057825	2.5930878	2.2664629
H	-2.0464923	2.8567925	3.3035606
H	-2.3325332	3.2964265	1.6094881
H	-2.2024854	1.5893256	2.0705356
C	-2.0980860	2.1887140	-2.5818224
H	-3.1433567	2.4260117	-2.8142121
H	-1.4555824	2.7697976	-3.2560864
H	-1.9560118	1.1204185	-2.7850994
C	2.4010870	0.6820157	-2.9086382
H	2.6124433	0.6062918	-3.9832011
H	3.3179930	1.0123610	-2.4083496
H	2.1649489	-0.3233777	-2.5385462
F	-4.1613682	-2.1034768	0.0289058
B	-4.1997218	-0.7303574	-0.3642772
F	-4.5670615	-0.6263296	-1.7160080
F	-2.8485746	-0.2251894	-0.2239382
F	-5.0463354	-0.0033544	0.4723094
F	2.5459981	4.7192714	-2.1190659
B	1.6748944	5.4310054	-1.3452216
F	1.8489339	6.7649203	-1.2852976
F	0.3933881	4.9670581	-1.3330551
F	2.2188101	4.9627330	0.2761105

Int-4 (20-L2)

Thermal correction = 1606.70 kJ/mol

Solvation energy = -113.08 kJ/mol

C	-1.1527888	-2.8291833	-0.8106258
C	-0.9636417	-3.0603214	0.6909968
H	-2.1230082	-2.3491252	-1.0013964
H	-1.1221404	-3.7756616	-1.3678825
H	-1.8836147	-3.4512923	1.1437182
H	-0.1537275	-3.7792847	0.8778121
Pt	0.1578621	0.0669617	0.0181249
P	0.1550827	-1.6903413	-1.4303519
P	-0.4734576	-1.4867104	1.5622146
C	1.5987688	2.0158323	0.4864235
C	0.1827141	2.0249912	0.9927624
C	2.7842131	2.1772585	1.1050260
H	2.7476391	2.2811849	2.1944364
C	-0.3726149	2.3843154	2.3318421
H	0.0200922	1.6780367	3.0809941
C	1.0254548	1.8973422	-0.9101090
C	-0.2828095	2.2331502	-0.3764715
C	1.6353015	1.9699014	-2.2737413
H	2.0727868	2.9768457	-2.4019383
C	-1.5755486	2.6926205	-0.9492614
H	-2.3822227	2.1395420	-0.4475428
H	0.0266478	3.3750949	2.6183655
H	-1.6998993	3.7527000	-0.6607146
H	0.8349383	1.8832533	-3.0200402
C	-1.7978660	-1.1079570	2.7407296
C	-1.4879810	-0.6235946	4.0217206
C	-3.1354373	-1.1839888	2.3235905
C	-2.5130405	-0.2165456	4.8759057
C	-4.1523106	-0.7676751	3.1816933
C	-3.8442181	-0.2821676	4.4554095
H	-0.4499536	-0.5723313	4.3514079
H	-3.3931189	-1.5370588	1.3254508
H	-2.2694442	0.1569575	5.8714177
H	-5.1837217	-0.8068851	2.8326564
H	-4.6423998	0.0484039	5.1211735
C	0.9326448	-1.9632373	2.6257214
C	2.0823685	-1.1654328	2.6693546
C	0.8691979	-3.1257725	3.4094969
C	3.1605284	-1.5289315	3.4786348
C	1.9502981	-3.4917763	4.2104374
C	3.0988891	-2.6944430	4.2447732
H	2.1295988	-0.2602382	2.0597528
H	-0.0312415	-3.7420836	3.4015720
H	4.0510948	-0.8997237	3.5068562
H	1.8957383	-4.3992011	4.8128902
H	3.9429432	-2.9823355	4.8725977
C	-0.2437040	-1.4150195	-3.1784321

C	0.7117641	-1.5703483	-4.1943610
C	-1.5361672	-0.9657208	-3.4982692
C	0.3756460	-1.2817251	-5.5176163
C	-1.8658799	-0.6898075	-4.8248777
C	-0.9128626	-0.8432257	-5.8353053
H	1.7154811	-1.9208718	-3.9534883
H	-2.2878353	-0.8143724	-2.7232222
H	1.1227714	-1.4046747	-6.3027864
H	-2.8753434	-0.3491848	-5.0553945
H	-1.1734313	-0.6224469	-6.8711932
C	1.6659241	-2.7119615	-1.3900898
C	2.7515178	-2.2841468	-0.6147986
C	1.7321145	-3.9523019	-2.0450170
C	3.8858260	-3.0864001	-0.4844607
C	2.8687613	-4.7502452	-1.9197298
C	3.9449501	-4.3198399	-1.1359850
H	2.6866955	-1.3196130	-0.1087335
H	0.8951936	-4.2916782	-2.6576247
H	4.7203820	-2.7491085	0.1308764
H	2.9148712	-5.7127354	-2.4305282
H	4.8294077	-4.9498326	-1.0341402
C	4.1361513	2.2842665	0.4703480
H	4.0742244	2.4485904	-0.6112692
H	4.7061123	3.1209076	0.9028414
H	4.7411164	1.3760893	0.6380703
C	-1.9020240	2.4012041	2.3844103
H	-2.2515062	2.5310964	3.4162579
H	-2.3119460	3.2233009	1.7836064
H	-2.3239000	1.4642995	2.0006824
C	-1.7183020	2.5342013	-2.4617021
H	-2.7405260	2.7879697	-2.7683036
H	-1.0241072	3.1832826	-3.0131033
H	-1.5405645	1.4940507	-2.7604318
C	2.6992304	0.9075005	-2.5562259
H	3.1410085	1.0552470	-3.5509567
H	3.5087367	0.9227047	-1.8166833
H	2.2529492	-0.0928192	-2.5296228
F	-4.2119301	-1.9406527	-0.6917663
B	-4.2373931	-0.5285385	-0.9172364
F	-4.5042453	-0.2624667	-2.2710767
F	-2.9129643	-0.0384025	-0.6102484
F	-5.1632392	0.0814719	-0.0670436

6 References

[1] A. Tronnier, A. Poethig, E. Herdtweck, T. Strassner, "C^C* Cyclometalated Platinum(II) NHC Complexes with *β*-Ketoimine Ligands" *Organometallics* **2014**, *33*, 898-908.

[2] E. Costa, P. G. Pringle, M. Ravetz, R. J. Puddephatt, in *Inorganic Syntheses, Vol. 31*, **1996**, pp. 284-286.

[3] H. C. Clark, L. E. Manzer, "Reactions of (π-1,5-cyclooctadiene) organoplatinum(II) compounds and the synthesis of perfluoroalkylplatinum complexes" *J. Organomet. Chem.* **1973**, *59*, 411-428.

[4] O. Shyshov, R. C. Brachvogel, T. Bachmann, R. Srikantharajah, D. Segets, F. Hampel, R. Puchta, M. von Delius, "Adaptive Behavior of Dynamic Orthoester Cryptands" *Angew. Chem. Int. Ed. Engl.* **2017**, *56*, 776-781.

[5] A. Akhdar, J.-M. Andanson, S. Faure, M. Traïkia, A. Gautier, "Application of Quantitative ^{1}H and ^{19}F NMR to Organometallics" *J. Organomet. Chem.* **2021**, *950*.

[6] A. O. Mattes, D. Russell, E. Tishchenko, Y. Liu, R. H. Cichewicz, S. J. Robinson, "Application of ^{19}F quantitative NMR to pharmaceutical analysis" *Concepts Magn. Reson. A* **2016**, *45A,* e21422.

[7] H. A. Naqi, T. J. Woodman, S. M. Husbands, I. S. Blagbrough, "^{19}F and ^{1}H quantitative-NMR spectroscopic analysis of fluorinated third-generation synthetic cannabinoids" *Anal. Methods* **2019**, *11*, 3090-3100.

[8] G. M. Sheldrick, "SADABS Program for Area Detector Absorption Correction", Institute for Inorganic Chemistry, University of Göttingen, Göttingen, Germany, **1996**.

[9] G. M. Sheldrick, "SHELXT - integrated space-group and crystal-structure determination" *Acta Crystallogr. Sect. A* **2015**, *71*, 3-8.

[10] G. M. Sheldrick, "Crystal structure refinement with SHELXL" *Acta Crystallogr. C* **2015**, *71*, 3-8.

[11] J. Wassenaar, J. N. Reek, "INDOLPhos: novel hybrid phosphine-phosphoramidite ligands for asymmetric hydrogenation and hydroformylation" *Dalton Trans.* **2007**, 3750-3753.

[12] G. Anderson, H. Clark, J. Davies, "Reactivity of platinum diolefin complexes. 2. Reactions with bulky and chelating Group 5B ligands and studies relating to carbonyl insertion" *Inorg. Chem.* **1981**, *20*, 3607-3611.

[13] Y. Y. Kuo, R. C. Da Costa, H. A. Sparkes, M. F. Haddow, G. R. Owen, "Palladium and Platinum Complexes Containing Diphenyl-2-(3-methyl)indolylphosphine" *Eur. J. Inorg. Chem.* **2020**, *2020*, 4195-4202.

[14] H. C. Clark, P. N. Kapoor, I. J. McMahon, "Mixed ligand complexes of platinum(0) containing diphosphines" *J. Organomet. Chem.* **1984**, *265*, 107-115.

[15] W. Villarreal, L. Colina-Vegas, C. Rodrigues de Oliveira, J. C. Tenorio, J. Ellena, F. C. Gozzo, M. R. Cominetti, A. G. Ferreira, M. A. Ferreira, M. Navarro, A. A. Batista, "Chiral Platinum(II) Complexes Featuring Phosphine and Chloroquine Ligands as Cytotoxic and Monofunctional DNA-Binding Agents" *Inorg. Chem.* **2015**, *54*, 11709-11720.

[16] A. L. Bandini, G. Banditelli, M. A. Cinellu, G. Sanna, G. Minghetti, F. Demartin, M. Manassero, "1,1'-Bis (diphenylphosphino) ferrocene complexes of platinum (II) and platinum (I). Crystal and molecular structure of [(L-L)Pt(*μ*-H)(*μ*-CO)Pt(L-L)][BF_4]·0.5 H_2O, where L-L= Fe(η^5-$C_5H_4PPh_2)_2$" *Inorg. Chem.* **1989**, *28*, 404-410.

[17] G. Petöcz, Z. Berente, T. Kégl, L. Kollár, "Xantphos as cis- and trans-chelating ligand in square-planar platinum(II) complexes. Hydroformylation of styrene with platinum-xantphos-tin(II)chloride system" *J. Organomet. Chem.* **2004**, *689*, 1188-1193.

[18] R. Romeo, G. D'Amico, "Mechanistic Insight into the Protonolysis of the Pt−C Bond as a Model for C−H Bond Activation by Platinum(II) Complexes" *Organometallics* **2006**, *25*, 3435-3446.

[19] V. Scarcia, A. Furlani, B. Longato, B. Corain, G. Pilloni, "Heteropolymetallic complexes of 1,1'-bis(diphenylphosphino)ferrocene (dppf). IV. Solvolytic behavior and cytostatic properties towards the KB cell-line of dppf and 1,2-bis(diphenylphosphino)ethane *cis*-complexes of Pt(II) and Pd(II)" *Inorg. Chim. Acta* **1988**, *153*, 67-70.

[20] A. C. Durham, C. R. Liu, Y. M. Wang, "Iron-Mediated C-H Functionalization of Unactivated Alkynes for the Synthesis of Derivatized Dihydropyrrolones: Regioselectivity Under Thermodynamic Control" *Chem. Eur. J.* **2023**, *29*, e202301195.

[21] F. Rami, F. Bächtle, B. Plietker, "Hydroboration of internal alkynes catalyzed by FeH(CO)(NO)($PPh_3)_2$: a case of boron-source controlled regioselectivity" *Catal. Sci. Technol.* **2020**, *10*, 1492-1497.

[22] X. Dong, W. Jiang, D. Hua, X. Wang, L. Xu, X. Wu, "Radical-mediated vicinal addition of alkoxysulfonyl/fluorosulfonyl and trifluoromethyl groups to aryl alkyl alkynes" *Chem. Sci.* **2021**, *12*, 11762-11768.

[23] H. Zuo, E. Irran, H. F. T. Klare, M. Oestreich, "Electrophilic Activation of S-Si Reagents by Silylium Ions for Their Regio- and Diastereoselective Addition Across C-C Multiple Bonds" *Angew. Chem. Int. Ed. Engl.* **2024**, *63*, e202401599.

[24] G. Boche, U. Fährmann, "Stereospezifische Darstellung der (*Z*) ‐ bzw. (*E*) ‐ Isomeren von einigen Vinylfluoriden" *Chem. Ber.* **1981**, *114*, 4005-4009.

[25] L. Pfeifer, V. Gouverneur, "Controlled Single and Double Iodofluorination of Alkynes with DIH- and HF-Based Reagents" *Org. Lett.* **2018**, *20*, 1576-1579.

[26] S. Sander, T. Braun, "Platinum-Catalyzed Hydrofluorination of Alkynes: Hydrogen Bonding to Indolylphosphine Ligands to Provide Fluoride Reactivity" *Angew. Chem. Int. Ed. Engl.* **2022**, *61*, e202204678.

[27] J. A. Akana, K. X. Bhattacharyya, P. Muller, J. P. Sadighi, "Reversible C−F bond formation and the Au-catalyzed hydrofluorination of alkynes" *J. Am. Chem. Soc.* **2007**, *129*, 7736−7737.

[28] D. Mulryan, J. Rodwell, N. A. Phillips, M. R. Crimmin, "Au(I) Catalyzed HF Transfer: Tandem Alkyne Hydrofluorination and Perfluoroarene Functionalization" *ACS Catal.* **2022**, *12*, 3411−3419.

[29] J. Wenz, C. A. Rettenmeier, H. Wadepohl, L. H. Gade, "Catalytic C−F bond activation of geminal difluorocyclopropanes by nickel(I) complexes via a radical mechanism" *Chem. Commun.* **2016**, *52*, 202-205.

[30] L. Chausset-Boissarie, N. Cheval, C. Rolando, "Palladium-Catalyzed Cross-Coupling of Gem-Bromofluoroalkenes with Alkylboronic Acids for the Synthesis of Alkylated Monofluoroalkenes" *Molecules* **2020**, *25*.

[31] R. Guo, X. Qi, H. Xiang, P. Geaneotes, R. Wang, P. Liu, Y. M. Wang, "Stereodivergent Alkyne Hydrofluorination Using Protic Tetrafluoroborates as Tunable Reagents" *Angew. Chem. Int. Ed. Engl.* **2020**, *59*, 16651-16660.

[32] W. R. Dolbier Jr, X. X. Rong, M. D. Bartberger, H. Koroniak, B. E. Smart, Z.-Y. Yang, "Cyclization reactivities of fluorinated hex-5-enyl radicals" *J. Chem. Soc. Perkin Trans. 2* **1998**, 219-232.

[33] A. Tlili, N. Xia, F. Monnier, M. Taillefer, "A very simple copper-catalyzed synthesis of phenols employing hydroxide salts" *Angew. Chem. Int. Ed. Engl.* **2009**, *48*, 8725-8728.

[34] H. P. Mercier, M. D. Moran, G. J. Schrobilgen, C. Steinberg, R. J. Suontamo, "The syntheses of carbocations by use of the noble-gas oxidant, $[XeOTeF_5][Sb(OTeF_5)_6]$: the syntheses and characterization of the CX_3^+ (X = Cl, Br, $OTeF_5$) and $CBr(OTeF_5)_2^+$ cations and theoretical studies of CX_3^+ and BX_3 (X = F, Cl, Br, I, $OTeF_5$)" *J. Am. Chem. Soc.* **2004**, *126*, 5533-5548.

[35] E. L. Myers, C. P. Butts, V. K. Aggarwal, "$BF_3{\cdot}OEt_2$ and TMSOTf: A synergistic combination of Lewis acids" *Chem. Commun.* **2006**, 4434-4436.

[36] TURBOMOLE V7.7 2022, a development of University of Karlsruhe and Forschungszentrum Karlsruhe GmbH, 1989–2007, TURBOMOLE GmbH, since 2007; available from https://www.turbomole.org.

[37] S. G. Balasubramani, G. P. Chen, S. Coriani, M. Diedenhofen, M. S. Frank, Y. J. Franzke, F. Furche, R. Grotjahn, M. E. Harding, C. Hattig, A. Hellweg, B. Helmich-Paris, C. Holzer, U. Huniar, M. Kaupp, A. Marefat Khah, S. Karbalaei Khani, T. Muller, F. Mack, B. D. Nguyen, S. M. Parker, E. Perlt, D. Rappoport, K. Reiter, S. Roy, M. Ruckert, G. Schmitz, M. Sierka, E. Tapavicza, D. P. Tew, C. van Wullen, V. K. Voora, F. Weigend, A. Wodynski, J. M. Yu, "TURBOMOLE: Modular program suite for ab initio quantum-chemical and condensed-matter simulations" *J. Chem. Phys.* **2020**, *152*, 184107.

[38] A. D. Becke, "Density-functional exchange-energy approximation with correct asymptotic behavior" *Phys. Rev. A* **1988**, *38*, 3098-3100.

[39] J. P. Perdew, "Density-functional approximation for the correlation energy of the inhomogeneous electron gas" *Phys. Rev. B* **1986**, *33*, 8822-8824.

[40] S. Grimme, J. Antony, S. Ehrlich, H. Krieg, "A consistent and accurate ab initio parametrization of density functional dispersion correction (DFT-D) for the 94 elements H-Pu" *J. Chem. Phys.* **2010**, *132*, 154104.

[41] S. Grimme, S. Ehrlich, L. Goerigk, "Effect of the damping function in dispersion corrected density functional theory" *J. Comput. Chem.* **2011**, *32*, 1456-1465.

[42] A. Schäfer, C. Huber, R. Ahlrichs, "Fully optimized contracted Gaussian basis sets of triple zeta valence quality for atoms Li to Kr" *J. Chem. Phys.* **1994**, *100*, 5829-5835.

[43] N. Mardirossian, M. Head-Gordon, "*ω*B97M-V: A combinatorially optimized, range-separated hybrid, meta-GGA density functional with VV10 nonlocal correlation" *J. Chem. Phys.* **2016**, *144*, 214110.

[44] P. Pracht, F. Bohle, S. Grimme, "Automated exploration of the low-energy chemical space with fast quantum chemical methods" *Phys. Chem. Chem. Phys.* **2020**, *22*, 7169-7192.

[45] A. Klamt, "Conductor-like Screening Model for Real Solvents: A New Approach to the Quantitative Calculation of Solvation Phenomena" *J. Phys. Chem.* **2002**, *99*, 2224-2235.

[46] A. Klamt, V. Jonas, T. Bürger, J. C. W. Lohrenz, "Refinement and Parametrization of COSMO-RS" *J. Phys. Chem. A* **1998**, *102*, 5074-5085.

[47] A. E. Reed, R. B. Weinstock, F. Weinhold, "Natural population analysis" *J. Chem. Phys.* **1985**, *83*, 735-746.
